# Physiological and Proteomic Analyses of Molybdenum- and Ethylene-Responsive Mechanisms in Rubber Latex

**DOI:** 10.3389/fpls.2018.00621

**Published:** 2018-05-15

**Authors:** Le Gao, Yong Sun, Min Wu, Dan Wang, Jiashao Wei, Bingsun Wu, Guihua Wang, Wenguan Wu, Xiang Jin, Xuchu Wang, Peng He

**Affiliations:** ^1^Rubber Research Institute, Chinese Academy of Tropical Agricultural Sciences, Haikou, China; ^2^College of Life Sciences, Key Laboratory for Ecology of Tropical Islands, Ministry of Education, Hainan Normal University, Haikou, China; ^3^Institute of Tropical Biosciences and Biotechnology, Chinese Academy of Tropical Agricultural Sciences, Haikou, China

**Keywords:** comparative proteomics, ethylene, *Hevea brasiliensis*, molybdenum stimulation, natural rubber biosynthesis, rubber latex

## Abstract

Molybdenum (Mo) is an essential micronutrient in many plants. In the rubber tree *Hevea brasiliensis*, Mo application can reduce the shrinkage of the tapping line, decrease tapping panel dryness, and finally increase rubber latex yield. After combined Mo with ethylene (Eth), these effects become more obvious. However, the molecular mechanism remains unclear. Here, we compared the changed patterns of physiological parameters and protein accumulation in rubber latex after treated with Mo and/or Eth. Our results demonstrated that both Eth and Mo can improve the contents of thiol, sucrose, and dry yield in rubber latex. However, lutoid bursting is significantly inhibited by Mo. Comparative proteomics identified 169 differentially expressed proteins, including 114 unique proteins, which are mainly involved in posttranslational modification, carbohydrate metabolism, and energy production. The abundances of several proteins involved in rubber particle aggregation are decreased upon Mo stimulation, while many enzymes related to natural rubber biosynthesis are increased. Comparison of the accumulation patterns of 25 proteins revealed that a large portion of proteins have different changed patterns with their gene expression levels. Activity assays of six enzymes revealed that Mo stimulation can increase latex yield by improving the activity of some Mo-responsive enzymes. These results not only deepen our understanding of the rubber latex proteome but also provide new insights into the molecular mechanism of Mo-stimulated rubber latex yield.

## Introduction

Natural rubber, a very long chain of cis-1,4-polyisoprene polymer, is generated from over 2,500 plant species, but the rubber tree *Hevea brasiliensis* is the only commercially cultivated one (van Beilen and Poirier, 2007). Natural rubber is stored in a specialized laticifer cell that located in the secondary phloem of the rubber tree (Hagel et al., 2008). Rubber latex is the cytoplasm of a specialized laticifer cell, a type of cell in the phloem of the rubber tree (Hagel et al., 2008; Cho et al., 2014; Wang et al., 2015). Rubber latex contains various macromolecules, including proteins, starches, sugars, tannins, resins, and alkaloids (Wang et al., 2015), and these macromolecules are important for the properties of rubber.

In natural rubber production, latex is collected by a non-destructive regular tapping of the trunk bark of rubber tree (Tian et al., 2015). To obtain more rubber latex, an ethylene (Eth) generator, ethephon, or ethrel (chloro-2-ethyl phosphonic acid, Eth), is widely used as an effective activator to stimulate natural rubber biosynthesis (NRB; Chrestin et al., 1989; Liu, 2016). Treatment of rubber tree bark with ethephon induces a several-fold increase in the volume of latex and stimulates rubber latex regeneration between different tapping (Coupé and Chrestin, 1989). Eth can improve the stability of lutoids, which can increase rubber yield by prolonging the latex outflow time (Wang et al., 2015). However, excessive stimulation of ethephon can cause several serious adverse effects: due to strong tapping, the latex is strongly diluted, the nutrients are greatly lost along with latex outflow; metabolites are exhausted; and the contents of nucleic acids, nitrogen, and proteins are reduced (Chua, 1967). Strong tapping can also promote the accumulation of active oxygen, disturb the imbalance between peroxidation activity and scavenging activity, increase the lutoid rupture and release of coagulation factors, cause the *in situ* coagulation of latex, and ultimately result in the occurrence of tapping panel dryness (Putranto et al., 2015). This stimulation effect is associated with marked changes in both physiology and metabolism of laticifer, including the contents of dry rubber, total thiol, sucrose, Mg^2+^, and inorganic phosphorus; turgor pressure and lutoid stability; latex regeneration; sucrose transport, nucleotide acid, and protein synthesis; adenine nucleotide pools; and compartmental ion balance (Coupé and Chrestin, 1989; Lestari et al., 2017).

Several transport-related proteins, such as sucrose transporter (Dusotoit-Coucaud et al., 2010; Tang et al., 2010), aquaporin (Tungngoen et al., 2009; Tong et al., 2016), and quebrachitol transporter (Dusotoit-Coucaud et al., 2010), are associated with Eth stimulation of latex in the rubber tree. However, the gene expression levels of most enzymes involved in NRB are inhibited or unchanged upon Eth (Adiwilaga and Kush, 1996; Zhu and Zhang, 2009; Wang et al., 2015). Recently, we found that after Eth treatment, the total abundance of rubber elongation factor (REF) and small rubber particle protein (SRPP) did not change significantly, but some of their family members or protein isoforms (species) were sharply increased. Many other proteins, including hydrolase, glucanase, potassium channel protein, ribosomal protein, galactosyltransferase, myo-inositol-1-phosphate synthase, and several kinase receptors were all induced by Eth stimulation (Wang et al., 2015).

Molybdenum (Mo), an essential microelement in plants, plays an important role in nitrate assimilation, sulfite detoxification, purine metabolism, and the synthesis of abscisic acid, auxin, and glucosinolate (Qin et al., 2017). Mo-nutrition affects many aspects of the physiological and biochemical of plants, such as chlorophyll content (Kumchai et al., 2013; Wu et al., 2014; Hadi et al., 2016), total dry matter and biomass (Kaushik and Govil, 2001; Wu et al., 2014; Liu et al., 2017), proline content (Kumchai et al., 2013; Hadi et al., 2016), ABA and IAA contents (Walker-Simmons et al., 1989), soluble proteins (Kaushik and Govil, 2001), Mo-enzyme activity (Wang et al., 1999; Kaufholdt et al., 2016; Liu et al., 2017), and antioxidant enzymes (Wu et al., 2014). In the rubber tree, ammonium molybdate application increases rubber latex yield and inhibits the activity of acid phosphatase in the lutoids, as well as helps to increase and preserve the concentrations of the energy molecule ATP and reducing agent NADPH (Ribaillier, 1968).

In our preliminary study, we noticed that application of ammonium molybdate on the tapping panel of rubber tree bark can improve rubber latex yield, and this stimulation effect becomes more obvious when mixed Mo with Eth. Here, our interest is focused on the proteomic mechanism of Eth and ammonium molybdate on the stimulation of rubber latex yield. Our results showed that Eth and ammonium molybdate can improve rubber latex yield by regulating the accumulation of proteins involved in rubber particle aggregation (RPA), latex coagulation, rubber biosynthesis, and antioxidant response.

## Materials and Methods

### Plant Material and Treatment

A total of 48 matured (∼8-year-old) and newly tapped rubber trees (*H. Brasiliensis* Mull. Arg., clone RY 7-33-97) that were never treated with Eth were selected and randomized into four groups. These plants were grown at an experimental farm of the Chinese Academy of Tropical Agricultural Sciences in Danzhou City, Hainan Province, China. Different treatments were applied on the tapping panel as described (Doungmusik and Sdoodee, 2012). For Eth treatment, 0.5% (V/V) ethephon was dissolved in ddH_2_O (termed Eth hereafter), 1.5% (W/V) ammonium molybdate was dissolved in ddH_2_O as Mo treatment (termed Mo hereafter), and 0.5% (V/V) ethephon and 1.5% (W/V) ammonium molybdate were dissolved in ddH_2_O as the combined treatment (EMo). An equal volume of ddH_2_O was used as the control (CK). After treatment for 1 day (24 h), these plants were tapped to collect fresh latex. Three biological replicates were performed for each treatment. After tapping, the first 20 drops were discarded. Subsequent latex drops were collected in ice-chilled glass beakers for further analysis.

### Determination of Lutoid Bursting Index, Dry Rubber, Thiol, and Sucrose Contents

The latex lutoid bursting index (LBI), thiol, and sucrose contents were determined immediately after tapping. LBI was determined as described (Yeang, 1986). For dry rubber content, the collected fresh latex was solidified with 5% acetic acid, then washed with tapped water, and dried at 80°C for 3 days. The thiol (Boyne and Ellman, 1972) and sucrose (Dubois et al., 1956) contents were determined as described.

### Protein Extraction and Two-Dimensional Gel Electrophoresis (2-DE)

Total latex proteins were extracted as described (Wang et al., 2010). Protein concentration was determined by Bradford assay with BSA as a standard. For 2-DE, 1,300 μg proteins were loaded onto 24-cm, pH 3-10 linear gradient IPG strips (GE Healthcare, Uppsala, Sweden), and isoelectric focusing and gel electrophoresis were performed as described (Wang et al., 2013). Three biological replicates were performed for each sample. Protein spots in gel were visualized by the CBB-G250 staining, and images were analyzed with the ImageMaster 2D Platinum software (GE Healthcare). The average Vol% for each matched protein spot was calculated based on all the detected gel images from the three biological repeats (**Supplementary Figure S1**). First, we compared Eth, Mo, and EMo with CK, as well as EMo with Eth and EMo with Mo; the protein spots that, in at least one pair, showed an at least 1.5-fold change in abundance (*p* < 0.05) were termed as differentially expressed protein (DEP) spots. Then, these protein spots were positively identified by MALDI TOF MS/MS.

### Protein Identification via Mass Spectrometry

The DEP spots were manually excised and in-gel digested with bovine trypsin as described (Wang et al., 2010, 2015). Mass spectra of the peptides were acquired on an AB 5800 MALDI-TOF/TOF mass spectrometry (MS) instrument (AB SCIEX, Foster City, CA, United States) as described (Wang et al., 2015). The measured tryptic peptide masses were searched using ProteinPilot software (version 5.0) with an in-house MASCOT server against a self-constructed database derived from the original *H. brasiliensis* genome scaffolds (BioProject ID: PRJNA80191^1^) and the draft genome (GenBank: AJJZ01000000) as described (Tang et al., 2016); this database included 46,718 sequences and 17,435,757 residues. Positively identification of protein spots was with scores higher than 65 (*p* < 0.001, the threshold with 95% confidence was 31), at least two matched peptides, and a minimum peptide coverage greater than 7%. Detailed identification information was provided in **Supplementary Table S1** and **Supplementary Figure S2**.

### Protein Classification and Hierarchical Analysis

The identified proteins were searched against the UniProt database to confirm their functions. Blast2GO software was used to determine the proteins involved in KEGG pathway and GO information for cellular component, biological process, and molecular function. Functional catalog software was used to obtain the corresponding COG codes. Hierarchical clustering of the protein abundant profiles was performed using TreeView software (version 1.1.6).

### RNA Extraction and qRT-PCR Analysis

Latex RNA was isolated as described (Tang et al., 2010). Primer pairs used in qRT-PCR were provided in **Supplementary Table S3**. The gene expression level was normalized by the gene of ubiquitin-conjugating enzyme E2B (UBC2B) as an internal control. The relative gene expression levels were calculated by the 2^-ΔΔ^*^C_T_^* method (Livak and Schmittgen, 2001).

### Western Blotting Analysis

Western blotting was performed as described (Wang et al., 2015). Approximately 30 μg proteins were loaded per lane. After electrophoresis, the proteins were transferred from the gel onto a polyvinylidenedifluoride (PVDF) membrane under semi-dry conditions and by applying electric current. Blocking was performed with 5% non-fat milk, after PVDF membrane was incubated with the antibody obtained from rabbit blood. The bound rabbit IgG was detected in separate blots with horseradish peroxidase (HRP) conjugated with anti-rabbit IgG and Clarity Western ECL substrate (Bio-Rad, CA, United States). To validate protein expression levels, three biological replicates were performed for each protein.

### Enzyme Activity Assay

Fresh latex was centrifuged at 20,000 × *g* for 60 min at 4°C, and the middle layer was collected to further centrifuge at 34,000 × *g* for 60 min at 4°C as purified C-serum for assaying enzyme activity. The activities of superoxide dismutase (SOD), ascorbate peroxidase (APX), and glutathione reductase (GR) were determined as described (Yu et al., 2011). The activities of nitrate reductase (NR; Foyer et al., 1998), aldehyde oxidase (AO), and xanthine dehydrogenase (XDH; Sagi et al., 1999) were assayed as described.

### Statistical Analysis

All results are presented as the means ±*SE* of at least three replicates. Data were analyzed by one-way ANOVA using the statistical software SPSS 18.0 (SPSS Inc., Chicago, IL, United States). The treatment mean values were compared by least significant difference *post hoc* test. A *p* value less than 0.05 was considered statistically significant.

## Results

### Mo and Ethylene Can Significantly Improve Rubber Latex Yield

**FIGURE 1:**
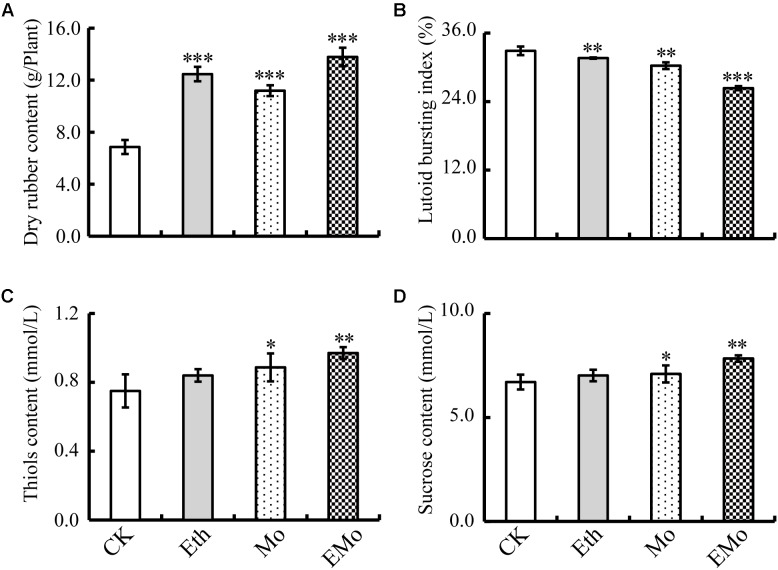
Changes in rubber latex parameters under different treatments. The dry rubber content **(A)**, lutoid bursting index **(B)**, thiol content **(C)**, and sucrose content **(D)** in the latex collected from rubber trees treated with control (CK), 0.5% ethylene (Eth), 1.5% ammonium molybdate (Mo), and 0.5% Eth +1.5% ammonium molybdate (EMo) were highlighted. Values are the mean ±*SD* (^∗^*P* ≤ 0.05, ^∗∗^*P* ≤ 0.01, and ^∗∗∗^*P* ≤ 0.001).

It is widely known that Eth stimulation can significantly improve rubber latex yield. Here, our results demonstrated that both Eth and Mo treatments could improve the dry content of rubber latex. The highest value (13.8 g/plant) was detected in the EMo treatment (**Figure 1A**). Some parameters of rubber latex were also examined to determine the stimulation effects of Eth and Mo. Both Eth and Mo inhibited the bursting of lutoid in latex and generated a significantly decreased LBI. In CK, LBI was 32.9%, and this value was decreased to 26.3% after EMo treatment (**Figure 1B**). A low lutoid bursting rate can reduce lutoid-mediated RPA and sequential latex coagulation, which helps to maintain latex flow and generate much more rubber latex (Wang et al., 2015). Both thiol (**Figure 1C**) and sucrose (**Figure 1D**) contents were significantly improved under Mo and EMo treatments, but these values were not changed significantly if only Eth was applied.

### Identification of Differentially Expressed Proteins in Rubber Latex Under Eth and Mo Treatments

**FIGURE 2:**
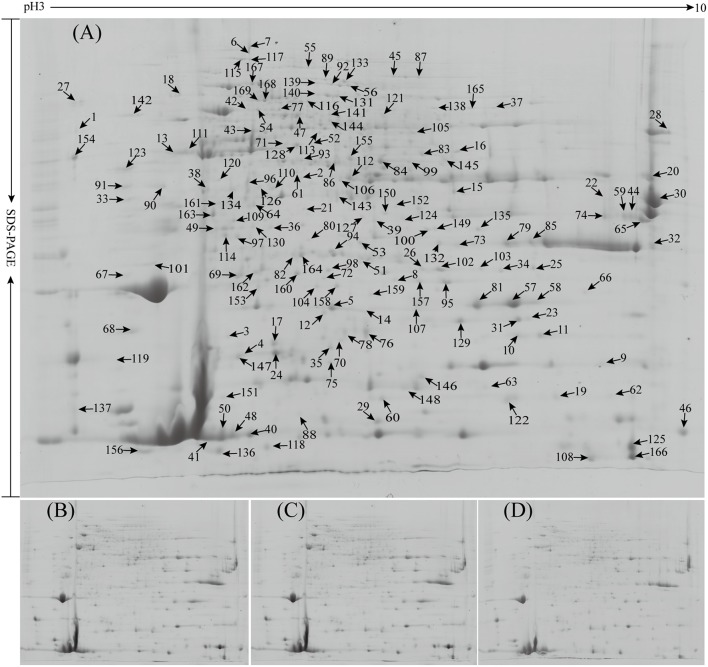
Proteome profiles of rubber latex under different treatments. Total latex proteins from rubber trees treated with the control (CK, **A**), 0.5% ethylene (Eth, **B**), 1.5% ammonium molybdate (Mo, **C**), and 0.5% Eth combined with 1.5% ammonium molybdate (EMo, **D**) were separated by 2-DE, and the positively identified DEPs in the 2-DE gels were marked with numbers (from spot 1 to 169). The protein identities are listed in **Supplementary Figure S2** and **Supplementary Table S1**.

To investigate the DEPs upon different treatments, the protein profiles of rubber latex under four treatments (CK, Eth, Mo, and EMo) were obtained using 2-DE analysis with pH 3–10, 24-cm IPG strips. On Coomassie Brilliant Blue-G250 stained gels, 1,200 ± 58, 1,188 ± 39, 1,174 ± 39, and 1,176 ± 48 spots were detected in the latex from CK, Eth, Mo, and EMo treatments, respectively (**Figure 2**). After gel image analysis, based on the calculated average Vol% of each matched protein spot, 169 DEPs showed an at least 1.5-fold change in abundance (*p* < 0.05) were positively identified by MALDI TOF MS/MS. These DEPs contained 114 unique proteins (**Supplementary Figure S2** and **Supplementary Table S1**).

Compared to the untreated plants, Eth produced 59 DEPs, including 39 reduced protein species and 20 induced ones. Among them, the top five reduced proteins were uncharacterized protein (Spot 1, termed S1 hereafter), aldose 1-epimerase (ADEP; S2), ADP-ribosylation factor GTPase-activating protein (S3), pro-hevein (PH; S4), and osmotin-like protein OSM34 (OSM; S5); the top five induced proteins were pyridoxal biosynthesis protein PDX1 (S39), uncharacterized protein Os08g0359500 (S40), REF (S41), importin subunit alpha-1 (S42), and actin-7 (S43; **Figure 2A** and **Supplementary Table S1**). Under Mo treatment, 81 protein species were reduced, but only 13 induced proteins were observed. Among them, the top five reduced proteins were ATP-citrate synthase alpha chain protein 2 (S113), patatin-like protein 2 (PLP2; S13), OSM (spots 57 and 58), and elongation factor 1-delta (EF1; S33); the top five induced proteins were cysteine proteinase inhibitor 12 (CPI12; S102), pyridoxal biosynthesis protein PDX1 (S39), chlorophyllase type 0 (S148), abscisic stress-ripening protein 1 (S159), and proteasome subunit alpha type-5 (S101). For the combined Eth- and Mo- treated samples, 48 reduced and 19 induced protein species were determined in EMo. The top five reduced protein species in EMo are proteasome subunit beta type-4 (S25), ADEP (S2), cysteine synthase (S64), REF (S151), and ubiquitin-conjugating enzyme E2 35 (UBCE; S60); the top five EMo-induced proteins are CPI12 (S102), prefoldin subunit 5 (PFS5; S62), REF (S48), malignant T-cell-amplified sequence 1 (S66), and SOD (S158) (**Figure 2A** and **Supplementary Table S1**). Interestingly, among the 114 identified unique proteins, 27 had multi-protein forms, and six members contained more than four protein species or isoforms. They are hevamine-A (scaffold0143_850373.mRNA1, spots 32, 73, 79, 80, 85, and 132), PH (scaffold0155_515853.mRNA1, spots 4, 17, 24, and 29), glucan endo-1,3-beta-glucosidase (GEBG; scaffold0625_1132.mRNA1, spots 44, 59, 65, and 74), REF (scaffold1222_136753.mRNA1, spots 18, 38, 41, 46, 48, 50, 88, 108, 125, 151, and 166), acetyl-CoA acetyltransferase (ACAT, scaffold1479_76107.mRNA1, spots 52, 84, 99, and 155), and osmotin (scaffold4512_855.mRNA1, spots 5, 14, 57, 58, 63, 81, and 107; **Figure 2A** and **Supplementary Table S1**). These protein species might be generated from alternative splicing and various post-translational modifications.

### Hierarchical Clustering of the Changed Patterns of the Differentially Expressed Proteins

**FIGURE 3:**
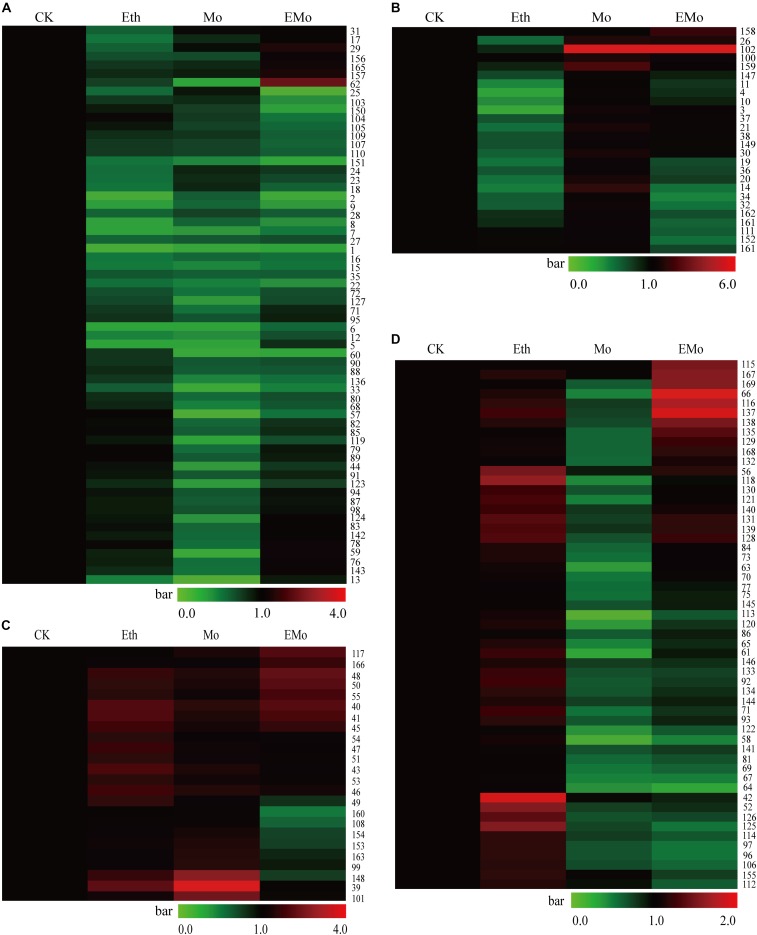
Cluster of the identified DEPs among the different treatments. A hierarchical cluster of the identified 169 DEPs is presented. In cluster **(A)**, the proteins were reduced after Eth and Mo treatments. In cluster **(B)**, the proteins were reduced by Eth but induced by Mo. In cluster **(C)**, the proteins were induced after Eth and Mo treatments. In cluster **(D)**, the proteins were reduced by Mo but induced by Eth.

To better understand the protein accumulation patterns after treatment, hierarchical clustering analysis was performed on the 169 DEPs, resulting in four main clusters (**Figure 3**). Cluster 1 contained 64 DEPs, which were all reduced in both the Eth and Mo treatments, and only several of them were induced in the EMo-treated condition (**Figure 3A**). These proteins are mainly involved in protein synthesis and degradation (e.g., elongation factor, heat shock proteins, proteasome subunits, and ubiquitin-conjugating enzyme), carbohydrate metabolism (e.g., triosephosphateisomerase, GEBG, and hevamine-A), defense and antioxidant, and biosynthesis of secondary metabolites. The second cluster contained 26 DEPs, which were reduced in Eth but induced in Mo treatment, and some of them were induced in EMo (**Figure 3B**). It is noteworthy that CPI12 (S102, scaffold0017_613025.mRNA1) had a very high abundance (approximately sixfold higher than the CK) in the Mo and EM treatments. These proteins are mainly involved in biosynthesis of secondary metabolites (e.g., PH, REF, and SRPP), protein degradation, and defense and antioxidant (**Figure 3B**). The third cluster contained 24 DEPs, which were induced in both Eth and Mo treatments, and most of them were also induced in EMo (**Figure 3C**). They are mainly involved in biosynthesis of secondary metabolite and carbohydrate metabolism. Cluster 4 had 55 DEPs, which were induced in Eth and reduced in Mo, and some of them were reduced or induced in EMo. They are mainly involved in biosynthesis of secondary metabolites, carbohydrate metabolism, and protein degradation (**Figure 3D** and **Supplementary Table S1**).

Venn diagram analysis of the identified 169 DEPs from the rubber latex treated with Eth, Mo, and EMo revealed that a large portion of the proteins (95 protein species) were Mo-responsive proteins. Among them, 51 protein species were responded solely to Mo application. After treatment with Eth or EMo, only 23 protein species were identified. Only 15 proteins were identified as DEPs among the three treatments (**Supplementary Figure S3A**). For the 52 induced or upregulated protein species, 12, 8, and 11 were responded to Eth, Mo, and EMo, respectively. Only an uncharacterized protein Os08g0359500 (S40, scaffold0166_1819893.mRNA1) was always induced under the three kinds of treatments (**Supplementary Figure S3B**). Compared to the upregulated proteins, more downregulated protein species were detected. There were 17, 51, and 16 proteins in the latex after treatment with Eth, Mo, and EMo, respectively (**Supplementary Figure S3C**).

### Functional Analysis of the Differentially Expressed Proteins

**FIGURE 4:**
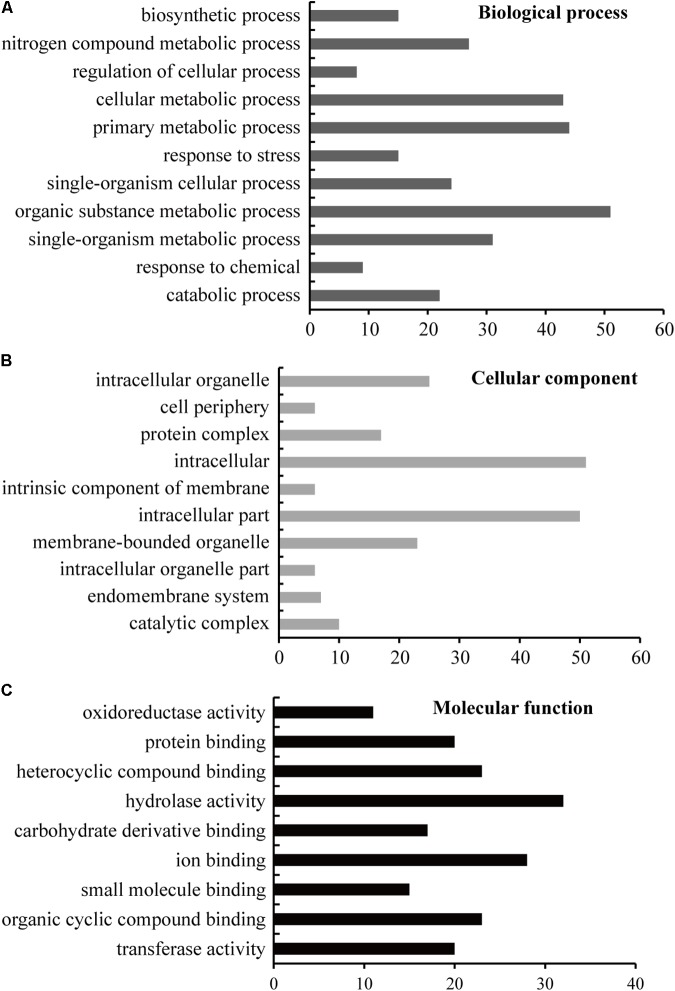
GO enrichment analysis of the identified proteins. The sequence of the identified 114 unique DEPs was lorded into Blast2GO software for blast, mapping, and InterProScan analysis to obtain GO information at the biological process **(A)**, cellular component **(B)**, and molecular function **(C)** levels; level 3 was selected as the final results. The detailed GO information is listed in **Supplementary Table S2**.

GO distribution analysis of all DEPs was performed by using Blast2GO software to confirm the biological process, cellular component, and molecular function (**Figure 4**). In biological process, the largest proportion, including 51 unique proteins, was in the organic substance metabolic process (GO: 0071704), 44 unique proteins occur in primary metabolic process (GO: 0044238), 43 proteins are in cellular metabolic process, and 31 proteins are in single-organism metabolic process, followed by nitrogen compound metabolic process (GO: 0006807), single-organism cellular process, catabolic process, response to stress, biosynthetic process, response to chemical, and regulation of cellular process (**Figure 4A** and **Supplementary Table S2**). In cellular component, the largest part, including 51 unique proteins, is in intracellular (GO: 0005622); the second part, including 50 unique proteins, is in intracellular part (GO: 0044424); the third part, including 25 unique proteins, is in intracellular organelle (GO: 0043229), followed by membrane-bounded organelle, protein complex, catalytic complex, endomembrane system, intracellular organelle part, intrinsic component of membrane, and cell periphery (**Figure 4B** and **Supplementary Table S2**). For molecular function ontology, 32 unique proteins have hydrolase activity (GO: 0016787), 28 unique proteins have ion binding ability (GO: 0043167), and 23 unique proteins have heterocyclic compound binding (GO: 1901363), followed by organic cyclic compound binding activity, transferase activity, protein binding activity, carbohydrate derivative binding activity, small molecule binding activity, and oxidoreductase activity (**Figure 4C** and **Supplementary Table S2**).

**FIGURE 5:**
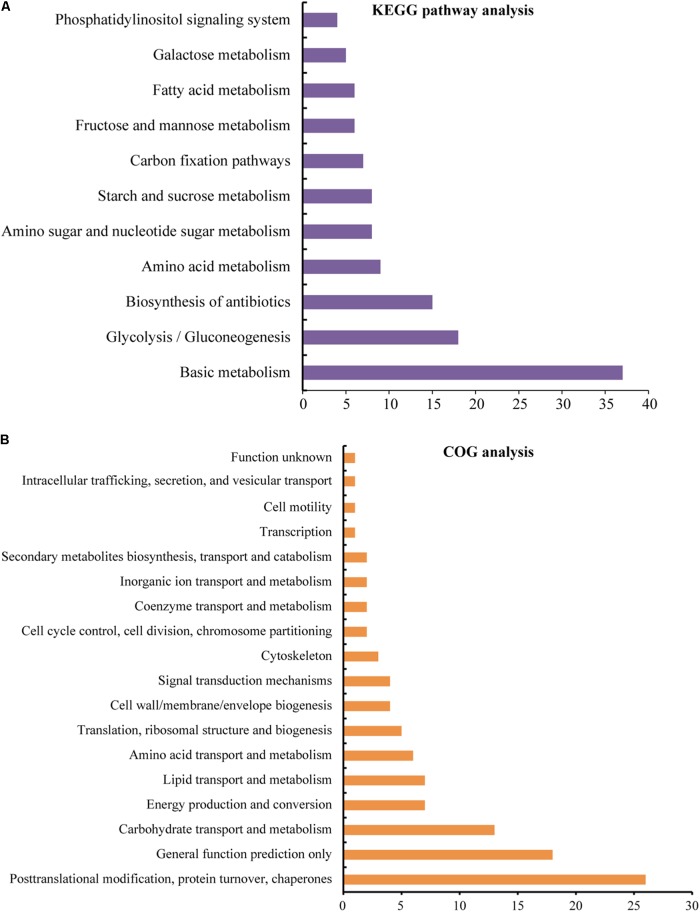
KEGG pathway and COG functional analyses of the identified proteins. The sequence of the identified 114 unique DEPs was also analyzed by Blast2GO for KEGG pathways **(A)** analysis and performed by WebMGA to obtain COG functions **(B)**; the e-value was 0.001. The detailed information is listed in **Supplementary Table S2**.

To determine the detailed KEGG pathways and COG functional category (Cluster of Orthologous Groups of proteins) of the identified 114 unique proteins, we used Blast2GO software to perform KEGG pathway analysis and used WebMGA for COG analysis. In the KEGG analysis, these proteins were involved in 54 pathways (**Supplementary Table S2**), and 11 main pathways were enriched (**Figure 5A** and **Supplementary Table S2**). These pathways are involved in basic metabolism (37 proteins), glycolysis/gluconeogenesis (18 proteins), biosynthesis of antibiotics (15 proteins), amino acid metabolism (nine proteins), amino sugar and nucleotide sugar metabolism (eight proteins), starch and sucrose metabolism (eight proteins), carbon fixation pathways (seven proteins), fructose and mannose metabolism (six proteins), fatty acid metabolism (six proteins), galactose metabolism (five proteins), and phosphatidylinositol signaling system (four proteins). For COG analysis, 94 out of the 114 unique proteins have functional information, and they were classified into 18 categories. Among them, five unique proteins [glucose and ribitol dehydrogenase (GRDH), calmodulin-related protein, calcium-binding protein CML49 (CBP), dehydrogenase, and urease accessory protein G] have multi-COG categories, and the main categories include post-translational modification, protein turnover, chaperones (26 unique proteins), general function prediction, carbohydrate transport and metabolism (13 unique proteins), energy production and conversion (seven unique proteins), lipid transport and metabolism, amino acid transport and metabolism, and translation, ribosomal structure, and biogenesis (**Figure 5B** and **Supplementary Table S2**).

### Comparison of Protein Accumulation and Gene Expression Patterns of Some Typical DEPs

**FIGURE 6:**
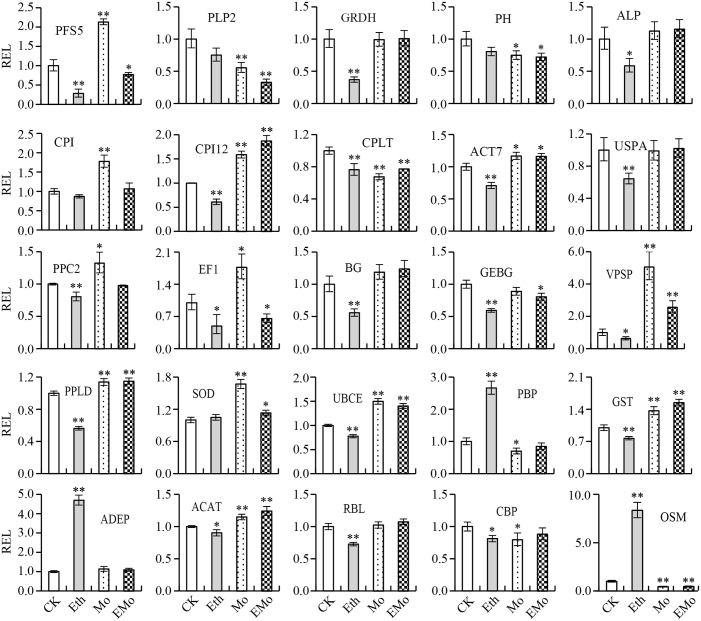
Gene expression analysis of the typical proteins from rubber latex. The 25 typical proteins of DEPs were performed by qRT-PCR analysis to determine their corresponding gene expression patterns. Values are the mean ±*SD* (^∗^*P* ≤ 0.05 and ^∗∗^*P* ≤ 0.01). REL, relative expression level; PFS5, prefoldin subunit 5; PLP2, patatin-like protein 2; GRDH, glucose and ribitol dehydrogenase; PH, pro-hevein; ALP, annexin-like protein; CPI, cysteine proteinase inhibitor; CPI12, cysteine proteinase inhibitor 12; CPLT, chlorophyllase type 0; ACT7, actin-7; USPA, universal stress protein A-like protein; PPC2, phosphoinositide phospholipase C 2; EF1, elongation factor 1-delta; BG, beta-glucosidase; GEBG, glucan endo-1,3-beta-glucosidase; VPSP, vacuolar protein sorting-associated protein 2; PPLD, phospholipase D alpha 1; SOD, superoxide dismutase; UBCE, ubiquitin-conjugating enzyme E2; PBP, pyridoxal biosynthesis protein PDX1; GST, glutathione S-transferase; ADEP, aldose 1-epimerase; ACAT, acetyl-CoA acetyltransferase; RBL, ricin B-like lectin; CBP, calcium-binding protein; OSM, osmotin. The primers used for qRT-PCR are provided in **Supplementary Table S3**.

To further evaluate the correlation between protein abundance and transcript level, 25 unique proteins were performed by qRT-PCR analysis to determine their corresponding gene expression patterns. The protein abundance fold changes of these 25 proteins were higher among all of the DEP spots in at least one treatment. These 25 unique proteins play important roles in multi-pathways, such as nature rubber biosynthesis, including PLP2, PH, beta-glucosidase (BG) 42, GEBG, ACAT, ricin B-like lectin (RBL) EULS3, OSM, and actin-7 (ACT7); signal conduction system, including phosphoinositide phospholipase C 2 (PPC2), phospholipase D alpha 1 (PPLD), and CBP; protein synthesis and degradation, including PFS5, cysteine proteinase inhibitor (CPI), CPI12, EF1, and UBCE; and antioxidant system, including SOD [Mn] and glutathione S-transferase PARB (GST). The cooperation of these proteins may ultimately induce the rubber yield, so analysis of the RNA expression level of these proteins will help us to better understand the role of these proteins in this process. Among them, 11 members were identified from different spots (**Figure 2A** and **Supplementary Table S1**) and had multiple protein species (**Supplementary Table S3**). The primer pairs were designed according to the gene sequence of each unique protein (**Supplementary Table S3**). The gene expression levels of the 25 unique proteins were evaluated (**Figure 6** and **Supplementary Table S3**). The results demonstrated that for the 11 unique proteins with multiple protein isoforms, three proteins, named PLP2 (spots 13 and 111), PH (spots 4, 17, 24, and 29), and chlorophyllase type 0 (CPLT, spots 76, 78, and 148), appeared similar expressional patterns for their protein abundance and gene expression level. Three proteins, named GEBG (spots 20, 22, and 30), RBL (spots 23, 31, and 129), and GRDH (spots 96 and 110) showed similar trends at certain treatments for protein and gene accumulation. However, the other five proteins, including CPI (spots 118 and 136), ACT7 (spots 6, 7, and 43), PPC2 (spots 168 and 169), ACAT (spots 52, 84, 99, and 155), and OSM (spots 5, 14, 57, 58, 63, 81, and 107), appeared opposite expression trends with their gene expression levels (**Figure 6** and **Supplementary Table S3**).

For the 14 unique proteins identified from only one spot, three proteins named PFS5 (S62), BG (S77), and ADEP (S2), showed opposite gene expression trends with protein. Eight proteins (annexin, elongation factor 1, phospholipase D, etc.) showed similar trends with their gene levels under some treatments. Three proteins (CPI12, vacuolar protein sorting-associated protein 2, and SOD) had similar expression patterns with their gene levels (**Figure 6** and **Supplementary Table S3**). These results revealed that a large portion of proteins had different changed patterns with their gene expression levels.

### Western Bolt and Enzyme Activity Assays of Some Mo-Responsive Proteins

**FIGURE 7:**
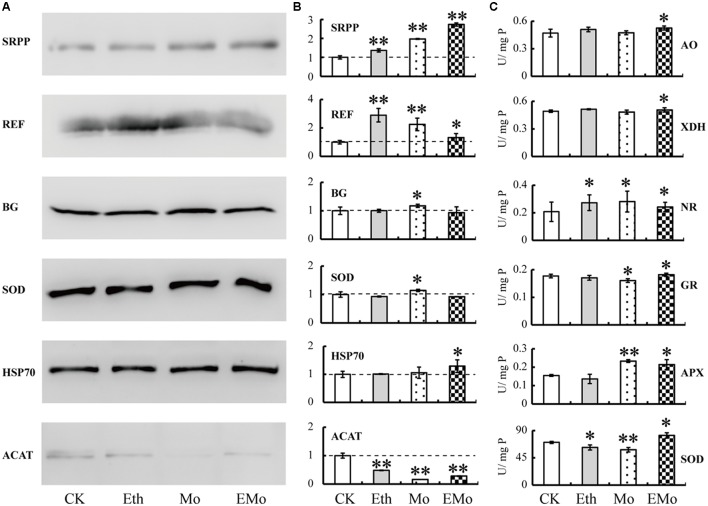
Western blotting and enzyme activity assay of Mo-responsive enzymes. The accumulation patterns of six DEPs from rubber latex under different treatments (Eth, Mo, and EMo) were examined by Western blotting **(A)**. The means of the relative abundance to CK were calculated from three independent experiments. Values are the mean ± *SD* (^∗^*P* ≤ 0.05 and ^∗∗^*P* ≤ 0.01). The dotted line represents the relative abundance value in CK **(B)**. The enzyme activities of six Mo-related enzymes were examined **(C)**. U/mg *P* value represents the specific activity of the enzyme and also means the number of enzyme activity units in unit protein. Values are the mean ± *SD* (^∗^*P* ≤ 0.05 and ^∗∗^*P* ≤ 0.01). SRPP, small rubber particle protein; REF, rubber elongation factor; BG, beta glucosidase; SOD, superoxide dismutase; HSP70, heat-shock 70 kDa protein; ACAT, acetyl-CoA acetyltransferase; APX, ascorbate peroxidase; GR, glutathione reductase; AO, aldehyde oxidase; XDH, xanthine dehydrogenase; NR, nitrate reductase.

The 2-DE results only showed the protein spot abundance (usually one protein isoform changed its abundance), and Western blotting showed the whole protein expression level. Thus, we performed Western blotting to determine the general accumulation patterns of six Mo-responsive proteins involved in NRB (REF, SRPP, and ACAT) and latex flow (BG, SOD, and HSP70; **Figure 7A**), and their relative abundance was calculated (**Figure 7B**). Among them, the abundance of five detected proteins was changed significantly after treatment with Mo, but two proteins, named BG and HSP70, did not significantly change when treated with Eth or Mo. Two proteins (SRPP and REF) were significantly induced and one protein (ACAT) was reduced by Eth. After treatment with EMo, the abundances of three proteins, named SRPP, REF, and HSP70, were significantly induced, but the accumulation of ACAT was significantly decreased (**Figures 7A,B**).

The activity of six enzymes, including three Mo-related enzymes, named NR, XDH, and AO, and three antioxidant enzymes, named SOD, APX, and GR, were further examined (**Figure 7C**). As a necessary nutrient element in higher plants, Mo is mainly recognized by Mo-containing enzymes. NR is a molybdo-enzyme that reduces nitrate (NO^3-^) to nitrite (NO^2-^). This reaction is critical for protein production in most crop plants, as nitrate is the predominant source of nitrogen in fertilized soils. XDH belongs to the group of Mo-containing hydroxylases that are involved in the oxidative metabolism of purine; this enzyme can be converted into xanthine oxidase by reversible sulfhydryl oxidation or by irreversible proteolytic modification. AO is a member of the molybd-flavo protein family. For the three Mo-related enzymes (AO, XHD, and NR), all of their activities were significantly induced after EMo treatment, but only NR was also induced after Eth and Mo treatments. Compared to CK, the activities of the three antioxidant enzymes were all induced under the three treatments, but only the activity of APX was upregulated in Mo. For the three antioxidant enzymes, their activities changed little in the different treatments compared to CK, and the activity of SOD was significantly reduced after treatment with Eth (**Figure 7C**).

## Discussion

### The First Protein Profiles in Rubber Latex About Mo Nutrition

Recently, many proteins had been identified by proteomics-based technologies from different Hevea latex components, such as total latex (D’Amato et al., 2010; Li et al., 2011; Wang et al., 2015; Dai et al., 2016; Habib et al., 2017), rubber particles (Rojruthai et al., 2010; Xiang et al., 2012; Dai et al., 2013, 2016; Wang et al., 2016; Yamashita et al., 2016), C-serum (Wagner et al., 2007; Wang et al., 2010; Havanapan et al., 2016), and lutoids (Wang et al., 2010, 2013; Habib et al., 2017). Proteins involved in mevalonate (MVA) pathway are crucial for NRB (Wang et al., 2015).

It is well known that latex regeneration is an important limiting factor for rubber production, and plant nutrition supplication is an indispensable key factor (Chao et al., 2015; Long et al., 2015). In rubber production, Mo nutrition is important for increasing rubber latex yield if it is used on the tapping panel of rubber tree, and Mo can also reduce the undesirable effects caused by Eth over-application, but the mechanism remains unclear (d’Auzac et al., 1997). Here, we performed the first comparative proteomics and identified 169 Mo- and Eth-related proteins in rubber latex. In past proteomic studies of rubber latex, the pH 4–7 linear gradient IPG strips were used for protein separation, but we noticed that rubber latex also contained many alkaline proteins, usually in lutoids (Wang et al., 2013). Therefore, in this work, we used pH 3–10 linear gradient IPG strips to perform 2-DE, and obtained the first and quality protein profiles of rubber latex under Mo-nutrition application. In the 2-DE gels, we found several hundred alkaline proteins, and some of them were also identified as DEPs; they were mainly involved in rubber biosynthesis or RPA. These proteins included elongation factor 1-alpha, GEBG, hevamine-A, PH, osmotin, and several REF isoforms (**Figure 1** and **Supplementary Table S1**).

### Mo Can Change the Accumulation of Many Proteins Involved in Rubber Particle Aggregation and Rubber Biosynthesis

Ethylene stimulation of latex yield in virgin trees might result from a coordinated regulation of two internal limiting factors. The first is latex dilution, which determines the duration of latex outflow time, and the second is rubber biosynthesis. For the first factor, this process may depend on the turgor pressure of laticifer cells (Dusotoit-Coucaud et al., 2009) and latex coagulation (Wang et al., 2013). For turgor pressure of laticifers, upregulation of a quebrachitol transporter (HbPLT2; Dusotoit-Coucaud et al., 2010), two aquaporins (HbPIP2;1 and HbTIP1; Tungngoen et al., 2009), and osmotin (Tong et al., 2016) may play important roles. Osmotin is an abundant cationic multifunctional protein adapted to environments of low osmotic potential (Abdin et al., 2011). In rubber trees, osmotin is located at lutoids, but its protein abundance and gene expression can be regulated by Eth application (Tong et al., 2016). In regularly tapped rubber trees, *HbOsmotin* expression was drastically inhibited in rubber latex after tapping, although the expression was subsequently recovered by Eth stimulation. However, in virgin plants, exogenous Eth application slightly decreased *HbOsmotin* expression. *HbOsmotin* overexpression in *Arabidopsis* ultimately reduced the osmotic stress tolerance by raising the water potential in plants (Tong et al., 2016). In this work, seven osmotin species were identified (**Figure 2** and **Supplementary Table S1**), most of their abundance decreased after Eth or Mo treatment, but the gene expression level was more than eightfold higher in Eth and reduced in Mo and EMo treatments (**Figure 6** and **Supplementary Table S3**), indicating that Mo can inhibit the protein accumulation and gene expression of osmotin in rubber latex. Lutoid rupture is the main reason for latex coagulation; after rupture, the lutoid can release lots of cations, acid hydrolases, oxidoreductases, hevein, and lectin, and ultimately cause RPA (Wang et al., 2013). In this study, we also noticed that the LBI was reduced under all three treatments (**Figure 1B**). The results suggested that Mo treatment can decrease the protein accumulation and gene expression of osmotin, and reduce the LBI, thus helping to reduce lutoid rupture and prolong latex outflow time.

Maintaining high outflow speed and time after tapping is a guarantee for latex production. The formation of the protein network is important for stopping latex flow and improving RPA (Wu et al., 2014). In a new model of lutoid-mediated RPA and latex coagulation, we concluded that hevein and glucanase can induce RPA, whereas chitinase can obviously inhibit this process, and the addition of chitinase inhibits the RPA process that is induced by hevein. If hevein, glucanase, and chitinase are simultaneously released after lutoid burst, they act as positive activators of RPA (Wang et al., 2013). In this proteomic study, two homology proteins of GEBG were reduced in Eth and EMo but induced by Mo. The isoelectric point of these two homology proteins is differential, and the molecular weight is similar. One protein (scaffold0625_11329.mRNA1) showed a higher abundance than the other. However, its gene expression was inhibited under all treatments (**Figure 6**). PH, as the precursor of hevein, contains a hevein-like N-terminal domain (Soedjanaatmadja et al., 1995). Both hevein and PH have high chitin-binding activity and can interact selectively and non-covalently with chitin, which is similar to lectin. In our proteomic results, seven PH protein species were identified, and the protein abundance and gene expression were reduced under all treatments. Six protein spots were identified as hevamine-A (scaffold0143_850373.mRNA1), also named chitinase (Wititsuwannakul et al., 2008). Three protein spots were identified as lectin, and their abundance was decreased under Eth and Mo treatments and almost did not change in EMo (**Figure 2** and **Supplementary Table S1**). In summary, the positive regulation proteins of RPA were all reduced, and the negative regulation protein of RPA was not changed under all treatments. Our results revealed that Mo combined with Eth can inhibit RPA and latex coagulation, and thus improve the rubber latex yield.

Sucrose is the unique precursor of NRB. Proteins involved in sucrose synthesis and metabolism also play important roles in rubber biosynthesis (Chow et al., 2007). Here, several enzymes involved in sucrose metabolism were identified as DEPs (**Figure 2** and **Supplementary Table S1**). Upregulation of these proteins may increase sucrose metabolism to provide enough substrates for NRB. Among them, phosphoglyceratemutase catalyzes the internal transfer of a phosphate group from C-3 to C-2, which results in the conversion of 3-phosphoglycerate (3PG) to 2-phosphoglycerate (2PG) through a 2,3-bisphosphoglycerate intermediate (Johnsen and Schonheit, 2007). After Eth and EMo treatments, this protein was upregulated to supply more substrate for sucrose metabolism. Pyrophosphate–fructose 6–phosphate 1–phosphotransferase (spots 138 and 165) catalyzes the reversible conversion of fructose 6-phosphate and fructose 1,6-bisphosphate. Upregulation of this enzyme will increase the substrate supply of rubber biosynthesis under EMo treatment. As an oxaloacetate decarboxylase in eukaryotes, acylpyruvase (S26) is involved in the conversion of oxaloacetate into pyruvate (Pircher et al., 2015). Upregulation of this enzyme after Mo and EMo treatments will provide more pyruvate. ATP citrate synthase (spots 37 and 113) catalyzes the chemical reaction ADP to form ATP, and downregulation of ATP synthase in Mo and EMo treatments will guarantee the consumption of acetyl-CoA to format rubber poly-molecules. The main role of fructokinase (spots 126 and 134) is in sucrose and fructose metabolism (Odanaka et al., 2002). In our results, fructokinase was induced by Eth but reduced by Mo and EMo treatments. Downregulation of several proteins, such as triosephosphateisomerase, UDP-glucose 6-dehydrogenase, and uridylyltransferase, inhibited sucrose metabolism to maintain the soluble sugar content, that helps to maintain the cell osmotic potential under Mo treatment. Sucrose transporters are important in the substrate supply of rubber biosynthesis. They mediate the increased transport of sucrose into laticifer cells (Dusotoit-Coucaud et al., 2010; Tang et al., 2010). However, in this proteomic study, we did not identify sucrose transporters as DEPs.

A series of enzymes involved in the MVA pathway play crucial roles in NRB. These enzymes are ACAT (Sando et al., 2008), 3-hydroxy-3-methylglutaryl coenzyme A reductase (HMGR; Sando et al., 2008; Zhu and Zhang, 2009), 3-hydroxy-3-methylglutaryl coenzyme A synthase (HMGS; Sando et al., 2008), mevalonate diphosphate decarboxylase (MEVD; Wu et al., 2017), mevalonate kinase (MEVK), farnesyldiphosphate synthase (FADS; Adiwilaga and Kush, 1996), cis-prenyltransferase (CPT; Takahashi et al., 2012), REF (Cornish, 2001; Berthelot et al., 2012), and SRPP (Cornish, 2001; Berthelot et al., 2014). In this proteomic study, only three related proteins were identified. ACAT catalyzes a Claisen-type condensation of two acetyl-CoA units to form acetoacetyl-CoA, which is recognized as the first step in the MVA pathway (Kirby and Keasling, 2009). In rubber trees, under Eth treatment, the gene and protein expression of most ACAT isoforms was depressed (Wang et al., 2015). Our results showed that five protein spots were identified as two ACAT members (scaffold1479_76107 and scaffold0992_72818), and they were induced by Eth but reduced by EMo. During rubber elongation, REF and SRPP are the two key proteins (Sando et al., 2008). However, they were not obviously changed at both the gene and protein levels upon Eth treatment (Wang et al., 2015). In our results, several REF species were reduced, but only one SRPP (S152) was reduced under Mo treatment (**Supplementary Table S1**).

### Mo Can Improve Latex Yield Might Partially by Activating the Activity of Antioxidant Enzymes

Mo, as an essential microelement in plants, plays a very important role in nitrate assimilation, sulfite detoxification, purine metabolism, and the synthesis of abscisic acid, auxin, and glucosinolates (Qin et al., 2017). Our results demonstrated that the activity of the three Mo-containing enzymes (AO, XDH, and NR) was significantly increased under Eth, Mo, and EMo treatments (**Figure 7**). Therefore, we consider that Mo may play important roles in reducing the negative effects caused by the over-application of ethephon in natural rubber production.

Redox homoeostasis is controlled by the biosynthesis and reduction of antioxidants, and many ROS-scavenging enzymes in rubber latex have been studied for a long time (Putranto et al., 2015). Rubber latex contains three major antioxidants, named thiol, ascorbate, and tocotrienol. The total thiol content is positively correlated with latex production and is traditionally used to monitor the physiological status of the rubber trees (Sreelatha et al., 2009). In our results, the total thiol content was increased under treatments, especially, under EMo (**Figure 1**). Antioxidant enzymes, including SOD, CAT, APX, and GST are crucial for breaking down the harmful end-products of oxidative modification. Among them, SOD abundance was increased under Eth treatment (Wang et al., 2015). In this work, the protein abundance, gene expression, and enzyme activity of MnSOD were all induced by EMo treatment. Although GR and APX were not identified as DEPs, their enzyme activities were increased under Mo and EMo treatments (**Figure 7**). GST can catalyze the reduction of organic hydroperoxides using glutathione as a coenzyme. The abundance of GST was increased by Eth treatment (Wang et al., 2015). In this proteomic study, five protein spots were identified as four unique GST members. The GST accumulation was almost reduced after Eth, Mo, and EMo treatments (**Figure 2** and **Supplementary Table S1**), indicating the importance of GST in Eth- and Mo-induced rubber latex production.

## Author Contributions

XW and PH: conceived and designed the experiments. LG, YS, and MW: performed the experiments. DW, JW, GW, and XJ: analyzed the data. BW and WW: contributed reagents/materials/analysis tools. YS and XW: wrote and polished the paper.

## Conflict of Interest Statement

The authors declare that the research was conducted in the absence of any commercial or financial relationships that could be construed as a potential conflict of interest.

## Footnotes

1 www.ncbi.nlm.nih.gov/nuccore/448814761

## Supplementary Material

FIGURE S1 The three duplicate 2-DE gel profiles of the three treatments and CK.

FIGURE S2 Detail information for MS identification of DEPs in rubber latex.

FIGURE 3 All of the identified 169 DEPs from the latex treated with Eth, Mo, and EMo were performed venn diagram analysis **(A)**. Then, the upregulated **(B)** and downregulated **(C)** proteins were classified to show their changed patterns after different treatments.

TABLE S1 MS identification of DEPs in rubber latex after Eth, Mo, and EMo treatments.

TABLE S2 Functional analysis of the identified DEPs in rubber latex.

TABLE S3 Primers and qRT-PCR analysis of the gene expression of the corresponding DEPs in rubber latex.
