# Supplementary material for: Physiological and Proteomic Analyses of Molybdenum- and Ethylene-Responsive Mechanisms in Rubber Latex
Source: Front Plant Sci. 2018 May 15;9:621. doi: 10.3389/fpls.2018.00621 (PMC5962772; doi:10.3389/fpls.2018.00621)
Supplement: FIGURE S1 — The three duplicate 2-DE gel profiles of the three treatments and CK. [file Presentation_1.PDF]

Supplemental Figure S1:

The three duplicate 2-DE gel profiles of the three treatments and CK

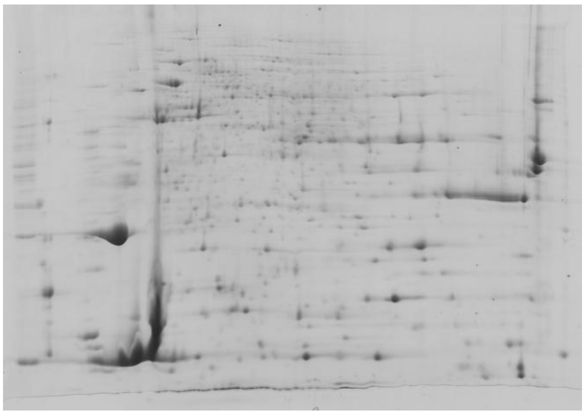

CK1

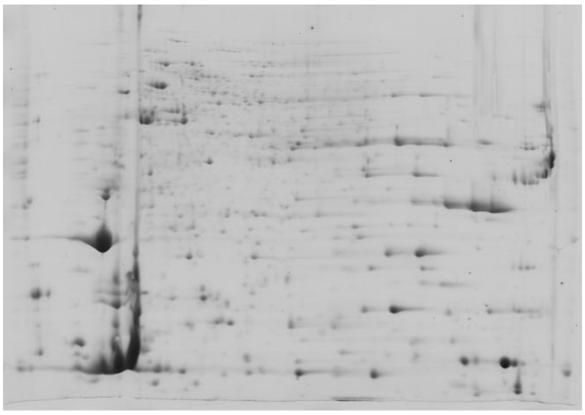

CK2

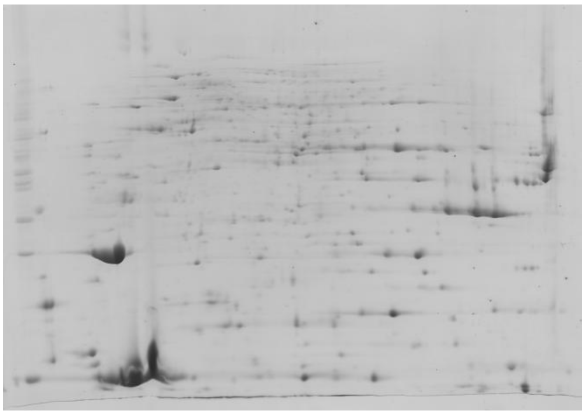

CK3

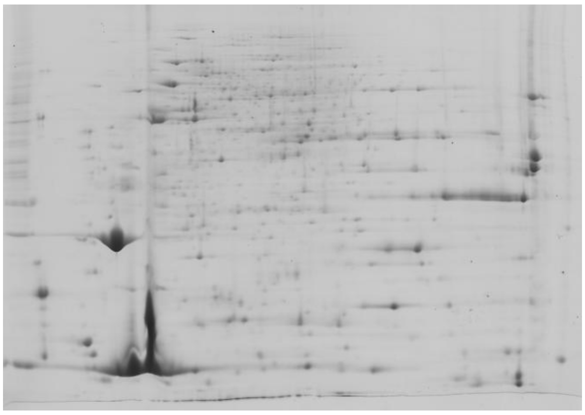

Eth1

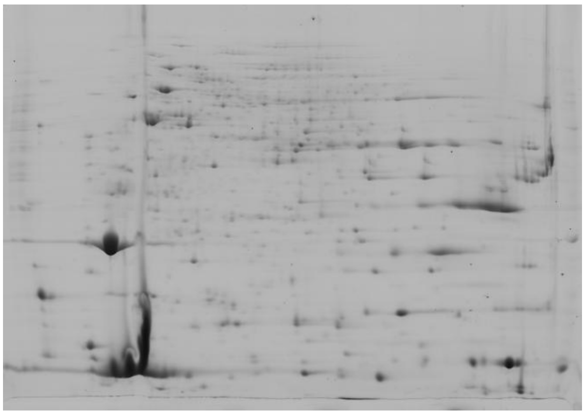

Eth2

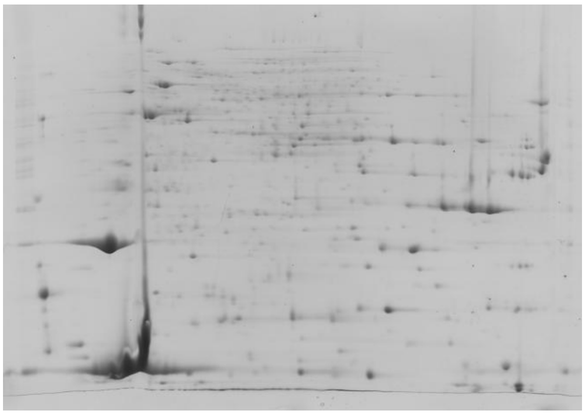

Eth3

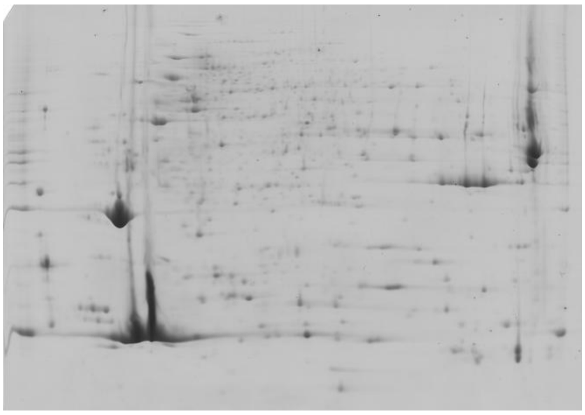

Mo1

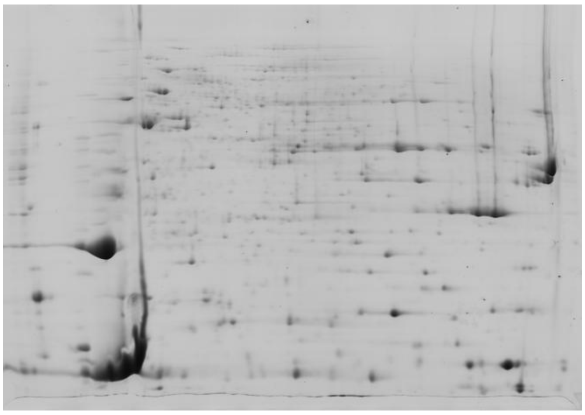

Mo2

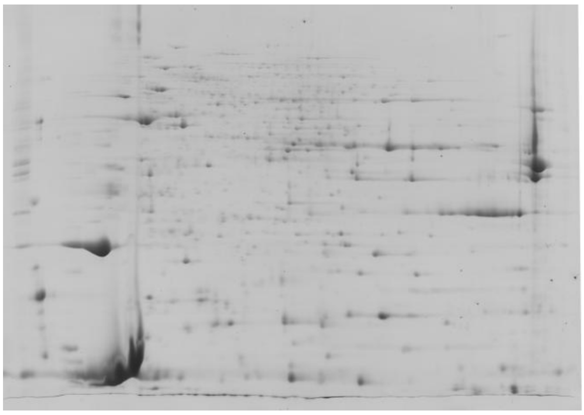

Mo3

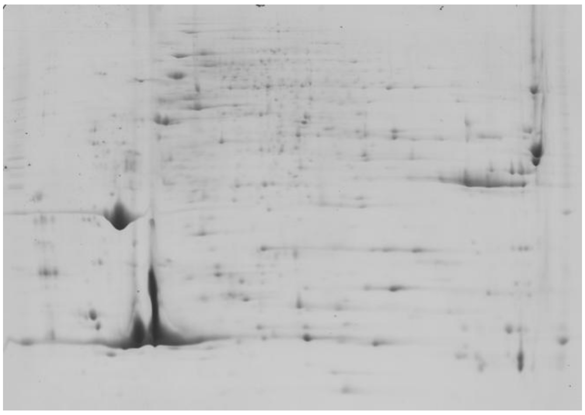

EMo1

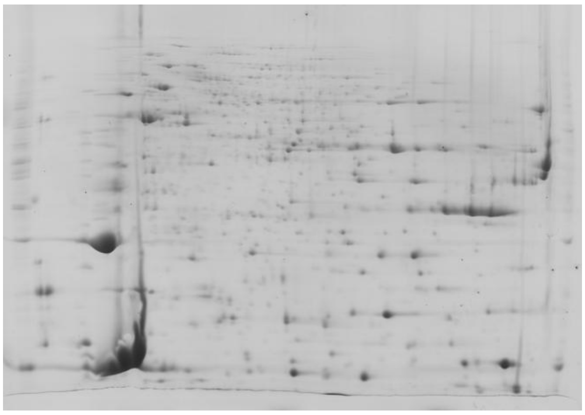

EMo2

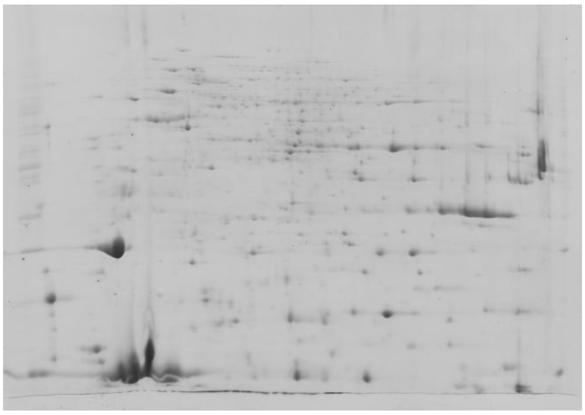

EMo3
