# Supplementary material for: Physiological and Proteomic Analyses of Molybdenum- and Ethylene-Responsive Mechanisms in Rubber Latex
Source: Front Plant Sci. 2018 May 15;9:621. doi: 10.3389/fpls.2018.00621 (PMC5962772; doi:10.3389/fpls.2018.00621)

Supplementary Figure S2

Detail information for MS identification of DEPs in rubber latex

Database searched results of the identified proteins in the 2-DE gels. Spot numbers of the **169** proteins correspond to the proteins that listed in Figure 2.

Accession No.: The accession number of identified proteins in the rubber tree genome database.

Peptide sequences: The amino acid sequence of the matched peptides from the identified protein.

PFF Mascot score: The score of protein database search after MALDI TOF/TOF MS.

Sequence coverage %: The percentage of the matched peptides in the identified protein.

Matched peptides No.: The number of matched peptides from identified protein.

Calculated Mr: The theoretical molecular weight of identified protein.

Calculated pI: The theoretical isoelectric point of identified protein.

Spot No.:**1**

Accession No.: **scaffold0698_149194.mRNA1**

Protein name： **uncharacterized protein**

Peptide sequences: K.EFEELKPEVESVR.E; R.SCCGLFEVLR.R

PFF Mascot score: **[98]**   Sequence coverage %: **[8]**

Matched peptides No.: **[2]**

Calculated Mr: **29873**   Calculated *p*I: **4.17**

Data base searched result:

Ions score is -10*Log(P), where P is the probability that the observed match is a random event.
Individual ions scores > 30 indicate identity or extensive homology (p<0.05).
Protein scores are derived from ions scores as a non-probabilistic basis for ranking protein hits.

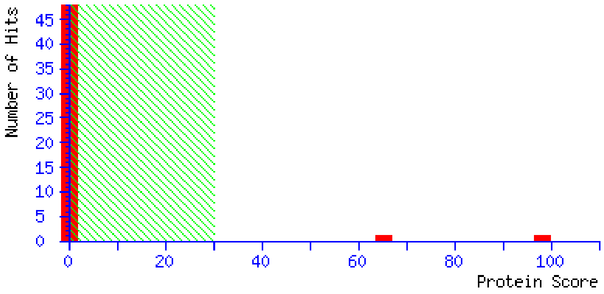

Matched peptide sequences: shown in Bold Red

```
  1 MEEFSNGQIE VAQKVETDEE DNRGVAKEFE ELKPEVESVR EIVVDGNSES
 51 FKETHDVLSS DSSNSPEEEA EAKDQSLSAK DSGTLEEEVK KDVTDIIIES
101 AESIVPLPQV LSSTTETSIE KSQEPDVTEV EQKEIEVKIF PSSNETNEKT
151 TLDETDGGVS EVAPDGSKET RLPSSDDKRD LSAIATDTVS DGVEEAIVIA
201 LEENTGEPSA FVDKESEENT DDSLKPSNNA TIVGSSDGFP ESKGNPHVTS
251 LSQRTLQRTS WRSCCGLFEV LRR
```

Matched peptide information:

Start - End	Observed	Mr(expt)	Mr(calc)	ppm	Miss	Sequence	
28 - 40	1590.7535	1589.7463	1589.7937	-30	1	K.EFEELKPEVESVR.E	(Ions score 93)
263 - 272	1240.5476	1239.5403	1239.5740	-27	0	R.SCCGLFEVLR.R	(Ions score 37)

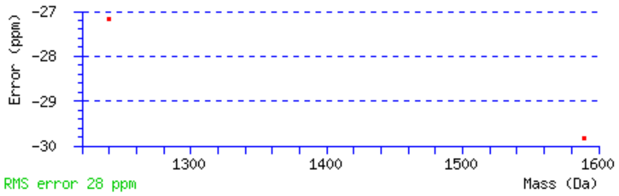

Spot No.: **2**

Accession No.: **scaffold1173_171897.mRNA1**

Protein name： **Aldose 1-epimerase**

Peptide sequences: **K.ITNLGCIITSLSVPDR.N;**

K.GAAPYFGAIVGR.V;K.FTLNGVEYTLPINKPPNSLHGGHK.G;K.YQSHDGEEGYPGDLSVTATYTLTSSTTMR.L

PFF Mascot score: **[127]** Sequence coverage %: **[23]**

Matched peptides No.: **[4]**

Calculated Mr: **36961** Calculated *p*I: **5.70**

Data base searched result:

Ions score is -10*Log(P), where P is the probability that the observed match is a random event.
Individual ions scores > 30 indicate identity or extensive homology (p<0.05).
Protein scores are derived from ions scores as a non-probabilistic basis for ranking protein hits.

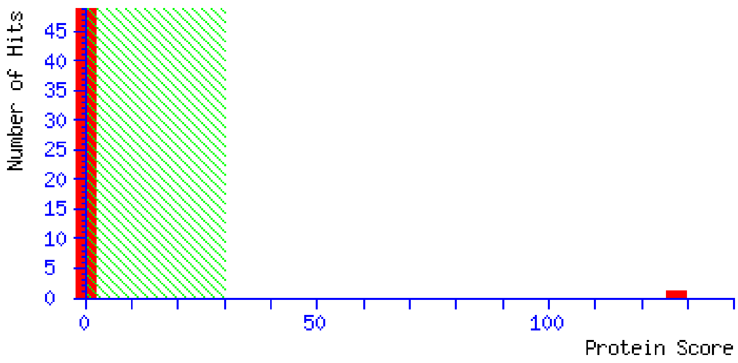

Matched peptide sequences: shown in Bold Red

```
  1 MADQTQNPQI FELNNGSMLV KITNLGCIIT SLSVPDRNGN LADVVLGFDS
 51 VEPYLKGAAP YFGAIVGRVA NRIKNGKFTL NGVEYTLPIN KPPNSLHGGH
101 KGFDKVIWEV AEYKKGENSS ITFKYQSHDG EEGYPGDLSV TATYTLTSST
151 TMRLDMEAVP KDKATPVSLA QHTYWNLAGH NSGNILDHSI QIWGSHVTPV
201 DENTVPTGEI MPVKGTPFDF SSEKRVGASI LEVGLGYDHN YVVDCGEEQS
251 GLKRAAKLKD PSSSRVLNLW TNAPGMQFYT GNYVDGVVGK GGAVYGKHSG
301 LCLETQGFPN AINQSNFPSV VVQPGETYKH TMLFEFSVE
```

Matched peptide information:

Start - End	Observed	Mr(expt)	Mr(calc)	ppm	Miss	Sequence
22 - 37	1758.9076	1757.9003	1757.9346	-19	0	K.ITNLGCIITSLSVPDR.N (Ions score 72)
57 - 68	1178.6006	1177.5933	1177.6244	-26	0	K.GAAPYFGAIVGR.V (Ions score 64)
78 - 101	2633.3069	2632.2996	2632.3762	-29	1	K.FTLNGVEYTLPINKPPNSLHGGHK.G (Ions score 27)
125 - 153	3167.3708	3166.3636	3166.4037	-13	0	K.YQSHDGEEGYPGDLSVTATYTLTSSTTMR.L (Ions score 45)

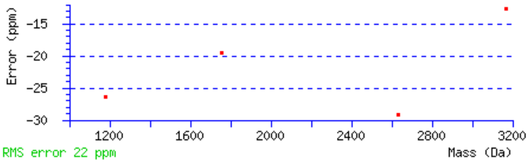

Spot No.: 3

Accession No.: **scaffold0901_132647.mRNA1**

Protein name： **ADP-ribosylation factor GTPase-activating protein AGD13**

Peptide sequences: **R.DHGGSDPYVVVTSGHQK.L; K.NNSNPEWNDELTLSITDLNVPIK.L;K.LEVFDKDTFTEDDK.M; K.MGDAEIDIKPYIEILK.M; K.VDNCLADESHIIWNNGEITQDMHLR.L**

PFF Mascot score: **[467]** Sequence coverage %: **[55]**

Matched peptides No.: **[5]**

Calculated Mr: **19416** Calculated *p*I: **5.14**

Data base searched result:

Ions score is -10*Log(P), where P is the probability that the observed match is a random event.
Individual ions scores > 31 indicate identity or extensive homology (p<0.05).
Protein scores are derived from ions scores as a non-probabilistic basis for ranking protein hits.

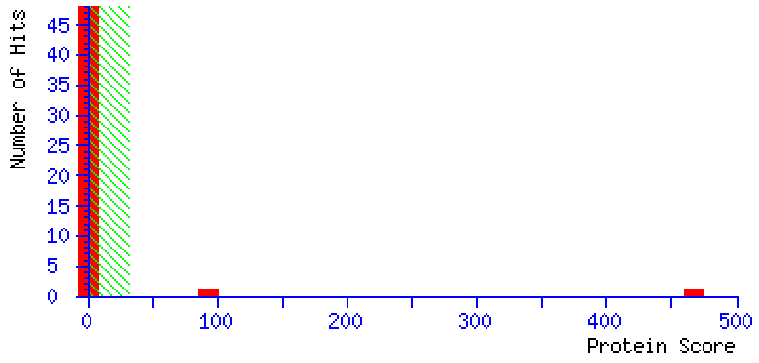

Matched peptide sequences: shown in Bold Red

```
  1 MENILGLLRI RVRRGVNLAV RDHGGSDPYV VVTSGHQKLK TRTEKNNSNP
 51 EWNDELTLSI TDLNVPIKLE VFDKDTFTED DKMGDAEIDI KPYIEILKMG
101 LQNLPNGCVV KKVPPKVDNC LADESHIIWN NGEITQDMHL RLRNVECGEV
151 EVQIKWINVP GSKGLEIEGS S
```

Matched peptide information:

Start - End	Observed	Mr(expt)	Mr(calc)	ppm	Miss	Sequence
22 - 38	1782.7490	1781.7417	1781.8333	-51	0	R.DHGGSDPYVVVTSGHQK.L (Ions score 123)
46 - 68	2626.1785	2625.1712	2625.2922	-46	0	K.NNSNPEWNDELTLSITDLNVPIK.L (Ions score 153)
69 - 82	1701.7014	1700.6941	1700.7781	-49	1	K.LEVFDKDTFTEDDK.M (Ions score 106)
83 - 98	1847.8888	1846.8815	1846.9750	-51	1	K.MGDAEIDIKPYIEILK.M (Ions score 77)
117 - 141	2980.2241	2979.2168	2979.3603	-48	0	K.VDNCLADESHIIWNNGEITQDMHLR.L (Ions score 120)

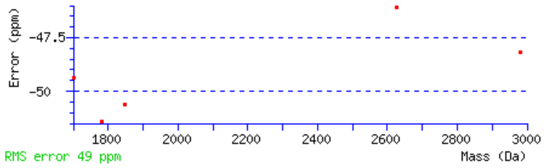

Spot No.: **4**

Accession No.: **scaffold0155_515853.mRNA1**

Protein name：**Pro-hevein**

Peptide sequences: **K.YGWTAFCGPVGAHGQPSCGK.C; R.IVDQCSNGGLDLDVNVFR.Q**

PFF Mascot score: **[211]** Sequence coverage %: **[18]**

Matched peptides No.: **[2]**

Calculated Mr: **23042** Calculated *p*I: **8.15**

Data base searched result:

Ions score is -10*Log(P), where P is the probability that the observed match is a random event.
Individual ions scores > 30 indicate identity or extensive homology (p<0.05).
Protein scores are derived from ions scores as a non-probabilistic basis for ranking protein hits.

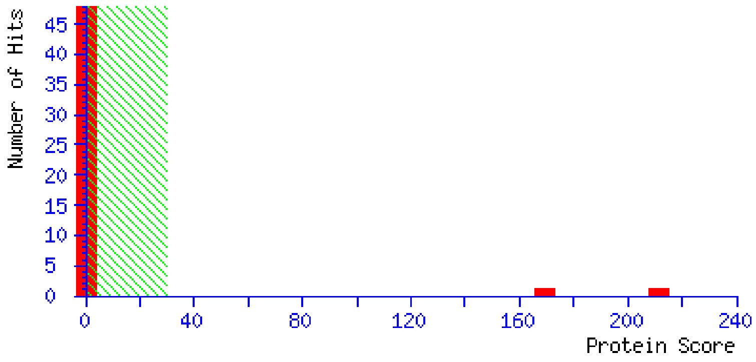

Matched peptide sequences: shown in **Bold Red**

```
  1 MGRVMNIFIV VLLCLTGVAI AEQCGRQAGG KLCPNNLCCS QYGWCGSSDD
 51 YCSPSKNCQS NCKGGGGGGG GGGGSASNVL ATYHLYNPQQ HGWDLNAVSA
101 YCSTWDANKP YSWRSKYGWT AFCGPVGAHG QPSCGKCLSV TNTGTGAKTT
151 VRIVDQCSNG GLDLDVNVFR QLDTDGKGYE RGHLTVNYQF VNCGDSFNPL
201 FSIMKSSVIN
```

Matched peptide information:

Start - End	Observed	Mr(expt)	Mr(calc)	ppm	Miss	Sequence	
117 - 136	2136.8301	2135.8228	2135.9306	-50	0	K.YGWTAFCGPVGAHGQPSCGK.C	(Ions score 83)
153 - 170	2020.8917	2019.8844	2019.9684	-42	0	R.IVDQCSNGGLDLDVNVFR.Q	(Ions score 158)

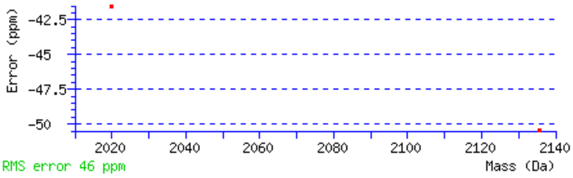

Spot No.: 5

Accession No.: **scaffold4512_855.mRNA1**

Protein name：**Osmotin-like protein OSM34**

Peptide sequences: **R.NNCPYTVWAAASPGGGR.R;R.RLDQGQTWELNVPAGTSMAR.I; R.LDQGQTWELNVPAGTSMAR.I; R.TNCNFDGSGK.G; K.CRPLFCTADINGQCPNQLK.A; K.APGGCNNPCTVFK.T; K.TNEYCCTEGYGTCGPTEFSK.F; R.CSDAYSYPQDDPSSTFTCPGGTNYR.V**

PFF Mascot score: **[607]** Sequence coverage %: **[50]**

Matched peptides No.: **[8]**

Calculated Mr: **27682** Calculated *p*I: **5.23**

Data base searched result:

Ions score is -10*Log(P), where P is the probability that the observed match is a random event.
Individual ions scores > 31 indicate identity or extensive homology (p<0.05).
Protein scores are derived from ions scores as a non-probabilistic basis for ranking protein hits.

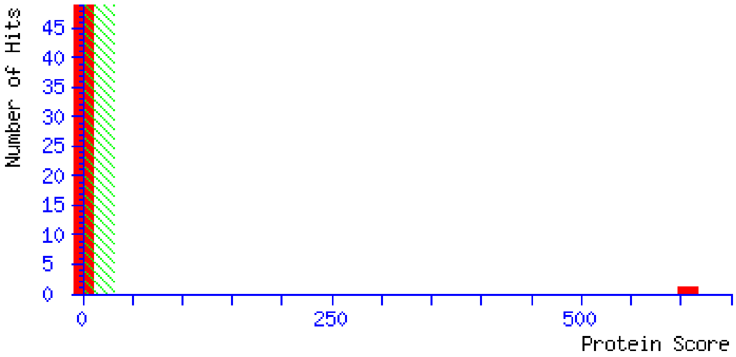

Matched peptide sequences: shown in Bold Red

```
  1 MSNFNIFLIS IFLLSALFFT SSDGATFTIR NNCPYTVWAA ASPGGGRRLD
 51 QGQTWELNVP AGTSMARIWG RTNCNFDGSG KGHCQTGDCG GILACQGWGV
101 PPNTLAEYAL NQFGNLDFYD ISLVDGFNIP IEFSPTSGAK DKCRPLFCTA
151 DINGQCPNQL KAPGGCNNPC TVFKTNEYCC TEGYGTCGPT EFSKFFKSRC
201 SDAYSYPQDD PSSTFTCPGG TNYRVVFCPA RSPHFPLEMV REKDVE
```

Matched peptide information:

Start - End	Observed	Mr(expt)	Mr(calc)	ppm	Miss	Sequence
31 - 47	1777.7050	1776.6977	1776.8002	-58	0	R.NNCPYTVWAAASPGGGR.R (Ions score 141)
48 - 67	2229.9822	2228.9749	2229.0960	-54	1	R.RLDQGQTWELNVPAGTSMAR.I (Ions score 47)
49 - 67	2073.8831	2072.8758	2072.9949	-57	0	R.LDQGQTWELNVPAGTSMAR.I (Ions score 175)
72 - 81	1099.3757	1098.3685	1098.4400	-65	0	R.TNCNFDGSGK.G (Ions score 31)
143 - 161	2291.9348	2290.9275	2291.0609	-58	1	K.CRPLFCTADINGQCPNQLK.A (Ions score 47)
162 - 174	1421.5402	1420.5329	1420.6228	-63	0	K.APGGCNNPCTVFK.T (Ions score 74)
175 - 194	2360.7610	2359.7537	2359.9032	-63	0	K.TNEYCCTEGYGTCGPTEFSK.F (Ions score 116)
200 - 224	2845.9517	2844.9444	2845.1232	-63	0	R.CSDAYSYPQDDPSSTFTCPGGTNYR.V (Ions score 154)

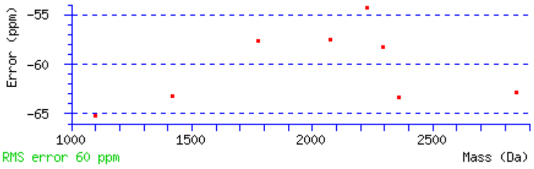

RMS error 60 ppm

Spot No.:6

Accession No.: scaffold0026_1110574.mRNA1

Protein name： Actin-7

Peptide sequences: K.AGFAGDDAPR.A; R.AVFPSIVGR.P; K.IWHHTFYNELR.V; R.VAPEEHPVLLTEAPLNPK.A; R.TTGIVLDSGDGVSHTVPIYEGYALPHAILR.L; R.GYMFTTTAER.E; K.LAYVALDYEQELETAK.S; K.NYELPDGQVITIGAER.F; K.DLYGNIVLSGGSTMFPGIADR.M; K.GEYDESGPSIVHR.K

PFF Mascot score: [644]   Sequence coverage %: [40]

Matched peptides No.: [10]

Calculated Mr: 41897   Calculated *p*I: 5.31

Data base searched result:

Ions score is -10*Log(P), where P is the probability that the observed match is a random event.
Individual ions scores > 31 indicate identity or extensive homology (p<0.05).
Protein scores are derived from ions scores as a non-probabilistic basis for ranking protein hits.

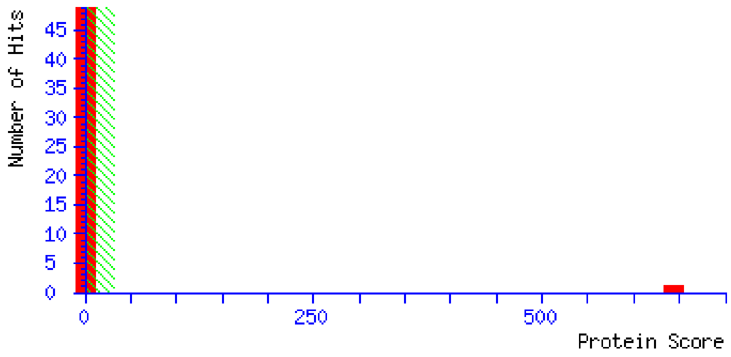

Matched peptide sequences: shown in Bold Red

```
  1 MADAEDIQPL VCDNGTGMVK AGFAGDDAPR AVFPSIVGRP RHTGVMVGMG
 51 QKDAYVGDEA QSKRGILTLK YPIEHGIVSN WDDMEKIWHH TFYNELRVAP
101 EEHPVLLTEA PLNPKANREK MTQIMFETFN VPAMYVAIQA VLSLYASGRT
151 TGIVLDSGDG VSHTVPIYEG YALPHAILRL DLAGRDLTDA LMKILTERGY
201 MFTTTAEREI VRDMKEKLAY VALDYEQELE TAKSSSSVEK NYELPDGQVI
251 TIGAERFRCP EVLFQPSLIG MEAAGIHETT YNSIMKCDVD IRKDLYGNIV
301 LSGGSTMFPG IADRMSKEIT ALAPSSMKIK VVAPPERKYS VWIGGSILAS
351 LSTFQQMWIS KGEYDESGPS IVHRKCF
```

Matched peptide information:

Start - End	Observed	Mr(expt)	Mr(calc)	ppm	Miss	Sequence
21 - 30	976.3781	975.3708	975.4410	-72	0	K.AGFAGDDAPR.A (Ions score 44)
31 - 39	945.4824	944.4751	944.5444	-73	0	R.AVFPSIVGR.P (Ions score 48)
87 - 97	1515.6470	1514.6397	1514.7419	-67	0	K.IWHHTFYNELR.V (Ions score 77)
98 - 115	1953.9282	1952.9209	1953.0571	-70	0	R.VAPEEHPVLLTEAPLNPK.A (Ions score 103)
150 - 179	3151.4067	3150.3995	3150.6350	-75	0	R.TTGIVLDSGDGVSHTVPIYEGYALPHAILR.L (Ions score 47)
199 - 208	1176.4526	1175.4454	1175.5281	-70	0	R.GYMFTTTAER.E (Ions score 65)
218 - 233	1855.8101	1854.8028	1854.9251	-66	0	K.LAYVALDYEQELETAK.S (Ions score 82)
241 - 256	1774.7760	1773.7687	1773.8897	-68	0	K.NYELPDGQVITIGAER.F (Ions score 137)
294 - 314	2182.9358	2181.9285	2182.0729	-66	0	K.DLYGNIVLSGGSTMFPGIADR.M (Ions score 170)
362 - 374	1445.5649	1444.5577	1444.6583	-70	0	K.GEYDESGPSIVHR.K (Ions score 115)

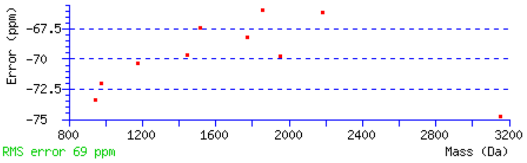

Spot No.:7

Accession No.: **scaffold0026_1110574.mRNA1**

Protein name： **Actin-7**

Peptide sequences:

K.AGFAGDDAPR.A;R.AVFPSIVGR.P;K.IWHHTFYNELR.V;R.VAPEEHPVLLTEAPLNPK.A;R.TTGIVLDSGDGVSHTVPIYEGYALPHAILR.L;R.GYMFTTTAER.E;K.LAYVALDYEQELETAK.S;K.NYELPDGQVITIGAER.F;K.DLYGNIVLSGGSTMFPGIADR.M;K.GEYDESGPSIVHR.K

PFF Mascot score: **[644]** Sequence coverage %: **[40]**

Matched peptides No.: **[10]**

Calculated Mr: **41897** Calculated *p*I: **5.31**

Data base searched result:

Ions score is -10*Log(P), where P is the probability that the observed match is a random event.
Individual ions scores > 31 indicate identity or extensive homology (p<0.05).
Protein scores are derived from ions scores as a non-probabilistic basis for ranking protein hits.

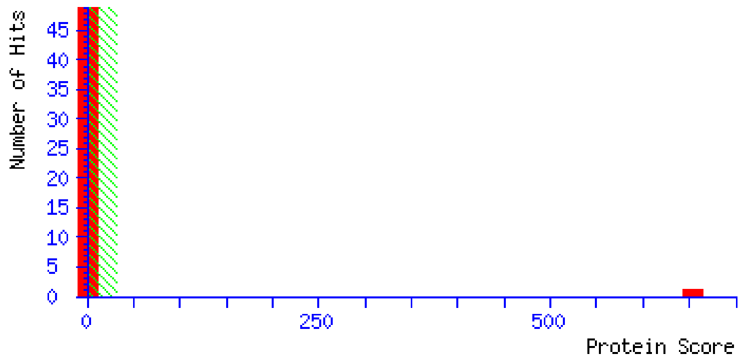

Matched peptide sequences: shown in Bold Red

```
  1 MADAEDIQPL VCDNGTGMVK AGFAGDDAPR AVFPSIVGRP RHTGVMVGMG
 51 QKDAYVGDEA QSKRGILTLK YPIEHGIVSN WDDMEKIWHH TFYNELRVAP
101 EEHPVLLTEA PLNPKANREK MTQIMFETFN VPAMYVAIQA VLSLYASGRT
151 TGIVLDSGDG VSHTVPIYEG YALPHAILRL DLAGRDLTDA LMKILTERGY
201 MFTTTAEREI VRDMKEKLAY VALDYEQELE TAKSSSSVEK NYELPDGQVI
251 TIGAERFRCP EVLFQPSLIG MEAAGIHETT YNSIMKCDVD IRKDLYGNIV
301 LSGGSTMFPG IADRMSKEIT ALAPSSMKIK VVAPPERKYS VWIGGSILAS
351 LSTFQQMWIS KGEYDESGPS IVHRKCF
```

Matched peptide information:

Start - End	Observed	Mr(expt)	Mr(calc)	ppm	Miss	Sequence
21 - 30	976.3926	975.3854	975.4410	-57	0	K.AGFAGDDAPR.A (Ions score 37)
31 - 39	945.4968	944.4896	944.5444	-58	0	R.AVFPSIVGR.P (Ions score 48)
87 - 97	1515.6680	1514.6607	1514.7419	-54	0	K.IWHHTFYNELR.V (Ions score 73)
98 - 115	1953.9354	1952.9281	1953.0571	-66	0	R.VAPEEHPVLLTEAPLNPK.A (Ions score 104)
150 - 179	3151.4197	3150.4124	3150.6350	-71	0	R.TTGIVLDSGDGVSHTVPIYEGYALPHAILR.L (Ions score 101)
199 - 208	1176.4705	1175.4632	1175.5281	-55	0	R.GYMFTTTAER.E (Ions score 56)
218 - 233	1855.8138	1854.8066	1854.9251	-64	0	K.LAYVALDYEQELETAK.S (Ions score 81)
241 - 256	1774.7943	1773.7870	1773.8897	-58	0	K.NYELPDGQVITIGAER.F (Ions score 145)
294 - 314	2182.9519	2181.9446	2182.0729	-59	0	K.DLYGNIVLSGGSTMFPGIADR.M (Ions score 155)
362 - 374	1445.5846	1444.5773	1444.6583	-56	0	K.GEYDESGPSIVHR.K (Ions score 109)

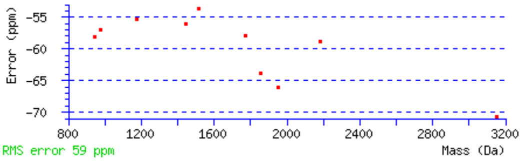

Spot No.:**8**

Accession No.: **scaffold0944_314376.mRNA1**

Protein name：**Glutathione S-transferase PARB**

Peptide sequences:

K.ELEFVFVNINMGIGEHK.K;R.AITQYIAHENQEK.G;K.VLDVYESR.L;K.YLAGDSFTLADMHHLPNLHLLFVTQSK.K;R.PHVSAWAADITAR.P

PFF Mascot score: **[317]** Sequence coverage %: **[36]**

Matched peptides No.: **[5]**

Calculated Mr: **23875** Calculated *p*I: **6.31**

Data base searched result:

Ions score is -10*Log(P), where P is the probability that the observed match is a random event.
Individual ions scores > 31 indicate identity or extensive homology ($p<0.05$).
Protein scores are derived from ions scores as a non-probabilistic basis for ranking protein hits.

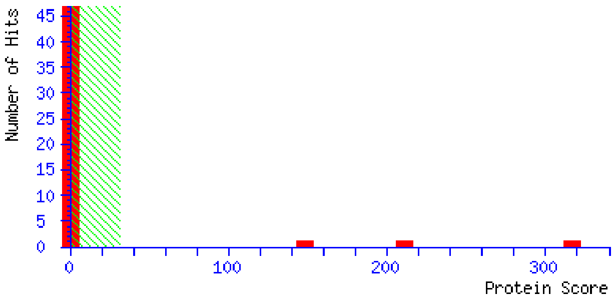

Matched peptide sequences: shown in Bold Red

```
  1 MAAIKVHGSP LSTATQRVLV SLHEKELEFV FVNINMGIGE HKKEPFISLN
 51 PFGQVPALEH GDLKLFESRA ITQYIAHENQ EKGTQLLCQG KQMAIVSVWM
101 EVEAQQFEPA ASKLNWEIFF KPLFGLTTDA AAVEENEAKL AKVLDVYESR
151 LAQSKYLAGD SFTLADMHHL PNLHLLFVTQ SKKLIESRPH VSAWAADITA
201 RPAWAKVLAM QKN
```

Matched peptide information:

Start - End	Observed	Mr(expt)	Mr(calc)	ppm	Miss	Sequence
26 - 42	1975.8385	1974.8312	1974.9873	-79	0	K.ELEFVFVNINMGIGEHK.K (Ions score 131)
70 - 82	1544.6414	1543.6341	1543.7630	-84	0	R.AITQYIAHENQEK.G (Ions score 84)
143 - 150	980.4189	979.4116	979.4975	-88	0	K.VLDVYESR.L (Ions score 49)
156 - 182	3068.3164	3067.3091	3067.5590	-81	0	K.YLAGDSFTLADMHHLPNLHLLFVTQSK.K (Ions score 32)
189 - 201	1394.6106	1393.6033	1393.7102	-77	0	R.PHVSAWAADITAR.P (Ions score 128)

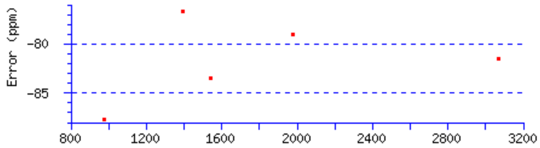

RMS error 81 ppm

Spot No.:**9**

Accession No.: **scaffold0217_269209.mRNA1**

Protein name： **Uncharacterized protein**

Peptide sequences:

K.SLDLLQEIR.L;R.LPMGLLPLDDIVEVGYNR.T;R.NVSYDKEVTAFVEDR.R;K.ITFGNPTGISR.T

PFF Mascot score: **[181]** Sequence coverage %: **[30]**

Matched peptides No.: **[4]**

Calculated Mr: **20418** Calculated *p*I: **9.49**

Data base searched result:

Ions score is -10*Log(P), where P is the probability that the observed match is a random event.
Individual ions scores > 31 indicate identity or extensive homology (p<0.05).
Protein scores are derived from ions scores as a non-probabilistic basis for ranking protein hits.

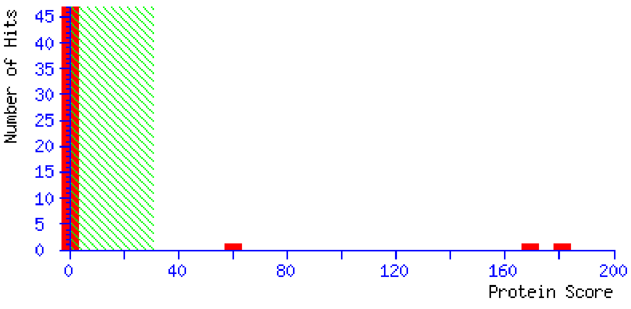

Matched peptide sequences: shown in Bold Red

```
  1 MLSLPPNAQP LFSFAKKNNP YFRVKTPFTQ MTSQPLTNHR EGAEIYHGES
 51 LCKQKSLDLL QEIRLPMGLL PLDDIVEVGY NRTTGFVWLK QKKRKEHKFR
101 AIGRNVSYDK EVTAFVEDRR MRRLTGVKSK EFLIWVTISD IYVDSGDTKK
151 ITFGNPTGIS RTFPVSAFEL EEEEKK
```

Matched peptide information:

Start - End	Observed	Mr(expt)	Mr(calc)	ppm	Miss	Sequence
56 - 64	1086.5454	1085.5381	1085.6080	-64	0	K.SLDLLQEIR.L (Ions score 57)
65 - 82	2013.9347	2012.9274	2013.0605	-66	0	R.LPMGLLPLDDIVEVGYNR.T (Ions score 98)
105 - 119	1771.7423	1770.7350	1770.8424	-61	1	R.NVSYDKEVTAFVEDR.R (Ions score 65)
151 - 161	1162.5509	1161.5436	1161.6142	-61	0	K.ITFGNPTGISR.T (Ions score 51)

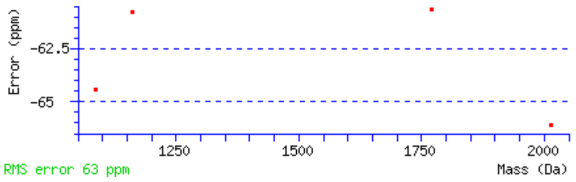

Spot No.:**10**

Accession No.: **scaffold1093_241290.mRNA1**

Protein name：**Pro-hevein**

Peptide sequences:

K.GGGGGGGGGGGGGSASNVR.A;K.YGWTAFCGPVGPHGQASCGK.C;R.IVDQCSNGGLDLDVNVFR.K

PFF Mascot score: **[263]** Sequence coverage %: **[26]**

Matched peptides No.: **[3]**

Calculated Mr: **23451** Calculated *p*I: **8.12**

Data base searched result:

Ions score is -10*Log(P), where P is the probability that the observed match is a random event.
Individual ions scores > 31 indicate identity or extensive homology (p<0.05).
Protein scores are derived from ions scores as a non-probabilistic basis for ranking protein hits.

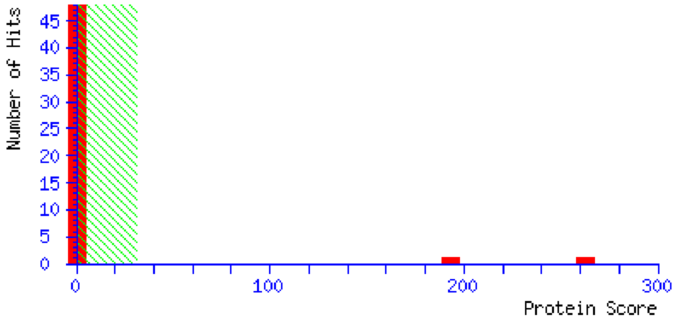

Matched peptide sequences: shown in Bold Red

```
  1 MGRVMNICVV VLLCLIGGAI AEQCGRQAGG KLCPNNLCCS QYGWCGSSDD
 51 YCSPSKNCQS NCKGGGGGGG GGGGGGSASN VRATYHLYNP QQHGWDLNAV
101 SAYCSTWDAN KPYSWRSKYG WTAFCGPVGP HGQASCGKCL RVTNTRTGAK
151 TTVRIVDQCS NGGLDLDVNV FRKLDTDGKG YEQGHLTVNY EFVNCGDSFN
201 PLFSIMDHQY IN
```

Matched peptide information:

Start - End	Observed	Mr(expt)	Mr(calc)	ppm	Miss	Sequence	
64 - 82	1374.5085	1373.5013	1373.6033	-74	0	K.GGGGGGGGGGGGGSASNVR.A	(Ions score 60)
119 - 138	2136.7827	2135.7754	2135.9306	-73	0	K.YGWTAFCGPVGPHGQASCGK.C	(Ions score 117)
155 - 172	2020.8341	2019.8268	2019.9684	-70	0	R.IVDQCSNGGLDLDVNVFR.K	(Ions score 148)

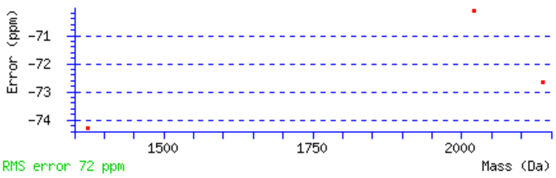

Spot No.:**11**

Accession No.: **scaffold1093_241290.mRNA1**

Protein name：**Pro-hevein**

Peptide sequences:

K.GGGGGGGGGGGGSASNVR.A;K.YGWTAFCGPVGPHGQASCGK.C;R.IVDQCSNGGLDLDVNVFR.K

PFF Mascot score: **[262]** Sequence coverage %: **[26]**

Matched peptides No.: **[3]**

Calculated Mr: **23451** Calculated *p*I: **8.12**

Data base searched result:

Ions score is -10*Log(P), where P is the probability that the observed match is a random event.
Individual ions scores > 31 indicate identity or extensive homology (p<0.05).
Protein scores are derived from ions scores as a non-probabilistic basis for ranking protein hits.

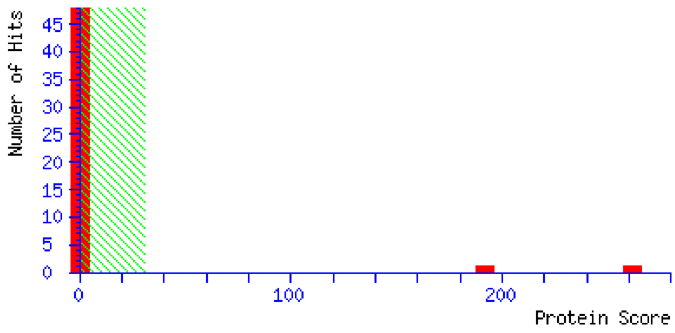

Matched peptide sequences: shown in **Bold Red**

```
  1 MGRVMNICVV VLLCLIGGAI AEQCGRQAGG KLCPNNLCCS QYGWCGSSDD
 51 YCSPSKNCQS NCKGGGGGGG GGGGGGSASN VRATYHLYNP QQHGWDLNAV
101 SAYCSTWDAN KPYSWRSKYG WTAFCGPVGP HGQASCGKCL RVTNTRTGAK
151 TTVRIVDQCS NGGLDLDVNV FRKLDTDGKG YEQGHLTVNY EFVNCGDSFN
201 PLFSIMDHQY IN
```

Matched peptide information:

Start - End	Observed	Mr(expt)	Mr(calc)	ppm	Miss	Sequence	
64 - 82	1374.5205	1373.5132	1373.6033	-66	0	K.GGGGGGGGGGGGSASNVR.A	(Ions score 59)
119 - 138	2136.7830	2135.7757	2135.9306	-73	0	K.YGWTAFCGPVGPHGQASCGK.C	(Ions score 96)
155 - 172	2020.8378	2019.8305	2019.9684	-68	0	R.IVDQCSNGGLDLDVNVFR.K	(Ions score 168)

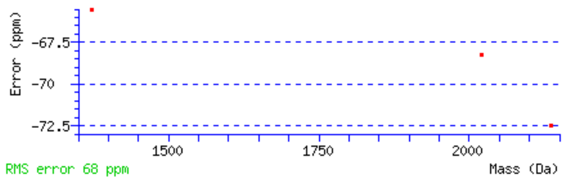

Spot No.:**12**

Accession No.: **scaffold0029_867537.mRNA1**

Protein name： **Universal stress protein A-like protein**

Peptide sequences: R.GLHLLEYFVNR.C;R.VLPDFLVVGCR.G;R.VFVGTVSEFCQK.H

PFF Mascot score: **[202]** Sequence coverage %: **[19]**

Matched peptides No.: **[3]**

Calculated Mr: **20167** Calculated *p*I: **5.87**

Data base searched result:

Ions score is -10*Log(P), where P is the probability that the observed match is a random event.
Individual ions scores > 31 indicate identity or extensive homology (p<0.05).
Protein scores are derived from ions scores as a non-probabilistic basis for ranking protein hits.

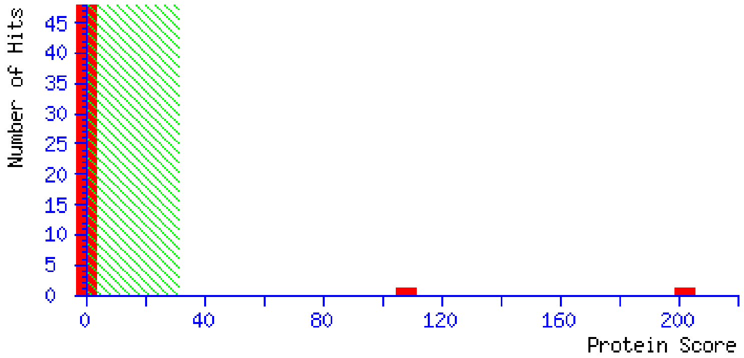

Matched peptide sequences: shown in Bold Red

```
  1 MESEPTRIMI AVNESTIKGY PHPSISSKGA FDWTLQKIVR SNTAGFKLLF
 51 LHVQVPDEDG FDDMDSIYAS PEDFKSMKQR DRVRGLHLLE YFVNRCHQIG
101 VACEAWIKSG DPKEVICHEV KRVLPDFLVV GCRGLGPFQR VFVGTVSEFC
151 QKHAECPVIS IKRRAEETPQ DPVDD
```

Matched peptide information:

Start - End	Observed	Mr(expt)	Mr(calc)	ppm	Miss	Sequence	
85 - 95	1360.6550	1359.6478	1359.7299	-60	0	R.GLHLLEYFVNR.C	(Ions score 82)
123 - 133	1274.6146	1273.6073	1273.6853	-61	0	R.VLPDFLVVGCR.G	(Ions score 99)
141 - 152	1400.5972	1399.5899	1399.6806	-65	0	R.VFVGTVSEFCQK.H	(Ions score 85)

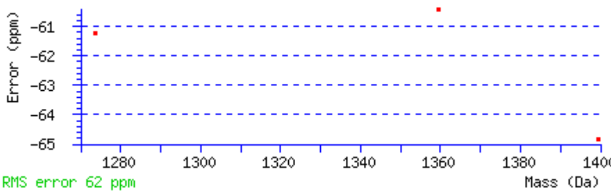

Spot No.:**13**

Accession No.: **scaffold0100_712967.mRNA1**

Protein name：**Patatin-like protein 2**

Peptide sequences:

K.ITVLSIDGGGIR.G;K.LQDLDGPDAR.I;K.SLDCEDYYLR.I;R.IQDDTLTGEESSGHIATEENLQR.L

PFF Mascot score: **[306]** Sequence coverage %: **[12]**

Matched peptides No.: **[4]**

Calculated Mr: **49142** Calculated *p*I: **5.13**

Data base searched result:

Ions score is -10*Log(P), where P is the probability that the observed match is a random event.
Individual ions scores > 31 indicate identity or extensive homology (p<0.05).
Protein scores are derived from ions scores as a non-probabilistic basis for ranking protein hits.

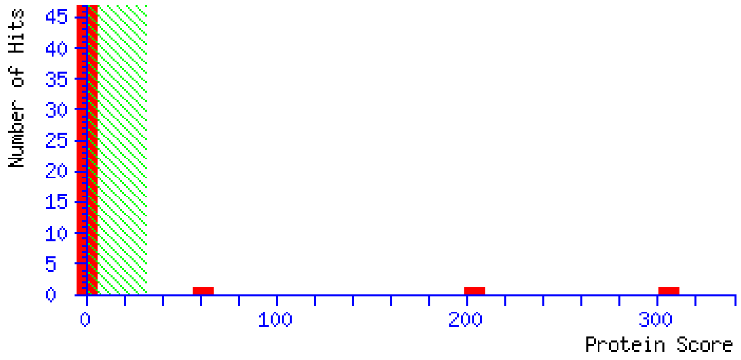

Matched peptide sequences: shown in Bold Red

1 MCLKSMLDIH DVTVHASTIL YIPKLHAQLH SHSQLVLINL LYEDRELSSL
51 DSNMATGSTT LTQGKK**ITVL SIDGGGIR**GI IPGIILASLE SK**LQDLDGPD**
101 **AR**IADYFDII AGTSTGGLIT TMLTAPNEDK KPIYQAKDIK DFYLENCPKI
151 FPKESRDTYD PIHSIGPIYD GEYLRELCNN LLKDLTVKDT LTDVIIPAFD
201 IKLLLPVIFS SDDAKCNALK NARLADVCIS TSAAPVLLPA HSFTTEDDKN
251 IHTFELIDGG VAATNPTLLA LTHIRNEIIR QNPRFIGANL TESKSRLVLS
301 LGTGKSEYKE KYNADMTSKW RLYNWALYNG NSPAVDIFSN ASSDMVDFHL
351 SALFK**SLDCE DYYLRIQDDT LTGEESSGHI ATEENLQR**LV EIGTELLEKQ
401 ESRINLDTGR LESIPGAPTN EAAIAKFAKL LSEERKLRQL K

Matched peptide information:

Start - End	Observed	Mr(expt)	Mr(calc)	ppm	Miss	Sequence
67 - 78	1200.6353	1199.6280	1199.6874	-50	0	K.ITVLSIDGGGIR.G (Ions score 66)
93 - 102	1099.4863	1098.4791	1098.5305	-47	0	K.LQDLDGPDAR.I (Ions score 52)
356 - 365	1333.5071	1332.4998	1332.5656	-49	0	K.SLDCEDYYLR.I (Ions score 64)
366 - 388	2543.0491	2542.0418	2542.1783	-54	0	R.IQDDTLTGEESSGHIATEENLQR.L (Ions score 219)

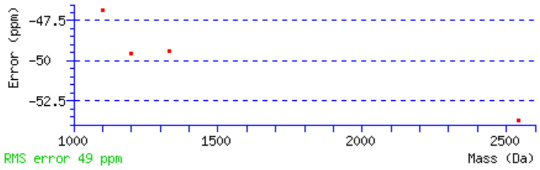

Spot No.:**14**

Accession No.: **scaffold4512_855.mRNA1**

Protein name：**Osmotin-like protein OSM34**

Peptide sequences:

R.NNCPYTVWAAASPGGGR.R;R.LDQGQTWELNVPAGTSMAR.I;K.CRPLFCTADINGQCPNQLK.A;K.APGGCNNPCTVFK.T;K.TNEYCCTEGYGTCGPTEFSK.F;R.CSDAYSYPQDDPSSTFTCPGGTNYR.V

PFF Mascot score: **[467]** Sequence coverage %: **[45]**

Matched peptides No.: **[6]**

Calculated Mr: **27682** Calculated *p*I: **5.23**

Data base searched result:

Ions score is -10*Log(P), where P is the probability that the observed match is a random event.
Individual ions scores > 30 indicate identity or extensive homology (p<0.05).
Protein scores are derived from ions scores as a non-probabilistic basis for ranking protein hits.

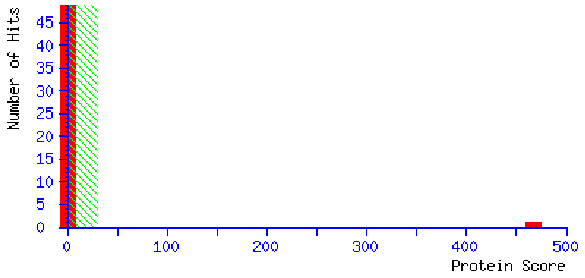

Matched peptide sequences: shown in Bold Red

```
  1 MSNFNIFLIS IFLLSALFFT SSDGATFTIR NNCPYTVWAA ASPGGGRRLD
 51 QGQTWELNVP AGTSMARIWG RTNCNFDGSG KGHCQTGDCG GILACQGWGV
101 PPNTLAEYAL NQFGNLDFYD ISLVDGFNIP IEFSPTSGAK DKCRPLFCTA
151 DINGQCPNQL KAPGGCNNPC TVFKTNEYCC TEGYGTCGPT EFSKFFKSRC
201 SDAYSYPQDD PSSTFTCPGG TNYRVVFCPA RSPHFPLEMV REKDVE
```

Matched peptide information:

Start - End	Observed	Mr(expt)	Mr(calc)	ppm	Miss	Sequence
31 - 47	1777.7087	1776.7015	1776.8002	-56	0	R.NNCPYTVWAAASPGGGR.R (Ions score 141)
49 - 67	2073.8826	2072.8753	2072.9949	-58	0	R.LDQGQTWELNVPAGTSMAR.I (Ions score 157)
143 - 161	2291.9358	2290.9285	2291.0609	-58	1	K.CRPLFCTADINGQCPNQLK.A (Ions score 28)
162 - 174	1421.5459	1420.5386	1420.6228	-59	0	K.APGGCNNPCTVFK.T (Ions score 46)
175 - 194	2360.7583	2359.7510	2359.9032	-64	0	K.TNEYCCTEGYGTCGPTEFSK.F (Ions score 80)
200 - 224	2845.9536	2844.9463	2845.1232	-62	0	R.CSDAYSYPQDDPSSTFTCPGGTNYR.V (Ions score 147)

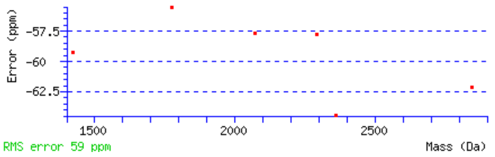

Spot No.:**15**

Accession No.: **scaffold0013_2366000.mRNA1**

Protein name： **Annexin-like protein RJ4**

Peptide sequences: **K.SSDYHVIVEIACVR.S;K.LLLGLVTAFR.Y;K.VLLGETADNEYKR.L**

PFF Mascot score: **[198]** Sequence coverage %: **[11]**

Matched peptides No.: **[3]**

Calculated Mr: **36064** Calculated *p*I: **7.72**

Data base searched result:

Ions score is -10*Log(P), where P is the probability that the observed match is a random event.
Individual ions scores > 31 indicate identity or extensive homology (p<0.05).
Protein scores are derived from ions scores as a non-probabilistic basis for ranking protein hits.

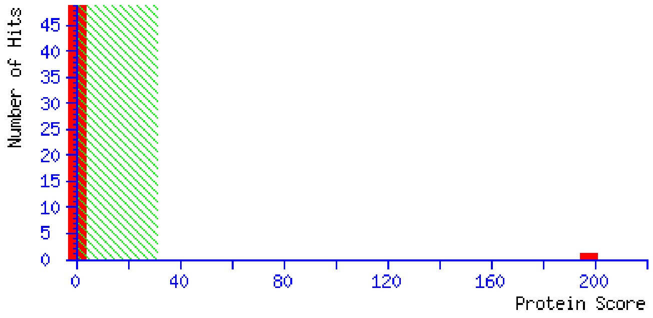

Matched peptide sequences: shown in Bold Red

```
  1 MATLVVPQNV SVVEDAENLR KAFEGWGTNE KVIISVLAHR NAAQRKQIRL
 51 AYWDLYQEDL VKRLESEIKG DFERAVYRWI LDPEDRDAVL ANVALKKSSD
101 YHVIVEIACV RSSEELLAVR RAYHARYKHS LEEDVAVHTT GDIRKLLLGL
151 VTAFRYEGAE VNSRLAKSEA DILHDAIKDK KINHDEVIRI LTTRSKTQLM
201 ATFNSFKDSQ GTSITKVLLG ETADNEYKRL LRIAIRCINE PIKYYEKVLR
251 NAFKKFGNDE DAITRVIVTR AEKDLQLIKE LYYSRNSVPL DQAVAKETSG
301 DYKDFLLALL GKQD
```

Matched peptide information:

Start - End	Observed	Mr(expt)	Mr(calc)	ppm	Miss	Sequence
98 - 111	1647.7398	1646.7325	1646.8086	-46	0	K.SSDYHVIVEIACVR.S (Ions score 110)
146 - 155	1102.6438	1101.6365	1101.6910	-49	0	K.LLLGLVTAFR.Y (Ions score 64)
217 - 229	1507.7044	1506.6971	1506.7678	-47	1	K.VLLGETADNEYKR.L (Ions score 87)

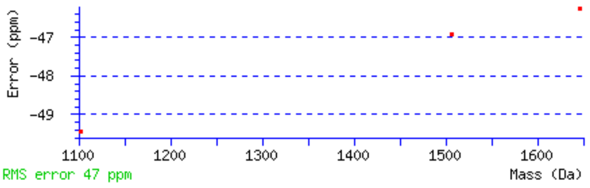

Spot No.:**16**

Accession No.: **scaffold0479_272073.mRNA1**

Protein name：**Obg-like ATPase 1**

Peptide sequences:

K.EAPAERPILGR.F;K.LSIPAENFPFCTIEPNEAR.V;K.SEVSAFLEIHDIAGLVR.G;R.GAHQGQGLGNNFLSHIR.A;R.AVDGIFHVLR.A;R.AFEDPDIIHVDDSVDPVR.D;R.DLEVIGNELR.L;K.IHAWVQEHGAEPIIPFSCVLER.N;K.APQAAGTIHSDFER.G

PFF Mascot score: **[689]** Sequence coverage %: **[35]**

Matched peptides No.: **[9]**

Calculated Mr: **44941** Calculated *p*I: **6.62**

Data base searched result:

Ions score is -10*Log(P), where P is the probability that the observed match is a random event.
Individual ions scores > 31 indicate identity or extensive homology (p<0.05).
Protein scores are derived from ions scores as a non-probabilistic basis for ranking protein hits.

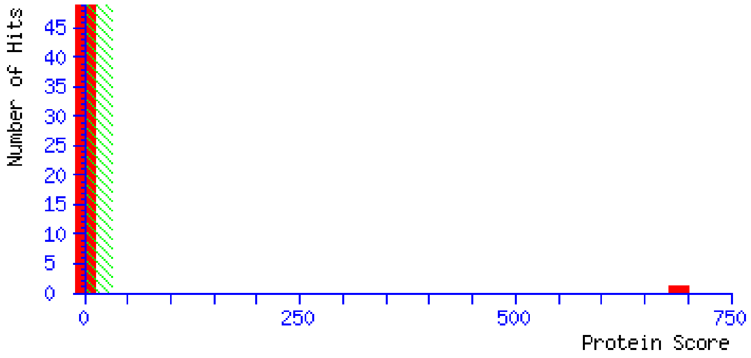

Matched peptide sequences: shown in Bold Red

1 MPPKSAKSKE **APAERPILGR** FSSHLKIGIV GLPNVGKSTL FNTLTK**LSIP**
51 **AENFPFCTIE PNEAR**VYIPD ERFEWLCQLY KPK**SEVSAFL EIHDIAGLVR**
101 **GAHQGQGLGN NFLSHIRAVD GIFHVLRAFE DPDIIHVDDS VDPVRDLEVI**
151 **GNELR**LKDIE FMDRRVEDLE KSMKRSNDKH LKIEHELCER VKAWLEGGKD
201 VRLGDWKAAD IEILNTFQLL TAKPVVYLVN MNEKDYQRKK NKFLPK**IHAW**
251 **VQEHGAEPII PFSCVLER**NL ADMAPDEATK YCEENKVQSA LPKIIKTGFS
301 AINLIYFFTA GPDEVKCWQI RRQTK**APQAA GTIHSDFER**G FICAEVMKFD
351 DLKELGSESA VKAAGKYRQE GKTYVVQDGD VIFFKFNVSG GGKK

Matched peptide information:

Start - End	Observed	Mr(expt)	Mr(calc)	ppm	Miss	Sequence
10 - 20	1208.6174	1207.6102	1207.6673	-47	1	K.EAPAERPILGR.F (Ions score 35)
47 - 65	2204.9790	2203.9717	2204.0572	-39	0	K.LSIPAENFPFCTIEPNEAR.V (Ions score 104)
84 - 100	1855.9220	1854.9147	1854.9839	-37	0	K.SEVSAFLEIHDIAGLVR.G (Ions score 163)
101 - 117	1805.8480	1804.8407	1804.9081	-37	0	R.GAHQGQGLGNNFLSHIR.A (Ions score 138)
118 - 127	1126.5896	1125.5823	1125.6295	-42	0	R.AVDGIFHVLR.A (Ions score 60)
128 - 145	2038.8877	2037.8804	2037.9644	-41	0	R.AFEDPDIIHVDDSVDPVR.D (Ions score 123)
146 - 155	1157.5681	1156.5608	1156.6088	-41	0	R.DLEVIGNELR.L (Ions score 75)
247 - 268	2588.1975	2587.1902	2587.3006	-43	0	K.IHAWVQEHGAEPIIPFSCVLER.N (Ions score 94)
326 - 339	1499.6660	1498.6587	1498.7164	-39	0	K.APQAAGTIHSDFER.G (Ions score 133)

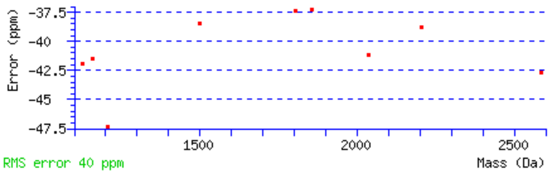

Spot No.:**17**

Accession No.: **scaffold0155_515853.mRNA1**

Protein name：**Pro-hevein**

Peptide sequences:

K.YGWTAFCGPVGAHGQPSCGK.C;R.IVDQCSNGGLDLDVNVFR.Q;R.QLDTDGKGYER.G

PFF Mascot score: **[211]**   Sequence coverage %: **[23]**

Matched peptides No.: **[3]**

Calculated Mr: **23042**   Calculated *p*I: **8.15**

Data base searched result:

Ions score is -10*Log(P), where P is the probability that the observed match is a random event.
Individual ions scores > 30 indicate identity or extensive homology (p<0.05).
Protein scores are derived from ions scores as a non-probabilistic basis for ranking protein hits.

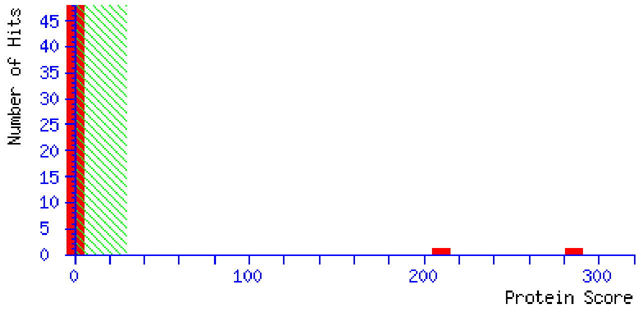

Matched peptide sequences: shown in Bold Red

```
  1 MGRVMNIFIV VLLCLTGVAI AEQCGRQAGG KLCPNNLCCS QYGWCGSSDD
 51 YCSPSKNCQS NCKGGGGGGG GGGGSASNVL ATYHLYNPQQ HGWDLNAVSA
101 YCSTWDANKP YSWRSKYGWT AFCGPVGAHG QPSCGKCLSV TNTGTGAKTT
151 VRIVDQCSNG GLDLDVNVFR QLDTDGKGYE RGHLTVNYQF VNCGDSFNPL
201 FSIMKSSVIN
```

Matched peptide information:

Start - End	Observed	Mr(expt)	Mr(calc)	ppm	Miss	Sequence
117 - 136	2136.8611	2135.8538	2135.9306	-36	0	K.YGWTAFCGPVGAHGQPSCGK.C (Ions score 97)
153 - 170	2020.9143	2019.9070	2019.9684	-30	0	R.IVDQCSNGGLDLDVNVFR.Q (Ions score 196)
171 - 181	1281.5598	1280.5525	1280.5997	-37	1	R.QLDTDGKGYER.G (Ions score 52)

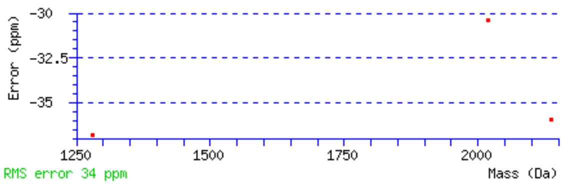

Spot No.:**18**

Accession No.: **scaffold1222_136753.mRNA1**

Protein name：**Rubber elongation factor protein**

Peptide sequences:

K.YLGFVQDAATYAVTTFSNVYLFAK.D;K.NVAVPLYNR.F;R.FSYIPNGALK.F;K.FVDSTVVASVTIIDR.S;K.DASIQVVSAIR.A

PFF Mascot score: **[313]** Sequence coverage %: **[50]**

Matched peptides No.: **[5]**

Calculated Mr: **14713** Calculated *p*I: **5.04**

Data base searched result:

Ions score is -10*Log(P), where P is the probability that the observed match is a random event.
Individual ions scores > 31 indicate identity or extensive homology (p<0.05).
Protein scores are derived from ions scores as a non-probabilistic basis for ranking protein hits.

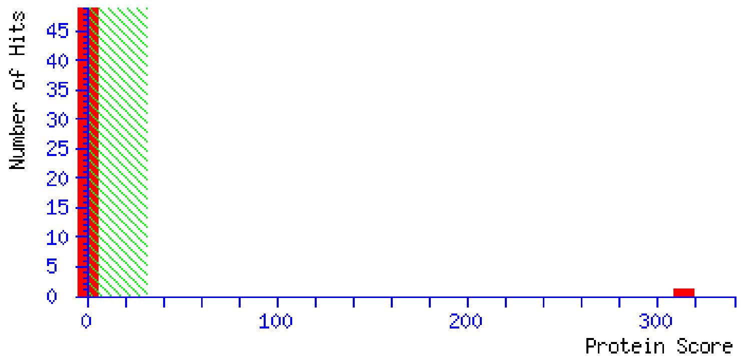

Matched peptide sequences: shown in Bold Red

```
  1 MAEDEDNQQG QGEGLKYLGF VQDAATYAVT TFSNVYLFAK DKSGPLQPGV
 51 DIIEGPVKNV AVPLYNRFSY IPNGALKFVD STVVASVTII DRSLPPIVKD
101 ASIQVVSAIR AAPEAARSLA SSLPGQTKIL AKVFYGEN
```

Matched peptide information:

Start - End	Observed	Mr(expt)	Mr(calc)	ppm	Miss	Sequence
17 - 40	2689.2585	2688.2513	2688.3476	-36	0	K.YLGFVQDAATYAVTTFSNVYLFAK.D (Ions score 79)
59 - 67	1045.5446	1044.5373	1044.5716	-33	0	K.NVAVPLYNR.F (Ions score 74)
68 - 77	1109.5523	1108.5450	1108.5917	-42	0	R.FSYIPNGALK.F (Ions score 51)
78 - 92	1621.8351	1620.8278	1620.8723	-27	0	K.FVDSTVVASVTIIDR.S (Ions score 145)
100 - 110	1158.6145	1157.6072	1157.6404	-29	0	K.DASIQVVSAIR.A (Ions score 82)

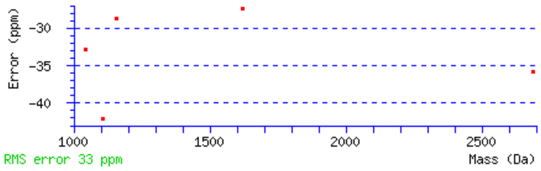

Spot No.:**19**

Accession No.: **scaffold1093_209300.mRNA1**

Protein name：**Pro-hevein**

Peptide sequences: K.YGWTAFCGPVGPHGQASCGK.C;R.IVDQCSNGGLDLDVNVFR.K

PFF Mascot score: **[250]** Sequence coverage %: **[36]**

Matched peptides No.: **[2]**

Calculated Mr: **11842** Calculated *p*I: **6.79**

Data base searched result:

Ions score is -10*Log(P), where P is the probability that the observed match is a random event.
Individual ions scores > 30 indicate identity or extensive homology (p<0.05).
Protein scores are derived from ions scores as a non-probabilistic basis for ranking protein hits.

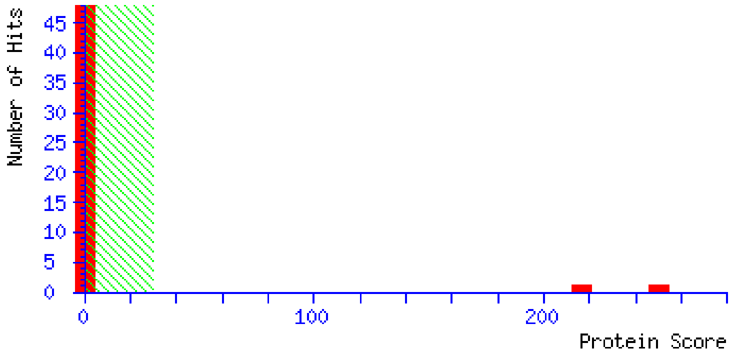

Matched peptide sequences: shown in Bold Red

```
  1 MSESYSWRSK YGWTAFCGPV GPHGQASCGK CLRVTNTRTG AKTTVRIVDQ
 51 CSNGGLDLDV NVFRKLDTDG KGYEQGHLTV NYEFVNCGDS FNPLFSIMDH
101 QYIN
```

Matched peptide information:

Start - End	Observed	Mr(expt)	Mr(calc)	ppm	Miss	Sequence	
11 - 30	2136.8860	2135.8787	2135.9306	-24	0	K.YGWTAFCGPVGPHGQASCGK.C	(Ions score 115)
47 - 64	2020.9313	2019.9240	2019.9684	-22	0	R.IVDQCSNGGLDLDVNVFR.K	(Ions score 165)

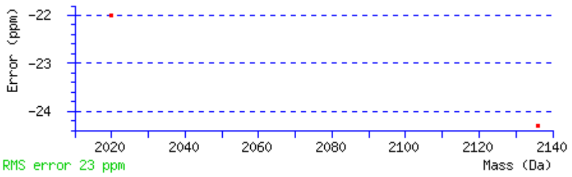

Spot No.:**20**

Accession No.: **scaffold0625_11329.mRNA1**

Protein name：**Glucan endo-1,3-beta-glucosidase, basic vacuolar isoform**

Peptide sequences:

R.IYDPNQAVLEALR.G;R.YIAVGNEISPVNR.G;R.GTAWLAQFVLPAMR.N;K.VSTAIDLTLVGNSYPPSAGAFR.D;R.SYLNPIIR.F;R.SPLLANIYPYFTYAGNPR.D;R.DISLPYALFTSPSVVVWDGQR.G;K.NLFDATLDALYSALER.A;R.ASGGSLEVVVSESGWPSAGAFAATFDNGR.T

PFF Mascot score: **[1073]** Sequence coverage %: **[41]**

Matched peptides No.: **[9]**

Calculated Mr: **41512** Calculated *p*I: **9.27**

Data base searched result:

Ions score is -10*Log(P), where P is the probability that the observed match is a random event.
Individual ions scores > 31 indicate identity or extensive homology (p<0.05).
Protein scores are derived from ions scores as a non-probabilistic basis for ranking protein hits.

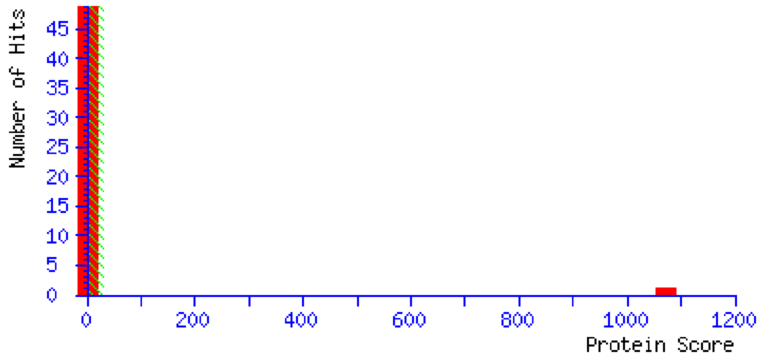

Matched peptide sequences: shown in Bold Red

```
  1 MAICSSTSGT SSSLPSRTTV MLLLFFFTAS VGITDAQVGV CYGMQGNNLP
 51 PVSEVIALYK KSNITRMRIY DPNQAVLEAL RGSNIELILG VPNSDLQSLT
101 NPSNAKSWVQ KNVRGFWSSV RFRYIAVGNE ISPVNRGTAW LAQFVLPAMR
151 NIHDAIRSAG LQDQIKVSTA IDLTLVGNSY PPSAGAFRDD VRSYLNPIIR
201 FLSSIRSPLL ANIYPYFTYA GNPRDISLPY ALFTSPSVVV WDGQRGYKNL
251 FDATLDALYS ALERASGGSL EVVVSESGWP SAGAFAATFD NGRTYLSNLI
301 QHVKRGTPKR PNRAIETYLF AMFDENKKQP EVEKHFGLFF PDKRPKYNLN
351 FGAEKNWDIS TEHNATILFL KSDM
```

Matched peptide information:

Start - End	Observed	Mr(expt)	Mr(calc)	ppm	Miss	Sequence
69 - 81	1501.7711	1500.7638	1500.7936	-20	0	R.IYDPNQAVLEALR.G (Ions score 127)
124 - 136	1431.7299	1430.7226	1430.7518	-20	0	R.YIAVGNEISPVNR.G (Ions score 111)
137 - 150	1560.8025	1559.7952	1559.8282	-21	0	R.GTAWLAQFVLPAMR.N (Ions score 110)
167 - 188	2236.1208	2235.1136	2235.1535	-18	0	K.VSTAIDLTLVGNSYPPSAGAFR.D (Ions score 218)
193 - 200	975.5377	974.5304	974.5549	-25	0	R.SYLNPIIR.F (Ions score 45)
207 - 224	2057.0098	2056.0025	2056.0418	-19	0	R.SPLLANIYPYFTYAGNPR.D (Ions score 154)
225 - 245	2350.1624	2349.1551	2349.2005	-19	0	R.DISLPYALFTSPSVVVWDGQR.G (Ions score 198)
249 - 264	1811.8868	1810.8796	1810.9101	-17	0	K.NLFDATLDALYSALER.A (Ions score 162)
265 - 293	2826.2695	2825.2623	2825.3257	-22	0	R.ASGGSLEVVVSESGWPSAGAFAATFDNGR.T (Ions score 190)

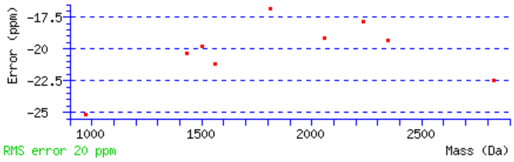

Spot No.:**21**

Accession No.: **scaffold0331_686228.mRNA1**

Protein name：**Vacuolar protein sorting-associated protein 2 homolog 1**

Peptide sequences:

R.KTPAELLR.E;K.SQLQGVSLR.I;K.IMQEFER.Q;K.VAQVETTGNDDSGIDSELQAR.L

PFF Mascot score: **[231]** Sequence coverage %: **[20]**

Matched peptides No.: **[4]**

Calculated Mr: **25163** Calculated *p*I: **5.55**

Data base searched result:

Ions score is -10*Log(P), where P is the probability that the observed match is a random event.
Individual ions scores > 31 indicate identity or extensive homology (p<0.05).
Protein scores are derived from ions scores as a non-probabilistic basis for ranking protein hits.

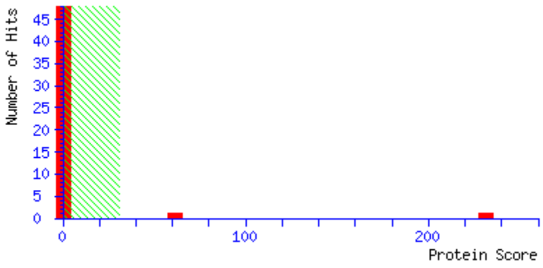

Matched peptide sequences: shown in Bold Red

```
  1 MSFLFGKRKT PAELLRENKR MLDKSIREIE RERQGLQAQE KKLIAEIKKS
 51 AKQGQMGAVK VMAKDLIRTR HQIEKFYKLK SQLQGVSLRI QTLKSTQAMG
101 EAMKGVTKAM GQMNRQMNLP SLQKIMQEFE RQNERMEMVT EVMGDAIDDA
151 LEGDEEEEET EELVNQVLDE IGIDINQELV NAPSSAVAAP AAKGKVAQVE
201 TTGNDDSGID SELQARLDNL RRM
```

Matched peptide information:

Start - End	Observed	Mr(expt)	Mr(calc)	ppm	Miss	Sequence
9 - 16	927.5458	926.5386	926.5549	-18	1	R.KTPAELLR.E (Ions score 8)
81 - 89	987.5403	986.5330	986.5509	-18	0	K.SQLQGVSLR.I (Ions score 48)
125 - 131	952.4347	951.4274	951.4484	-22	0	K.IMQEFER.Q (Ions score 39)
196 - 216	2205.0061	2203.9988	2204.0193	-9	0	K.VAQVETTGNDDSGIDSELQAR.L (Ions score 207)

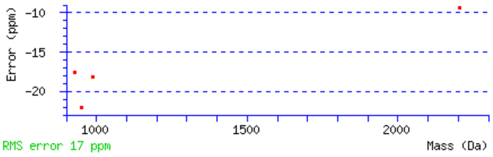

Spot No.:22

Accession No.: scaffold0625_11329.mRNA1

Protein name：Glucan endo-1,3-beta-glucosidase, basic vacuolar isoform

Peptide sequences:

R.IYDPNQAVLEALR.G;K.VSTAIDLTLVGNSYPPSAGAFR.D;R.SPLLANIYPYFTYAGNPR.D;R.DISLPYALFTSPSVVVWDGQR.G;K.NLFDATLDALYSALER.A

PFF Mascot score: [375] Sequence coverage %: [24]

Matched peptides No.: [5]

Calculated Mr: 41512 Calculated *p*I: 9.27

Data base searched result:

Ions score is -10*Log(P), where P is the probability that the observed match is a random event.
Individual ions scores > 30 indicate identity or extensive homology ($p<0.05$).
Protein scores are derived from ions scores as a non-probabilistic basis for ranking protein hits.

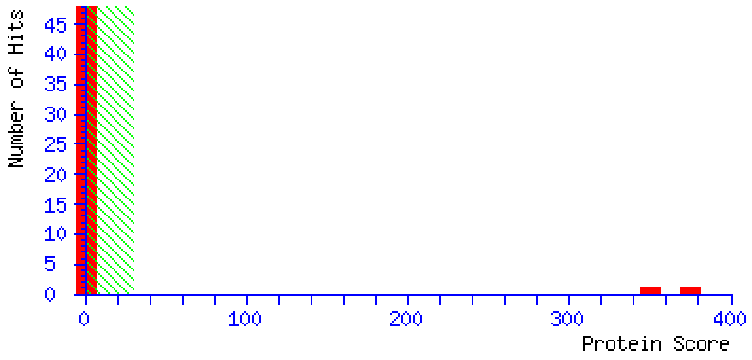

Matched peptide sequences: shown in Bold Red

```
  1 MAICSSTSGT SSSLPSRTTV MLLLFFFTAS VGITDAQVGV CYGMQGNNLP
 51 PVSEVIALYK KSNITRMRIY DPNQAVLEAL RGSNIELILG VPNSDLQSLT
101 NPSNAKSWVQ KNVRGFWSSV RFRYIAVGNE ISPVNRGTAW LAQFVLPAMR
151 NIHDAIRSAG LQDQIKVSTA IDLTLVGNSY PPSAGAFRDD VRSYLNPIIR
201 FLSSIRSPLL ANIYPYFTYA GNPRDISLPY ALFTSPSVVV WDGQRGYKNL
251 FDATLDALYS ALERASGGSL EVVVSESGWP SAGAFAATFD NGRTYLSNLI
301 QHVKRGTPKR PNRAIETYLF AMFDENKKQP EVEKHFGLFF PDKRPKYNLN
351 FGAEKNWDIS TEHNATILFL KSDM
```

Matched peptide information:

Start - End	Observed	Mr(expt)	Mr(calc)	ppm	Miss	Sequence
69 - 81	1501.7783	1500.7710	1500.7936	-15	0	R.IYDPNQAVLEALR.G (Ions score 79)
167 - 188	2236.1335	2235.1263	2235.1535	-12	0	K.VSTAIDLTLVGNSYPPSAGAFR.D (Ions score 112)
207 - 224	2057.0188	2056.0115	2056.0418	-15	0	R.SPLLANIYPYFTYAGNPR.D (Ions score 85)
225 - 245	2350.1746	2349.1673	2349.2005	-14	0	R.DISLPYALFTSPSVVVWDGQR.G (Ions score 108)
249 - 264	1811.8964	1810.8891	1810.9101	-12	0	K.NLFDATLDALYSALER.A (Ions score 103)

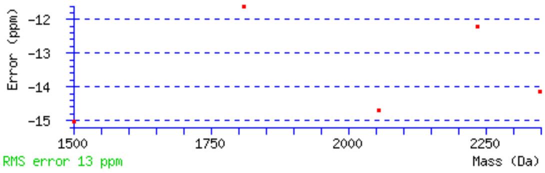

Spot No.:**23**

Accession No.: **scaffold1735_70085.mRNA1**

Protein name：**ricin B-like lectin EULS3**

Peptide sequences: **K.TESESSHCR.K;R.KPHLPSGSYLSK.K; K.ADPNFFLTIR.D;K.VVLAPPDPSDEFQHWYK.D;K.VVLAPPDPSDEFQHWYKDEK.F;K.DEEGFPSFALVNK.A;K.HSFGPTHPVLLTPYDPDDLEASILWTESK.D;K.VVLAYWNK.G**

PFF Mascot score: **[425]** Sequence coverage %: **[55]**

Matched peptides No.: **[8]**

Calculated Mr: **20724** Calculated *p*I: **7.77**

Data base searched result:

Ions score is -10*Log(P), where P is the probability that the observed match is a random event.
Individual ions scores > 31 indicate identity or extensive homology (p<0.05).
Protein scores are derived from ions scores as a non-probabilistic basis for ranking protein hits.

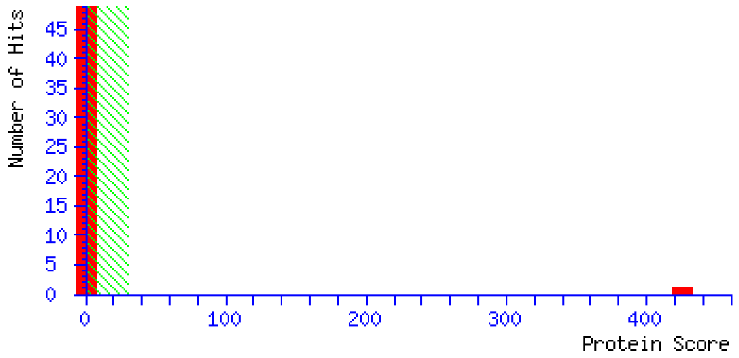

Matched peptide sequences: shown in Bold Red

```
  1 MEELGSSHEK TESESSHCRK PHLPSGSYLS KKPSYKVYCK ADPNFFLTIR
 51 DGKVVLAPPD PSDEFQHWYK DEKFSTMVKD EEGFPSFALV NKASGKALKH
101 SFGPTHPVLL TPYDPDDLEA SILWTESKDL GDGYRIVRML NNIRLNVEAL
151 RGDKKSGGVS NGTKVVLAYW NKGDHQKWRI APL
```

Matched peptide information:

Start	-	End	Observed	Mr(expt)	Mr(calc)	ppm	Miss	Sequence
11	-	19	1092.4293	1091.4220	1091.4302	-7	0	K.TESESSHCR.K (Ions score 51)
20	-	31	1313.7057	1312.6984	1312.7139	-12	1	R.KPHLPSGSYLSK.K (Ions score 73)
41	-	50	1193.6023	1192.5950	1192.6241	-24	0	K.ADPNFFLTIR.D (Ions score 60)
54	-	70	2027.9434	2026.9361	2026.9789	-21	0	K.VVLAPPDPSDEFQHWYK.D (Ions score 102)
54	-	73	2400.1013	2399.0940	2399.1434	-21	1	K.VVLAPPDPSDEFQHWYKDEK.F (Ions score 98)
80	-	92	1452.6652	1451.6579	1451.6933	-24	0	K.DEEGFPSFALVNK.A (Ions score 104)
100	-	128	3265.5303	3264.5230	3264.5979	-23	0	K.HSFGPTHPVLLTPYDPDDLEASILWTESK.D (Ions score 75)
165	-	172	992.5263	991.5190	991.5491	-30	0	K.VVLAYWNK.G (Ions score 59)

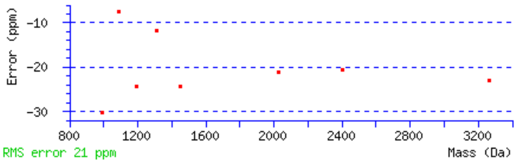

Spot No.:**24**

Accession No.: **scaffold0155_515853.mRNA1**

Protein name：**Pro-hevein**

Peptide sequences: K.YGWTAFCGPVGAHGQPSCGK.C;R.IVDQCSNGGLDLDVNVFR.Q

PFF Mascot score: **[235]** Sequence coverage %: **[18]**

Matched peptides No.: **[2]**

Calculated Mr: **23042** Calculated *p*I: **8.15**

Data base searched result:

Ions score is -10*Log(P), where P is the probability that the observed match is a random event.
Individual ions scores > 30 indicate identity or extensive homology ($p<0.05$).
Protein scores are derived from ions scores as a non-probabilistic basis for ranking protein hits.

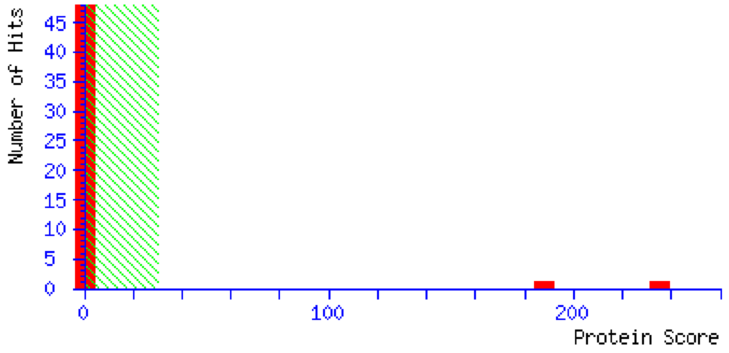

Matched peptide sequences: shown in Bold Red

```
  1 MGRVMNIFIV VLLCLTGVAI AEQCGRQAGG KLCPNNLCCS QYGWCGSSDD
 51 YCSPSKNCQS NCKGGGGGGG GGGGSASNVL ATYHLYNPQQ HGWDLNAVSA
101 YCSTWDANKP YSWRSKYGWT AFCGPVGAHG QPSCGKCLSV TNTGTGAKTT
151 VRIVDQCSNG GLDLDVNVFR QLDTDGKGYE RGHLTVNYQF VNCGDSFNPL
201 FSIMKSSVIN
```

Matched peptide information:

Start - End	Observed	Mr(expt)	Mr(calc)	ppm	Miss	Sequence	
117 - 136	2136.8762	2135.8689	2135.9306	-29	0	K.YGWTAFCGPVGAHGQPSCGK.C	(Ions score 83)
153 - 170	2020.9286	2019.9213	2019.9684	-23	0	R.IVDQCSNGGLDLDVNVFR.Q	(Ions score 182)

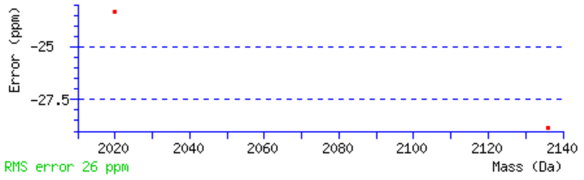

Spot No.: **25**

Accession No.: **scaffold0370_918136.mRNA1**

Protein name：**Proteasome subunit beta type-4**

Peptide sequences:

K.YKDGILMAADMAASYGSTLR.Y;K.DGILMAADMAASYGSTLR.Y;K.HSLLGASGEISDFQEILR.Y;R.YLDELILYDNMWDDGNSLGPK.E;K.EVHSYLTR.V;K.FNPLWNSLVLGGVK.N;K.YLGMVSMIGVNFEDNHVATGFGNHLAR.P;R.DEWHENLSFEDGVK.L;K.ITEEGVTISQPYALK.T

PFF Mascot score: **[823]** Sequence coverage %: **[55]**

Matched peptides No.: **[9]**

Calculated Mr: **27904** Calculated *p*I: **6.97**

Data base searched result:

Ions score is -10*Log(P), where P is the probability that the observed match is a random event.
Individual ions scores > 30 indicate identity or extensive homology (p<0.05).
Protein scores are derived from ions scores as a non-probabilistic basis for ranking protein hits.

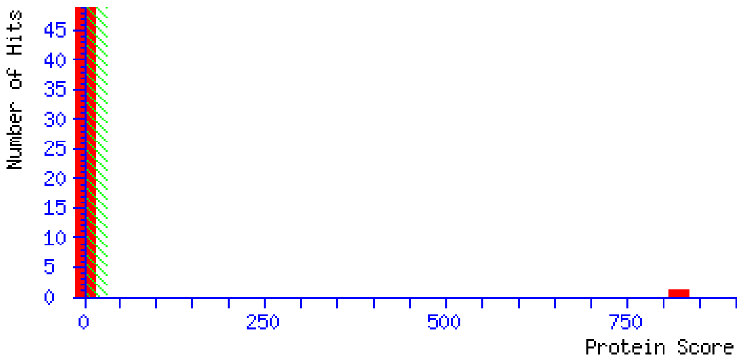

Matched peptide sequences: shown in Bold Red

1 MATTMVKENN SGPAQLLGPE SAFERTLYPY VTGTSVVALK **YKDGILMAAD**
51 **MAASYGSTLR** YKSVERMKSI GK**HSLLGASG** **EISDFQEILR** **YLDELILYDN**
101 **MWDDGNSLGP** **KEVHSYLTR**V MYNRRNK**FNP** **LWNSLVLGGV** K**NGQKYLGMV**
151 **SMIGVNFEDN** **HVATGFGNHL** **AR**PILR**DEWH** **ENLSFEDGVK** LLEKCMRVLL
201 YRDRSAINKL QIAK**ITEEGV** **TISQPYALK**T FWGFSAFQNP TVGAEGSW

Matched peptide information:

Start - End	Observed	Mr(expt)	Mr(calc)	ppm	Miss	Sequence
41 - 60	2133.9895	2132.9822	2133.0234	-19	1	K.YKDGILMAADMAASYGSTLR.Y (Ions score 144)
43 - 60	1842.8340	1841.8267	1841.8651	-21	0	K.DGILMAADMAASYGSTLR.Y (Ions score 130)
73 - 90	1971.9761	1970.9688	1971.0061	-19	0	K.HSLLGASGEISDFQEILR.Y (Ions score 201)
91 - 111	2471.0864	2470.0792	2470.1362	-23	0	R.YLDELILYDNMWDDGNSLGPK.E (Ions score 127)
112 - 119	1004.4935	1003.4862	1003.5087	-22	0	K.EVHSYLTR.V (Ions score 53)
128 - 141	1543.8209	1542.8136	1542.8558	-27	0	K.FNPLWNSLVLGGVK.N (Ions score 87)
146 - 172	2949.3362	2948.3289	2948.4062	-26	0	K.YLGMVSMIGVNFEDNHVATGFGNHLAR.P (Ions score 121)
177 - 190	1704.7046	1703.6973	1703.7427	-27	0	R.DEWHENLSFEDGVK.L (Ions score 116)
215 - 229	1648.8319	1647.8246	1647.8719	-29	0	K.ITEEGVTISQPYALK.T (Ions score 82)

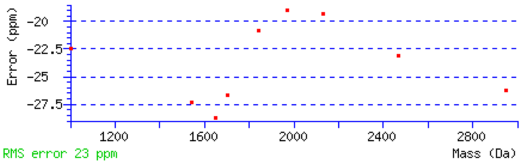

Spot No.:**26**

Accession No.: **scaffold0625_426005.mRNA1**

Protein name：**Acylpyruvase FAHD1, mitochondrial**

Peptide sequences:

R.DVPQTTAMDYVGGYALALDMTAR.E;K.GQDTFTPISSVLPK.S;K.SAVPDPDNLELWLK.V;K.ITAGITDLLDVHFNVEK.R

PFF Mascot score: **[382]** Sequence coverage %: **[30]**

Matched peptides No.: **[4]**

Calculated Mr: **24248** Calculated *p*I: **6.52**

Data base searched result:

Ions score is -10*Log(P), where P is the probability that the observed match is a random event.
Individual ions scores > 31 indicate identity or extensive homology ($p<0.05$).
Protein scores are derived from ions scores as a non-probabilistic basis for ranking protein hits.

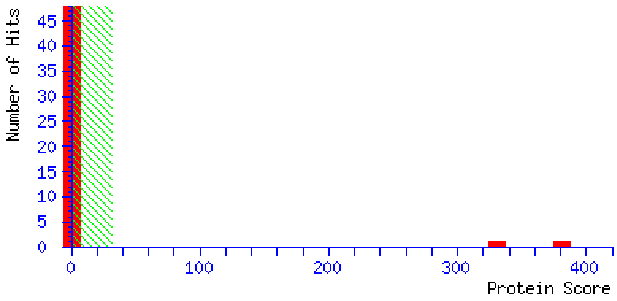

Matched peptide sequences: shown in Bold Red

```
  1 MATASSGVQK LLQVGTKIVA VGRNYAAHAK ELGNAVPKEP VLFLKPTSSY
 51 LENGGTIEIP HPLESLDHEV ELAVVIGQKA RDVPQTTAMD YVGGYALALD
101 MTAREIQASA KSAGLPWSVA KGQDTFTPIS SVLPKSAVPD PDNLELWLKV
151 DGDMRQKGST KDMIFKIPYL ISHISSIMTL FEGDVILTGT PQGVGPVKVG
201 QKITAGITDL LDVHFNVEKR RRPGSY
```

Matched peptide information:

Start - End	Observed	Mr(expt)	Mr(calc)	ppm	Miss	Sequence
82 - 104	2459.1016	2458.0943	2458.1508	-23	0	R.DVPQTTAMDYVGGYALALDMTAR.E (Ions score 201)
122 - 135	1489.7419	1488.7347	1488.7824	-32	0	K.GQDTFTPISSVLPK.S (Ions score 66)
136 - 149	1596.7777	1595.7704	1595.8195	-31	0	K.SAVPDPDNLELWLK.V (Ions score 77)
203 - 219	1884.9579	1883.9506	1883.9993	-26	0	K.ITAGITDLLDVHFNVEK.R (Ions score 127)

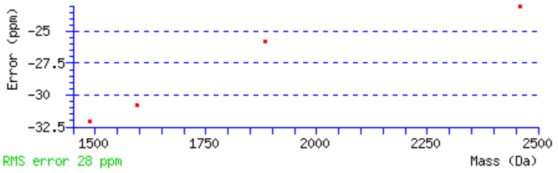

Spot No.:27

Accession No.: scaffold0564_474434.mRNA1

Protein name：Uncharacterized protein

Peptide sequences:

K.VGPDEEDNGGLVR.D;R.DFEELKPEVESVK.E;K.EVTNIIASAEPIESLPQELSSTVEASIEK.S;K.LPSSDVSNV IETDIVSEVR.G;K.VLPSLDENGGEPPALADVESK.R;K.SCCGLFEVLR.R

PFF Mascot score: [360] Sequence coverage %: [33]

Matched peptides No.: [6]

Calculated Mr: 33888 Calculated *p*I: 4.17

Data base searched result:

Ions score is -10*Log(P), where P is the probability that the observed match is a random event.
Individual ions scores > 30 indicate identity or extensive homology (p<0.05).
Protein scores are derived from ions scores as a non-probabilistic basis for ranking protein hits.

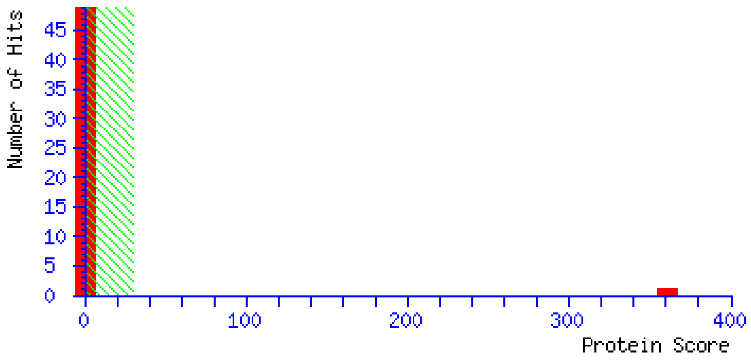

Matched peptide sequences: shown in Bold Red

```
  1 MEEFSVGKVD ATQKVGPDEE DNGGLVRDFE ELKPEVESVK EVVDENSQSL
 51 KETHDMLSSD SGNTPEEEAV DKDQSPSGKD SGTLKEEVEK EVTNIIASAE
101 PIESLPQELS STVEASIEKS KESDANEGKI FPSSNENNEM APVAADGVLN
151 VTKKIISPPT TLDEIVGDLS EEVSGGPEEI KLPSSDVSNV IETDIVSEVR
201 GIEEKVLPSL DENGGEPPAL ADVESKRVEE AKMVALEDDN GESSGIVDKE
251 SVESDDDSLK PSNNASIIGS SDGFPESTEH PHVISLNQRT LQDASLKSCC
301 GLFEVLRRSN GWLKI
```

Matched peptide information:

Start - End	Observed	Mr(expt)	Mr(calc)	ppm	Miss	Sequence
15 - 27	1356.5890	1355.5817	1355.6317	-37	0	K.VGPDEEDNGGLVR.D (Ions score 118)
28 - 40	1548.7181	1547.7109	1547.7719	-39	1	R.DFEELKPEVESVK.E (Ions score 62)
91 - 119	3084.4651	3083.4578	3083.5761	-38	0	K.EVTNIIASAEPIESLPQELSSTVEASIEK.S (Ions score 12)
182 - 200	2058.9788	2057.9715	2058.0481	-37	0	K.LPSSDVSNVIETDIVSEVR.G (Ions score 166)
206 - 226	2136.9780	2135.9708	2136.0586	-41	0	K.VLPSLDENGGEPPALADVESK.R (Ions score 50)
298 - 307	1240.5333	1239.5261	1239.5740	-39	0	K.SCCGLFEVLR.R (Ions score 82)

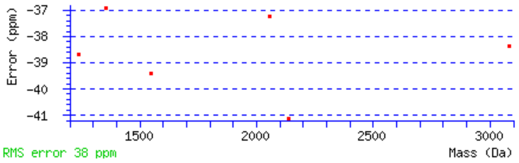

Spot No.: **28**

Accession No.: **scaffold0745_297880.mRNA1**

Protein name：**Elongation factor 1-alpha**

Peptide sequences:

K.YYCTVIDAPGHR.D;K.IGGIGTVPVGR.V;R.VETGILKPGMVVTFGPSGLTTEVK.S

PFF Mascot score: **[136]** Sequence coverage %: **[10]**

Matched peptides No.: **[3]**

Calculated Mr: **49754** Calculated *p*I: **9.20**

Data base searched result:

Ions score is -10*Log(P), where P is the probability that the observed match is a random event.
Individual ions scores > 31 indicate identity or extensive homology (p<0.05).
Protein scores are derived from ions scores as a non-probabilistic basis for ranking protein hits.

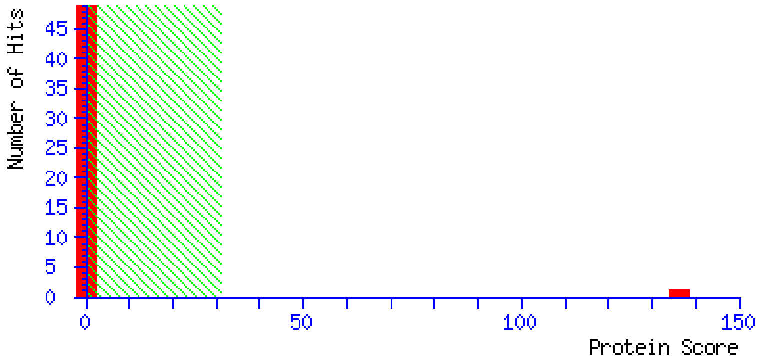

Matched peptide sequences: shown in Bold Red

```
  1 MGKEKVHINI VVIGHVDSGK STTTGHLIYK LGGIDKRVIE RFEKEAAEMN
 51 KRSFKYAWVL DKLKAERERG ITIDIALWKF ETTKYYCTVI DAPGHRDFIK
101 NMITGTSQAD CAVLIIDSTT GGFEAGISKD GQTREHALLA FTLGVRQMIC
151 CCNKMDATTP KYSKARYDEI VKEVASYLKK VGYNPEKIPF VPISGFEGDN
201 MIQRSTNLDW YKGPTLLEAL DQILEPKRPS DKPLRLPLQD VYKIGGIGTV
251 PVGRVETGIL KPGMVVTFGP SGLTTEVKSV EMHHEALQEA LPGDNVGFNV
301 KNVAVKDLKR GFVASNSKDD PAKEAANFTA QVIIMNHPGQ IGNGYAPVLD
351 CHTSHIAVKF AEILTKIDRR SGKELEKEPK FLKNGDAGFV KMIPTKPMVV
401 ETFSEYPPLG RFAVRDMRQT VAVGVIKSVE KKDPSGAKVT KSAAKKGGK
```

Matched peptide information:

Start - End	Observed	Mr(expt)	Mr(calc)	ppm	Miss	Sequence
85 - 96	1451.6102	1450.6030	1450.6663	-44	0	K.YYCTVIDAPGHR.D (Ions score 74)
244 - 254	1025.5663	1024.5590	1024.6030	-43	0	K.IGGIGTVPVGR.V (Ions score 45)
255 - 278	2460.2312	2459.2239	2459.3346	-45	1	R.VETGILKPGMVVTFGPSGLTTEVK.S (Ions score 75)

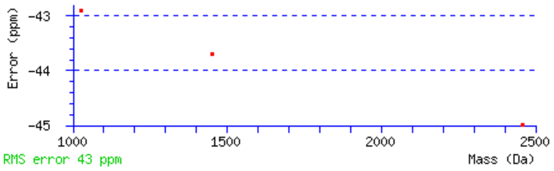

Spot No.:**29**

Accession No.: **scaffold0155_515853.mRNA1**

Protein name： **Pro-hevein**

Peptide sequences: **K.YGWTAFCGPVGAHGQPSCGK.C;R.IVDQCSNGGLDLDVNVFR.Q**

PFF Mascot score: **[268]** Sequence coverage %: **[18]**

Matched peptides No.: **[2]**

Calculated Mr: **23042** Calculated *p*I: **8.15**

Data base searched result:

Ions score is -10*Log(P), where P is the probability that the observed match is a random event.
Individual ions scores > 31 indicate identity or extensive homology (p<0.05).
Protein scores are derived from ions scores as a non-probabilistic basis for ranking protein hits.

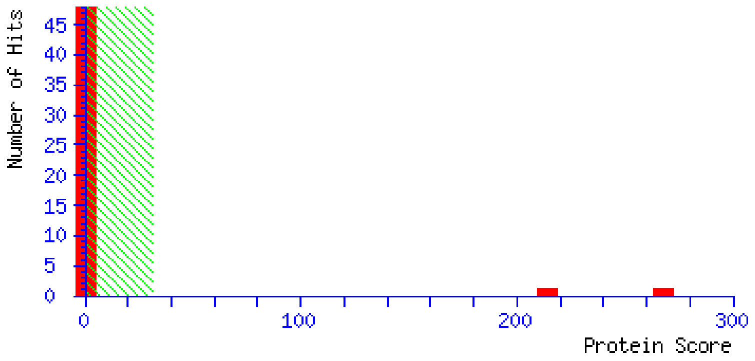

Matched peptide sequences: shown in Bold Red

1 MGRVMNIFIV VLLCLTGVAI AEQCGRQAGG KLCPNNLCCS QYGWCGSSDD
51 YCSPSKNCQS NCKGGGGGGG GGGGSASNVL ATYHLYNPQQ HGWDLNAVSA
101 YCSTWDANKP YSWRSK**YGWT** **AFCGPVGAHG** **QPSCGK**CLSV TNTGTGAKTT
151 VR**IVDQCSNG** **GLDLDVNVFR** QLDTDGKGYE RGHLTVNYQF VNCGDSFNPL
201 FSIMKSSVIN

Matched peptide information:

Start - End	Observed	Mr(expt)	Mr(calc)	ppm	Miss	Sequence	
117 - 136	2136.8162	2135.8089	2135.9306	-57	0	K.YGWTAFCGPVGAHGQPSCGK.C	(Ions score 116)
153 - 170	2020.8679	2019.8606	2019.9684	-53	0	R.IVDQCSNGGLDLDVNVFR.Q	(Ions score 182)

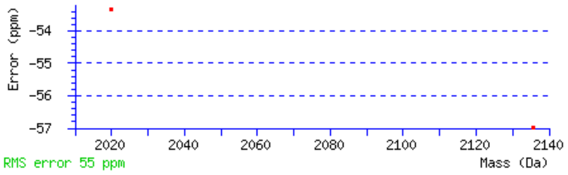

Spot No.:**30**

Accession No.: **scaffold0625_11329.mRNA1**

Protein name：**Glucan endo-1,3-beta-glucosidase, basic vacuolar isoform**

Peptide sequences:

R.IYDPNQAVLEALR.G;R.YIAVGNEISPVNR.G;R.GTAWLAQFVLPAMR.N;K.VSTAIDLTLVGNSYPPSAGAFR.D;K.VSTAIDLTLVGNSYPPSAGAFRDDVR.S;R.SYLNPIIR.F;R.SPLLANIYPYFTYAGNPR.D;R.DISLPYALFTSPSVVVWDGQR.G;K.NLFDATLDALYSALER.A;R.ASGGSLEVVVSESGWPSAGAFAATFDNGR.T

PFF Mascot score: **[846]** Sequence coverage %: **[42]**

Matched peptides No.: **[10]**

Calculated Mr: **41512** Calculated *p*I: **9.27**

Data base searched result:

Ions score is -10*Log(P), where P is the probability that the observed match is a random event.
Individual ions scores > 31 indicate identity or extensive homology ($p<0.05$).
Protein scores are derived from ions scores as a non-probabilistic basis for ranking protein hits.

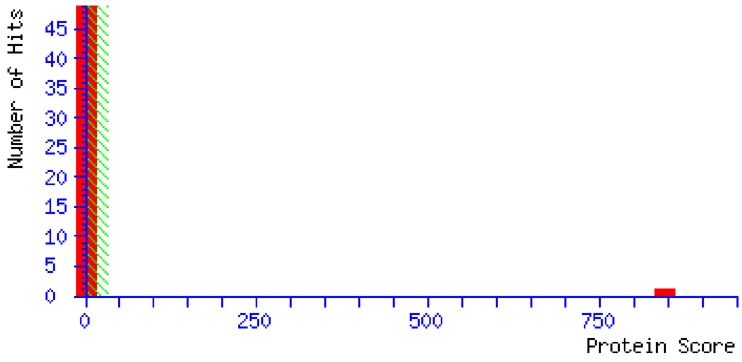

Matched peptide sequences: shown in Bold Red

```
  1 MAICSSTSGT SSSLPSRTTV MLLLFFFTAS VGITDAQVGV CYGMQGNNLP
 51 PVSEVIALYK KSNITRMRIY DPNQAVLEAL RGSNIELILG VPNSDLQSLT
101 NPSNAKSWVQ KNVRGFWSSV RFRYIAVGNE ISPVNRGTAW LAQFVLPAMR
151 NIHDAIRSAG LQDQIKVSTA IDLTLVGNSY PPSAGAFRDD VRSYLNPIIR
201 FLSSIRSPLL ANIYPYFTYA GNPRDISLPY ALFTSPSVVV WDGQRGYKNL
251 FDATLDALYS ALERASGGSL EVVVSESGWP SAGAFAATFD NGRTYLSNLI
301 QHVKRGTPKR PNRAIETYLF AMFDENKKQP EVEKHFGLFF PDKRPKYNLN
351 FGAEKNWDIS TEHNATILFL KSDM
```

Matched peptide information:

Start - End	Observed	Mr(expt)	Mr(calc)	ppm	Miss	Sequence
69 - 81	1501.7177	1500.7104	1500.7936	-55	0	R.IYDPNQAVLEALR.G (Ions score 88)
124 - 136	1431.6791	1430.6718	1430.7518	-56	0	R.YIAVGNEISPVNR.G (Ions score 75)
137 - 150	1560.7493	1559.7420	1559.8282	-55	0	R.GTAWLAQFVLPAMR.N (Ions score 88)
167 - 188	2236.0447	2235.0374	2235.1535	-52	0	K.VSTAIDLTLVGNSYPPSAGAFR.D (Ions score 143)
167 - 192	2721.2346	2720.2273	2720.3770	-55	1	K.VSTAIDLTLVGNSYPPSAGAFRDDVR.S (Ions score 2)
193 - 200	975.4985	974.4912	974.5549	-65	0	R.SYLNPIIR.F (Ions score 41)
207 - 224	2056.9434	2055.9361	2056.0418	-51	0	R.SPLLANIYPYFTYAGNPR.D (Ions score 151)
225 - 245	2350.0876	2349.0804	2349.2005	-51	0	R.DISLPYALFTSPSVVVWDGQR.G (Ions score 197)
249 - 264	1811.8210	1810.8138	1810.9101	-53	0	K.NLFDATLDALYSALER.A (Ions score 127)
265 - 293	2826.1811	2825.1739	2825.3257	-54	0	R.ASGGSLEVVVSESGWPSAGAFAATFDNGR.T (Ions score 177)

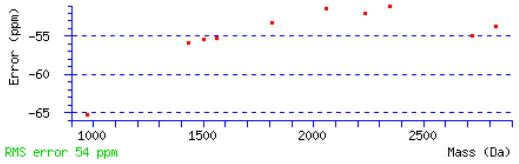

Spot No.:**31**

Accession No.: **scaffold1735_70085.mRNA1**

Protein name：**ricin B-like lectin EULS3**

Peptide sequences:

K.TESESSHCR.K;K.ADPNFFLTIR.D;K.VVLAPPDPSDEFQHWYK.D;K.VVLAPPDPSDEFQHWYKDEK.F;K.FSTMVKDEEGFPSFALVNK.A;K.DEEGFPSFALVNK.A;K.HSFGPTHPVLLTPYDPDDLEASILWTESK.D;K.VVLAYWNK.G

PFF Mascot score: **[494]** Sequence coverage %: **[51]**

Matched peptides No.: **[8]**

Calculated Mr: **20724** Calculated *p*I: **7.77**

Data base searched result:

Ions score is -10*Log(P), where P is the probability that the observed match is a random event.
Individual ions scores > 31 indicate identity or extensive homology (p<0.05).
Protein scores are derived from ions scores as a non-probabilistic basis for ranking protein hits.

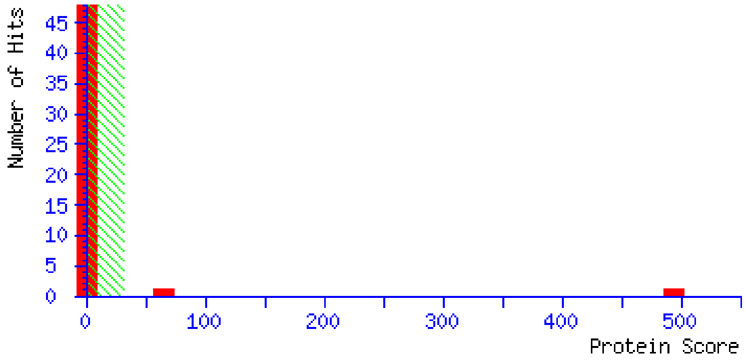

Matched peptide sequences: shown in **Bold Red**

```
  1 MEELGSSHEK TESESSHCRK PHLPSGSYLS KKPSYKVYCK ADPNFFLTIR
 51 DGKVVLAPPD PSDEFQHWYK DEKFSTMVKD EEGFPSFALV NKASGKALKH
101 SFGPTHPVLL TPYDPDDLEA SILWTESKDL GDGYRIVRML NNIRLNVEAL
151 RGDKKSGGVS NGTKVVLAYW NKGDHQKWRI APL
```

Matched peptide information:

Start - End	Observed	Mr(expt)	Mr(calc)	ppm	Miss	Sequence
11 - 19	1092.3724	1091.3652	1091.4302	-60	0	K.TESESSHCR.K (Ions score 47)
41 - 50	1193.5490	1192.5417	1192.6241	-69	0	K.ADPNFFLTIR.D (Ions score 56)
54 - 70	2027.8546	2026.8473	2026.9789	-65	0	K.VVLAPPDPSDEFQHWYK.D (Ions score 94)
54 - 73	2399.9971	2398.9898	2399.1434	-64	1	K.VVLAPPDPSDEFQHWYKDEK.F (Ions score 106)
74 - 92	2145.9106	2144.9034	2145.0452	-66	1	K.FSTMVKDEEGFPSFALVNK.A (Ions score 102)
80 - 92	1452.5966	1451.5893	1451.6933	-72	0	K.DEEGFPSFALVNK.A (Ions score 104)
100 - 128	3265.3818	3264.3746	3264.5979	-68	0	K.HSFGPTHPVLLTPYDPDDLEASILWTESK.D (Ions score 133)
165 - 172	992.4874	991.4801	991.5491	-70	0	K.VVLAYWNK.G (Ions score 54)

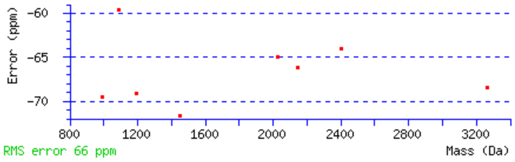

Spot No.:**32**

Accession No.: **scaffold0143_850373.mRNA1**

Protein name： **Hevamine-A**

Peptide sequences:

K.FGNGQTPQINLAGHCNPAAGGCTIVSNGIR.S;K.VMLSLGGGIGSYTLASQADAK.N;K.SSSRPLGDAVLDGIDFDIEHGSTLYWDDLAR.Y;R.PLGDAVLDGIDFDIEHGSTLYWDDLAR.Y;K.KVYLTAAPQCPFPDR.Y;K.VYLTAAPQCPFPDR.Y;K.IFLGLPAAPEAAGSGYVPPDVLISR.I

PFF Mascot score: **[830]** Sequence coverage %: **[39]**

Matched peptides No.: **[7]**

Calculated Mr: **34013** Calculated *p*I: **8.07**

Data base searched result:

Ions score is -10*Log(P), where P is the probability that the observed match is a random event.
Individual ions scores > 30 indicate identity or extensive homology ($p<0.05$).
Protein scores are derived from ions scores as a non-probabilistic basis for ranking protein hits.

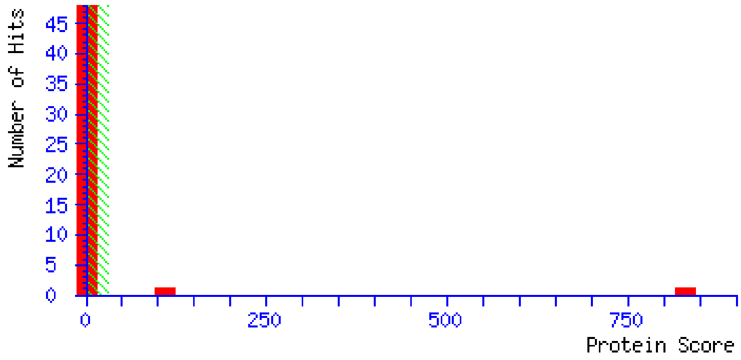

Matched peptide sequences: shown in Bold Red

```
  1 MATRTQAILL LLLAISLIMS SSHVDGGGIA IYWGQNGNEG TLTETCSTGK
 51 YSYVNIAFLN KFGNGQTPQI NLAGHCNPAA GGCTIVSNGI RSCQIQGIKV
101 MLSLGGGIGS YTLASQADAK NVADYLWNNF LGGKSSSRPL GDAVLDGIDF
151 DIEHGSTLYW DDLARYLSAY SKQGKKVYLT AAPQCPFPDR YLGTALNTGL
201 FDYVWVQFYN NPPCQYSSGN INNIINSWNR WTTSINAGKI FLGLPAAPEA
251 AGSGYVPPDV LISRILPEIK KSPKYGGVML WSKFYDDKNG YSSSIRDSVL
301 FLHSEKCMTV L
```

Matched peptide information:

Start - End	Observed	Mr(expt)	Mr(calc)	ppm	Miss	Sequence
62 - 91	3082.2617	3081.2544	3080.4669	256	0	K.FGNGQTPQINLAGHCNPAAGGCTIVSNGIR.S (Ions score 128)
100 - 120	2038.9144	2037.9072	2038.0405	-65	0	K.VMLSLGGGIGSYTLASQADAK.N (Ions score 178)
135 - 165	3420.4080	3419.4007	3419.6270	-66	1	K.SSSRPLGDAVLDGIDFDIEHGSTLYWDDLAR.Y (Ions score 121)
139 - 165	3003.2463	3002.2391	3002.4298	-64	0	R.PLGDAVLDGIDFDIEHGSTLYWDDLAR.Y (Ions score 184)
176 - 190	1762.7904	1761.7831	1761.8872	-59	1	K.KVYLTAAPQCPFPDR.Y (Ions score 111)
177 - 190	1634.7051	1633.6978	1633.7923	-58	0	K.VYLTAAPQCPFPDR.Y (Ions score 91)
240 - 264	2510.2161	2509.2088	2509.3580	-59	0	K.IFLGLPAAPEAAGSGYVPPDVLISR.I (Ions score 184)

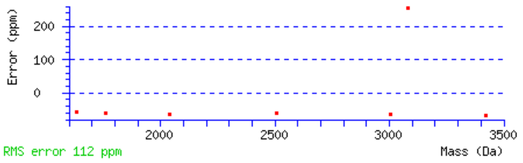

Spot No.:**33**

Accession No.: **scaffold0491_874744.mRNA1**

Protein name: **Elongation factor 1-delta**

Peptide sequences:

K.APSSEYVNVSR.W;R.WYYHIDALLR.I;K.AVAAAAEDDDDDVDLFGEETEEEK.K;K.SSVLLDVKPWDDETDMK.K;R.SIQMEGLLWGASK.L

PFF Mascot score: **[192]** Sequence coverage %: **[31]**

Matched peptides No.: **[5]**

Calculated Mr: **25911** Calculated *p*I: **4.51**

Data base searched result:

Ions score is -10*Log(P), where P is the probability that the observed match is a random event.
Individual ions scores > 31 indicate identity or extensive homology (p<0.05).
Protein scores are derived from ions scores as a non-probabilistic basis for ranking protein hits.

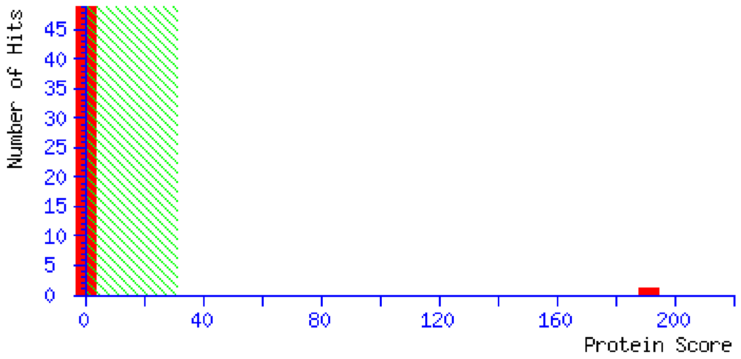

Matched peptide sequences: shown in Bold Red

```
  1 MAVTFYDLGS PAGLKKLDDY LLTHSYITGY QASKDDVTVY AALPKAPSSE
 51 YVNVSRWYYH IDALLRISGV TEEGSGVTVE GSAPITEEVI ATPPANDSKA
101 VAAAAEDDDD DVDLFGEETE EEKKAAEERA AAVKASAKKK ESGKSSVLLD
151 VKPWDDETDM KKLEEAVRSI QMEGLLWGAS KLVPVGYGIK KLQIMLTIVD
201 DLVSVDNLIE EHLTVEPVNE HVQSCDIVAF NKICKQS
```

Matched peptide information:

Start - End	Observed	Mr(expt)	Mr(calc)	ppm	Miss	Sequence
46 - 56	1208.5186	1207.5113	1207.5833	-60	0	K.APSSEYVNVSR.W (Ions score 68)
57 - 66	1349.6156	1348.6083	1348.6928	-63	0	R.WYYHIDALLR.I (Ions score 82)
100 - 123	2582.8994	2581.8921	2582.0667	-68	0	K.AVAAAAAEDDDDDVDLFGEETEEEK.K (Ions score 40)
145 - 161	1977.8140	1976.8067	1976.9401	-67	1	K.SSVLLDVKPWDDETDMK.K (Ions score 74)
169 - 181	1419.6374	1418.6302	1418.7228	-65	0	R.SIQMEGLLWGASK.L (Ions score 48)

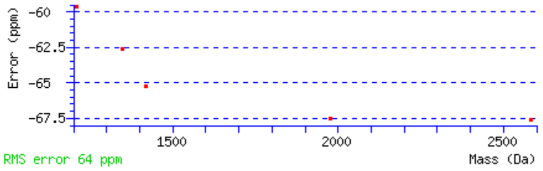

Spot No.:**34**

Accession No.: **scaffold0935_348908.mRNA1**

Protein name：**Proteasome subunit beta type-5**

Peptide sequences:

K.IIEINPYMLGTMAGGAADCQFWHR.N;K.LLANILYSYR.G;R.GMGLSVGTMIAGWDETGPGLYYVDSEGG R.L;R.FSVGSGSPYAYGVLDSGYR.Y;R.YDLSIEEAAELAR.R;R.AIYHATFR.D

PFF Mascot score: **[472]** Sequence coverage %: **[37]**

Matched peptides No.: **[7]**

Calculated Mr: **29355** Calculated *p*I: **5.86**

Data base searched result:

Ions score is -10*Log(P), where P is the probability that the observed match is a random event.
Individual ions scores > 31 indicate identity or extensive homology (p<0.05).
Protein scores are derived from ions scores as a non-probabilistic basis for ranking protein hits.

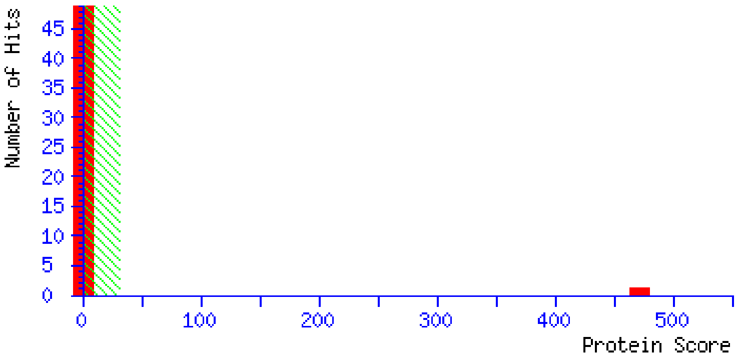

Matched peptide sequences: shown in **Bold Red**

```
  1 MKLDTSGLES AASLFGASSE LFDGFATAPS FELPVTNDFD GFQKEAIQMV
 51 KPAKGTTTLA FIFKEGVMVA ADSRASMGGY ISSQSVKKII EINPYMLGTM
101 AGGAADCQFW HRNLGIKCRL HELANKRRIS VTGASKLLAN ILYSYRGMGL
151 SVGTMIAGWD ETGPGLYYVD SEGGRLKGTR FSVGSGSPYA YGVLDSGYRY
201 DLSIEEAAEL ARRAIYHATF RDGASGGVAS VYYVGPNGWK KLSGDDVGEL
251 HYKYYPVMPS TVEQEMVEVS GA
```

Matched peptide information:

Start	–	End	Observed	Mr(expt)	Mr(calc)	ppm	Miss	Sequence	
89	–	112	2751.1169	2750.1097	2750.2767	-61	0	K.IIEINPYMLGTMAGGAADCQFWHR.N	(Ions score 32)
137	–	146	1225.6282	1224.6209	1224.6866	-54	0	K.LLANILYSYR.G	(Ions score 78)
147	–	175	2975.1653	2974.1580	2974.3477	-64	0	R.GMGLSVGTMIAGWDETGPGLYYVDSEGGR.L	(Ions score 156)
181	–	199	1981.8212	1980.8139	1980.9218	-54	0	R.FSVGSGSPYAYGVLDSGYR.Y	(Ions score 193)
200	–	212	1479.6545	1478.6473	1478.7252	-53	0	R.YDLSIEEAAELAR.R	(Ions score 115)
214	–	221	978.4853	977.4780	977.5083	-31	0	R.AIYHATFR.D	(Ions score 39)

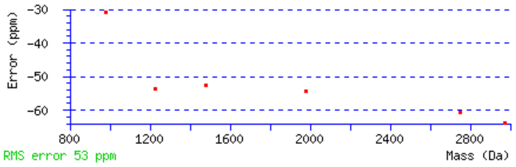

Spot No.:**35**

Accession No.: **scaffold0645_687748.mRNA1**

Protein name：**18.5 kDa class I heat shock protein**

Peptide sequences:

R.TNIFDPFSLDVWDPFHDFPFPSTALSAPR.F;R.FEFANETSAFANTR.I;K.EEVKVEIEEGNVLQISGER.S;K.VEIEEGNVLQISGER.S;R.FRLPENAK.V

PFF Mascot score: **[420]** Sequence coverage %: **[43]**

Matched peptides No.: **[5]**

Calculated Mr: **18335** Calculated *p*I: **6.00**

Data base searched result:

Ions score is -10*Log(P), where P is the probability that the observed match is a random event.
Individual ions scores > 31 indicate identity or extensive homology (p<0.05).
Protein scores are derived from ions scores as a non-probabilistic basis for ranking protein hits.

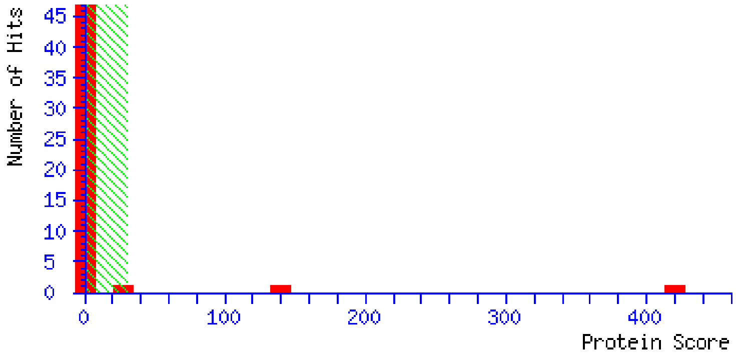

Matched peptide sequences: shown in Bold Red

```
  1 MSLIPSSLFG GRRTNIFDPF SLDVWDPFHD FPFPSTALSA PRFEFANETS
 51 AFANTRIDWK ETPEAHVFKA DLPGLKKEEV KVEIEEGNVL QISGERSKEK
101 EEKNDKLHRV ERSSGKFLRR FRLPENAKVD QVKASMESGV LTVTVPKEEV
151 KKPDVKAIDI SG
```

Matched peptide information:

Start	-	End	Observed	Mr(expt)	Mr(calc)	ppm	Miss	Sequence
14	-	42	3336.3960	3335.3887	3335.5928	-61	0	R.TNIFDPFSLDVWDPFHDFPFPSTALSAPR.F (Ions score 56)
43	-	56	1604.6473	1603.6401	1603.7267	-54	0	R.FEFANETSAFANTR.I (Ions score 128)
78	-	96	2156.9954	2155.9881	2156.0961	-50	1	K.EEVKVEIEEGNVLQISGER.S (Ions score 192)
82	-	96	1671.7681	1670.7608	1670.8475	-52	0	K.VEIEEGNVLQISGER.S (Ions score 117)
121	-	128	974.4775	973.4703	973.5345	-66	1	R.FRLPENAK.V (Ions score 41)

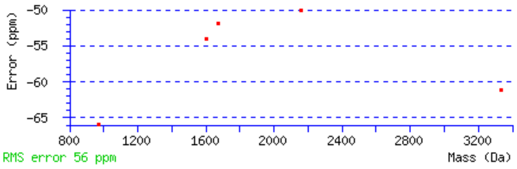

Spot No.:36

Accession No.: **scaffold1194_13551.mRNA1**

Protein name：**(+)-neomenthol dehydrogenase**

Peptide sequences:

K.GIGFEICR.Q;R.QLASNGIVVVLTAR.D;K.TDINLNMGILSVEEGAESPAR.L;K.EVFVDADNLSEER.I;K.EVFVDADNLSEERIDEVLGK.Y;R.LALLPNDGPSGCFFFR.K

PFF Mascot score: **[332]**　　Sequence coverage %: **[10]**

Matched peptides No.: **[6]**

Calculated Mr: **88348**　　Calculated *p*I: **7.08**

Data base searched result:

Ions score is -10*Log(P), where P is the probability that the observed match is a random event.
Individual ions scores > 30 indicate identity or extensive homology (p<0.05).
Protein scores are derived from ions scores as a non-probabilistic basis for ranking protein hits.

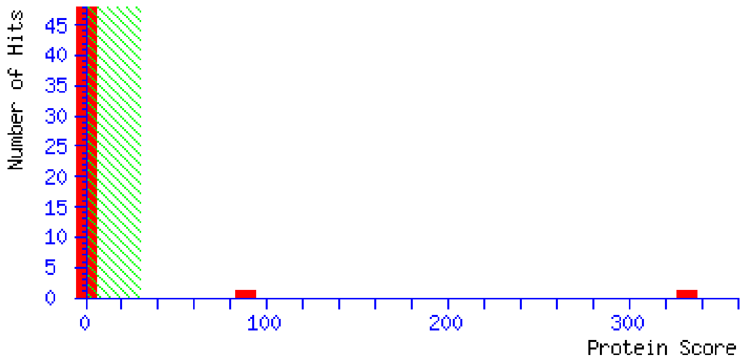

Matched peptide sequences: shown in Bold Red

```
  1 MTEALIPLLQ LSDSPRIVNV SSSMGKLKNV TNEWAKEVFV AADNLSEERI
 51 DEVLSKYLKD YKEGSLESKR WPAFMSAYIL SKAAMNAYTR ILAKKLPNFR
101 INCVCPGFVK TDINLNMGVL SVEEGAESPA RLALLPNDGP SGCFFSLTHK
151 YYLQWHKQPR GNSFLIHIHR QYSYLYLPCP LLRLLLTTSS SSFFLFRYAV
201 VTGANKGIGF EICRQLASNG IVVVLTARDE KRGLEAFQKL KDSGFSDLLV
251 FHQLDVADAA SIAALVDFIK TKFGKLDIMV NNAGVLGIKV DGDAFITEPG
301 KEGASINWHK THQTYELAEE CLTINYYGAK RMVEVLIPLL QLSDSPRIVN
351 VSSSMGKLKN VTNEWAKEVF VAADNLPEER IDEVLSKYLK DYKEGSLESK
401 GWPAFMSAYI LSKAAMNAYT RILAKKLPTF RINCVCPGFV KTDINLNMGI
451 LSVEEGAESP ARLALLPNDG PSGCFFTVIL FYLLLLLLRL LVTNCSFFFF
501 RYAVVTGANK GIGFEICRQL ASNGIVVVLT ARDEKRGLEA FQKLKDSGFS
551 DLLVFHQLDV ADAASIATLA NFIKTQFGKL DILVWNNAGV GGIEVDVDAL
601 KAESSKDSGI NWHKVSTQTY ESAEECLTIN YYGAKRMTEA LIPLLQLSDS
651 PRIVNVSSSM GKLKNVTNEW AKEVFVDADN LSEERIDEVL GKYLKDYKEG
701 SLESKGWPAF MSAYILSKAA MNAYTRILAK KLPTFRINCV CPGFVKTDIN
751 LNMGILSVEE GAESPARLAL LPNDGPSGCF FFRKEESPF
```

Matched peptide information:

Start - End	Observed	Mr(expt)	Mr(calc)	ppm	Miss	Sequence
207 - 214	951.4416	950.4343	950.4644	-32	0	K.GIGFEICR.Q (Ions score 38)
215 - 228	1440.8117	1439.8044	1439.8460	-29	0	R.QLASNGIVVVLTAR.D (Ions score 102)
442 - 462	2216.0049	2214.9976	2215.0790	-37	0	K.TDINLNMGILSVEEGAESPAR.L (Ions score 149)
673 - 685	1522.6564	1521.6491	1521.6947	-30	0	K.EVFVDADNLSEER.I (Ions score 70)
673 - 692	2277.0383	2276.0311	2276.1172	-38	1	K.EVFVDADNLSEERIDEVLGK.Y (Ions score 36)
768 - 783	1810.8383	1809.8310	1809.8872	-31	0	R.LALLPNDGPSGCFFFR.K (Ions score 78)

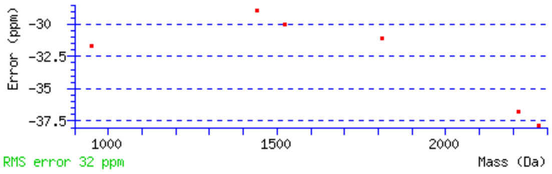

Spot No.:**37**

Accession No.: **scaffold1220_115657.mRNA1**

Protein name： **ATP-citrate synthase beta chain protein 1**

Peptide sequences:

R.MLDFDFLCGR.E;R.VTDGIYEGIAIGGDVFPGSTLSDHVLR.F;K.DLVSSLVSGLLTIGPR.F;R.VELLQLFAR.T

PFF Mascot score: **[263]** Sequence coverage %: **[10]**

Matched peptides No.: **[4]**

Calculated Mr: **66478** Calculated *p*I: **7.95**

Data base searched result:

Ions score is -10*Log(P), where P is the probability that the observed match is a random event.
Individual ions scores > 31 indicate identity or extensive homology (p<0.05).
Protein scores are derived from ions scores as a non-probabilistic basis for ranking protein hits.

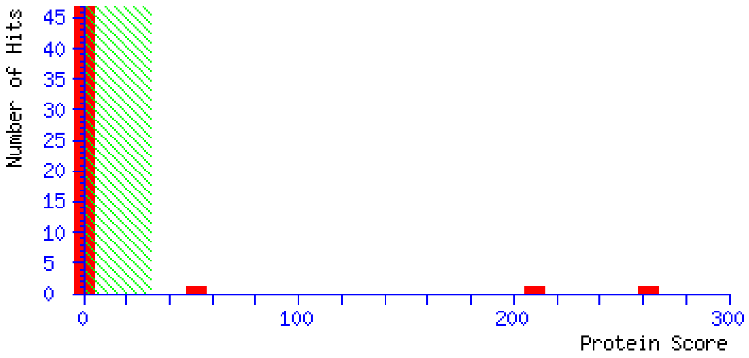

Matched peptide sequences: shown in Bold Red

1 MATGQLFSRT TQALFYNYKQ LPIQR**MLDFD** **FLCGR**ETPSV AGIINPGAEG
51 FQKLFFGQEE IAIPVHSTIE VACAAHPTAD VFINFASFRS AAVSSMAALK
101 QPTIRVVAII AEGVPESDTK QLIAYARSNN KVVIGPATVG GIQAGAFKIG
151 DTAGTIDNII ACKLYRPGSV GFVSKSGGMS NELYNTIAR**V** **TDGIYEGIAI**
201 **GGDVFPGSTL** **SDHVLR**FNNI PQVKMMVVLG ELGGRDEYSL VEALKQGKVT
251 KPVVAWVSGT CARLFKSEVQ FGHAGAKSGG EMESAQAKNQ ALKDAGAVVP
301 TSYEAFETAI KETFQKLVEE GKITPVKEIK PPQIPEDLNT AIKSGKVRAP
351 THIISTISDD RGEEPCYAGV PMSSIVEQGY GVGDVISLLW FKRSLPRYCT
401 HFIEICIMLC ADHGPCVSGA HNTIVTARAG K**DLVSSLVSG** **LLTIGPR**FGG
451 AIDDAARYFK DAYDRGLTPY EFVESMKKKG IRVPGIGHRI KRGDNRDKR**V**
501 **ELLQLFAR**TH FPSVKYMEYA VQVETYTLSK ANNLVLNVDG AIGSLFLDLL
551 AGSGMFTKPE IDEIVEIGYL NGLFVLARSI GLIGHTFDQK RLKQPLYRHP
601 WEDVLYTK

Matched peptide information:

Start - End	Observed	Mr(expt)	Mr(calc)	ppm	Miss	Sequence
26 - 35	1273.5244	1272.5171	1272.5631	-36	0	R.MLDFDFLCGR.E (Ions score 74)
190 - 216	2788.3086	2787.3013	2787.4080	-38	0	R.VTDGIYEGIAIGGDVFPGSTLSDHVLR.F (Ions score 111)
432 - 447	1626.8898	1625.8825	1625.9352	-32	0	K.DLVSSLVSGLLTIGPR.F (Ions score 111)
500 - 508	1088.6080	1087.6008	1087.6390	-35	0	R.VELLQLFAR.T (Ions score 55)

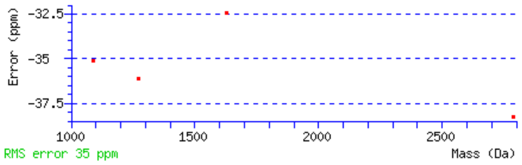

Spot No.:**38**

Accession No.: **scaffold1222_136753.mRNA1**

Protein name： **Rubber elongation factor protein**

Peptide sequences: K.NVAVPLYNR.F;K.FVDSTVVASVTIIDR.S;K.DASIQVVSAIR.A

PFF Mascot score: **[181]** Sequence coverage %: **[25]**

Matched peptides No.: **[3]**

Calculated Mr: **14713** Calculated *p*I: **5.04**

Data base searched result:

Ions score is -10*Log(P), where P is the probability that the observed match is a random event.
Individual ions scores > 31 indicate identity or extensive homology (p<0.05).
Protein scores are derived from ions scores as a non-probabilistic basis for ranking protein hits.

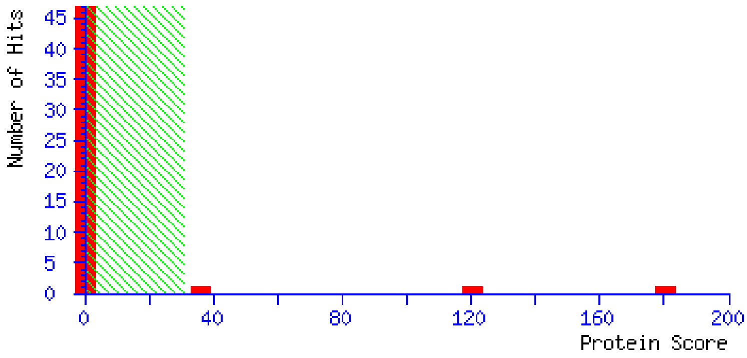

Matched peptide sequences: shown in Bold Red

```
  1 MAEDEDNQQG QGEGLKYLGF VQDAATYAVT TFSNVYLFAK DKSGPLQPGV
 51 DIIEGPVKNV AVPLYNRFSY IPNGALKFVD STVVASVTII DRSLPPIVKD
101 ASIQVVSAIR AAPEAARSLA SSLPGQTKIL AKVFYGEN
```

Matched peptide information:

Start - End	Observed	Mr(expt)	Mr(calc)	ppm	Miss	Sequence
59 - 67	1045.5503	1044.5430	1044.5716	-27	0	K.NVAVPLYNR.F (Ions score 61)
78 - 92	1621.8475	1620.8403	1620.8723	-20	0	K.FVDSTVVASVTIIDR.S (Ions score 128)
100 - 110	1158.6215	1157.6142	1157.6404	-23	0	K.DASIQVVSAIR.A (Ions score 54)

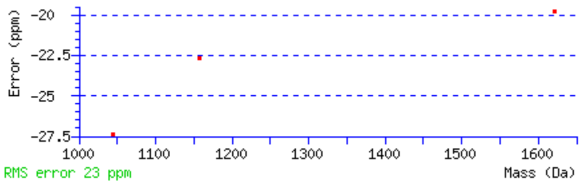

Spot No.:**39**

Accession No.: **scaffold0686_576053.mRNA1**

Protein name：**Pyridoxal biosynthesis protein PDX1**

Peptide sequences:

R.GGVIMDVVNPEQAR.I;R.IAEEAGACAVMALER.V;R.IGHFVEAQILEAIGIDYVDESEVLTPADEENHINK.H;R.IPFVCGCR.N;K.GEAGTGNVIEAVR.H

PFF Mascot score: **[162]** Sequence coverage %: **[27]**

Matched peptides No.: **[5]**

Calculated Mr: **33397** Calculated *p*I: **6.26**

Data base searched result:

Ions score is -10*Log(P), where P is the probability that the observed match is a random event.
Individual ions scores > 31 indicate identity or extensive homology (p<0.05).
Protein scores are derived from ions scores as a non-probabilistic basis for ranking protein hits.

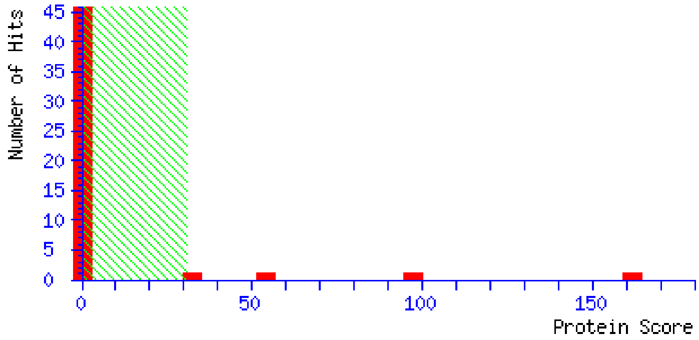

Matched peptide sequences: shown in Bold Red

```
  1 MAGTGVVAVY GNGAITETKK SPFSVKVGLA QMLRGGVIMD VVNPEQARIA
 51 EEAGACAVMA LERVPADIRA QGGVARMSDP QLIKEIKQSV TIPVMAKARI
101 GHFVEAQILE AIGIDYVDES EVLTPADEEN HINKHNFRIP FVCGCRNLGE
151 ALRRIREGAA MIRTKGEAGT GNVIEAVRHV RSVMGDIRLL RNMDDDEVFT
201 FAKKIAAPYD LVMQTKQLGR LPVVQFAAGG VATPADAALM MQLGCDGVFV
251 GSGVFKSGDP ARRARAIVQA VTHYSDPDML AEVSCGLGEA MVGINLNDKK
301 VERFANRSE
```

Matched peptide information:

Start - End	Observed	Mr(expt)	Mr(calc)	ppm	Miss	Sequence
35 - 48	1484.7329	1483.7256	1483.7453	-13	0	R.GGVIMDVVNPEQAR.I (Ions score 68)
49 - 63	1590.7413	1589.7341	1589.7541	-13	0	R.IAEEAGACAVMALER.V (Ions score 87)
100 - 134	3907.8130	3906.8057	3906.9163	-28	0	R.IGHFVEAQILEAIGIDYVDESEVLTPADEENHINK.H (Ions score 40)
139 - 146	1008.4561	1007.4488	1007.4681	-19	0	R.IPFVCGCR.N (Ions score 51)
166 - 178	1272.6345	1271.6272	1271.6470	-16	0	K.GEAGTGNVIEAVR.H (Ions score 27)

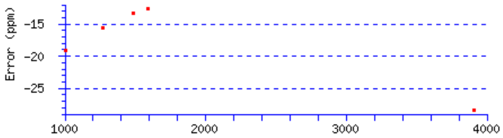

RMS error 18 ppm

Spot No.:**40**

Accession No.: **scaffold0166_1819893.mRNA1**

Protein name：**Uncharacterized protein**

Peptide sequences: K.LSDEEPDVDYR.I;R.TFQLVAGGKPGALIR.P

PFF Mascot score: **[129]** Sequence coverage %: **[22]**

Matched peptides No.: **[2]**

Calculated Mr: **12855** Calculated *p*I: **5.37**

Data base searched result:

Ions score is -10*Log(P), where P is the probability that the observed match is a random event.
Individual ions scores > 31 indicate identity or extensive homology (p<0.05).
Protein scores are derived from ions scores as a non-probabilistic basis for ranking protein hits.

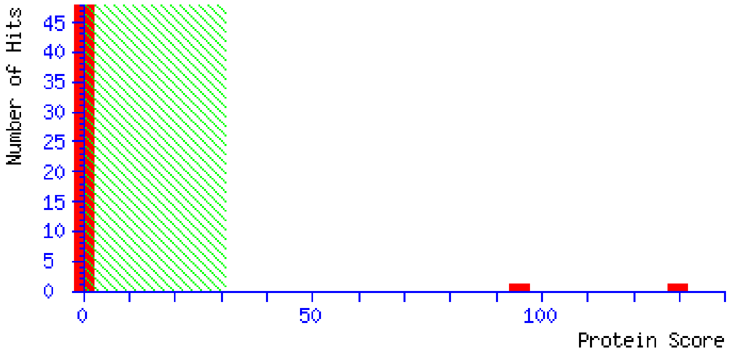

Matched peptide sequences: shown in Bold Red

```
  1 MSNTSDYTSK ESLTNLFKEI SVDEQEQEIA ILSILLEGIS VEKLSDEEPD
 51 VDYRIRSFSE LFDKLPSDSI YPTAFFNTHH IPTKWLRAGG RVRTFQLVAG
101 GKPGALIRPK QSKS
```

Matched peptide information:

Start - End	Observed	Mr(expt)	Mr(calc)	ppm	Miss	Sequence	
44 - 54	1337.5874	1336.5801	1336.5783	1	0	K.LSDEEPDVDYR.I	(Ions score 68)
94 - 108	1527.9012	1526.8940	1526.8933	0	1	R.TFQLVAGGKPGALIR.P	(Ions score 93)

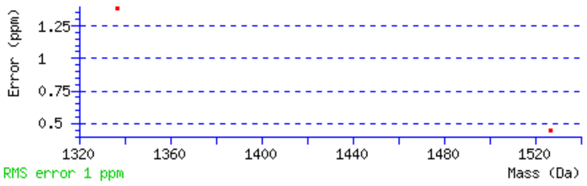

Spot No.:**41**

Accession No.: **scaffold1222_136753.mRNA1**

Protein name：**Rubber elongation factor protein**

Peptide sequences:

K.YLGFVQDAATYAVTTFSNVYLFAK.D;K.DKSGPLQPGVDIIEGPVK.N;K.NVAVPLYNR.F;R.FSYIPNGALK.F;K.FVDSTVVASVTIIDR.S;K.DASIQVVSAIR.A

PFF Mascot score: **[294]** Sequence coverage %: **[63]**

Matched peptides No.: **[6]**

Calculated Mr: **14713** Calculated *p*I: **5.04**

Data base searched result:

Ions score is -10*Log(P), where P is the probability that the observed match is a random event.
Individual ions scores > 31 indicate identity or extensive homology (p<0.05).
Protein scores are derived from ions scores as a non-probabilistic basis for ranking protein hits.

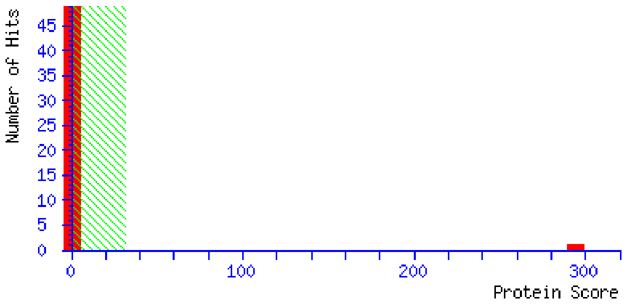

Matched peptide sequences: shown in Bold Red

```
  1 MAEDEDNQQG QGEGLKYLGF VQDAATYAVT TFSNVYLFAK DKSGPLQPGV
 51 DIIEGPVKNV AVPLYNRFSY IPNGALKFVD STVVASVTII DRSLPPIVKD
101 ASIQVVSAIR AAPEAARSLA SSLPGQTKIL AKVFYGEN
```

Matched peptide information:

Start - End	Observed	Mr(expt)	Mr(calc)	ppm	Miss	Sequence
17 - 40	2689.3347	2688.3274	2688.3476	-7	0	K.YLGFVQDAATYAVTTFSNVYLFAK.D (Ions score 110)
41 - 58	1849.0012	1847.9939	1847.9993	-3	1	K.DKSGPLQPGVDIIEGPVK.N (Ions score 47)
59 - 67	1045.5614	1044.5541	1044.5716	-17	0	K.NVAVPLYNR.F (Ions score 55)
68 - 77	1109.5828	1108.5755	1108.5917	-15	0	R.FSYIPNGALK.F (Ions score 51)
78 - 92	1621.8658	1620.8586	1620.8723	-8	0	K.FVDSTVVASVTIIDR.S (Ions score 110)
100 - 110	1158.6356	1157.6283	1157.6404	-10	0	K.DASIQVVSAIR.A (Ions score 66)

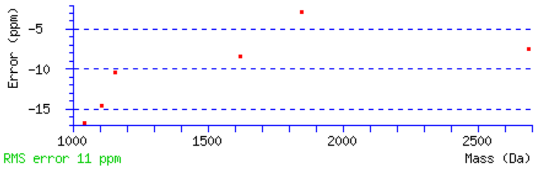

Spot No.:**42**

Accession No.: **scaffold0878_145544.mRNA1**

Protein name: **Importin subunit alpha-1b**

Peptide sequences:

R.SPPIEEVIQAGVVPR.F;R.FVEFLMR.E;R.EDFPQLQFEAAWALTNIASGTSENTR.V;K.IQAVIEAGVCPR.L

PFF Mascot score: **[123]** Sequence coverage %: **[11]**

Matched peptides No.: **[4]**

Calculated Mr: **59161** Calculated *p*I: **5.28**

Data base searched result:

Ions score is -10*Log(P), where P is the probability that the observed match is a random event.
Individual ions scores > 31 indicate identity or extensive homology (p<0.05).
Protein scores are derived from ions scores as a non-probabilistic basis for ranking protein hits.

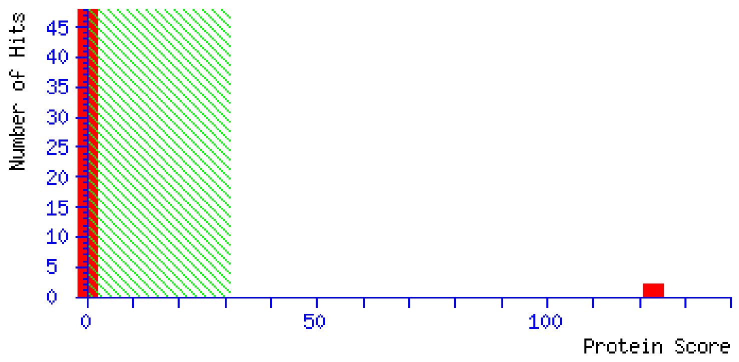

Matched peptide sequences: shown in Bold Red

```
  1 MSLRPSARTE VRRNRYKVAV DAEESRRRRE DNMVEIRKNR REESLQKKRR
 51 EGLQTQPMPA AIHSSAVEKR LEHLPSMVAG VWSDDSNLLL EATTQFRKLL
101 SIERSPPIEE VIQAGVVPRF VEFLMREDFP QLQFEAAWAL TNIASGTSEN
151 TRVVIDHGAV PIFVKLLGSP SDDVREQAVW ALGNVAGDSP KCRDLVLGHG
201 ALIPLLAQLN EHAKLSMLRN ATWTLSNFCR GKPQPPFDQV KPALPALAHL
251 IHSNDEEVLT DACWALSYLS DGTNDKIQAV IEAGVCPRLV ELLLHPSPSV
301 LIPALRTVGN IVTGDDMQTQ CIINHQALPC LLNLLTNNYK KSIKKEACWT
351 ISNITAGNKE QIQAVIEANI IAPLVHLLQN AEFDIKKEAA WAISNATSGG
401 THDQIKYLVS QGCIKPLCDL LICPDPRIVT VCLEGLENIL KVGEADKNLG
451 STGGVNQYAQ MIDDAEGLEK IENLQSHDNT EIYEKAVKIL ETYWLEEEDE
501 TMPPGDASQS GFQFGGSEMP SVPSGGFNFS
```

Matched peptide information:

Start - End	Observed	Mr(expt)	Mr(calc)	ppm	Miss	Sequence
105 - 119	1590.8744	1589.8671	1589.8777	-7	0	R.SPPIEEVIQAGVVPR.F (Ions score 113)
120 - 126	941.4836	940.4763	940.4841	-8	0	R.FVEFLMR.E (Ions score 16)
127 - 152	2896.3347	2895.3274	2895.3675	-14	0	R.EDFPQLQFEAAWALTNIASGTSENTR.V (Ions score 38)
277 - 288	1312.7009	1311.6937	1311.6969	-2	0	K.IQAVIEAGVCPR.L (Ions score 25)

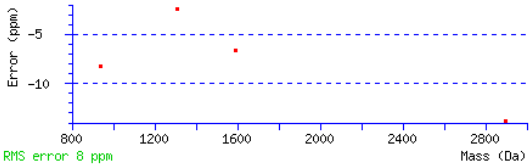

Spot No.:43

Accession No.: scaffold0026_1110574.mRNA1

Protein name： Actin-7

Peptide sequences:

K.AGFAGDDAPR.A;R.AVFPSIVGR.P;K.IWHHTFYNELR.V;R.VAPEEHPVLLTEAPLNPK.A;R.TTGIVLDSGDGVSHTVPIYEGYALPHAILR.L;R.GYMFTTTAER.E;K.NYELPDGQVITIGAER.F;K.DLYGNIVLSGGSTMFPGIADR.M;K.GEYDESGPSIVHR.K

PFF Mascot score: [521] Sequence coverage %: [36]

Matched peptides No.: [9]

Calculated Mr: 41897 Calculated *p*I: 5.31

Data base searched result:

Ions score is -10*Log(P), where P is the probability that the observed match is a random event.
Individual ions scores > 31 indicate identity or extensive homology (p<0.05).
Protein scores are derived from ions scores as a non-probabilistic basis for ranking protein hits.

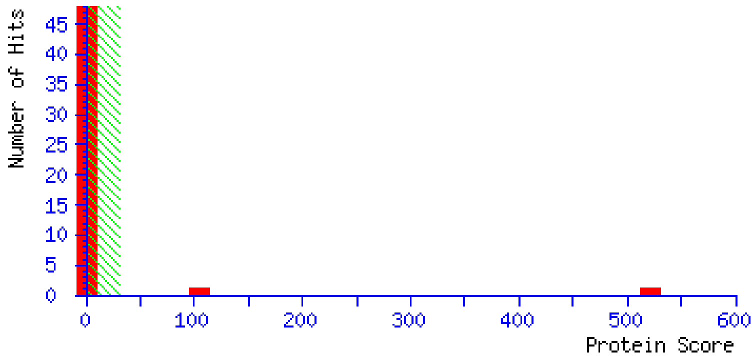

Matched peptide sequences: shown in Bold Red

1 MADAEDIQPL VCDNGTGMVK **AGFAGDDAPR AVFPSIVGR**P RHTGVMVGMG
51 QKDAYVGDEA QSKRGILTLK YPIEHGIVSN WDDMEK**IWHH TFYNELRVAP**
101 **EEHPVLLTEA PLNPK**ANREK MTQIMFETFN VPAMYVAIQA VLSLYASGR**T**
151 **TGIVLDSGDG VSHTVPIYEG YALPHAILRL** DLAGRDLTDA LMKILTER**GY**
201 **MFTTTAER**EI VRDMKEKLAY VALDYEQELE TAKSSSSVEK **NYELPDGQVI**
251 **TIGAER**FRCP EVLFQPSLIG MEAAGIHETT YNSIMKCDVD IRK**DLYGNIV**
301 **LSGGSTMFPG IADR**MSKEIT ALAPSSMKIK VVAPPERKYS VWIGGSILAS
351 LSTFQQMWIS K**GEYDESGPS IVHR**KCF

Matched peptide information:

Start - End	Observed	Mr(expt)	Mr(calc)	ppm	Miss	Sequence
21 - 30	976.4400	975.4327	975.4410	-8	0	K.AGFAGDDAPR.A (Ions score 29)
31 - 39	945.5394	944.5322	944.5444	-13	0	R.AVFPSIVGR.P (Ions score 39)
87 - 97	1515.7417	1514.7344	1514.7419	-5	0	K.IWHHTFYNELR.V (Ions score 70)
98 - 115	1954.0541	1953.0468	1953.0571	-5	0	R.VAPEEHPVLLTEAPLNPK.A (Ions score 102)
150 - 179	3151.6201	3150.6128	3150.6350	-7	0	R.TTGIVLDSGDGVSHTVPIYEGYALPHAILR.L (Ions score 99)
199 - 208	1176.5269	1175.5196	1175.5281	-7	0	R.GYMFTTTAER.E (Ions score 52)
241 - 256	1774.8904	1773.8831	1773.8897	-4	0	K.NYELPDGQVITIGAER.F (Ions score 145)
294 - 314	2183.0730	2182.0657	2182.0729	-3	0	K.DLYGNIVLSGGSTMFPGIADR.M (Ions score 114)
362 - 374	1445.6577	1444.6504	1444.6583	-5	0	K.GEYDESGPSIVHR.K (Ions score 91)

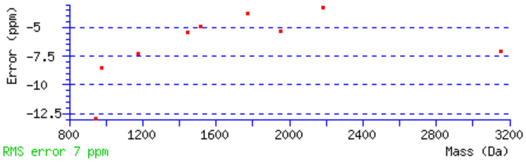

Spot No.:**44**

Accession No.: **scaffold0625_1132.mRNA1**

Protein name：**Glucan endo-1,3-beta-glucosidase, basic vacuolar isoform**

Peptide sequences:

R.IYDPNQAVLEALR.G;K.VSTAIDLTLVGNSYPPSAGAFR.D;R.SYLDPIIGFLSSIR.S;R.SPLLANIYPYFTYADNPR.D;R.DISLPYALFTSPSVVVWDGQR.G;K.NLFDATLDALYSALER.A;R.ASGGSLEVVVSESGWPSAGAFAATFDNGR.T;K.HFGLFFPDK.R

PFF Mascot score: **[764]** Sequence coverage %: **[37]**

Matched peptides No.: **[8]**

Calculated Mr: **41184** Calculated *p*I: **7.77**

Data base searched result:

Ions score is -10*Log(P), where P is the probability that the observed match is a random event.
Individual ions scores > 30 indicate identity or extensive homology (p<0.05).
Protein scores are derived from ions scores as a non-probabilistic basis for ranking protein hits.

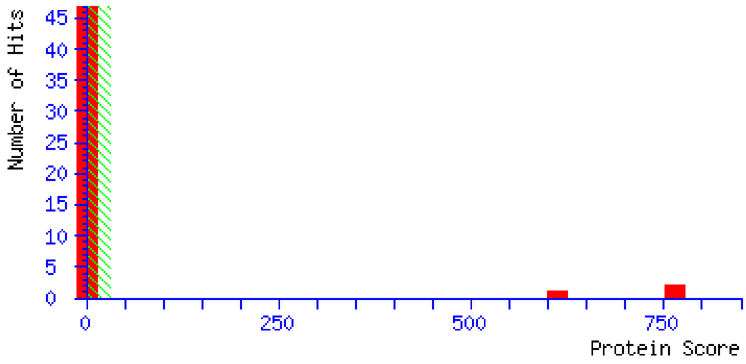

Matched peptide sequences: shown in Bold Red

```
  1 MAISSSTSGT SSSLPSRTTV MLLLIFFTAS LGITDAQVGV CYGMQGNNLP
 51 SVSEVIALYK QSNIKRMRIY DPNQAVLEAL RGSNIELILG VPNSDLQSLT
101 NPSNANSWVQ KNVRGFWSSV RFRYIAVGNE ISPVNGGTAW LAQFVLPAMR
151 NIHDAIRSAG LQDQIKVSTA IDLTLVGNSY PPSAGAFRDD VRSYLDPIIG
201 FLSSIRSPLL ANIYPYFTYA DNPRDISLPY ALFTSPSVVV WDGQRGYKNL
251 FDATLDALYS ALERASGGSL EVVVSESGWP SAGAFAATFD NGRTYLSNLI
301 QHVKGGTPKR PNRAIETYLF AMFDENKKQP EVEKHFGLFF PDKRPKYNLN
351 FGAEKNWDIS TEHNATILFL KSDM
```

Matched peptide information:

Start	-	End	Observed	Mr(expt)	Mr(calc)	ppm	Miss	Sequence
69	-	81	1501.8020	1500.7947	1500.7936	1	0	R.IYDPNQAVLEALR.G (Ions score 117)
167	-	188	2236.1694	2235.1622	2235.1535	4	0	K.VSTAIDLTLVGNSYPPSAGAFR.D (Ions score 172)
193	-	206	1580.8711	1579.8638	1579.8610	2	0	R.SYLDPIIGFLSSIR.S (Ions score 112)
207	-	224	2115.0630	2114.0557	2114.0473	4	0	R.SPLLANIYPYFTYADNPR.D (Ions score 55)
225	-	245	2350.2156	2349.2083	2349.2005	3	0	R.DISLPYALFTSPSVVVWDGQR.G (Ions score 183)
249	-	264	1811.9249	1810.9177	1810.9101	4	0	K.NLFDATLDALYSALER.A (Ions score 147)
265	-	293	2826.3330	2825.3257	2825.3257	0	0	R.ASGGSLEVVVSESGWPSAGAFAATFDNGR.T (Ions score 138)
335	-	343	1107.5513	1106.5440	1106.5549	-10	0	K.HFGLFFPDK.R (Ions score 51)

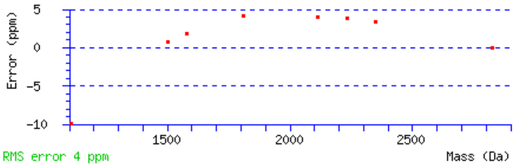

Spot No.:**45**

Accession No.: **scaffold1465_23608.mRNA1**

Protein name：

5-methyltetrahydropteroyltriglutamate--homocysteinemethyltransferase

Peptide sequences:

K.YIPSNTFSYYDQVLDTTAMLGAVPPR.Y;K.KLDLPILPTTTIGSFPQTIELR.R; K.LQEELDIDVLVHGEPER.N;K.GMLTGPVTILNWSFVR.N;K.AGINVIQIDEAALR.E;K.SEQAFYLDWAVHSFR.I;K.YGAGIGPGVYDIHSPR.I

PFF Mascot score: **[558]** Sequence coverage %: **[16]**

Matched peptides No.: **[7]**

Calculated Mr: **85146** Calculated *p*I: **6.30**

Data base searched result:

Ions score is -10*Log(P), where P is the probability that the observed match is a random event.
Individual ions scores > 30 indicate identity or extensive homology (p<0.05).
Protein scores are derived from ions scores as a non-probabilistic basis for ranking protein hits.

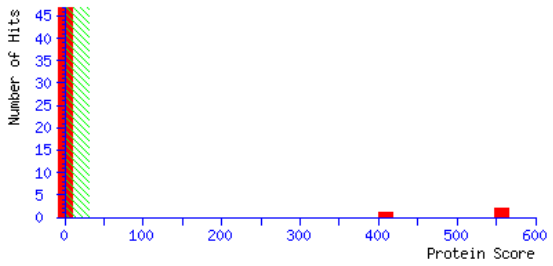

Matched peptide sequences: shown in Bold Red

```
  1 MASHIVGYPR MGPKRELKFA LESFWDKKSS AEDLEKVAAD LRTSIWKQMA
 51 GAGIKYIPSN TFSYYDQVLD TTAMLGAVPP RYGWSGGEIG FDVYFSMARG
101 NASVPAMEMT KWFDTNYHFI VPELGPDVQF SYASHKAVTE YKEAKALGVD
151 SVPVLIGPVS YLLLSKPAKG VEKTFSLISL LGKILPVYKE VISELKAAGA
201 SWIQFDEPTL VMDLDSHKLQ AFTDAYSELE STLSGLNVLI ETYFADIPAK
251 AFKTLTSLKG VSAYGFDLVR GTKTLDLIKS EFPKGKYLFA GVVDGRNIWA
301 NDLASSLSTL HELEGIVGKD KLVVSTSCSL LHTAVDLVNE TKLDKEIKSW
351 LAFAAQKVVE VNALAKALAG EKDEAFFSAN AAAQASRKSS PRVTNEAVQK
401 AAAALRGSDH RRATNVTARL DAQQKKLDLP ILPTTTIGSF PQTIELRRVR
451 REYKANKISE DDYIKAIKEE IQKVVKLQEE LDIDVLVHGE PERNDMVEYF
501 GEQLSGFAFT VNGWVQSYGS RCVKPPIIYG DVSRPKPMTV FWSSTAQSMT
551 ARPMKGMLTG PVTILNWSFV RNDQPRFETC YQIALAIKDE VEDLEKAGIN
601 VIQIDEAALR EGLPLRKSEQ AFYLDWAVHS FRITNCGVQD TTQIHTHMCY
651 SNFNDIIHSI IDMDADVITI ENSRSDEKLL SVFREGVKYG AGIGPGVYDI
701 HSPRIPSTEE IADRINKMLA VLEKNILWVN PDCGLKTRKY SEVKPALNNM
751 VAAAKLLRTQ LASAK
```

Matched peptide information:

Start - End	Observed	Mr(expt)	Mr(calc)	ppm	Miss	Sequence	
56 - 81	2919.3826	2918.3753	2918.4160	-14	0	K.YIPSNTFSYYDQVLDTTAMLGAVPPR.Y	(Ions score 101)
426 - 447	2453.3731	2452.3658	2452.3941	-12	1	K.KLDLPILPTTTIGSFPQTIELR.R	(Ions score 120)
477 - 493	1990.9906	1989.9833	1990.0007	-9	0	K.LQEELDIDVLVHGEPER.N	(Ions score 119)
556 - 571	1790.9436	1789.9363	1789.9549	-10	0	K.GMLTGPVTILNWSFVR.N	(Ions score 75)
597 - 610	1482.8060	1481.7988	1481.8202	-14	0	K.AGINVIQIDEAALR.E	(Ions score 88)
618 - 632	1855.8562	1854.8489	1854.8689	-11	0	K.SEQAFYLDWAVHSFR.I	(Ions score 107)
689 - 704	1658.8124	1657.8051	1657.8213	-10	0	K.YGAGIGPGVYDIHSPR.I	(Ions score 128)

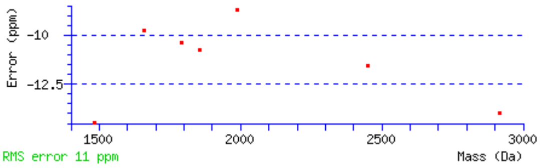

Spot No.: **46**

Accession No.: **scaffold1222_136753.mRNA1**

Protein name：**Rubber elongation factor protein**

Peptide sequences:

K.YLGFVQDAATYAVTTFSNVYLFAK.D;K.DKSGPLQPGVDIIEGPVK.N;K.SGPLQPGVDIIEGPVKNVAVPLYNR.F;K.NVAVPLYNR.F;K.FVDSTVVASVTIIDR.S;K.DASIQVVSAIR.A

PFF Mascot score: **[449]**   Sequence coverage %: **[55]**

Matched peptides No.: **[6]**

Calculated Mr: **14713**   Calculated *p*I: **5.04**

Data base searched result:

Ions score is -10*Log(P), where P is the probability that the observed match is a random event.
Individual ions scores > 31 indicate identity or extensive homology (p<0.05).
Protein scores are derived from ions scores as a non-probabilistic basis for ranking protein hits.

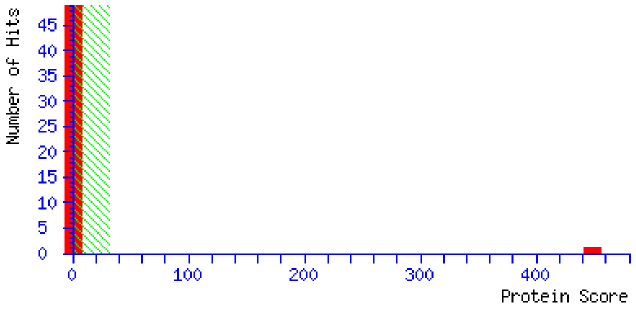

Matched peptide sequences: shown in Bold Red

```
  1 MAEDEDNQQG QGEGLKYLGF VQDAATYAVT TFSNVYLFAK DKSGPLQPGV
 51 DIIEGPVKNV AVPLYNRFSY IPNGALKFVD STVVASVTII DRSLPPIVKD
101 ASIQVVSAIR AAPEAARSLA SSLPGQTKIL AKVFYGEN
```

Matched peptide information:

Start - End	Observed	Mr(expt)	Mr(calc)	ppm	Miss	Sequence	
17 - 40	2689.3562	2688.3489	2688.3476	1	0	K.YLGFVQDAATYAVTTFSNVYLFAK.D	(Ions score 126)
41 - 58	1849.0000	1847.9927	1847.9993	-4	1	K.DKSGPLQPGVDIIEGPVK.N	(Ions score 86)
43 - 67	2632.4368	2631.4295	2631.4384	-3	1	K.SGPLQPGVDIIEGPVKNVAVPLYNR.F	(Ions score 72)
59 - 67	1045.5651	1044.5578	1044.5716	-13	0	K.NVAVPLYNR.F	(Ions score 65)
78 - 92	1621.8757	1620.8685	1620.8723	-2	0	K.FVDSTVVASVTIIDR.S	(Ions score 137)
100 - 110	1158.6361	1157.6288	1157.6404	-10	0	K.DASIQVVSAIR.A	(Ions score 104)

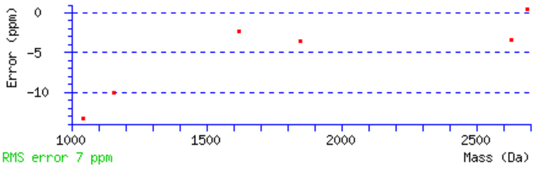

Spot No.:47

Accession No.: scaffold0014_3373206.mRNA1

Protein name：UDP-glucose 6-dehydrogenase 1

Peptide sequences:

K.CPSIEVAVVDISVSR.I;K.FQILSNPEFLAEGTAISDLLSPDR.V;K.DVYAQWVPEDR.I;R.LSIYDPQVTDDQIQR.D

PFF Mascot score: [256] Sequence coverage %: [13]

Matched peptides No.: [4]

Calculated Mr: 53491 Calculated *p*I: 5.90

Data base searched result:

Ions score is -10*Log(P), where P is the probability that the observed match is a random event.
Individual ions scores > 31 indicate identity or extensive homology ($p<0.05$).
Protein scores are derived from ions scores as a non-probabilistic basis for ranking protein hits.

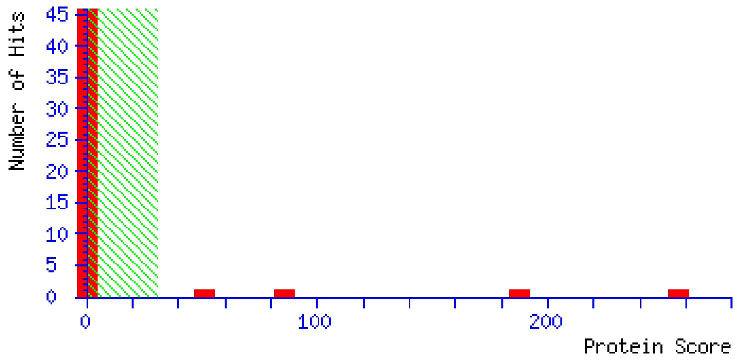

Matched peptide sequences: shown in Bold Red

1 MVKICCIGAG YVGGPTMAVI ALK**CPSIEVA VVDISVSR**IN AWNSEQLPIY
51 EPGLDGVVKE CRGRNLFFST EVEKHVSEAD IVFVSVNTPT KTRGLGAGKA
101 ADLTYWESAA RMIADVSKSN KIVVEKSTVP VKTAEAIEKI LTHNSKGIK**F**
151 **QILSNPEFLA EGTAISDLLS PDR**VLIGGRE TPEGQAAIEA LK**DVYAQWVP**
201 **EDR**ILTTNLW SAELSKLAAN AFLAQRISSV NAMSALCEAT GADVTQVSYA
251 VGKDTRIGPK FLNASVGFGG SCFQKDILNL VYICECNGLP EVAEYWKQVI
301 KINDYQKNRF VNRVVSSMFN TVSNKKIAIL GFAFKKDTGD TRETPAIDVC
351 KGLLGDKAR**L SIYDPQVTDD QIQR**DLTMKK FDWDHPLHLQ PTSPTTVKQV
401 TVVWDAYEAT KDAHGLCFLT EWDEFKTLDY KRIYDNMQKP AFVFDGRNVV
451 NVDKLREIGF IVYSIGKPLD AWLKDMPAVA

Matched peptide information:

Start - End	Observed	Mr(expt)	Mr(calc)	ppm	Miss	Sequence
24 - 38	1630.8463	1629.8390	1629.8396	-0	0	K.CPSIEVAVVDISVSR.I (Ions score 103)
150 - 173	2633.3355	2632.3282	2632.3384	-4	0	K.FQILSNPEFLAEGTAISDLLSPDR.V (Ions score 112)
193 - 203	1377.6400	1376.6327	1376.6361	-2	0	K.DVYAQWVPEDR.I (Ions score 56)
360 - 374	1790.8898	1789.8825	1789.8846	-1	0	R.LSIYDPQVTDDQIQR.D (Ions score 75)

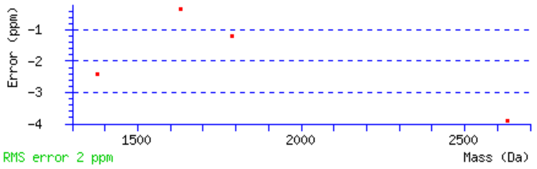

Spot No.:**48**

Accession No.: **scaffold1222_136753.mRNA1**

Protein name： **Rubber elongation factor protein**

Peptide sequences:

K.YLGFVQDAATYAVTTFSNVYLFAK.D;K.NVAVPLYNR.F;R.FSYIPNGALK.F;K.FVDSTVVASVTIIDR.S;K.DASIQVVSAIR.A

PFF Mascot score: **[411]** Sequence coverage %: **[50]**

Matched peptides No.: **[5]**

Calculated Mr: **14713** Calculated *p*I: **5.04**

Data base searched result:

Ions score is -10*Log(P), where P is the probability that the observed match is a random event.
Individual ions scores > 31 indicate identity or extensive homology (p<0.05).
Protein scores are derived from ions scores as a non-probabilistic basis for ranking protein hits.

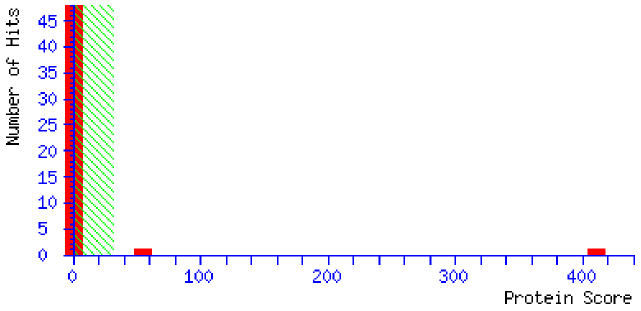

Matched peptide sequences: shown in Bold Red

```
  1 MAEDEDNQQG QGEGLKYLGF VQDAATYAVT TFSNVYLFAK DKSGPLQPGV
 51 DIIEGPVKNV AVPLYNRFSY IPNGALKFVD STVVASVTII DRSLPPIVKD
101 ASIQVVSAIR AAPEAARSLA SSLPGQTKIL AKVFYGEN
```

Matched peptide information:

Start - End	Observed	Mr(expt)	Mr(calc)	ppm	Miss	Sequence
17 - 40	2689.3086	2688.3013	2688.3476	-17	0	K.YLGFVQDAATYAVTTFSNVYLFAK.D (Ions score 156)
59 - 67	1045.5547	1044.5474	1044.5716	-23	0	K.NVAVPLYNR.F (Ions score 64)
68 - 77	1109.5700	1108.5627	1108.5917	-26	0	R.FSYIPNGALK.F (Ions score 61)
78 - 92	1621.8569	1620.8497	1620.8723	-14	0	K.FVDSTVVASVTIIDR.S (Ions score 139)
100 - 110	1158.6267	1157.6194	1157.6404	-18	0	K.DASIQVVSAIR.A (Ions score 109)

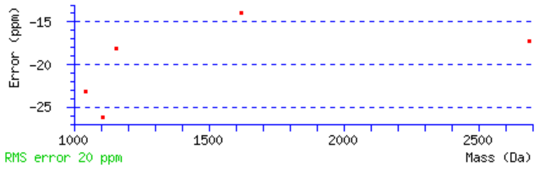

Spot No.:**49**

Accession No.: **scaffold0457_538964.mRNA1**

Protein name： **Alpha-soluble NSF attachment protein 2**

Peptide sequences:

K.HEAAQAYVDAAHCYK.K;K.TSTNEAISCLGQAVDLFCDIGR.I;K.AADFFQGEEVTTSANQCK.Q;K.AIEIYEEIAR.Q;K.GDVVAITNALER.Y;R.YQDLDPTFSGTR.D

PFF Mascot score: **[401]** Sequence coverage %: **[30]**

Matched peptides No.: **[6]**

Calculated Mr: **32767** Calculated *p*I: **5.05**

Data base searched result:

Ions score is -10*Log(P), where P is the probability that the observed match is a random event.
Individual ions scores > 31 indicate identity or extensive homology (p<0.05).
Protein scores are derived from ions scores as a non-probabilistic basis for ranking protein hits.

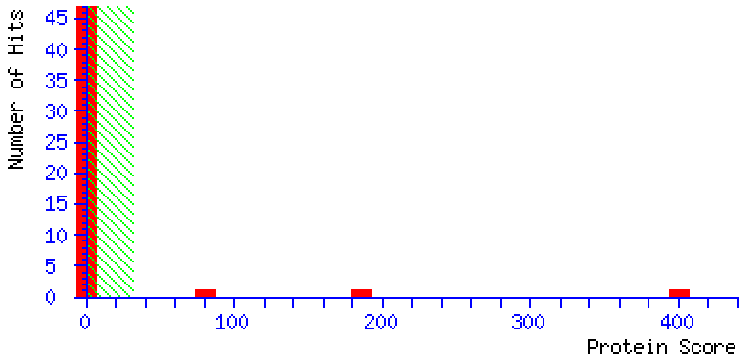

Matched peptide sequences: shown in Bold Red

1 MGDQIARGEE FEKKAEKKLN GWGLFGSKFE DAADLFDKAA NSFKLAKSWD
51 KAGSTYVKLA NCHLKLDSK**H** **EAAQAYVDAA** **HCYKKTSTNE** **AISCLGQAVD**
101 **LFCDIGR**ISM AARYYKEIGE LYESEANFEK AIDFYEK**AAD** **FFQGEEVTTS**
151 **ANQCK**QKVAQ FAAQLEQYQK **AIEIYEEIAR** QSLGNNLLKY GVKGHLLNAG
201 ICHLCK**GDVV** **AITNALERYQ** **DLDPTFSGTR** DYKLLADIAA AIDEEDIAKF
251 TDVVKEFDSM TPLDSWKTTL LLRVKEKLKA KELEEDDLT

Matched peptide information:

Start - End	Observed	Mr(expt)	Mr(calc)	ppm	Miss	Sequence
70 - 84	1733.7212	1732.7139	1732.7627	-28	0	K.HEAAQAYVDAAHCYK.K (Ions score 122)
86 - 107	2427.0647	2426.0574	2426.1206	-26	0	K.TSTNEAISCLGQAVDLFCDIGR.I (Ions score 109)
138 - 155	2002.8256	2001.8183	2001.8738	-28	0	K.AADFFQGEEVTTSANQCK.Q (Ions score 81)
171 - 180	1206.5988	1205.5915	1205.6292	-31	0	K.AIEIYEEIAR.Q (Ions score 80)
207 - 218	1257.6538	1256.6465	1256.6725	-21	0	K.GDVVAITNALER.Y (Ions score 72)
219 - 230	1399.6117	1398.6044	1398.6416	-27	0	R.YQDLDPTFSGTR.D (Ions score 88)

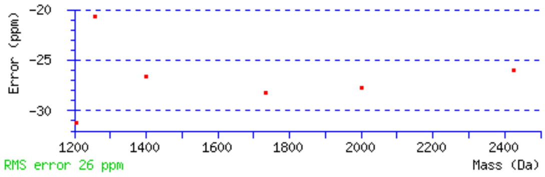

Spot No.:**50**

Accession No.: **scaffold1222_136753.mRNA1**

Protein name： **Rubber elongation factor protein**

Peptide sequences:

K.YLGFVQDAATYAVTTFSNVYLFAK.D;K.DKSGPLQPGVDIIEGPVK.N;K.SGPLQPGVDIIEGPVKNVAVPLYNR.F;R.NVAVPLYNR.F;R.FSYIPNGALK.F;K.FVDSTVVASVTIIDR.S;K.DASIQVVSAIR.A

PFF Mascot score: **[480]**   Sequence coverage %: **[63]**

Matched peptides No.: **[7]**

Calculated Mr: **14713**   Calculated *p*I: **5.04**

Data base searched result:

Ions score is -10*Log(P), where P is the probability that the observed match is a random event.
Individual ions scores > 31 indicate identity or extensive homology (p<0.05).
Protein scores are derived from ions scores as a non-probabilistic basis for ranking protein hits.

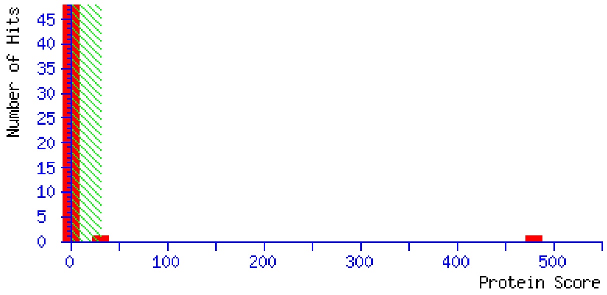

Matched peptide sequences: shown in Bold Red

```
  1 MAEDEDNQQG QGEGLKYLGF VQDAATYAVT TFSNVYLFAK DKSGPLQPGV
 51 DIIEGPVKNV AVPLYNRFSY IPNGALKFVD STVVASVTII DRSLPPIVKD
101 ASIQVVSAIR AAPEAARSLA SSLPGQTKIL AKVFYGEN
```

Matched peptide information:

Start - End	Observed	Mr(expt)	Mr(calc)	ppm	Miss	Sequence
17 - 40	2689.2725	2688.2652	2688.3476	-31	0	K.YLGFVQDAATYAVTTFSNVYLFAK.D (Ions score 134)
41 - 58	1848.9608	1847.9535	1847.9993	-25	1	K.DKSGPLQPGVDIIEGPVK.N (Ions score 132)
43 - 67	2632.3535	2631.3462	2631.4384	-35	1	K.SGPLQPGVDIIEGPVKNVAVPLYNR.F (Ions score 27)
59 - 67	1045.5453	1044.5380	1044.5716	-32	0	K.NVAVPLYNR.F (Ions score 69)
68 - 77	1109.5633	1108.5561	1108.5917	-32	0	R.FSYIPNGALK.F (Ions score 55)
78 - 92	1621.8395	1620.8322	1620.8723	-25	0	K.FVDSTVVASVTIIDR.S (Ions score 131)
100 - 110	1158.6158	1157.6086	1157.6404	-28	0	K.DASIQVVSAIR.A (Ions score 103)

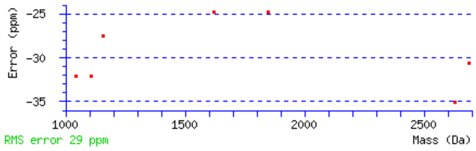

Spot No.:51

Accession No.: scaffold0884_428899.mRNA1

Protein name：Triosephosphateisomerase, cytosolic

Peptide sequences:

K.FFVGGNWK.C;K.DLLRPDFQVAAQNCWVR.K;R.SLLNESNEFVGDK.V;K.VIACIGETLEQR.E;K.VATPAQAQEVHLELR.K;K.VATPAQAQEVHLELRK.W;K.WLHDNVCAEVAASTR.I

PFF Mascot score: [451] Sequence coverage %: [32]

Matched peptides No.: [7]

Calculated Mr: 27571 Calculated *p*I: 5.90

Data base searched result:

Ions score is -10*Log(P), where P is the probability that the observed match is a random event.
Individual ions scores > 31 indicate identity or extensive homology (p<0.05).
Protein scores are derived from ions scores as a non-probabilistic basis for ranking protein hits.

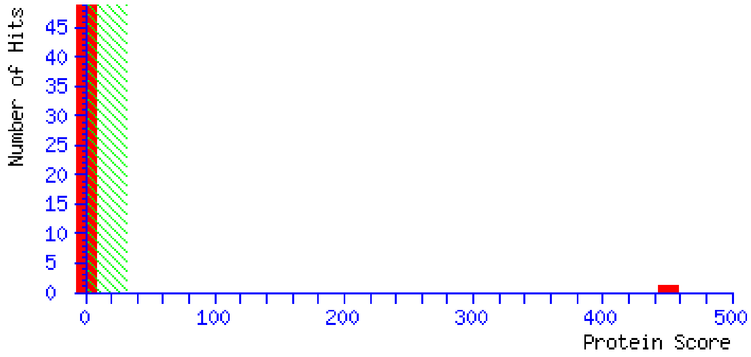

Matched peptide sequences: shown in Bold Red

```
  1 MARKFFVGGN WKCNGATEEV KKIVTTLNEA EVPSHDVVEV VVSPPFVFIP
 51 PVKDLLRPDF QVAAQNCWVR KGGAFTGEVS AEMLVNLSVP WVILGHSERR
101 SLLNESNEFV GDKVAYALSQ GLKVIACIGE TLEQRESGST MAVVAAQTKA
151 IAEKVTNWTN VVLAYEPVWA IGTGKVATPA QAQEVHLELR KWLHDNVCAE
201 VAASTRIIYG GSVNGANCKE LAAKPDVDGF LVGGASLKPE FIDIIKSATV
251 KKE
```

Matched peptide information:

Start - End	Observed	Mr(expt)	Mr(calc)	ppm	Miss	Sequence
5 - 12	954.4294	953.4222	953.4760	-56	0	K.FFVGGNWK.C (Ions score 58)
54 - 70	2087.9614	2086.9542	2087.0371	-40	1	K.DLLRPDFQVAAQNCWVR.K (Ions score 113)
101 - 113	1451.6323	1450.6250	1450.6940	-48	0	R.SLLNESNEFVGDK.V (Ions score 77)
124 - 135	1388.6635	1387.6562	1387.7129	-41	0	K.VIACIGETLEQR.E (Ions score 89)
176 - 190	1661.8309	1660.8237	1660.8896	-40	0	K.VATPAQAQEVHLELR.K (Ions score 131)
176 - 191	1789.9222	1788.9150	1788.9846	-39	1	K.VATPAQAQEVHLELRK.W (Ions score 40)
192 - 206	1728.7439	1727.7366	1727.8049	-40	0	K.WLHDNVCAEVAASTR.I (Ions score 127)

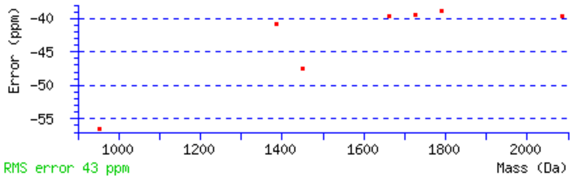

Spot No.:**52**

Accession No.: **scaffold1479_76107.mRNA1**

Protein name： **Acetyl-CoA acetyltransferase, cytosolic 1**

Peptide sequences:

R.ANVDPSLVQEVFFGNVLSANLGQAPAR.Q;R.EDQDNYAIHSFER.G;R.GIAAQDSGAFAWEIVPVEVSGGR.G;K.VNVHGGAVSLGHPLGCSGAR.I

PFF Mascot score: **[299]** Sequence coverage %: **[20]**

Matched peptides No.: **[4]**

Calculated Mr: **41646** Calculated *p*I: **6.01**

Data base searched result:

Ions score is -10*Log(P), where P is the probability that the observed match is a random event.
Individual ions scores > 31 indicate identity or extensive homology (p<0.05).
Protein scores are derived from ions scores as a non-probabilistic basis for ranking protein hits.

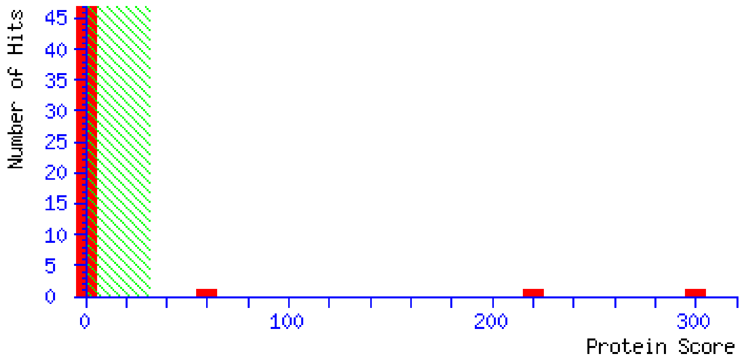

Matched peptide sequences: shown in Bold Red

1 MAPVAAAEIK PRDVCIVGVA RTPMGGFLGL LSTLPATKLG SIAIEAALKR
51 **ANVDPSLVQE VFFGNVLSAN LGQAPAR**QAA LGAGIPNSVV CTTVNKVCAS
101 GMKATMLAAQ SIQLGINDVV VAGGMESMSN APKYLAEARK GSRLGHDSVV
151 DGMLKDGLWD VFNDVGMGSC AEICADNHSI TR**EDQDNYAI HSFERGIAAQ**
201 **DSGAFAWEIV PVEVSGGR**GK PSTIVDKDEG LGKFDPVKLR KLRPSFKENG
251 GTVTAGNASS ISDGAAALVL VSGETALKLG LQVIAKITGY ADAAQAPELF
301 TTAPALAIPK AVSNAGLDAS QVDYYEINEA FAVVALANQK LLGLNPEK**VN**
351 **VHGGAVSLGH PLGCSGAR**IL VTLLGVLRQK NGKYGVGGVC NGGGGASALV
401 VELL

Matched peptide information:

Start - End	Observed	Mr(expt)	Mr(calc)	ppm	Miss	Sequence
51 - 77	2813.3088	2812.3016	2812.4508	-53	0	R.ANVDPSLVQEVFFGNVLSANLGQAPAR.Q (Ions score 84)
183 - 195	1623.6234	1622.6161	1622.6961	-49	0	R.EDQDNYAIHSFER.G (Ions score 99)
196 - 218	2316.0535	2315.0462	2315.1546	-47	0	R.GIAAQDSGAFAWEIVPVEVSGGR.G (Ions score 156)
349 - 368	1944.8862	1943.8790	1943.9748	-49	0	K.VNVHGGAVSLGHPLGCSGAR.I (Ions score 40)

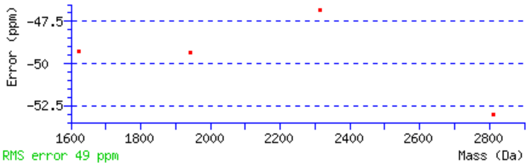

Spot No.:**53**

Accession No.: **scaffold0438_1023124.mRNA1**

Protein name：**Probable phosphatase SPAC5H10.03**

Peptide sequences:

K.KIDLVITSPLLR.A;R.AMQTAVGVFGGER.S;K.SPPIVAVELCR.E;K.TRQETEIAVVTHHR.F;R.QETEIAVVTHHR.F;R.FLQYTLNALANDFHPSVR.S;K.EFVNCELR.S

PFF Mascot score: **[454]** Sequence coverage %: **[27]**

Matched peptides No.: **[7]**

Calculated Mr: **31328** Calculated *p*I: **7.07**

Data base searched result:

Ions score is -10*Log(P), where P is the probability that the observed match is a random event.
Individual ions scores > 31 indicate identity or extensive homology (p<0.05).
Protein scores are derived from ions scores as a non-probabilistic basis for ranking protein hits.

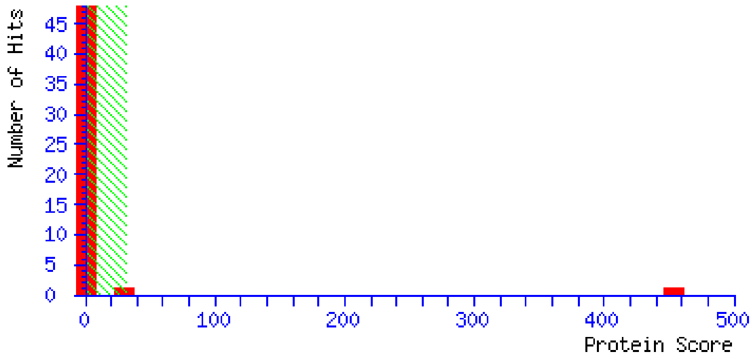

Matched peptide sequences: shown in Bold Red

```
  1 MIFFSFIRNF ILRRAYFLRK LCVFLFSFSY LDSADMDSTT SQCLIPLGHS
 51 KIIHLVRHAQ ANHNVAGKKD LGALLSPEFF DAQLSPLGLE QVSNLCNNVN
101 TSGLFKKIDL VITSPLLRAM QTAVGVFGGE RSSGLKSPPI VAVELCRERT
151 GVHPCDKRRT ITEYSSLFPQ IDFSLMESDD DNLWKADVRE TDEEVAARGL
201 KFMNWLKTRQ ETEIAVVTHH RFLQYTLNAL ANDFHPSVRS EMCKEFVNCE
251 LRSMVIVDKR MMNCPATDGS GGSV
```

Matched peptide information:

Start	-	End	Observed	Mr(expt)	Mr(calc)	ppm	Miss	Sequence
107	-	118	1367.7915	1366.7842	1366.8548	-52	1	K.KIDLVITSPLLR.A (Ions score 92)
119	-	131	1322.5806	1321.5733	1321.6449	-54	0	R.AMQTAVGVFGGER.S (Ions score 119)
137	-	147	1240.6148	1239.6075	1239.6645	-46	0	K.SPPIVAVELCR.E (Ions score 75)
208	-	221	1676.7913	1675.7840	1675.8754	-55	1	K.TRQETEIAVVTHHR.F (Ions score 88)
210	-	221	1419.6571	1418.6498	1418.7266	-54	0	R.QETEIAVVTHHR.F (Ions score 75)
222	-	239	2105.9717	2104.9644	2105.0694	-50	0	R.FLQYTLNALANDFHPSVR.S (Ions score 122)
245	-	252	1066.4375	1065.4302	1065.4913	-57	0	K.EFVNCELR.S (Ions score 67)

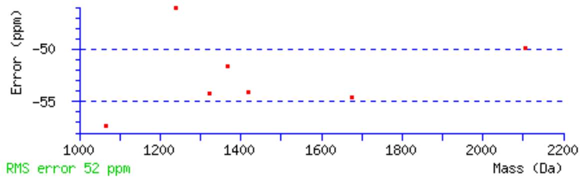

Spot No.:**54**

Accession No.: **scaffold0878_145544.mRNA1**

Protein name：**Importin subunit alpha-1b**

Peptide sequences:

R.LEHLPSMVAGVWSDDSNLLLEATTQFR.K;R.SPPIEEVIQAGVVPR.F;R.EDFPQLQFEAAWALTNIASGTSENTR.V;R.NATWTLSNFCR.G;K.IQAVIEAGVCPR.L;R.LVELLLHPSPSVLIPALR.T

PFF Mascot score: **[441]** Sequence coverage %: **[20]**

Matched peptides No.: **[6]**

Calculated Mr: **59161** Calculated *p*I: **5.28**

Data base searched result:

Ions score is -10*Log(P), where P is the probability that the observed match is a random event.
Individual ions scores > 30 indicate identity or extensive homology (p<0.05).
Protein scores are derived from ions scores as a non-probabilistic basis for ranking protein hits.

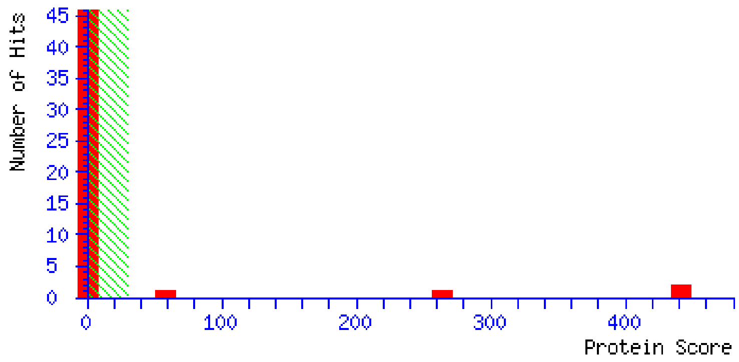

Matched peptide sequences: shown in Bold Red

```
  1 MSLRPSARTE VRRNRYKVAV DAEESRRRRE DNMVEIRKNR REESLQKKRR
 51 EGLQTQPMPA AIHSSAVEKR LEHLPSMVAG VWSDDSNLLL EATTQFRKLL
101 SIERSPPIEE VIQAGVVPRF VEFLMREDFP QLQFEAAWAL TNIASGTSEN
151 TRVVIDHGAV PIFVKLLGSP SDDVREQAVW ALGNVAGDSP KCRDLVLGHG
201 ALIPLLAQLN EHAKLSMLRN ATWTLSNFCR GKPQPPFDQV KPALPALAHL
251 IHSNDEEVLT DACWALSYLS DGTNDKIQAV IEAGVCPRLV ELLLHPSPSV
301 LIPALRTVGN IVTGDDMQTQ CIINHQALPC LLNLLTNNYK KSIKKEACWT
351 ISNITAGNKE QIQAVIEANI IAPLVHLLQN AEFDIKKEAA WAISNATSGG
401 THDQIKYLVS QGCIKPLCDL LICPDPRIVT VCLEGLENIL KVGEADKNLG
451 STGGVNQYAQ MIDDAEGLEK IENLQSHDNT EIYEKAVKIL ETYWLEEEDE
501 TMPPGDASQS GFQFGGSEMP SVPSGGFNFS
```

Matched peptide information:

Start	-	End	Observed	Mr(expt)	Mr(calc)	ppm	Miss	Sequence
71	-	97	3029.3818	3028.3746	3028.4964	-40	0	R.LEHLPSMVAGVWSDDSNLLLEATTQFR.K (Ions score 32)
105	-	119	1590.8232	1589.8160	1589.8777	-39	0	R.SPPIEEVIQAGVVPR.F (Ions score 142)
127	-	152	2896.2463	2895.2391	2895.3675	-44	0	R.EDFPQLQFEAAWALTNIASGTSENTR.V (Ions score 186)
220	-	230	1369.5855	1368.5782	1368.6245	-34	0	R.NATWTLSNFCR.G (Ions score 57)
277	-	288	1312.6559	1311.6486	1311.6969	-37	0	K.IQAVIEAGVCPR.L (Ions score 79)
289	-	306	1967.1338	1966.1265	1966.1979	-36	0	R.LVELLLHPSPSVLIPALR.T (Ions score 86)

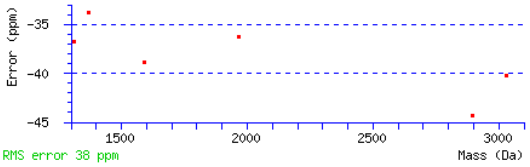

Spot No.: **55**

Accession No.: **scaffold0331_834438.mRNA1**

Protein name: **Phospholipase D alpha 1**

Peptide sequences:

R.AYVPVEELLDGQEIDR.W;K.YPGVPYSFYSQR.Q;R.VLMLVWDDR.T;R.IVSYVGGIDLCDGR.Y;R.YDTPFHSLFR.T;R.LEGPIAWDVLFNFEQR.W;K.SGEYEPSEKPEADSDYLR.A;K.MMIVDDEYIIIGSANINQR.S;K.YWDLYSSETLEHDLPGHLLR.Y

PFF Mascot score: **[487]** Sequence coverage %: **[17]**

Matched peptides No.: **[9]**

Calculated Mr: **88898** Calculated *p*I: **5.60**

Data base searched result:

Ions score is -10*Log(P), where P is the probability that the observed match is a random event.
Individual ions scores > 31 indicate identity or extensive homology (p<0.05).
Protein scores are derived from ions scores as a non-probabilistic basis for ranking protein hits.

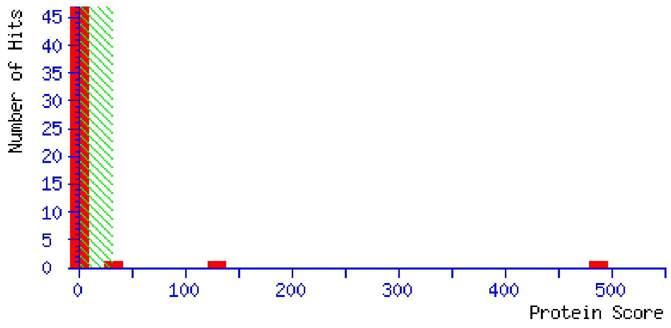

Matched peptide sequences: shown in Bold Red

```
  1 MENVEATLGF GKGSSKLYAT VDLEKARVGR TRVLEKEDTN PRWYESFIIY
 51 CAHLASNVIF TIKERNPIGA TLIGRAYVPV EELLDGQEID RWVEILDKEK
101 NPIHGGSKIH VKLQYFDVTK DPNWGRGIRS PKYPGVPYSF YSQRQGCKVS
151 LYQDAHVPDK FVPKIPLAGG KYYEPHRCWE DVFDAITNAK HLIYITGWSV
201 YTEISLVRDS RRPKSGGDIT LGELLKKKAS EGVRVLMLVW DDRTSVGLLK
251 KDGLMATHDE ETEHYFQNTD VHCILCPRNP DDGGSIVQDL SISTMFTHHQ
301 KIVVVDSAMP NGDSQRRRIV SYVGGIDLCD GRYDTPFHSL FRTLDTAHHD
351 DFHQPNFTGA SIQKGGPREP WHDIHSRLEG PIAWDVLFNF EQRWKKQGGK
401 DLLVQLRELE DIIISPSPVM YPDDYETWNV QLFRSIDGGA AFGFPETPED
451 AARAGLISGK DNIIDRSIQD AYIHAIRRAK NFIYIENQYF LGSSFCWSPD
501 GIKPEDINAL HVIPKELSLK IVSKIEAGER FTVYVVVPMW PEGIPESGSV
551 QAILDWQKRT MEVMYRDVVQ ALKAKGIEEN LRNYLTFFCL GNREAKKSGE
601 YEPSEKPEAD SDYLRAQEAR RFMIYVHAKM MIVDDEYIII GSANINQRSM
651 DGARDSEIAM GAYQPYHLST REPARGQIHG FRMALWYEHL GMLDETFLYP
701 ESEECVRKVN QIADKYWDLY SSETLEHDLP GHLLRYPVGI ASEGDVTELP
751 GTEFFPDTKA RVLGAKSDYL PPMLTT
```

Matched peptide information:

Start - End	Observed	Mr(expt)	Mr(calc)	ppm	Miss	Sequence
76 - 91	1845.8320	1844.8248	1844.9156	-49	0	R.AYVPVEELLDGQEIDR.W (Ions score 106)
133 - 144	1463.6245	1462.6172	1462.6881	-48	0	K.YPGVPYSFYSQR.Q (Ions score 69)
235 - 243	1146.5363	1145.5290	1145.5903	-54	0	R.VLMLVWDDR.T (Ions score 44)
319 - 332	1523.6788	1522.6716	1522.7450	-48	0	R.IVSYVGGIDLCDGR.Y (Ions score 79)
333 - 342	1282.5560	1281.5488	1281.6142	-51	0	R.YDTPFHSLFR.T (Ions score 45)
378 - 393	1933.8874	1932.8802	1932.9734	-48	0	R.LEGPIAWDVLFNFEQR.W (Ions score 104)
598 - 615	2071.8030	2070.7957	2070.9018	-51	1	K.SGEYEPSEKPEADSDYLR.A (Ions score 86)
630 - 648	2194.9773	2193.9700	2194.0762	-48	0	K.MMIVDDEYIIIGSANINQR.S (Ions score 109)
716 - 735	2444.0608	2443.0535	2443.1808	-52	0	K.YWDLYSSETLEHDLPGHLLR.Y (Ions score 81)

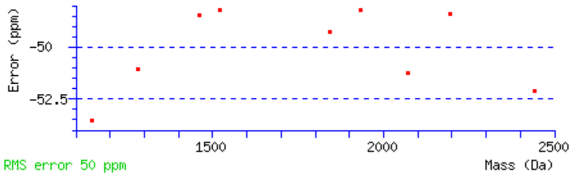

Spot No.:**56**

Accession No.: **scaffold0280_1322570.mRNA1**

Protein name： **Dynamin-related protein 4C**

Peptide sequences:

K.SSVLESLAGINLPR.G;K.GIPDLTMIDLPGITR.V;R.GEFDEYLNDHQMHCTAR.L;R.LVEMLNLYSNELHK.C;R.NFLVEEIR.L;K.LMNQQDTFR.G;K.AHQDVLHQAFDLK.M;K.EMEKEIISELMSNQGGVIER.M;K.EIISEL MSNQGGVIER.M

PFF Mascot score: **[538]** Sequence coverage %: **[16]**

Matched peptides No.: **[9]**

Calculated Mr: **74315** Calculated *p*I: **5.91**

Data base searched result:

Ions score is -10*Log(P), where P is the probability that the observed match is a random event.
Individual ions scores > 31 indicate identity or extensive homology (p<0.05).
Protein scores are derived from ions scores as a non-probabilistic basis for ranking protein hits.

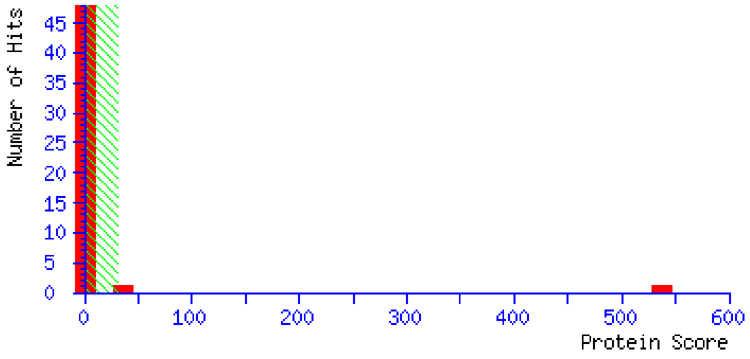

Matched peptide sequences: shown in Bold Red

```
  1 MAPISNGPDL VEYEDANMEN QVPLVSSYND HIRPLLDAVD KLRHLKVMNE
 51 GIQLPTIVVV GDQSSGKSSV LESLAGINLP RGQGICTRVP LVMRLQHQPT
101 PTPEVFLEFN GKTVYTDEAR AADAISLATD EIAGDGKGIS NTPLTLVVKK
151 KGIPDLTMID LPGITRVPVH GQPEDIYEQI TGIITEYIRP EESIILNVLS
201 ATVDFPTCES IRMSRQVDKT GERTLAVVTK SDKAPEGLLE KVAADDVNIG
251 LGYVCVKNRI GDESFEDARK EEAQLFKKHP LLSRIDKSMV GIPFLAQKLT
301 QIQATIIAKC LPDIARNINE KLNASISELN KMPRTPSSPA EAMTAFMGIV
351 GSAKESLRKI LIRGEFDEYL NDHQMHCTAR LVEMLNLYSN ELHKCSESDP
401 TRNFLVEEIR LLEESKGIKL PNFLPHSALL SILQKKVDGI SRMHIDFVEK
451 IWDYIESVVL SVLMHHSENY HQLQSSTRRA GQNLISKMKE QSIGWVTEII
501 QMEKMTDYTC NPNYMSEWSK LMNQQDTFRG KILIQGHSKA KIDGIGEVEA
551 GHIKAHQDVL HQAFDLKMRM TAYWKIVLSR LVDSMALHLQ FCVQNLVNKE
601 MEKEIISELM SNQGGVIERM MEESPSIAAK REKLNKSIKL LGESKKVLGN
651 IMDKIATYSD
```

Matched peptide information:

Start	-	End	Observed	Mr(expt)	Mr(calc)	ppm	Miss	Sequence	
68	-	81	1455.7542	1454.7469	1454.8093	-43	0	K.SSVLESLAGINLPR.G	(Ions score 96)
152	-	166	1611.8091	1610.8018	1610.8702	-42	0	K.GIPDLTMIDLPGITR.V	(Ions score 103)
364	-	380	2122.7825	2121.7752	2121.8633	-42	0	R.GEFDEYLNDHQMHCTAR.L	(Ions score 106)
381	-	394	1702.8033	1701.7961	1701.8759	-47	0	R.LVEMLNLYSNELHK.C	(Ions score 67)
403	-	410	1019.5070	1018.4997	1018.5447	-44	0	R.NFLVEEIR.L	(Ions score 50)
521	-	529	1152.4987	1151.4914	1151.5393	-42	0	K.LMNQQDTFR.G	(Ions score 49)
555	-	567	1521.7086	1520.7013	1520.7736	-47	0	K.AHQDVLHQAFDLK.M	(Ions score 104)
600	-	619	2292.0286	2291.0213	2291.1137	-40	1	K.EMEKEIISELMSNQGGVIER.M	(Ions score 37)
604	-	619	1774.8323	1773.8250	1773.8931	-38	0	K.EIISELMSNQGGVIER.M	(Ions score 159)

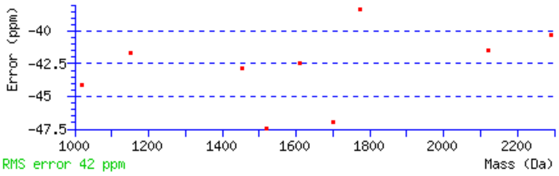

Spot No.:**57**

Accession No.: **scaffold4512_855.mRNA1**

Protein name：**Osmotin-like protein OSM34**

Peptide sequences:

R.NNCPYTVWAAASPGGGR.R;R.LDQGQTWELNVPAGTSMAR.I;R.TNCNFDGSGK.G;K.APGGCNNPCTVFK.T;R.CSDAYSYPQDDPSSTFTCPGGTNYR.V

PFF Mascot score: **[453]** Sequence coverage %: **[34]**

Matched peptides No.: **[5]**

Calculated Mr: **27682** Calculated *p*I: **5.23**

Data base searched result:

Ions score is -10*Log(P), where P is the probability that the observed match is a random event.
Individual ions scores > 31 indicate identity or extensive homology (p<0.05).
Protein scores are derived from ions scores as a non-probabilistic basis for ranking protein hits.

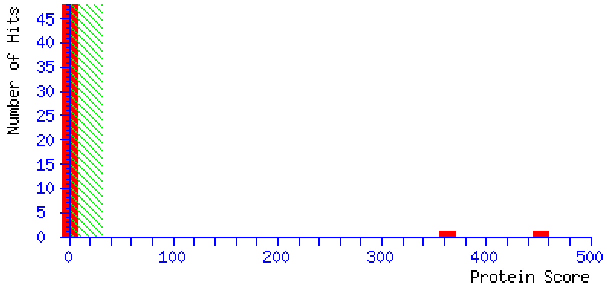

Matched peptide sequences: shown in Bold Red

```
  1 MSNFNIFLIS IFLLSALFFT SSDGATFTIR NNCPYTVWAA ASPGGGRRLD
 51 QGQTWELNVP AGTSMARIWG RTNCNFDGSG KGHCQTGDCG GILACQGWGV
101 PPNTLAEYAL NQFGNLDFYD ISLVDGFNIP IEFSPTSGAK DKCRPLFCTA
151 DINGQCPNQL KAPGGCNNPC TVFKTNEYCC TEGYGTCGPT EFSKFFKSRC
201 SDAYSYPQDD PSSTFTCPGG TNYRVVFCPA RSPHFPLEMV REKDVE
```

Matched peptide information:

Start - End	Observed	Mr(expt)	Mr(calc)	ppm	Miss	Sequence
31 - 47	1777.7324	1776.7251	1776.8002	-42	0	R.NNCPYTVWAAASPGGGR.R (Ions score 165)
49 - 67	2073.9529	2072.9456	2072.9949	-24	0	R.LDQGQTWELNVPAGTSMAR.I (Ions score 92)
72 - 81	1099.3859	1098.3786	1098.4400	-56	0	R.TNCNFDGSGK.G (Ions score 36)
162 - 174	1421.5599	1420.5527	1420.6228	-49	0	K.APGGCNNPCTVFK.T (Ions score 82)
200 - 224	2846.0019	2844.9947	2845.1232	-45	0	R.CSDAYSYPQDDPSSTFTCPGGTNYR.V (Ions score 198)

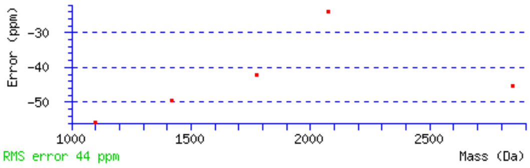

Spot No.:**58**

Accession No.: **scaffold4512_855.mRNA1**

Protein name：**Osmotin-like protein OSM34**

Peptide sequences:

R.NNCPYTVWAAASPGGGR.R;R.LDQGQTWELNVPAGTSMAR.I;K.APGGCNNPCTVFK.T;R.CSDAYSYPQDDPSSTFTCPGGTNYR.V

PFF Mascot score: **[348]**

Sequence coverage %: **[30]**

Matched peptides No.: **[4]**

Calculated Mr: **27682**

Calculated *p*I: **5.23**

Data base searched result:

Ions score is -10*Log(P), where P is the probability that the observed match is a random event.
Individual ions scores > 31 indicate identity or extensive homology (p<0.05).
Protein scores are derived from ions scores as a non-probabilistic basis for ranking protein hits.

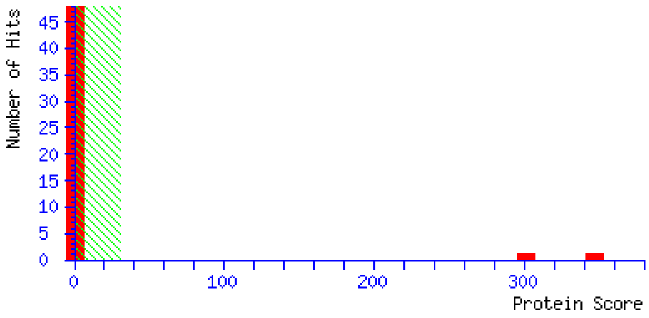

Matched peptide sequences: shown in Bold Red

```
  1 MSNFNIFLIS IFLLSALFFT SSDGATFTIR NNCPYTVWAA ASPGGGRRLD
 51 QGQTWELNVP AGTSMARIWG RTNCNFDGSG KGHCQTGDCG GILACQGWGV
101 PPNTLAEYAL NQFGNLDFYD ISLVDGFNIP IEFSPTSGAK DKCRPLFCTA
151 DINGQCPNQL KAPGGCNNPC TVFKTNEYCC TEGYGTCGPT EFSKFFKSRC
201 SDAYSYPQDD PSSTFTCPGG TNYRVVFCPA RSPHFPLEMV REKDVE
```

Matched peptide information:

Start	-	End	Observed	Mr(expt)	Mr(calc)	ppm	Miss	Sequence
31	-	47	1777.7688	1776.7615	1776.8002	-22	0	R.NNCPYTVWAAASPGGGR.R (Ions score 139)
49	-	67	2073.9902	2072.9830	2072.9949	-6	0	R.LDQGQTWELNVPAGTSMAR.I (Ions score 68)
162	-	174	1421.5964	1420.5892	1420.6228	-24	0	K.APGGCNNPCTVFK.T (Ions score 81)
200	-	224	2846.0400	2845.0328	2845.1232	-32	0	R.CSDAYSYPQDDPSSTFTCPGGTNYR.V (Ions score 144)

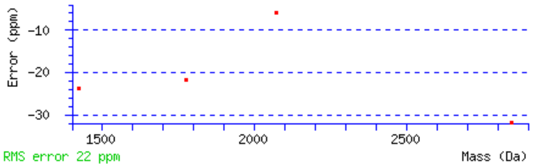

Spot No.:**59**

Accession No.: **scaffold0625_1132.mRNA1**

Protein name：**Glucan endo-1,3-beta-glucosidase, basic vacuolar isoform**

Peptide sequences:

R.IYDPNQAVLEALR.G;K.VSTAIDLTLVGNSYPPSAGAFR.D;R.SYLDPIIGFLSSIR.S;R.DISLPYALFTSPSVVVWDGQR.G;K.NLFDATLDALYSALER.A;K.HFGLFFPDK.R

PFF Mascot score: **[691]** Sequence coverage %: **[25]**

Matched peptides No.: **[6]**

Calculated Mr: **41184** Calculated *p*I: **7.77**

Data base searched result:

Ions score is -10*Log(P), where P is the probability that the observed match is a random event.
Individual ions scores > 31 indicate identity or extensive homology (p<0.05).
Protein scores are derived from ions scores as a non-probabilistic basis for ranking protein hits.

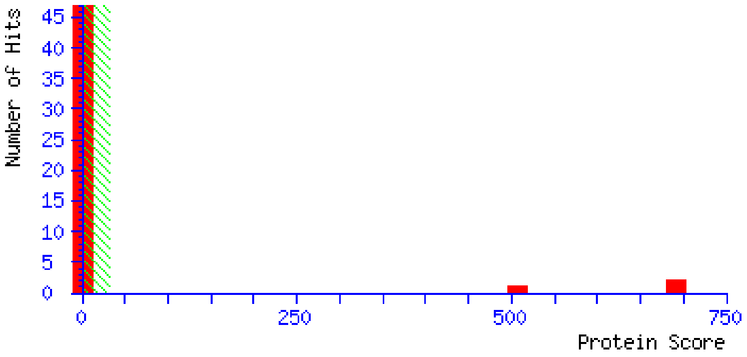

Matched peptide sequences: shown in Bold Red

1 MAISSSTSGT SSSLPSRTTV MLLLIFFTAS LGITDAQVGV CYGMQGNNLP
51 SVSEVIALYK QSNIKRMR**IY** **DPNQAVLEAL** **R**GSNIELILG VPNSDLQSLT
101 NPSNANSWVQ KNVRGFWSSV RFRYIAVGNE ISPVNGGTAW LAQFVLPAMR
151 NIHDAIRSAG LQDQIK**VSTA** **IDLTLVGNSY** **PPSAGAFR**DD VR**SYLDPIIG**
201 **FLSSIR**SPLL ANIYPYFTYA DNPR**DISLPY** **ALFTSPSVVV** **WDGQR**GYK**NL**
251 **FDATLDALYS** **ALER**ASGGSL EVVVSESGWP SAGAFAATFD NGRTYLSNLI
301 QHVKGGTPKR PNRAIETYLF AMFDENKKQP EVEK**HFGLFF** **PDK**RPKYNLN
351 FGAEKNWDIS TEHNATILFL KSDM

Matched peptide information:

Start - End	Observed	Mr(expt)	Mr(calc)	ppm	Miss	Sequence
69 - 81	1501.7440	1500.7367	1500.7936	-38	0	R.IYDPNQAVLEALR.G (Ions score 124)
167 - 188	2236.0847	2235.0774	2235.1535	-34	0	K.VSTAIDLTLVGNSYPPSAGAFR.D (Ions score 206)
193 - 206	1580.8102	1579.8029	1579.8610	-37	0	R.SYLDPIIGFLSSIR.S (Ions score 126)
225 - 245	2350.1265	2349.1192	2349.2005	-35	0	R.DISLPYALFTSPSVVVWDGQR.G (Ions score 179)
249 - 264	1811.8558	1810.8486	1810.9101	-34	0	K.NLFDATLDALYSALER.A (Ions score 153)
335 - 343	1107.5111	1106.5038	1106.5549	-46	0	K.HFGLFFPDK.R (Ions score 55)

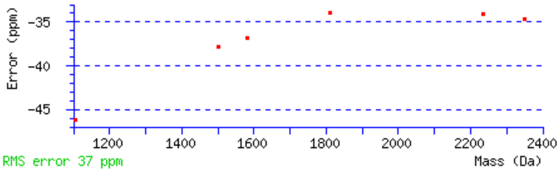

Spot No.:**60**

Accession No.: **scaffold0444_175845.mRNA1**

Protein name：**Ubiquitin-conjugating enzyme E2 35**

Peptide sequences:

R.LLSEPAPGISASPSEDNMR.Y;R.YFNVMILGPTQSPYEGGVFK.L;K.LELFLPEEYPMAAPK.V;K.DKWSPALQIR.T;K.WSPALQIR.T;R.TVLLSIQALLSAPNPDDPLSENIAK.H

PFF Mascot score: **[389]** Sequence coverage %: **[58]**

Matched peptides No.: **[6]**

Calculated Mr: **17266** Calculated *p*I: **6.74**

Data base searched result:

Ions score is -10*Log(P), where P is the probability that the observed match is a random event.
Individual ions scores > 31 indicate identity or extensive homology (p<0.05).
Protein scores are derived from ions scores as a non-probabilistic basis for ranking protein hits.

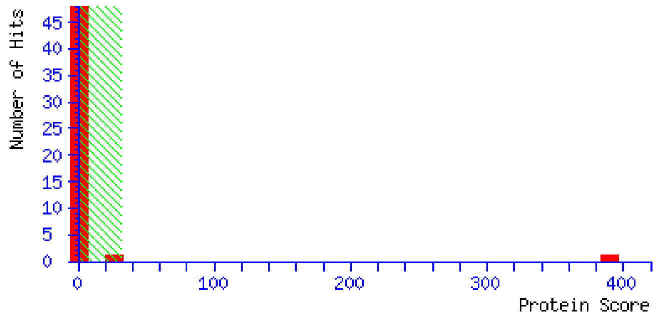

Matched peptide sequences: shown in Bold Red

```
  1 MANSNLPRRI IKETQRLLSE PAPGISASPS EDNMRYFNVM ILGPTQSPYE
 51 GGVFKLELFL PEEYPMAAPK VRFLTKIYHP NIDKLGRICL DILKDKWSPA
101 LQIRTVLLSI QALLSAPNPD DPLSENIAKH WKTNEAEAVE TAKEWTRLYA
151 SGA
```

Matched peptide information:

Start - End	Observed	Mr(expt)	Mr(calc)	ppm	Miss	Sequence
17 - 35	1970.8882	1969.8809	1969.9415	-31	0	R.LLSEPAPGISASPSEDNMR.Y (Ions score 146)
36 - 55	2247.0447	2246.0374	2246.1082	-32	0	R.YFNVMILGPTQSPYEGGVFK.L (Ions score 69)
56 - 70	1747.8298	1746.8226	1746.8902	-39	0	K.LELFLPEEYPMAAPK.V (Ions score 86)
95 - 104	1213.6210	1212.6137	1212.6615	-39	1	K.DKWSPALQIR.T (Ions score 92)
97 - 104	970.4978	969.4905	969.5396	-51	0	K.WSPALQIR.T (Ions score 64)
105 - 129	2619.3374	2618.3301	2618.4167	-33	0	R.TVLLSIQALLSAPNPDDPLSENIAK.H (Ions score 76)

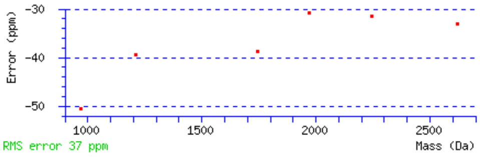

Spot No.:**61**

Accession No.: **scaffold0664_98467.mRNA1**

Protein name： **Caffeic acid 3-O-methyltransferase**

Peptide

sequences:K.SALELNVIDIISTAGNSGASLSAPEIAQR.I;K.NPEAPVLLDR.M;K.SWYHLNEAILEGGTPFNR.A;R.AYGMNQFEYPGTDQR.F;K.WVLHDWNDDLCLK.L;R.TRQEFEALASK.S

PFF Mascot score:**[456]** Sequence coverage %: **[29]**

Matched peptides No.: **[6]**

Calculated Mr:**36827**Calculated *p*I: **5.54**

Data base searched result:

Ions score is -10*Log(P), where P is the probability that the observed match is a random event.
Individual ions scores > 31 indicate identity or extensive homology (p<0.05).
Protein scores are derived from ions scores as a non-probabilistic basis for ranking protein hits.

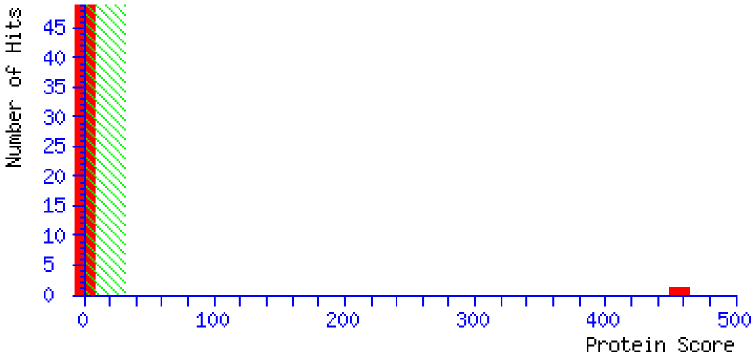

Matched peptide sequences: shown in Bold Red

```
  1 MTGSEQSNSA SIMSGRSDEE TWNLAIDLAN TVILPMVLKS ALELNVIDII
 51 STAGNSGASL SAPEIAQRIP EAKNPEAPVL LDRMLRLLAT YDIVKCSSNT
101 KENGEAERLY APGPICKFLT KNKNGSGSAA PLLLLHHDEV FMKSWYHLNE
151 AILEGGTPFN RAYGMNQFEY PGTDQRFNRV FNDAMSSYTT YLVKKILDAY
201 KGFDGLKSLV DVGGNSGVTL NSITSKYPHI KGINYDLPHV LADAPSFPGV
251 EHVAGDMFKS VPKGDAIILK WVLHDWNDDL CLKLLKNCWE ALPSNERTRQ
301 EFEALASKSG FSSCEFICCA YNSWVIEFHK
```

Matched peptide information:

Start - End	Observed	Mr(expt)	Mr(calc)	ppm	Miss	Sequence
40 - 68	2897.4597	2896.4524	2896.5141	-21	0	K.SALELNVIDIISTAGNSGASLSAPEIAQR.I (Ions score 189)
74 - 83	1123.5945	1122.5872	1122.6033	-14	0	K.NPEAPVLLDR.M (Ions score 69)
144 - 161	2103.9902	2102.9830	2103.0174	-16	0	K.SWYHLNEAILEGGTPFNR.A (Ions score 149)
162 - 176	1776.7351	1775.7278	1775.7573	-17	0	R.AYGMNQFEYPGTDQR.F (Ions score 95)
271 - 283	1713.7689	1712.7616	1712.7981	-21	0	K.WVLHDWNDDLCLK.L (Ions score 71)
298 - 308	1279.6480	1278.6407	1278.6568	-13	1	R.TRQEFEALASK.S (Ions score 20)

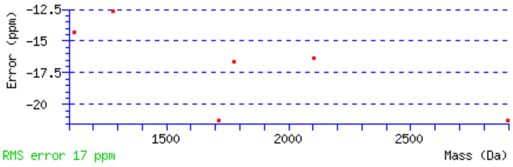

Spot No.: **62**

Accession No.: **scaffold0066_550349.mRNA1**

Protein name：**Probable prefoldin subunit 5**

Peptide sequences:

K.ALKEQTDLEVNLLQESLNNIR.T;K.EQTDLEVNLLQESLNNIR.T;R.LEIASSALHDLSLRPQGK.K;K.VLVDIGTGYFVEK.T;K.SNFDQLIELASK.K

PFF Mascot score: **[456]**   Sequence coverage %: **[40]**

Matched peptides No.: **[5]**

Calculated Mr: **17309**   Calculated *p*I: **8.59**

Data base searched result:

Ions score is -10*Log(P), where P is the probability that the observed match is a random event.
Individual ions scores > 30 indicate identity or extensive homology (p<0.05).
Protein scores are derived from ions scores as a non-probabilistic basis for ranking protein hits.

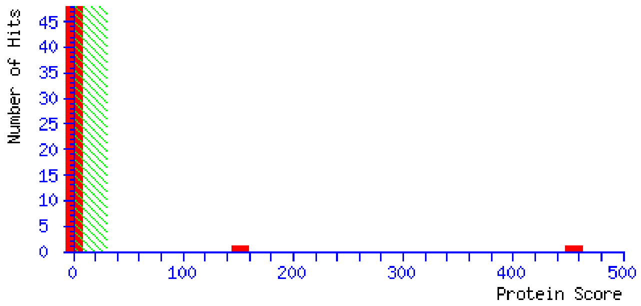

Matched peptide sequences: shown in Bold Red

```
  1 MASSKGGGGG GSSPVVRVAE MEKMSIEQLK ALKEQTDLEV NLLQESLNNI
 51 RTATTRLEIA SSALHDLSLR PQGKKMLVPL TASLYVPGKL DDANKVLVDI
101 GTGYFVEKTM AEGKEYCERK INLLKSNFDQ LIELASKKKT AADEAGAVLQ
151 AKLKQMVPAT
```

Matched peptide information:

Start - End	Observed	Mr(expt)	Mr(calc)	ppm	Miss	Sequence
31 - 51	2440.3213	2439.3140	2439.2969	7	1	K.ALKEQTDLEVNLLQESLNNIR.T (Ions score 114)
34 - 51	2128.1062	2127.0989	2127.0807	9	0	K.EQTDLEVNLLQESLNNIR.T (Ions score 186)
57 - 74	1935.0845	1934.0772	1934.0585	10	1	R.LEIASSALHDLSLRPQGK.K (Ions score 112)
96 - 108	1439.7760	1438.7687	1438.7708	-1	0	K.VLVDIGTGYFVEK.T (Ions score 98)
126 - 137	1364.7056	1363.6983	1363.6983	-0	0	K.SNFDQLIELASK.K (Ions score 61)

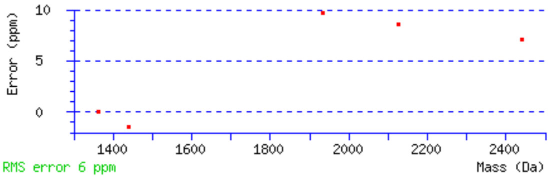

Spot No.:**63**

Accession No.: **scaffold4512_855.mRNA1**

Protein name：**Osmotin-like protein OSM34**

Peptide sequences: R.NNCPYTVWAAASPGGGR.R;K.APGGCNNPCTVFK.T

PFF Mascot score: **[112]** Sequence coverage %: **[12]**

Matched peptides No.: **[2]**

Calculated Mr: **27682** Calculated *p*I: **5.23**

Data base searched result:

Ions score is -10*Log(P), where P is the probability that the observed match is a random event.
Individual ions scores > 31 indicate identity or extensive homology (p<0.05).
Protein scores are derived from ions scores as a non-probabilistic basis for ranking protein hits.

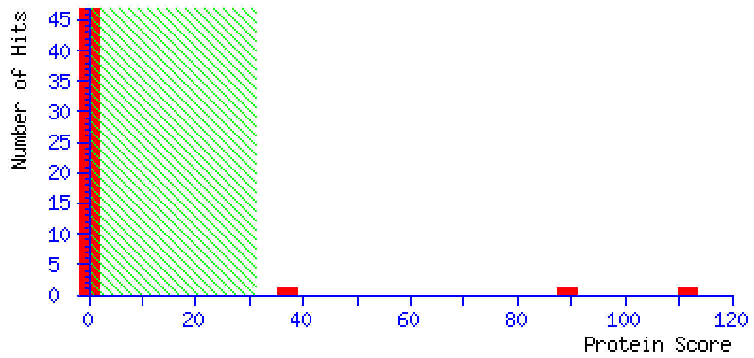

Matched peptide sequences: shown in Bold Red

```
  1 MSNFNIFLIS IFLLSALFFT SSDGATFTIR NNCPYTVWAA ASPGGGRRLD
 51 QGQTWELNVP AGTSMARIWG RTNCNFDGSG KGHCQTGDCG GILACQGWGV
101 PPNTLAEYAL NQFGNLDFYD ISLVDGFNIP IEFSPTSGAK DKCRPLFCTA
151 DINGQCPNQL KAPGGCNNPC TVFKTNEYCC TEGYGTCGPT EFSKFFKSRC
201 SDAYSYPQDD PSSTFTCPGG TNYRVVFCPA RSPHFPLEMV REKDVE
```

Matched peptide information:

Start - End	Observed	Mr(expt)	Mr(calc)	ppm	Miss	Sequence	
31 - 47	1777.8068	1776.7995	1776.8002	-0	0	R.NNCPYTVWAAASPGGGR.R	(Ions score 90)
162 - 174	1421.6248	1420.6175	1420.6228	-4	0	K.APGGCNNPCTVFK.T	(Ions score 47)

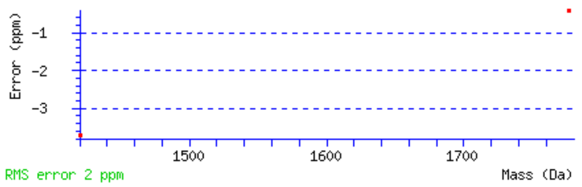

Spot No.:64

Accession No.: scaffold0114_231043.mRNA1

Protein name： Cysteine synthase

Peptide sequences:

K.TPLVYLNHVVDGCVAR.I;K.LIITMPASMSLER.R;K.VDALVSGIGTGGTVTGAGQYLK.E;K.LYGVEPVESAVLSGGKPGPHK.I;K.EGLLAGISSGAAAAAAIR.I;K.LIVVVFPSFGER.Y;R.YLSSVLFESVK.H

PFF Mascot score: [585] Sequence coverage %: [16]

Matched peptides No.: [7]

Calculated Mr: 75280 Calculated *p*I: 6.22

Data base searched result:

Ions score is -10*Log(P), where P is the probability that the observed match is a random event.
Individual ions scores > 31 indicate identity or extensive homology (p<0.05).
Protein scores are derived from ions scores as a non-probabilistic basis for ranking protein hits.

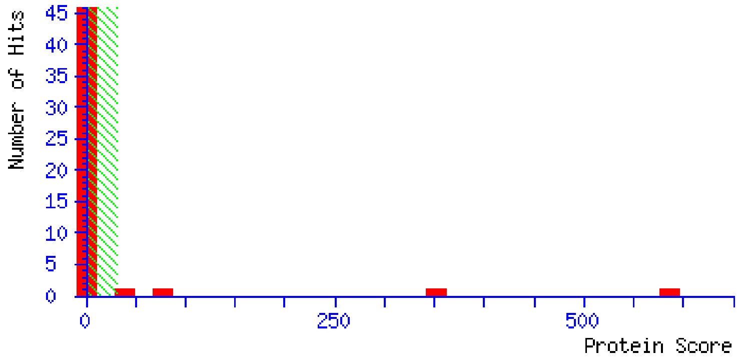

Matched peptide sequences: shown in Bold Red

1 MLQQRYIISY KLELINIAVL LQSHPQLLVG NGLQATCRTL APPWLMLARS
51 RSSPGSIKND CRSQRETGWN RTRLSLKMAE EKIAIAKDVT ELIGK**TPLVY**
101 **LNHVVDGCVA R**IAAKLELME PCSSVKDRIG YSMIADAEEK GLIKPGESVL
151 IEPTSGNTGI GLAFMAAAKG YK**LIITMPAS MSLER**RMVLR AFGAELVLTD
201 PARGMKGAVQ KAEEILAKTP NSYILQQFEN PANPKIHYET TGPEIWKGSG
251 GK**VDALVSGI GTGGTVTGAG QYLK**EQNPDI K**LYGVEPVES AVLSGGKPGP**
301 **HK**IQGIGAGF IPGVLDVGIL DEVVQISSEE SIETAKLLAL K**EGLLAGISS**
351 **GAAAAAAIR**I AKRPENAGK**L IVVVFPSFGE RYLSSVLFES VK**HEAENMTF
401 DGNTGIALED CEARIAAKLE MMEPCFSVKD RIAHSMIKDA EEKGLIIPGK
451 TILIEPTSGN TGIGLASIAA VKGYKLMLTM PASMSLERRI VLRALGAEVH
501 LTDPAMGFNG VLQKTDELLS KTPNGHMLRQ FDNPANPKIH YETTGPEIWR
551 DSGGKVDALV AGIGTGGTVT GAGKFLKEKN SEIKVYGVEP VESAVLNGRN
601 PGPHLIQGIG AGVIPSVLDI DLLDEVLQVS SEEAIETAQL LALKEGLLVG
651 ISSGAAVAAA IKLAKRPENA GKLIAVILPS FGERYLSTKL FDSVRHEVEN
701 MTID

Matched peptide information:

Start - End	Observed	Mr(expt)	Mr(calc)	ppm	Miss	Sequence
96 - 111	1812.9362	1811.9289	1811.9353	-4	0	K.TPLVYLNHVVDGCVAR.I (Ions score 101)
173 - 185	1461.7715	1460.7642	1460.7731	-6	0	K.LIITMPASMSLER.R (Ions score 82)
253 - 274	2064.0872	2063.0799	2063.0899	-5	0	K.VDALVSGIGTGGTVTGAGQYLK.E (Ions score 153)
282 - 302	2121.1257	2120.1185	2120.1266	-4	1	K.LYGVEPVESAVLSGGKPGPHK.I (Ions score 119)
342 - 359	1598.8802	1597.8730	1597.8787	-4	0	K.EGLLAGISSGAAAAAAIR.I (Ions score 144)
370 - 381	1362.7637	1361.7564	1361.7707	-11	0	K.LIVVVFPSFGER.Y (Ions score 78)
382 - 392	1271.6735	1270.6662	1270.6809	-12	0	R.YLSSVLFESVK.H (Ions score 87)

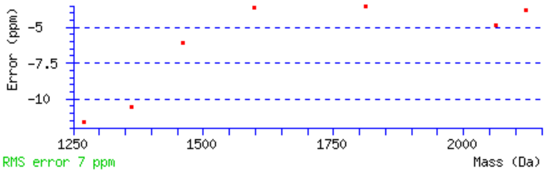

Spot No.:**65**

Accession No.: **scaffold0625_1132.mRNA1**

Protein name： **Glucan endo-1,3-beta-glucosidase, basic vacuolar isoform**

Peptide sequences:

R.IYDPNQAVLEALR.G;R.YIAVGNEISPVNGGTAWLAQFVLPAMR.N;K.VSTAIDLTLVGNSYPPSAGAFR.D;R.SYLDPIIGFLSSIR.S;R.DISLPYALFTSPSVVVWDGQR.G;K.NLFDATLDALYSALER.A;K.HFGLFFPDK.R

PFF Mascot score: **[744]** Sequence coverage %: **[32]**

Matched peptides No.: **[7]**

Calculated Mr: **41184** Calculated *p*I: **7.77**

Data base searched result:

Ions score is -10*Log(P), where P is the probability that the observed match is a random event.
Individual ions scores > 31 indicate identity or extensive homology (p<0.05).
Protein scores are derived from ions scores as a non-probabilistic basis for ranking protein hits.

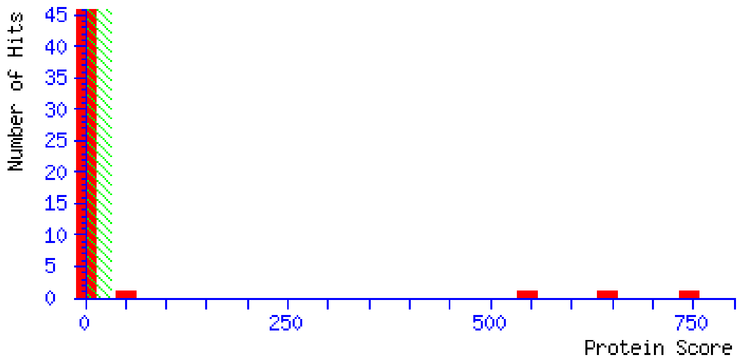

Matched peptide sequences: shown in Bold Red

```
  1 MAISSSTSGT SSSLPSRTTV MLLLIFFTAS LGITDAQVGV CYGMQGNNLP
 51 SVSEVIALYK QSNIKRMRIY DPNQAVLEAL RGSNIELILG VPNSDLQSLT
101 NPSNANSWVQ KNVRGFWSSV RFRYIAVGNE ISPVNGGTAW LAQFVLPAMR
151 NIHDAIRSAG LQDQIKVSTA IDLTLVGNSY PPSAGAFRDD VRSYLDPIIG
201 FLSSIRSPLL ANIYPYFTYA DNPRDISLPY ALFTSPSVVV WDGQRGYKNL
251 FDATLDALYS ALERASGGSL EVVVSESGWP SAGAFAATFD NGRTYLSNLI
301 QHVKGGTPKR PNRAIETYLF AMFDENKKQP EVEKHFGLFF PDKRPKYNLN
351 FGAEKNWDIS TEHNATILFL KSDM
```

Matched peptide information:

Start - End	Observed	Mr(expt)	Mr(calc)	ppm	Miss	Sequence
69 - 81	1501.8092	1500.8019	1500.7936	6	0	R.IYDPNQAVLEALR.G (Ions score 110)
124 - 150	2874.4941	2873.4869	2873.4898	-1	0	R.YIAVGNEISPVNGGTAWLAQFVLPAMR.N (Ions score 130)
167 - 188	2236.1765	2235.1692	2235.1535	7	0	K.VSTAIDLTLVGNSYPPSAGAFR.D (Ions score 196)
193 - 206	1580.8752	1579.8680	1579.8610	4	0	R.SYLDPIIGFLSSIR.S (Ions score 114)
225 - 245	2350.2212	2349.2139	2349.2005	6	0	R.DISLPYALFTSPSVVVWDGQR.G (Ions score 168)
249 - 264	1811.9318	1810.9245	1810.9101	8	0	K.NLFDATLDALYSALER.A (Ions score 139)
335 - 343	1107.5656	1106.5583	1106.5549	3	0	K.HFGLFFPDK.R (Ions score 68)

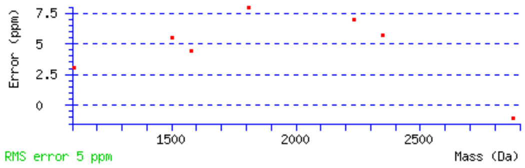

Spot No.:**66**

Accession No.: **scaffold0340_174387.mRNA1**

Protein name：**Malignant T-cell-amplified sequence 1**

Peptide sequences:

K.KFSYEDVSSQNQVK.A;K.FSYEDVSSQNQVK.A;R.QSIADEYPGLEPVLEDLLPK.K;K.CQNHLNLVMVNNVPLFFNVR.D;R.DGPYMPTLR.L;R.LLHQYPNIMK.K;K.GIGVDNMHYLNDGLWK.M

PFF Mascot score: **[341]** Sequence coverage %: **[49]**

Matched peptides No.: **[7]**

Calculated Mr: **20282** Calculated *p*I: **8.46**

Data base searched result:

Ions score is -10*Log(P), where P is the probability that the observed match is a random event.
Individual ions scores > 30 indicate identity or extensive homology (p<0.05).
Protein scores are derived from ions scores as a non-probabilistic basis for ranking protein hits.

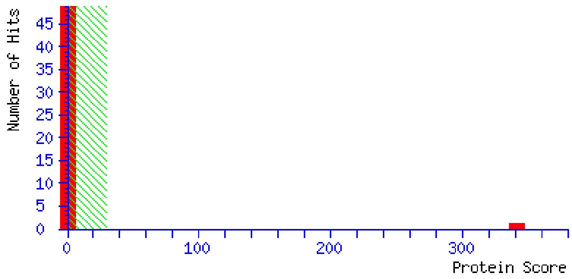

Matched peptide sequences: shown in Bold Red

```
  1 MFKKFSYEDV SSQNQVKASV QRKIRQSIAD EYPGLEPVLE DLLPKKSPLI
 51 VAKCQNHLNL VMVNNVPLFF NVRDGPYMPT LRLLHQYPNI MKKLQVDRGA
101 IKFVLAGANI MCPGLTSPGG ALDEEVEAET PVAIMAEGKQ HALAIGFTKM
151 SAKDIKAINK GIGVDNMHYL NDGLWKMERL D
```

Matched peptide information:

Start - End	Observed	Mr(expt)	Mr(calc)	ppm	Miss	Sequence
4 - 17	1658.8019	1657.7946	1657.7948	-0	1	K.KFSYEDVSSQNQVK.A (Ions score 63)
5 - 17	1530.7062	1529.6989	1529.6998	-1	0	K.FSYEDVSSQNQVK.A (Ions score 96)
26 - 45	2226.1470	2225.1397	2225.1467	-3	0	R.QSIADEYPGLEPVLEDLLPK.K (Ions score 71)
54 - 73	2428.2378	2427.2305	2427.2304	0	0	K.CQNHLNLVMVNNVPLFFNVR.D (Ions score 87)
74 - 82	1049.5057	1048.4985	1048.5012	-3	0	R.DGPYMPTLR.L (Ions score 48)
83 - 92	1256.6760	1255.6688	1255.6747	-5	0	R.LLHQYPNIMK.K (Ions score 59)
161 - 176	1831.8823	1830.8750	1830.8723	1	0	K.GIGVDNMHYLNDGLWK.M (Ions score 95)

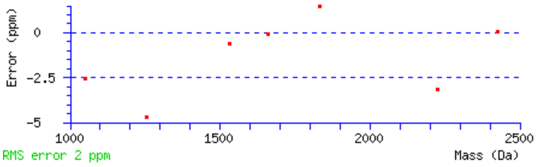

Spot No.:**67**

Accession No.: **scaffold0197_1486649.mRNA1**

Protein name：**Uncharacterized protein Os08g0359500**

Peptide sequences:

K.VFITVLLPDAK.N;K.VNLEPEGVFTFSASAGAGDNLYELK.L;R.SIFCILEK.A;R.GDGKPPHYLK.V

PFF Mascot score: **[235]** Sequence coverage %: **[29]**

Matched peptides No.: **[4]**

Calculated Mr: **20458** Calculated *p*I: **4.48**

Data base searched result:

Ions score is -10*Log(P), where P is the probability that the observed match is a random event.
Individual ions scores > 31 indicate identity or extensive homology (p<0.05).
Protein scores are derived from ions scores as a non-probabilistic basis for ranking protein hits.

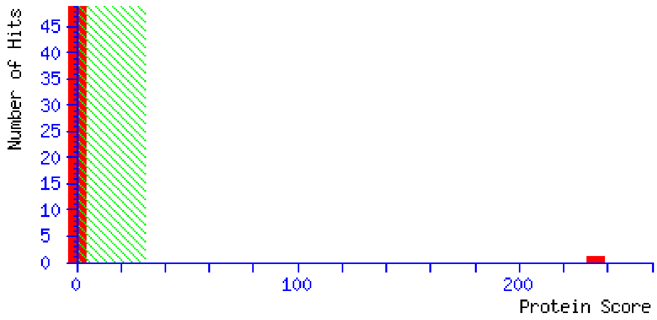

Matched peptide sequences: shown in Bold Red

1 MSRHPEVKWA QRVDK**VFITV** **LLPDAK**NAKV **NLEPEGVFTF** **SASAGAGDNL**
51 **YELK**LELHDK VNVEESKINI GVR**SIFCILE** **K**AEKGWWKKL LR**GDGKPPHY**
101 **LK**VDWDKWVD EDEDDGGLGN NFDMGGMDFS NFGDMGGMGD DGMGEFDDSD
151 DEDQEVAKPE KAEGAAKTGE EEKLEEKKDA APSS

Matched peptide information:

Start - End	Observed	Mr(expt)	Mr(calc)	ppm	Miss	Sequence
16 - 26	1215.7223	1214.7150	1214.7275	-10	0	K.VFITVLLPDAK.N (Ions score 59)
30 - 54	2628.2825	2627.2752	2627.2755	-0	0	K.VNLEPEGVFTFSASAGAGDNLYELK.L (Ions score 173)
74 - 81	1009.5311	1008.5238	1008.5314	-8	0	R.SIFCILEK.A (Ions score 45)
93 - 102	1111.5875	1110.5802	1110.5822	-2	1	R.GDGKPPHYLK.V (Ions score 47)

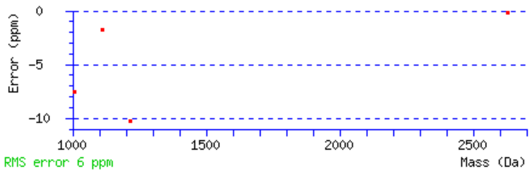

Spot No.:**68**

Accession No.: **scaffold0625_591792.mRNA1**

Protein name：**Translationally-controlled tumor protein homolog**

Peptide sequences:

R.MLVYQDLLTGDELLSDSFPYK.E;K.EIHNGILWEVEGK.W;K.WVVQGAVDVDIGANPSAEGADEDEGVDDQAVK.V;K.VVDIVDTFR.L; R.LQEQPAFDKK.Q;K.LSDLQFFVGESMHDDGSLVFAYYR.E;

PFF Mascot score: **[505]**

Sequence coverage %: **[64]**

Matched peptides No.: **[6]**

Calculated Mr: **19156**

Calculated *p*I: **4.47**

Data base searched result:

Ions score is -10*Log(P), where P is the probability that the observed match is a random event.
Individual ions scores > 31 indicate identity or extensive homology (p<0.05).
Protein scores are derived from ions scores as a non-probabilistic basis for ranking protein hits.

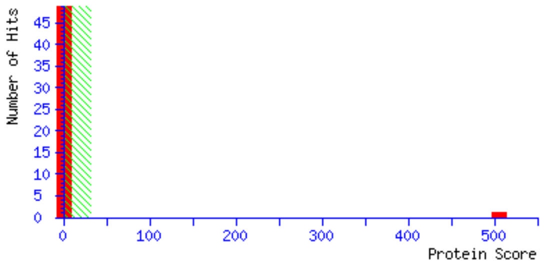

Matched peptide sequences: shown in Bold Red

```
  1 MLVYQDLLTG DELLSDSFPY KEIHNGILWE VEGKWVVQGA VDVDIGANPS
 51 AEGADEDEGV DDQAVKVVDI VDTFRLQEQP AFDKKQFVTY MKRFIKLLTP
101 KLDEEKQESF KKNIEGATKF LLSKLSDLQF FVGESMHDDG SLVFAYYREG
151 ATDPTFLYFA YALKEVKC
```

Matched peptide information:

Start - End	Observed	Mr(expt)	Mr(calc)	ppm	Miss	Sequence
1 - 21	2447.1821	2446.1749	2446.1978	-9	0	-.MLVYQDLLTGDELLSDSFPYK.E (Ions score 145)
22 - 34	1523.7560	1522.7487	1522.7780	-19	0	K.EIHNGILWEVEGK.W (Ions score 111)
35 - 66	3255.4526	3254.4454	3254.4852	-12	0	K.WVVQGAVDVDIGANPSAEGADEDEGVDDQAVK.V (Ions score 136)
67 - 75	1063.5508	1062.5435	1062.5710	-26	0	K.VVDIVDTFR.L (Ions score 76)
76 - 85	1203.6078	1202.6005	1202.6295	-24	1	R.LQEQPAFDKK.Q (Ions score 50)
125 - 148	2796.2681	2795.2608	2795.2901	-10	0	K.LSDLQFFVGESMHDDGSLVFAYYR.E (Ions score 136)

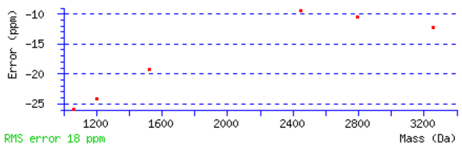

Spot No.:69

Accession No.: **scaffold0387_85080.mRNA1**

Protein name： **Macro domain-containing protein VPA0103**

Peptide sequences:

K.MLGGGGADGAIHR.A;R.AAGPELLDACYR.V;R.VPEVQPGVR.C;K.LPASHVIHTVGPIYDTDR.G

PFF Mascot score: **[170]** Sequence coverage %: **[19]**

Matched peptides No.: **[4]**

Calculated Mr: **29500** Calculated *p*I: **7.67**

Data base searched result:

Ions score is -10*Log(P), where P is the probability that the observed match is a random event.
Individual ions scores > 31 indicate identity or extensive homology (p<0.05).
Protein scores are derived from ions scores as a non-probabilistic basis for ranking protein hits.

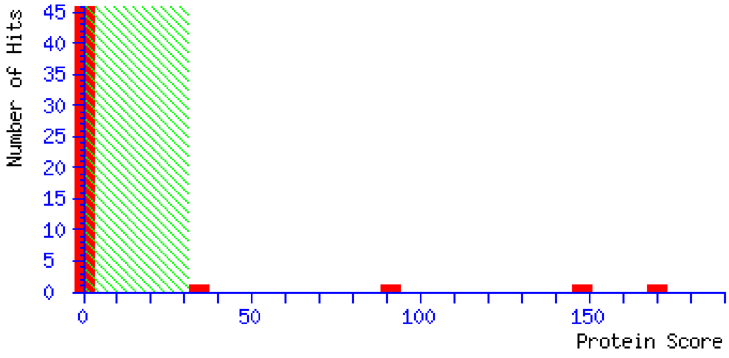

Matched peptide sequences: shown in Bold Red

1	MTALCTGILL	VGSRLSTPQF	LVAASPHSSL	SLSSNNAYLV	KRSLHYCYHR
51	LLKKHLPLTS	PSSIMAQIES	YAGVSTAPSF	DDGGDNITVF	PLSSSSVLKI
101	NKGDITKWFV	DGSSDAIVNP	ANEK**MLGGGG**	**ADGAIHRAAG**	**PELLDACYRV**
151	**PEVQPGVR**CP	TGEARITLGF	K**LPASHVIHT**	**VGPIYDTDR**G	PASSLKNAYR
201	NSLTVAKDNN	IKYIAFPALS	CGVYGYPYEE	AAAVATSTVK	EFADDLKEIH
251	FVLFSDDIYN	IWLKKAKQLL	LP		

Matched peptide information:

Start - End	Observed	Mr(expt)	Mr(calc)	ppm	Miss	Sequence
125 - 137	1211.6077	1210.6004	1210.5877	10	0	K.MLGGGGADGAIHR.A (Ions score 22)
138 - 149	1335.6429	1334.6357	1334.6289	5	0	R.AAGPELLDACYR.V (Ions score 75)
150 - 158	980.5494	979.5422	979.5451	-3	0	R.VPEVQPGVR.C (Ions score 33)
172 - 189	1991.0464	1990.0391	1990.0272	6	0	K.LPASHVIHTVGPIYDTDR.G (Ions score 121)

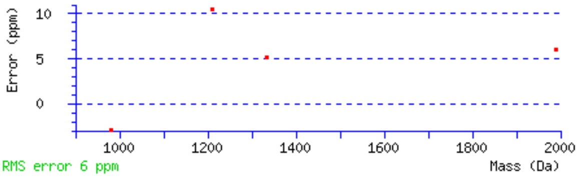

RMS error 6 ppm

Spot No.:**70**

Accession No.: **scaffold1772_2818.mRNA1**

Protein name： **universal stress protein PHOS32**

Peptide sequences:

R.EVTLPSLIPVVPKPELER.E;R.DILIAIDHGPNSK.H;K.HAFDWALIHLCR.L;K.NDVVYEMAQGLMEK.L;K.LAVEAFQVVMVK.S;R.GIVQSVLQGSVSEYCFHHCK.A

PFF Mascot score: **[470]** Sequence coverage %: **[45]**

Matched peptides No.: **[6]**

Calculated Mr: **22504** Calculated *p*I: **7.70**

Data base searched result:

Ions score is -10*Log(P), where P is the probability that the observed match is a random event.
Individual ions scores > 31 indicate identity or extensive homology (p<0.05).
Protein scores are derived from ions scores as a non-probabilistic basis for ranking protein hits.

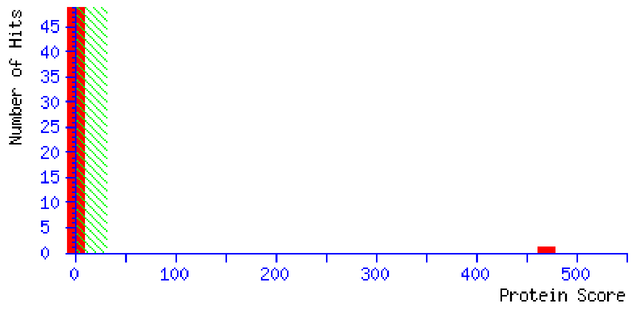

Matched peptide sequences: shown in Bold Red

```
  1 MEVLNEEEEY NWREVTLPSL IPVVPKPELE RESGERRRGR DILIAIDHGP
 51 NSKHAFDWAL IHLCRLADTI HLVHAVSSVK NDVVYEMAQG LMEKLAVEAF
101 QVVMVKSVAR IVEGDAGKVI CKEAERLRPA AVVMGTRGRG IVQSVLQGSV
151 SEYCFHHCKA APVIIVPGKR FIEYKLYCEF GITTINTHRY KYTYKI
```

Matched peptide information:

Start - End	Observed	Mr(expt)	Mr(calc)	ppm	Miss	Sequence
14 - 31	2016.1592	2015.1519	2015.1667	-7	1	R.EVTLPSLIPVVPKPELER.E (Ions score 140)
41 - 53	1392.7228	1391.7155	1391.7409	-18	0	R.DILIAIDHGPNSK.H (Ions score 81)
54 - 65	1538.7526	1537.7453	1537.7612	-10	0	K.HAFDWALIHLCR.L (Ions score 99)
81 - 94	1626.7255	1625.7182	1625.7429	-15	0	K.NDVVYEMAQGLMEK.L (Ions score 94)
95 - 106	1333.7242	1332.7170	1332.7475	-23	0	K.LAVEAFQVVMVK.S (Ions score 76)
140 - 159	2335.0727	2334.0655	2334.0885	-10	0	R.GIVQSVLQGSVSEYCFHHCK.A (Ions score 131)

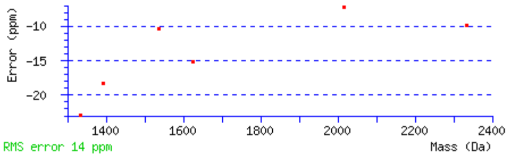

Spot No.:71

Accession No.: scaffold0005_28735.mRNA1

Protein name：S-adenosylmethionine synthase 2

Peptide sequences:

K.NGTCPWLRPDGK.T;K.TQVTVEYYNDNGAMVPVR.V;K.TIFHLNPSGR.F;R.FVIGGPHGDAGLTGR.K;K.ENFDFRPGMISINLDLK.R

PFF Mascot score: [182]   Sequence coverage %: [18]

Matched peptides No.: [5]

Calculated Mr: 43540   Calculated *p*I: 5.59

Data base searched result:

Ions score is -10*Log(P), where P is the probability that the observed match is a random event.
Individual ions scores > 31 indicate identity or extensive homology (p<0.05).
Protein scores are derived from ions scores as a non-probabilistic basis for ranking protein hits.

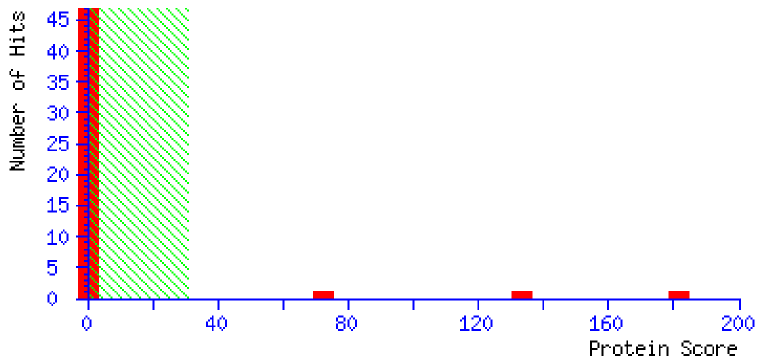

Matched peptide sequences: shown in Bold Red

```
  1 METFLYTSES VNEGHPDKLC DQVSDAILDA CLEQDPDSKV ACETCTKTNM
 51 VMVFGEITTK ANVDYEKIVR DTCRSIGFVS DDVGLDADKC KVLVYIEQQS
101 PDIAQGVHGH LTKRPEEIGA GDQGHMFGYA TDETPELMPL SHVLATKLGA
151 RLTEVRKNGT CPWLRPDGKT QVTVEYYNDN GAMVPVRVHT VLISTQHDET
201 VTNDEIAADL KEHVIKPVIP EKYLDEKTIF HLNPSGRFVI GGPHGDAGLT
251 GRKIIIDTYG GWGAHGGGAF SGKDPTKVDR SGAYIVRQAA KSIVASGLAR
301 RCIVQVSYAI GVPEPLSVFV DTYGTGKIPD KEILKIVKEN FDFRPGMISI
351 NLDLKRGGNG RFLKTAAYGH FGRDDADFTW EVVKPLKWEK PQA
```

Matched peptide information:

Start - End	Observed	Mr(expt)	Mr(calc)	ppm	Miss	Sequence
158 - 169	1400.6628	1399.6556	1399.6667	-8	1	K.NGTCPWLRPDGK.T (Ions score 37)
170 - 187	2055.9673	2054.9600	2054.9731	-6	0	K.TQVTVEYYNDNGAMVPVR.V (Ions score 78)
228 - 237	1141.6018	1140.5945	1140.6040	-8	0	K.TIFHLNPSGR.F (Ions score 47)
238 - 252	1453.7457	1452.7385	1452.7474	-6	0	R.FVIGGPHGDAGLTGR.K (Ions score 106)
339 - 355	2008.9985	2007.9913	2008.0088	-9	1	K.ENFDFRPGMISINLDLK.R (Ions score 29)

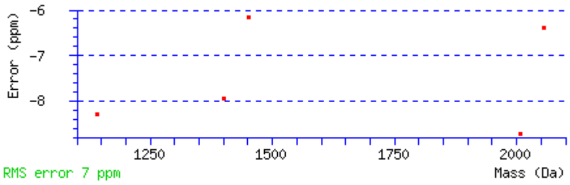

Spot No.:**72**

Accession No.: **scaffold0197_1256978.mRNA1**

Protein name：**Glutathione S-transferase**

Peptide sequences:

K.ELEFEFVNVNMGAGEHK.K;R.AITQYITHENPDK.G;R.AITQYITHENPDKGTQLLCTGK.Q;K.LANVLDVYESR.L;K.YLAGDSFTLADLHHLPNLHLLLVTQSK.K;R.PHVSAWAADITAR.P

PFF Mascot score: **[259]** Sequence coverage %: **[42]**

Matched peptides No.: **[6]**

Calculated Mr: **23988** Calculated *p*I: **5.81**

Data base searched result:

Ions score is -10*Log(P), where P is the probability that the observed match is a random event.
Individual ions scores > 31 indicate identity or extensive homology (p<0.05).
Protein scores are derived from ions scores as a non-probabilistic basis for ranking protein hits.

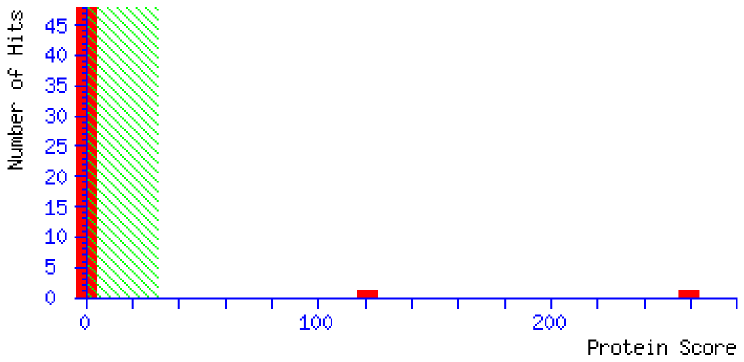

Matched peptide sequences: shown in Bold Red

```
  1 MMATIKVHGS PFSTATQRVL VSLYEKELEF EFVNVNMGAG EHKKEPFISL
 51 NPFGQVPALE DGDLKLFESR AITQYITHEN PDKGTQLLCT GKQMAIVSVW
101 MEVEAQKFEP AASKLNWEVF YKPFFGLTTD PAAVEENEAK LANVLDVYES
151 RLAQSKYLAG DSFTLADLHH LPNLHLLLVT QSKKLIDSRP HVSAWAADIT
201 ARPAWAKVLA MQKN
```

Matched peptide information:

Start	-	End	Observed	Mr(expt)	Mr(calc)	ppm	Miss	Sequence
27	-	43	1949.8723	1948.8650	1948.8989	-17	0	K.ELEFEFVNVNMGAGEHK.K (Ions score 108)
71	-	83	1529.7294	1528.7221	1528.7522	-20	0	R.AITQYITHENPDK.G (Ions score 56)
71	-	92	2488.2136	2487.2063	2487.2428	-15	1	R.AITQYITHENPDKGTQLLCTGK.Q (Ions score 28)
141	-	151	1278.6521	1277.6448	1277.6615	-13	0	K.LANVLDVYESR.L (Ions score 78)
157	-	183	3016.5554	3015.5481	3015.6182	-23	0	K.YLAGDSFTLADLHHLPNLHLLLVTQSK.K (Ions score 44)
190	-	202	1394.6976	1393.6904	1393.7102	-14	0	R.PHVSAWAADITAR.P (Ions score 78)

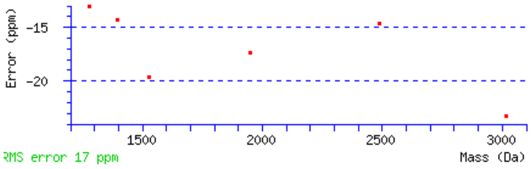

Spot No.:**73**

Accession No.: **scaffold0143_850373.mRNA1**

Protein name：**Hevamine-A**

Peptide sequences:

K.FGNGQTPQINLAGHCNPAAGGCTIVSNGIR.S;K.SSSRPLGDAVLDGIDFDIEHGSTLYWDDLAR.Y;R.PLGDAVLDGIDFDIEHGSTLYWDDLAR.Y;K.KVYLTAAPQCPFPDR.Y;K.VYLTAAPQCPFPDR.Y;K.IFLGLPAAPEAAGSGYVPPDVLISR.I

PFF Mascot score: **[521]** Sequence coverage %: **[32]**

Matched peptides No.: **[6]**

Calculated Mr: **34013** Calculated *p*I: **8.07**

Data base searched result:

Ions score is -10*Log(P), where P is the probability that the observed match is a random event.
Individual ions scores > 30 indicate identity or extensive homology (p<0.05).
Protein scores are derived from ions scores as a non-probabilistic basis for ranking protein hits.

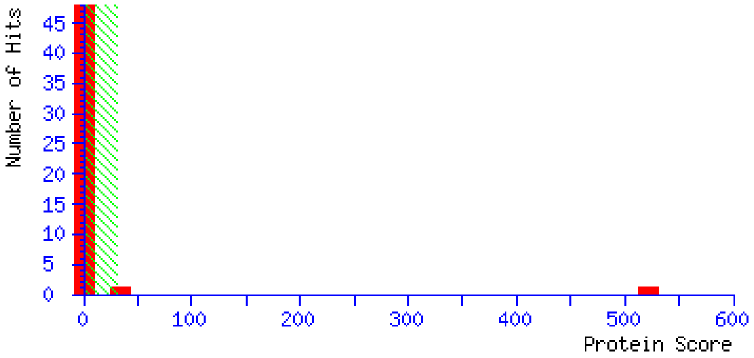

Matched peptide sequences: shown in Bold Red

```
  1 MATRTQAILL LLLAISLIMS SSHVDGGGIA IYWGQNGNEG TLTETCSTGK
 51 YSYVNIAFLN KFGNGQTPQI NLAGHCNPAA GGCTIVSNGI RSCQIQGIKV
101 MLSLGGGIGS YTLASQADAK NVADYLWNNF LGGKSSSRPL GDAVLDGIDF
151 DIEHGSTLYW DDLARYLSAY SKQGKKVYLT AAPQCPFPDR YLGTALNTGL
201 FDYVWVQFYN NPPCQYSSGN INNIINSWNR WTTSINAGKI FLGLPAAPEA
251 AGSGYVPPDV LISRILPEIK KSPKYGGVML WSKFYDDKNG YSSSIRDSVL
301 FLHSEKCMTV L
```

Matched peptide information:

Start - End	Observed	Mr(expt)	Mr(calc)	ppm	Miss	Sequence
62 - 91	3082.3796	3081.3724	3080.4669	294	0	K.FGNGQTPQINLAGHCNPAAGGCTIVSNGIR.S (Ions score 137)
135 - 165	3420.5325	3419.5252	3419.6270	-30	1	K.SSSRPLGDAVLDGIDFDIEHGSTLYWDDLAR.Y (Ions score 55)
139 - 165	3003.3601	3002.3528	3002.4298	-26	0	R.PLGDAVLDGIDFDIEHGSTLYWDDLAR.Y (Ions score 171)
176 - 190	1762.8639	1761.8566	1761.8872	-17	1	K.KVYLTAAPQCPFPDR.Y (Ions score 56)
177 - 190	1634.7706	1633.7634	1633.7923	-18	0	K.VYLTAAPQCPFPDR.Y (Ions score 80)
240 - 264	2510.3057	2509.2984	2509.3580	-24	0	K.IFLGLPAAPEAAGSGYVPPDVLISR.I (Ions score 152)

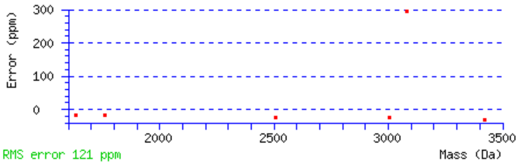

Spot No.:74

Accession No.: **scaffold0625_1132.mRNA1**

Protein name：**Glucan endo-1,3-beta-glucosidase, basic vacuolar isoform**

Peptide sequences:

R.IYDPNQAVLEALR.G;K.VSTAIDLTLVGNSYPPSAGAFR.D;R.SYLDPIIGFLSSIR.S;R.SPLLANIYPYFTYADNPR.D;R.DISLPYALFTSPSVVVWDGQR.G;K.NLFDATLDALYSALER.A;R.ASGGSLEVVVSESGWPSAGAFAATFDNGR.T

PFF Mascot score: **[733]** Sequence coverage %: **[35]**

Matched peptides No.: **[7]**

Calculated Mr: **41184** Calculated *p*I: **7.77**

Data base searched result:

Ions score is -10*Log(P), where P is the probability that the observed match is a random event.
Individual ions scores > 30 indicate identity or extensive homology (p<0.05).
Protein scores are derived from ions scores as a non-probabilistic basis for ranking protein hits.

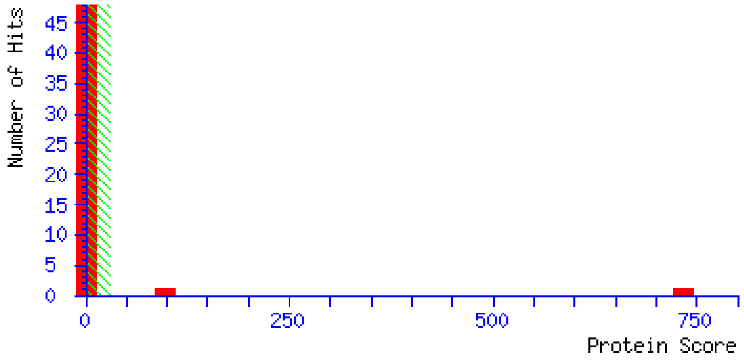

Matched peptide sequences: shown in Bold Red

```
  1 MAISSSTSGT SSSLPSRTTV MLLLIFFTAS LGITDAQVGV CYGMQGNNLP
 51 SVSEVIALYK QSNIKRMRIY DPNQAVLEAL RGSNIELILG VPNSDLQSLT
101 NPSNANSWVQ KNVRGFWSSV RFRYIAVGNE ISPVNGGTAW LAQFVLPAMR
151 NIHDAIRSAG LQDQIKVSTA IDLTLVGNSY PPSAGAFRDD VRSYLDPIIG
201 FLSSIRSPLL ANIYPYFTYA DNPRDISLPY ALFTSPSVVV WDGQRGYKNL
251 FDATLDALYS ALERASGGSL EVVVSESGWP SAGAFAATFD NGRTYLSNLI
301 QHVKGGTPKR PNRAIETYLF AMFDENKKQP EVEKHFGLFF PDKRPKYNLN
351 FGAEKNWDIS TEHNATILFL KSDM
```

Matched peptide information:

Start	-	End	Observed	Mr(expt)	Mr(calc)	ppm	Miss	Sequence
69	-	81	1501.7556	1500.7483	1500.7936	-30	0	R.IYDPNQAVLEALR.G (Ions score 86)
167	-	188	2236.1011	2235.0938	2235.1535	-27	0	K.VSTAIDLTLVGNSYPPSAGAFR.D (Ions score 184)
193	-	206	1580.8209	1579.8136	1579.8610	-30	0	R.SYLDPIIGFLSSIR.S (Ions score 93)
207	-	224	2114.9966	2113.9893	2114.0473	-27	0	R.SPLLANIYPYFTYADNPR.D (Ions score 105)
225	-	245	2350.1440	2349.1368	2349.2005	-27	0	R.DISLPYALFTSPSVVVWDGQR.G (Ions score 126)
249	-	264	1811.8689	1810.8616	1810.9101	-27	0	K.NLFDATLDALYSALER.A (Ions score 158)
265	-	293	2826.2456	2825.2383	2825.3257	-31	0	R.ASGGSLEVVVSESGWPSAGAFAATFDNGR.T (Ions score 156)

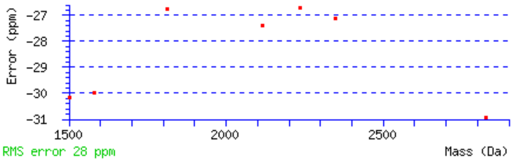

Spot No.:**75**

Accession No.: **scaffold0344_256841.mRNA1**

Protein name：**18.5 kDa class I heat shock protein**

Peptide sequences:

K.DFPFPSFASSSSLFPR.E;R.ENSAFVSTR.I;R.VLQISGER.H;K.EDKNDTWHR.V

PFF Mascot score: **[176]** Sequence coverage %: **[26]**

Matched peptides No.: **[4]**

Calculated Mr: **18064** Calculated *p*I: **5.97**

Data base searched result:

Ions score is -10*Log(P), where P is the probability that the observed match is a random event.
Individual ions scores > 31 indicate identity or extensive homology (p<0.05).
Protein scores are derived from ions scores as a non-probabilistic basis for ranking protein hits.

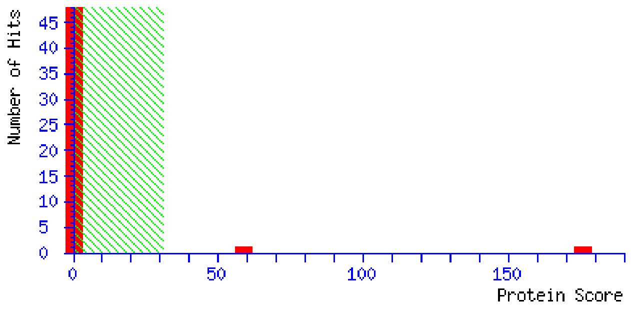

Matched peptide sequences: shown in Bold Red

```
  1 MSLTPFSGNR RSSIFDPFSL DIWDPFKDFP FPSFASSSSL FPRENSAFVS
 51 TRIDWKETPE AHVFKADLPG LRKEEVKVEI EDDRVLQISG ERHVEKEDKN
101 DTWHRVERSS GKFSRSFRLP ENTKMDQIKA SMENGVLTVT VPKAEVKKPD
151 VKAIEISG
```

Matched peptide information:

Start - End	Observed	Mr(expt)	Mr(calc)	ppm	Miss	Sequence
28 - 43	1788.7832	1787.7759	1787.8519	-42	0	K.DFPFPSFASSSSLFPR.E (Ions score 129)
44 - 52	1010.4426	1009.4354	1009.4829	-47	0	R.ENSAFVSTR.I (Ions score 53)
85 - 92	901.4721	900.4648	900.5029	-42	0	R.VLQISGER.H (Ions score 32)
97 - 105	1200.4703	1199.4631	1199.5319	-57	1	K.EDKNDTWHR.V (Ions score 55)

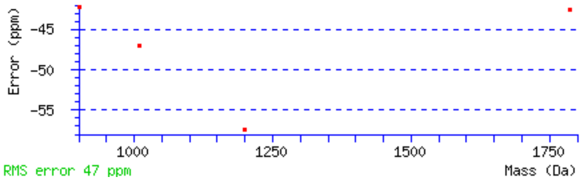

Spot No.:**76**

Accession No.: **scaffold0026_327123.mRNA1**

Protein name: **Chlorophyllase type 0**

Peptide sequences:

K.APASHFVTTDYGHMDMLDDNPTGILAIIANSICK.N;K.AYFQADSGDFMTILNEPSVAPAK.L

PFF Mascot score: **[182]** Sequence coverage %: **[16]**

Matched peptides No.: **[2]**

Calculated Mr: **37785** Calculated *p*I: **6.30**

Data base searched result:

Ions score is -10*Log(P), where P is the probability that the observed match is a random event.
Individual ions scores > 30 indicate identity or extensive homology (p<0.05).
Protein scores are derived from ions scores as a non-probabilistic basis for ranking protein hits.

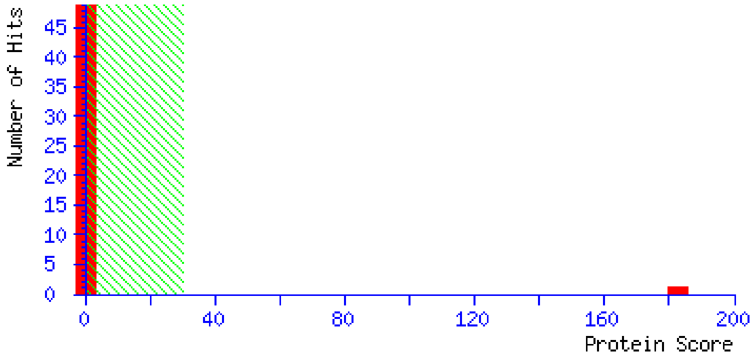

Matched peptide sequences: shown in Bold Red

```
  1 MLVTLLVILL ASALEAKPQF PTVVLLETKP VQDILDVFVT GSFPTKSIDV
 51 KKSNPASPPK PLLIVSPITD GTYPVFMFLH GTCLENYFYS NLLPHIASHG
101 FIVVAPQVYS CINWLIPKLP IRESKEIEFA AEVGNWLLSG LQSVLPEKVT
151 WDQDKLALGG HNRGGNIAFA LALGYSKTPL EVKISALVGL DPVGRVSTDP
201 KILTNVPHSF NLSIPVTVIG TGLGNESVCG VVGLACAPNY MNHVKFYNKC
251 KAPASHFVTT DYGHMDMLDD NPTGILAIIA NSICKNSKDP RDQMRRTVGG
301 LIVAFLKAYF QADSGDFMTI LNEPSVAPAK LDPVQFKEEQ NHAQV
```

Matched peptide information:

Start - End	Observed	Mr(expt)	Mr(calc)	ppm	Miss	Sequence
252 - 285	3688.5349	3687.5276	3687.7371	-57	0	K.APASHFVTTDYGHMDMLDDNPTGILAIIANSICK.N (Ions score 77)
308 - 330	2472.0693	2471.0621	2471.1679	-43	0	K.AYFQADSGDFMTILNEPSVAPAK.L (Ions score 128)

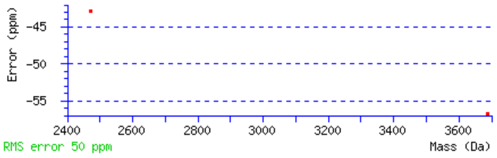

Spot No.:77

Accession No.: **scaffold0618_714426.mRNA1**

Protein name：**Beta-glucosidase 42**

Peptide sequences:

K.GTILDGSNGDVAVDHYHR.Y;K.DNQGGEVGLVVDCEWAEANSDKIEDK.A;R.NSLDFIGLNHYSSR.F;K.WEDGEPIGER.A;K.VLNYIVQR.Y

PFF Mascot score: **[263]** Sequence coverage %: **[15]**

Matched peptides No.: **[5]**

Calculated Mr: **56728** Calculated *p*I: **5.49**

Data base searched result:

Ions score is -10*Log(P), where P is the probability that the observed match is a random event.
Individual ions scores > 31 indicate identity or extensive homology (p<0.05).
Protein scores are derived from ions scores as a non-probabilistic basis for ranking protein hits.

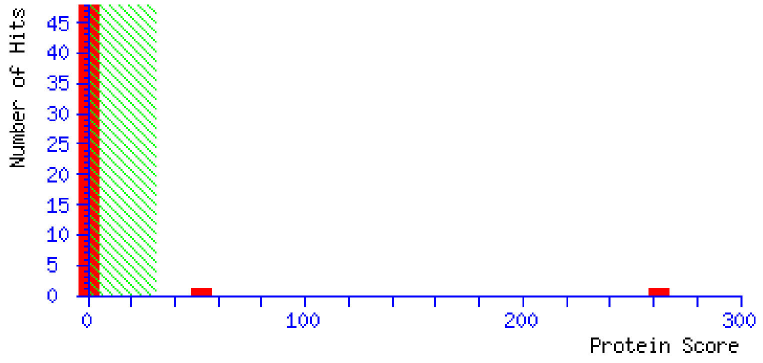

Matched peptide sequences: shown in Bold Red

```
  1 MIKKEKFLKE HPYLLEKEVS RSDFPPNFLF GVATSAYQIE GGCREGGRGP
 51 SIWDAFSHTK GTILDGSNGD VAVDHYHRYK EDIELIAKLG FDAYRFSLSW
101 SRIFPDGLGT KVNEEGIAFY NNIINALLEK GIEPYITLYH WDLPLHLQES
151 MGGWLNKEIV KYFAIYADTC FASFGDRVKK WITLNEPLQT AVNGFDTGIF
201 APGKHEQSDR EPFLASHHQI LAHATAVSIY RSMYKDNQGG EVGLVVDCEW
251 AEANSDKIED KAAAAKRLEF QLGWYLHPLY YGDYPEVMLM REILGDGLPK
301 FSEEDKELLR NSLDFIGLNH YSSRFIKHVT DGPAECYYYK AQEIERLAKW
351 EDGEPIGERA ASEWLYVCPW GLRKVLNYIV QRYNNPIIYV TENGMDDEDS
401 SAPLHEMLDD KLRVRYFKGY LAAVAQAIKD GADVRGYFAW SLLDNFEWAQ
451 GYTKRFGLIY VDYKNGLARH PKSSAYWFMR FLKGDEGKNG KE
```

Matched peptide information:

Start - End	Observed	Mr(expt)	Mr(calc)	ppm	Miss	Sequence
61 - 78	1925.8339	1924.8266	1924.9028	-40	0	K.GTILDGSNGDVAVDHYHR.Y (Ions score 78)
236 - 261	2877.1631	2876.1558	2876.2770	-42	1	K.DNQGGEVGLVVDCEWAEANSDKIEDK.A (Ions score 129)
311 - 324	1622.7273	1621.7200	1621.7849	-40	0	R.NSLDFIGLNHYSSR.F (Ions score 62)
350 - 359	1187.4805	1186.4732	1186.5255	-44	0	K.WEDGEPIGER.A (Ions score 75)
375 - 382	1004.5416	1003.5343	1003.5815	-47	0	K.VLNYIVQR.Y (Ions score 39)

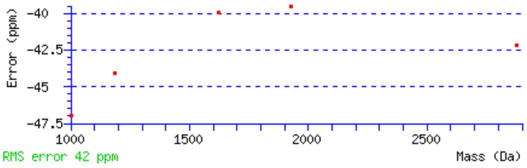

RMS error 42 ppm

Spot No.:78

Accession No.: scaffold0026_327123.mRNA1

Protein name：Chlorophyllase type 0

Peptide sequences:

K.APASHFVTTDYGHMDMLDDNPTGILAIIANSICK.N;R.TVGGLIVAFLK.A;K.AYFQADSGDFMTILNEPSVAPAK.L

PFF Mascot score: [158]

Sequence coverage %: [19]

Matched peptides No.: [3]

Calculated Mr: 37785

Calculated *p*I: 6.30

Data base searched result:

Ions score is -10*Log(P), where P is the probability that the observed match is a random event.
Individual ions scores > 30 indicate identity or extensive homology (p<0.05).
Protein scores are derived from ions scores as a non-probabilistic basis for ranking protein hits.

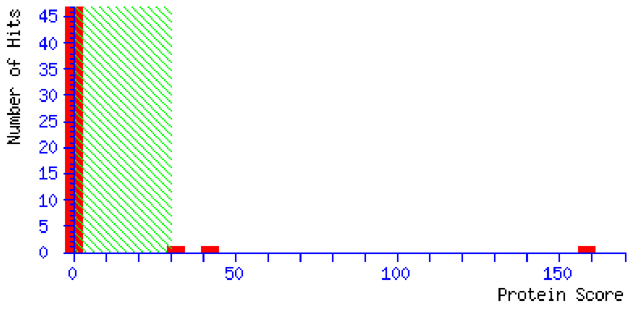

Matched peptide sequences: shown in Bold Red

```
  1 MLVTLLVILL ASALEAKPQF PTVVLLETKP VQDILDVFVT GSFPTKSIDV
 51 KKSNPASPPK PLLIVSPITD GTYPVFMFLH GTCLENYFYS NLLPHIASHG
101 FIVVAPQVYS CINWLIPKLP IRESKEIEFA AEVGNWLLSG LQSVLPEKVT
151 WDQDKLALGG HNRGGNIAFA LALGYSKTPL EVKISALVGL DPVGRVSTDP
201 KILTNVPHSF NLSIPVTVIG TGLGNESVCG VVGLACAPNY MNHVKFYNKC
251 KAPASHFVTT DYGHMDMLDD NPTGILAIIA NSICKNSKDP RDQMRRTVGG
301 LIVAFLKAYF QADSGDFMTI LNEPSVAPAK LDPVQFKEEQ NHAQV
```

Matched peptide information:

Start - End	Observed	Mr(expt)	Mr(calc)	ppm	Miss	Sequence
252 - 285	3688.5955	3687.5882	3687.7371	-40	0	K.APASHFVTTDYGHMDMLDDNPTGILAIIANSICK.N (Ions score 44)
297 - 307	1117.6459	1116.6386	1116.6907	-47	0	R.TVGGLIVAFLK.A (Ions score 66)
308 - 330	2472.0779	2471.0706	2471.1679	-39	0	K.AYFQADSGDFMTILNEPSVAPAK.L (Ions score 98)

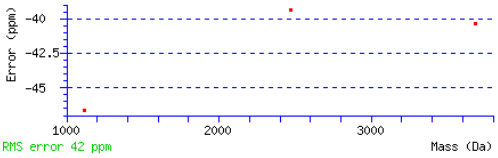

Spot No.:79

Accession No.: scaffold0143_850373.mRNA1

Protein name：Hevamine-A

Peptide sequences:

K.FGNGQTPQINLAGHCNPAAGGCTIVSNGIR.S;K.VMLSLGGGIGSYTLASQADAK.N;K.SSSRPLGDAVLDGIDFDIEHGSTLYWDDLAR.Y;R.PLGDAVLDGIDFDIEHGSTLYWDDLAR.Y;K.KVYLTAAPQCPFPDR.Y;K.VYLTAAPQCPFPDR.Y;K.IFLGLPAAPEAAGSGYVPPDVLISR.I;K.YGGVMLWSK.F

PFF Mascot score: [721] Sequence coverage %: [42]

Matched peptides No.: [8]

Calculated Mr: 34013 Calculated *p*I: 8.07

Data base searched result:

Ions score is -10*Log(P), where P is the probability that the observed match is a random event.
Individual ions scores > 30 indicate identity or extensive homology (p<0.05).
Protein scores are derived from ions scores as a non-probabilistic basis for ranking protein hits.

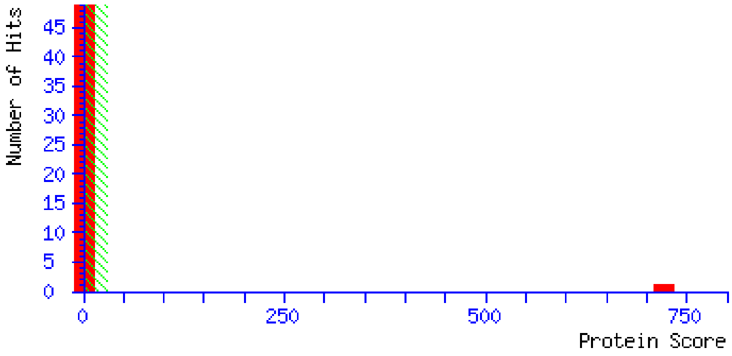

Matched peptide sequences: shown in **Bold Red**

```
  1 MATRTQAILL LLLAISLIMS SSHVDGGGIA IYWGQNGNEG TLTETCSTGK
 51 YSYVNIAFLN KFGNGQTPQI NLAGHCNPAA GGCTIVSNGI RSCQIQGIKV
101 MLSLGGGIGS YTLASQADAK NVADYLWNNF LGGKSSSRPL GDAVLDGIDF
151 DIEHGSTLYW DDLARYLSAY SKQGKKVYLT AAPQCPFPDR YLGTALNTGL
201 FDYVWVQFYN NPPCQYSSGN INNIINSWNR WTTSINAGKI FLGLPAAPEA
251 AGSGYVPPDV LISRILPEIK KSPKYGGVML WSKFYDDKNG YSSSIRDSVL
301 FLHSEKCMTV L
```

Matched peptide information:

Start – End	Observed	Mr(expt)	Mr(calc)	ppm	Miss	Sequence
62 – 91	3082.3567	3081.3494	3080.4669	286	0	K.FGNGQTPQINLAGHCNPAAGGCTIVSNGIR.S (Ions score 112)
100 – 120	2038.9880	2037.9808	2038.0405	-29	0	K.VMLSLGGGIGSYTLASQADAK.N (Ions score 101)
135 – 165	3420.5166	3419.5093	3419.6270	-34	1	K.SSSRPLGDAVLDGIDFDIEHGSTLYWDDLAR.Y (Ions score 87)
139 – 165	3003.3420	3002.3348	3002.4298	-32	0	R.PLGDAVLDGIDFDIEHGSTLYWDDLAR.Y (Ions score 170)
176 – 190	1762.8508	1761.8436	1761.8872	-25	1	K.KVYLTAAPQCPFPDR.Y (Ions score 85)
177 – 190	1634.7567	1633.7494	1633.7923	-26	0	K.VYLTAAPQCPFPDR.Y (Ions score 84)
240 – 264	2510.2920	2509.2847	2509.3580	-29	0	K.IFLGLPAAPEAAGSGYVPPDVLISR.I (Ions score 208)
275 – 283	1040.4850	1039.4777	1039.5161	-37	0	K.YGGVMLWSK.F (Ions score 65)

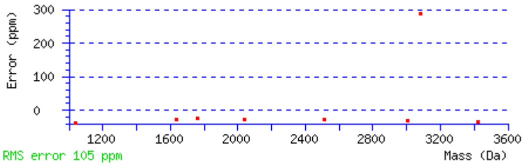

Spot No.:**80**

Accession No.: **scaffold0143_850373.mRNA1**

Protein name: **Hevamine-A**

Peptide sequences: **K.VYLTAAPQCPFPDR.Y;K.IFLGLPAAPEAAGSGYVPPDVLISR.I**

PFF Mascot score: **[94]** Sequence coverage %: **[12]**

Matched peptides No.: **[2]**

Calculated Mr: **34013** Calculated *p*I: **8.07**

Data base searched result:

Ions score is -10*Log(P), where P is the probability that the observed match is a random event.
Individual ions scores > 30 indicate identity or extensive homology (p<0.05).
Protein scores are derived from ions scores as a non-probabilistic basis for ranking protein hits.

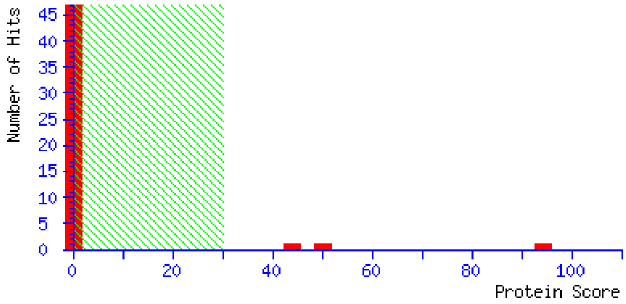

Matched peptide sequences: shown in Bold Red

```
  1 MATRTQAILL LLLAISLIMS SSHVDGGGIA IYWGQNGNEG TLTETCSTGK
 51 YSYVNIAFLN KFGNGQTPQI NLAGHCNPAA GGCTIVSNGI RSCQIQGIKV
101 MLSLGGGIGS YTLASQADAK NVADYLWNNF LGGKSSSRPL GDAVLDGIDF
151 DIEHGSTLYW DDLARYLSAY SKQGKKVYLT AAPQCPFPDR YLGTALNTGL
201 FDYVWVQFYN NPPCQYSSGN INNIINSWNR WTTSINAGKI FLGLPAAPEA
251 AGSGYVPPDV LISRILPEIK KSPKYGGVML WSKFYDDKNG YSSSIRDSVL
301 FLHSEKCMTV L
```

Matched peptide information:

Start - End	Observed	Mr(expt)	Mr(calc)	ppm	Miss	Sequence
177 - 190	1634.7592	1633.7519	1633.7923	-25	0	K.VYLTAAPQCPFPDR.Y (Ions score 40)
240 - 264	2510.2781	2509.2708	2509.3580	-35	0	K.IFLGLPAAPEAAGSGYVPPDVLISR.I (Ions score 81)

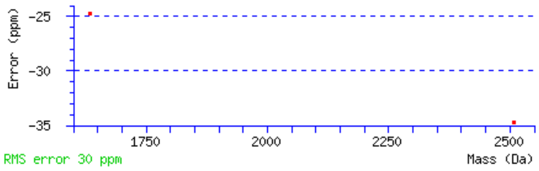

Spot No.:**81**

Accession No.: **scaffold4512_855.mRNA1**

Protein name：**Osmotin-like protein OSM34**

Peptide sequences:

R.NNCPYTVWAAASPGGGR.R;R.LDQGQTWELNVPAGTSMAR.I;K.APGGCNNPCTVFK.T;R.CSDAYSYPQDDPSSTFTCPGGTNYR.V

PFF Mascot score: **[424]**   Sequence coverage %: **[30]**

Matched peptides No.: **[4]**

Calculated Mr: **27682**   Calculated *p*I: **5.23**

Data base searched result:

Ions score is -10*Log(P), where P is the probability that the observed match is a random event.
Individual ions scores > 31 indicate identity or extensive homology (p<0.05).
Protein scores are derived from ions scores as a non-probabilistic basis for ranking protein hits.

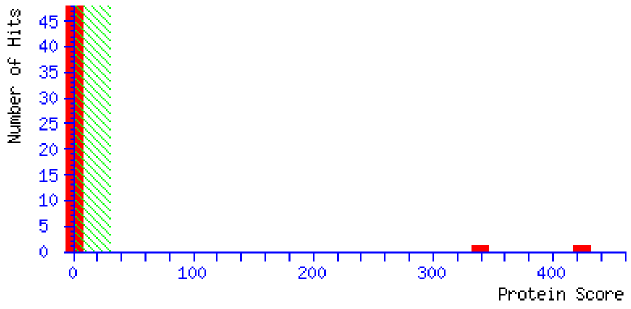

Matched peptide sequences: shown in Bold Red

```
  1 MSNFNIFLIS IFLLSALFFT SSDGATFTIR NNCPYTVWAA ASPGGGRRLD
 51 QGQTWELNVP AGTSMARIWG RTNCNFDGSG KGHCQTGDCG GILACQGWGV
101 PPNTLAEYAL NQFGNLDFYD ISLVDGFNIP IEFSPTSGAK DKCRPLFCTA
151 DINGQCPNQL KAPGGCNNPC TVFKTNEYCC TEGYGTCGPT EFSKFFKSRC
201 SDAYSYPQDD PSSTFTCPGG TNYRVVFCPA RSPHFPLEMV REKDVE
```

Matched peptide information:

Start - End	Observed	Mr(expt)	Mr(calc)	ppm	Miss	Sequence
31 - 47	1777.7799	1776.7726	1776.8002	-16	0	R.NNCPYTVWAAASPGGGR.R (Ions score 135)
49 - 67	2073.9722	2072.9649	2072.9949	-14	0	R.LDQGQTWELNVPAGTSMAR.I (Ions score 118)
162 - 174	1421.6011	1420.5938	1420.6228	-20	0	K.APGGCNNPCTVFK.T (Ions score 99)
200 - 224	2846.0686	2845.0613	2845.1232	-22	0	R.CSDAYSYPQDDPSSTFTCPGGTNYR.V (Ions score 156)

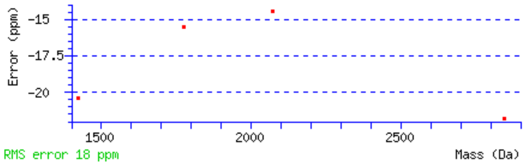

Spot No.:82

Accession No.: scaffold0884_428899.mRNA1

Protein name：Triosephosphateisomerase, cytosolic

Peptide sequences:

K.FFVGGNWK.C;K.DLLRPDFQVAAQNCWVR.K;R.SLLNESNEFVGDK.V;K.VIACIGETLEQR.E;K.VATPAQAQEVHLELR.K;K.VATPAQAQEVHLELRK.W;K.WLHDNVCAEVAASTR.I

PFF Mascot score: [449] Sequence coverage %: [32]

Matched peptides No.: [7]

Calculated Mr: 27571 Calculated *p*I: 5.90

Data base searched result:

Ions score is -10*Log(P), where P is the probability that the observed match is a random event.
Individual ions scores > 30 indicate identity or extensive homology ($p<0.05$).
Protein scores are derived from ions scores as a non-probabilistic basis for ranking protein hits.

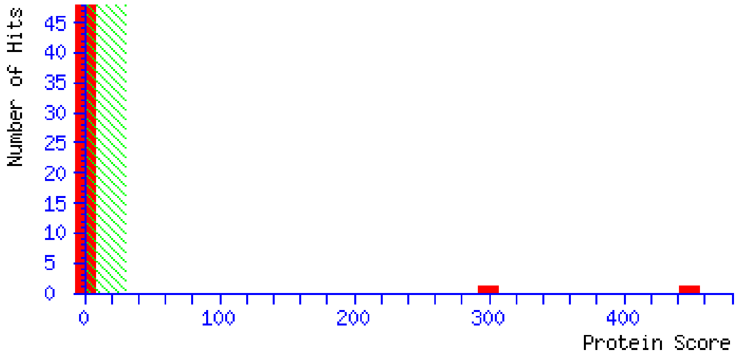

Matched peptide sequences: shown in Bold Red

1 MARK**FFVGGN WK**CNGATEEV KKIVTTLNEA EVPSHDVVEV VVSPPFVFIP
51 PVK**DLLRPDF QVAAQNCWVR** KGGAFTGEVS AEMLVNLSVP WVILGHSERR
101 **SLLNESNEFV GDK**VAYALSQ GLK**VIACIGE TLEQR**ESGST MAVVAAQTKA
151 IAEKVTNWTN VVLAYEPVWA IGTGK**VATPA QAQEVHLELR KWLHDNVCAE**
201 **VAASTR**IIYG GSVNGANCKE LAAKPDVDGF LVGGASLKPE FIDIIKSATV
251 KKE

Matched peptide information:

Start - End	Observed	Mr(expt)	Mr(calc)	ppm	Miss	Sequence
5 - 12	954.4412	953.4339	953.4760	-44	0	K.FFVGGNWK.C (Ions score 63)
54 - 70	2087.9934	2086.9861	2087.0371	-24	1	K.DLLRPDFQVAAQNCWVR.K (Ions score 109)
101 - 113	1451.6553	1450.6480	1450.6940	-32	0	R.SLLNESNEFVGDK.V (Ions score 82)
124 - 135	1388.6877	1387.6805	1387.7129	-23	0	K.VIACIGETLEQR.E (Ions score 96)
176 - 190	1661.8593	1660.8520	1660.8896	-23	0	K.VATPAQAQEVHLELR.K (Ions score 110)
176 - 191	1789.9508	1788.9435	1788.9846	-23	1	K.VATPAQAQEVHLELRK.W (Ions score 33)
192 - 206	1728.7687	1727.7614	1727.8049	-25	0	K.WLHDNVCAEVAASTR.I (Ions score 131)

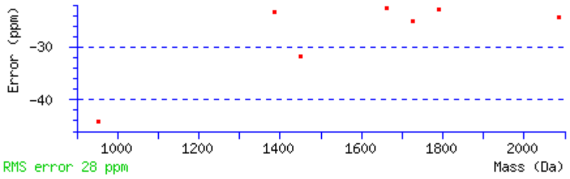

RMS error 28 ppm

Spot No.:83

Accession No.: scaffold0444_830660.mRNA1

Protein name：Alcohol dehydrogenase class-3

Peptide sequences: R.AATGVGIMMNDR.K;K.AFDLMHEGGCLR.C;
K.GWGTSVIVGVAASGQEISTR.P;K.DHDKPIQQVIIDLTDGGVDYSFECIGNVSVMR.S;

PFF Mascot score: [233] Sequence coverage %: [20]

Matched peptides No.: [4]

Calculated Mr: 41710 Calculated pI: 7.49

Data base searched result:

Ions score is -10*Log(P), where P is the probability that the observed match is a random event.
Individual ions scores > 31 indicate identity or extensive homology (p<0.05).
Protein scores are derived from ions scores as a non-probabilistic basis for ranking protein hits.

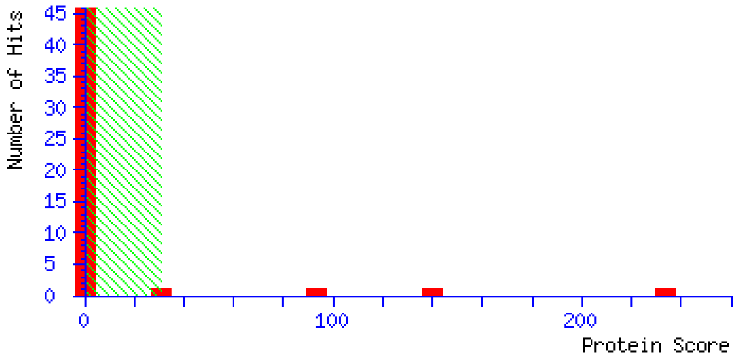

Matched peptide sequences: shown in Bold Red

```
  1 MDEVFQPIGR EGKGKAPIET ECPKKIQRIT NNPVIQKKVV IMPFSDESYS
 51 STNSSTNKDP EGLFPCILGH EAAGIVESVG EGVTEVQPGD HVIPCYQAEC
101 RECKFCRSGK TNLCGKVRAA TGVGIMMNDR KSRFSINGKP IYHFMGTSTF
151 SQYTVVHDVS VAKIDPQAPL EKVCLLGCGV PTGLGAVWNA AKVEAGSIVA
201 IFGLGTVGLA VAEGAKAAGA SRIIGIDIDS KKFDTAKNFG VTEFVNPKDH
251 DKPIQQVIID LTDGGVDYSF ECIGNVSVMR SALECCHKGW GTSVIVGVAA
301 SGQEISTRPF QLVTGRVWKG TAFGGFKSRS QVPWLVDKYM KKEIKVDEYI
351 THNLTLGEIN KAFDLMHEGG CLRCVLSTHV
```

Matched peptide information:

Start - End	Observed	Mr(expt)	Mr(calc)	ppm	Miss	Sequence
119 - 130	1235.5742	1234.5669	1234.5798	-10	0	R.AATGVGIMMNDR.K (Ions score 47)
249 - 280	3620.6455	3619.6382	3619.7287	-25	1	K.DHDKPIQQVIIDLTDGGVDYSFECIGNVSVMR.S (Ions score 101)
289 - 308	1975.0087	1974.0014	1974.0171	-8	0	K.GWGTSVIVGVAASGQEISTR.P (Ions score 97)
362 - 373	1405.6202	1404.6130	1404.6278	-11	0	K.AFDLMHEGGCLR.C (Ions score 74)

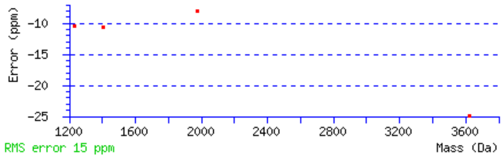

Spot No.:**84**

Accession No.: **scaffold1479_76107.mRNA1**

Protein name： **Acetyl-CoA acetyltransferase, cytosolic 1**

Peptide sequences:

M.APVAAAEIKPR.D;R.DVCIVGVAR.T;R.ANVDPSLVQEVFFGNVLSANLGQAPAR.Q;K.DGLWDVFNDV GMGSCAEICADNHSITR.E;R.EDQDNYAIHSFER.G;R.GIAAQDSGAFAWEIVPVEVSGGR.G;K.ITGYADAA QAPELFTTAPALAIPK.A;K.AVSNAGLDASQVDYYEINEAFAVVALANQK.L;K.VNVHGGAVSLGHPLGCSGA R.I;R.ILVTLLGVLR.Q

PFF Mascot score: **[1070]** Sequence coverage %: **[48]**

Matched peptides No.: **[106]**

Calculated Mr: **41646** Calculated *p*I: **6.01**

Data base searched result:

Ions score is -10*Log(P), where P is the probability that the observed match is a random event
Individual ions scores > 31 indicate identity or extensive homology (p<0.05).
Protein scores are derived from ions scores as a non-probabilistic basis for ranking protein hits.

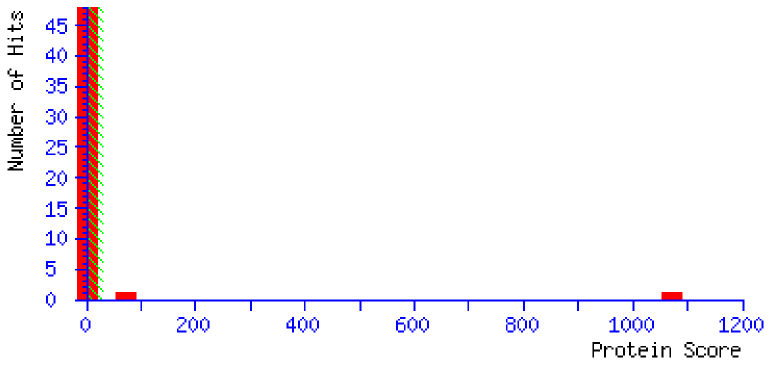

Matched peptide sequences: shown in Bold Red

```
  1 MAPVAAAEIK PRDVCIVGVA RTPMGGFLGL LSTLPATKLG SIAIEAALKR
 51 ANVDPSLVQE VFFGNVLSAN LGQAPARQAA LGAGIPNSVV CTTVNKVCAS
101 GMKATMLAAQ SIQLGINDVV VAGGMESMSN APKYLAEARK GSRLGHDSVV
151 DGMLKDGLWD VFNDVGMGSC AEICADNHSI TREDQDNYAI HSFERGIAAQ
201 DSGAFAWEIV PVEVSGGRGK PSTIVDKDEG LGKFDPVKLR KLRPSFKENG
251 GTVTAGNASS ISDGAAALVL VSGETALKLG LQVIAKITGY ADAAQAPELF
301 TTAPALAIPK AVSNAGLDAS QVDYYEINEA FAVVALANQK LLGLNPEKVN
351 VHGGAVSLGH PLGCSGARIL VTLLGVLRQK NGKYGVGGVC NGGGGASALV
401 VELL
```

Matched peptide information:

Start - End	Observed	Mr(expt)	Mr(calc)	ppm	Miss	Sequence
2 - 12	1122.6429	1121.6357	1121.6557	-18	1	M.APVAAAEIKPR.D (Ions score 68)
13 - 21	988.5054	987.4981	987.5172	-19	0	R.DVCIVGVAR.T (Ions score 51)
51 - 77	2813.4092	2812.4019	2812.4508	-17	0	R.ANVDPSLVQEVFFGNVLSANLGQAPAR.Q (Ions score 244)
156 - 182	3039.2427	3038.2354	3038.2957	-20	0	K.DGLWDVFNDVGMGSCAEICADNHSITR.E (Ions score 173)
183 - 195	1623.6733	1622.6661	1622.6961	-19	0	R.EDQDNYAIHSFER.G (Ions score 131)
196 - 218	2316.1321	2315.1248	2315.1546	-13	0	R.GIAAQDSGAFAWEIVPVEVSGGR.G (Ions score 211)
287 - 310	2430.2380	2429.2308	2429.2842	-22	0	K.ITGYADAAQAPELFTTAPALAIPK.A (Ions score 89)
311 - 340	3170.5054	3169.4981	3169.5567	-19	0	K.AVSNAGLDASQVDYYEINEAFAVVALANQK.L (Ions score 133)
349 - 368	1944.9539	1943.9466	1943.9748	-15	0	K.VNVHGGAVSLGHPLGCSGAR.I (Ions score 172)
369 - 378	1096.7263	1095.7190	1095.7380	-17	0	R.ILVTLLGVLR.Q (Ions score 59)

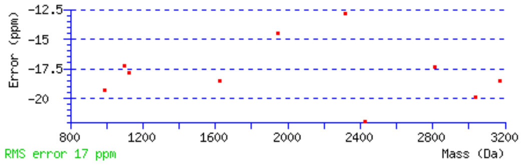

Spot No.:**85**

Accession No.: **scaffold0143_850373.mRNA1**

Protein name：**Hevamine-A**

Peptide sequences:

K.VMLSLGGGIGSYTLASQADAK.N;K.SSSRPLGDAVLDGIDFDIEHGSTLYWDDLAR.Y;R.PLGDAVLDGIDFDIEHGSTLYWDDLAR.Y;K.KVYLTAAPQCPFPDR.Y;K.VYLTAAPQCPFPDR.Y;K.IFLGLPAAPEAAGSGYVPPDVLISR.I;K.YGGVMLWSK.F

PFF Mascot score: **[605]**

Sequence coverage %: **[32]**

Matched peptides No.: **[7]**

Calculated Mr: **34013**

Calculated *p*I: **8.07**

Data base searched result:

Ions score is -10*Log(P), where P is the probability that the observed match is a random event.
Individual ions scores > 30 indicate identity or extensive homology (p<0.05).
Protein scores are derived from ions scores as a non-probabilistic basis for ranking protein hits.

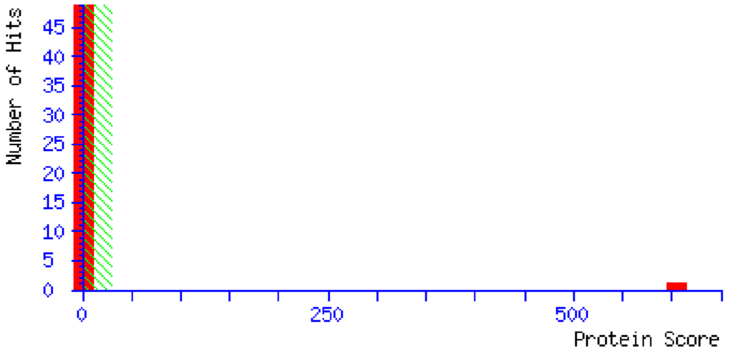

Matched peptide sequences: shown in Bold Red

```
  1 MATRTQAILL LLLAISLIMS SSHVDGGGIA IYWGQNGNEG TLTETCSTGK
 51 YSYVNIAFLN KFGNGQTPQI NLAGHCNPAA GGCTIVSNGI RSCQIQGIKV
101 MLSLGGGIGS YTLASQADAK NVADYLWNNF LGGKSSSRPL GDAVLDGIDF
151 DIEHGSTLYW DDLARYLSAY SKQGKKVYLT AAPQCPFPDR YLGTALNTGL
201 FDYVWVQFYN NPPCQYSSGN INNIINSWNR WTTSINAGKI FLGLPAAPEA
251 AGSGYVPPDV LISRILPEIK KSPKYGGVML WSKFYDDKNG YSSSIRDSVL
301 FLHSEKCMTV L
```

Matched peptide information:

Start - End	Observed	Mr(expt)	Mr(calc)	ppm	Miss	Sequence
100 - 120	2039.0148	2038.0075	2038.0405	-16	0	K.VMLSLGGGIGSYTLASQADAK.N (Ions score 94)
135 - 165	3420.5735	3419.5662	3419.6270	-18	1	K.SSSRPLGDAVLDGIDFDIEHGSTLYWDDLAR.Y (Ions score 80)
139 - 165	3003.3953	3002.3880	3002.4298	-14	0	R.PLGDAVLDGIDFDIEHGSTLYWDDLAR.Y (Ions score 168)
176 - 190	1762.8717	1761.8644	1761.8872	-13	1	K.KVYLTAAPQCPFPDR.Y (Ions score 88)
177 - 190	1634.7803	1633.7730	1633.7923	-12	0	K.VYLTAAPQCPFPDR.Y (Ions score 63)
240 - 264	2510.3376	2509.3304	2509.3580	-11	0	K.IFLGLPAAPEAAGSGYVPPDVLISR.I (Ions score 220)
275 - 283	1040.4962	1039.4889	1039.5161	-26	0	K.YGGVMLWSK.F (Ions score 57)

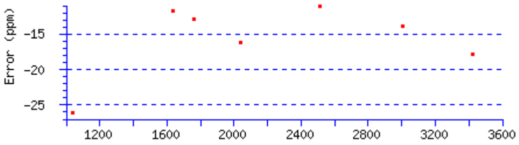

RMS error 16 ppm

Spot No.:**86**

Accession No.: **scaffold0947_141222.mRNA1**

Protein name：**Serpin-ZX**

Peptide sequences:

K.SNDHLSSFFSELVSVVFVDGSASGGPR.L;R.LSFANGVWVDR.S;K.EVLPFGSVDNTTR.L

PFF Mascot score: **[262]** Sequence coverage %: **[13]**

Matched peptides No.: **[3]**

Calculated Mr: **42822** Calculated *p*I: **5.94**

Data base searched result:

Ions score is -10*Log(P), where P is the probability that the observed match is a random event.
Individual ions scores > 31 indicate identity or extensive homology (p<0.05).
Protein scores are derived from ions scores as a non-probabilistic basis for ranking protein hits.

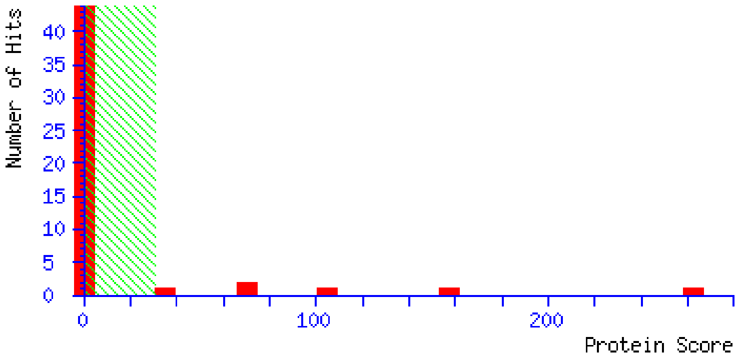

Matched peptide sequences: shown in Bold Red

```
  1 MDLRESIVNQ NAVTLGLSKH VLLTEAKTSN SVLSPLSIQV VLGLIAAGSK
 51 GPTLHQLLSF LNSKSNDHLS SFFSELVSVV FVDGSASGGP RLSFANGVWV
101 DRSLLLKPSF KQVVENVYKA ASNQVDFQNK AVEVTNEVNA WAEKETSGLI
151 KEVLPFGSVD NTTRLIFANA LYFKGAWNEK FNASTTKDYD FYLLNGSSVH
201 VSFMTSKKKQ FICAFDGFKV LGLPYKQGEA KRQFSMYFFL PDAKNGLPAL
251 VEKVGSDSGF LDHHLPQQKV EVGDFRIPRF KVSFGFEASK TLKGLGLVLP
301 FSDKGDLTEM VDSSVSQNLY VSSIFHKSFI EVNEEGTEAA AASAGVVNLE
351 SLTLADKLDF VADHPFLFLI REDISGMVLF IGQVLDPSQA K
```

Matched peptide information:

Start - End	Observed	Mr(expt)	Mr(calc)	ppm	Miss	Sequence
65 - 91	2797.3181	2796.3108	2796.3355	-9	0	K.SNDHLSSFFSELVSVVFVDGSASGGPR.L (Ions score 134)
92 - 102	1263.6425	1262.6352	1262.6408	-4	0	R.LSFANGVWVDR.S (Ions score 85)
152 - 164	1434.7141	1433.7068	1433.7151	-6	0	K.EVLPFGSVDNTTR.L (Ions score 106)

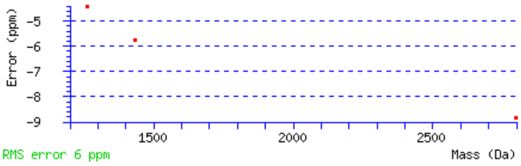

Spot No.:**87**

Accession No.: **scaffold1465_23608.mRNA1**

Protein name：

5-methyltetrahydropteroyltriglutamate--homocysteinemethyltransferase

Peptide sequences:

K.YIPSNTFSYYDQVLDTTAMLGAVPPR.Y;R.YGWSGGEIGFDVYFSMAR.G;K.WFDTNYHFIVPELGPDVQFSYASHK.A;K.KLDLPILPTTTIGSFPQTIELR.R;K.LDLPILPTTTIGSFPQTIELR.R;K.LQEELDIDVLVHGEPER.N;K.AGINVIQIDEAALR.E;K.SEQAFYLDWAVHSFR.I;K.YGAGIGPGVYDIHSPR.I

PFF Mascot score: **[510]** Sequence coverage %: **[20]**

Matched peptides No.: **[9]**

Calculated Mr: **85146** Calculated *p*I: **6.30**

Data base searched result:

Ions score is -10*Log(P), where P is the probability that the observed match is a random event.
Individual ions scores > 30 indicate identity or extensive homology (p<0.05).
Protein scores are derived from ions scores as a non-probabilistic basis for ranking protein hits.

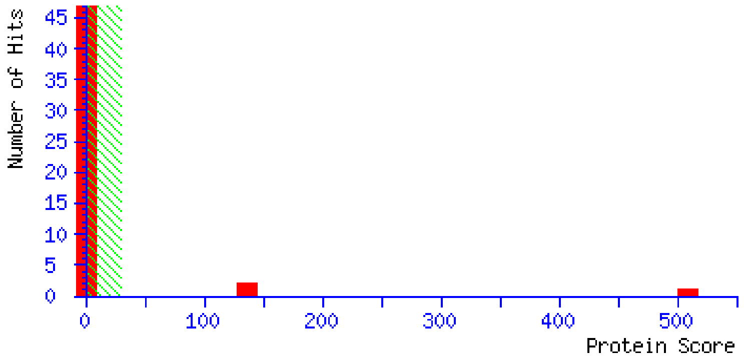

Matched peptide sequences: shown in Bold Red

```
  1 MASHIVGYPR MGPKRELKFA LESFWDKKSS AEDLEKVAAD LRTSIWKQMA
 51 GAGIKYIPSN TFSYYDQVLD TTAMLGAVPP RYGWSGGEIG FDVYFSMARG
101 NASVPAMEMT KWFDTNYHFI VPELGPDVQF SYASHKAVTE YKEAKALGVD
151 SVPVLIGPVS YLLLSKPAKG VEKTFSLISL LGKILPVYKE VISELKAAGA
201 SWIQFDEPTL VMDLDSHKLQ AFTDAYSELE STLSGLNVLI ETYFADIPAK
251 AFKTLTSLKG VSAYGFDLVR GTKTLDLIKS EFPKGKYLFA GVVDGRNIWA
301 NDLASSLSTL HELEGIVGKD KLVVSTSCSL LHTAVDLVNE TKLDKEIKSW
351 LAFAAQKVVE VNALAKALAG EKDEAFFSAN AAAQASRKSS PRVTNEAVQK
401 AAAALRGSDH RRATNVTARL DAQQKKLDLP ILPTTTIGSF PQTIELRRVR
451 REYKANKISE DDYIKAIKEE IQKVVKLQEE LDIDVLVHGE PERNDMVEYF
501 GEQLSGFAFT VNGWVQSYGS RCVKPPIIYG DVSRPKPMTV FWSSTAQSMT
551 ARPMKGMLTG PVTILNWSFV RNDQPRFETC YQIALAIKDE VEDLEKAGIN
601 VIQIDEAALR EGLPLRKSEQ AFYLDWAVHS FRITNCGVQD TTQIHTHMCY
651 SNFNDIIHSI IDMDADVITI ENSRSDEKLL SVFREGVKYG AGIGPGVYDI
701 HSPRIPSTEE IADRINKMLA VLEKNILWVN PDCGLKTRKY SEVKPALNNM
751 VAAAKLLRTQ LASAK
```

Matched peptide information:

Start - End	Observed	Mr(expt)	Mr(calc)	ppm	Miss	Sequence
56 - 81	2919.4341	2918.4268	2918.4160	4	0	K.YIPSNTFSYYDQVLDTTAMLGAVPPR.Y (Ions score 96)
82 - 99	2041.9300	2040.9228	2040.9040	9	0	R.YGWSGGEIGFDVYFSMAR.G (Ions score 75)
112 - 136	2997.4211	2996.4139	2996.4134	0	0	K.WFDTNYHFIVPELGPDVQFSYASHK.A (Ions score 36)
426 - 447	2453.4182	2452.4109	2452.3941	7	1	K.KLDLPILPTTTIGSFPQTIELR.R (Ions score 129)
427 - 447	2325.3250	2324.3177	2324.2991	8	0	K.LDLPILPTTTIGSFPQTIELR.R (Ions score 54)
477 - 493	1991.0353	1990.0280	1990.0007	14	0	K.LQEELDIDVLVHGEPER.N (Ions score 102)
597 - 610	1482.8455	1481.8382	1481.8202	12	0	K.AGINVIQIDEAALR.E (Ions score 76)
618 - 632	1855.8882	1854.8809	1854.8689	6	0	K.SEQAFYLDWAVHSFR.I (Ions score 94)
689 - 704	1658.8379	1657.8306	1657.8213	6	0	K.YGAGIGPGVYDIHSPR.I (Ions score 72)

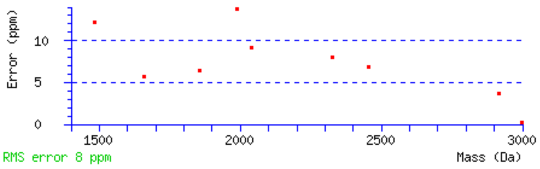

Spot No.: **88**

Accession No.: **scaffold1222_136753.mRNA1**

Protein name： **Rubber elongation factor protein**

Peptide sequences: **K.NVAVPLYNR.F;K.FVDSTVVASVTIIDR.S**

PFF Mascot score: **[76]** Sequence coverage %: **[17]**

Matched peptides No.: **[2]**

Calculated Mr: **14713** Calculated *p*I: **5.04**

Data base searched result:

Ions score is -10*Log(P), where P is the probability that the observed match is a random event
Individual ions scores > 31 indicate identity or extensive homology (p<0.05).
Protein scores are derived from ions scores as a non-probabilistic basis for ranking protein hits.

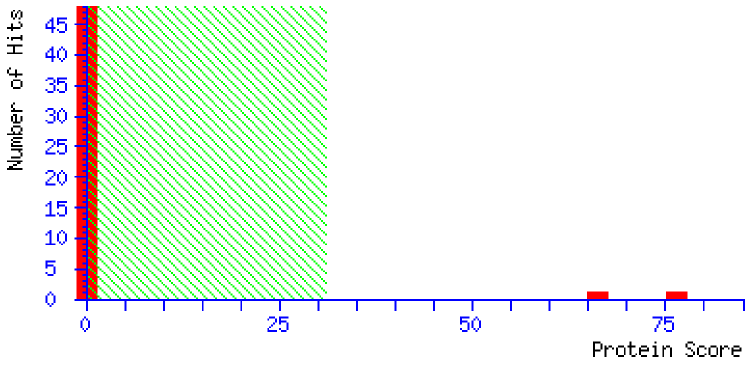

Matched peptide sequences: shown in Bold Red

```
  1 MAEDEDNQQG QGEGLKYLGF VQDAATYAVT TFSNVYLFAK DKSGPLQPGV
 51 DIIEGPVKNV AVPLYNRFSY IPNGALKFVD STVVASVTII DRSLPPIVKD
101 ASIQVVSAIR AAPEAARSLA SSLPGQTKIL AKVFYGEN
```

Matched peptide information:

Start - End	Observed	Mr(expt)	Mr(calc)	ppm	Miss	Sequence
59 - 67	1045.5696	1044.5623	1044.5716	-9	0	K.NVAVPLYNR.F (Ions score 37)
78 - 92	1621.8684	1620.8611	1620.8723	-7	0	K.FVDSTVVASVTIIDR.S (Ions score 68)

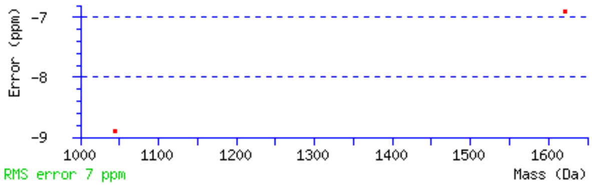

Spot No.:**89**

Accession No.: **scaffold0753_102318.mRNA1**

Protein name：**Hsp70-Hsp90 organizing protein 3**

Peptide sequences:

R.HFTDAISLAPSNHVLYSNR.S;R.LGAAHLGLNQIQDAIAAYK.K;R.MYLQQPDFVK.M;K.FRAPNAGEDMEVPEAEAPPPQPER.K;R.APNAGEDMEVPEAEAPPPQPER.K;K.AMELDDEDISYLTNR.A;K.HDPQNQELLDGVR.R;R.QVLVDFQENPK.A;K.LVNAGIVQIR.-

PFF Mascot score: **[394]** Sequence coverage %: **[20]**

Matched peptides No.: **[9]**

Calculated Mr: **65314** Calculated *p*I: **5.94**

Data base searched result:

Ions score is -10*Log(P), where P is the probability that the observed match is a random event.
Individual ions scores > 31 indicate identity or extensive homology (p<0.05).
Protein scores are derived from ions scores as a non-probabilistic basis for ranking protein hits.

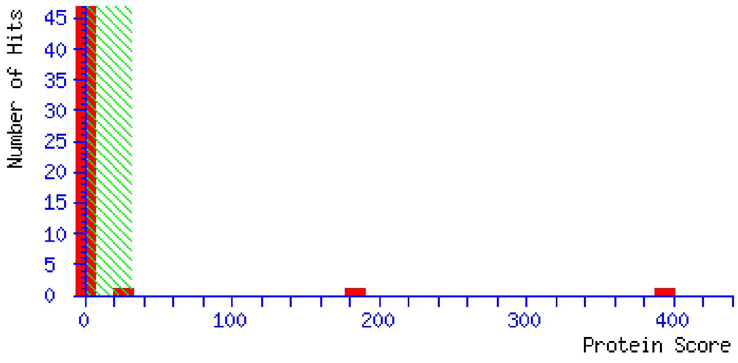

Matched peptide sequences: shown in Bold Red

```
  1 MAEEAKAKGN AAFSAGDYAG AVRHFTDAIS LAPSNHVLYS NRSAALASLH
 51 NYADALADAK KTVELKPDWS KGYSRLGAAH LGLNQIQDAI AAYKKGLEID
101 PNNEALKSGL ADAQAAASRS RSPPPPNPFG DAFSGPEMWA KLTADPTTRM
151 YLQQPDFVKM MQEIQKNPNN LNIYLKDQRV MQALGVLLNV KFRAPNAGED
201 MEVPEAEAPP PQPERKPEPT KEEKVSEPEP EPMEVSEGEK EAKERKAQAV
251 KEKELGNAAY KKKDFETAIA HYTKAMELDD EDISYLTNRA AVYLEMGKYE
301 ECIEDCDKAV ERGRELRSDF KMISRALTRK GTTLVKIAKC SKDYDLAIET
351 FQKALTEHRN PDTLKKLNDA ERAKKELEQQ EYFDSKLADE EREKGNEYFK
401 QQKYPEAVKH YTESLRRNPK DPKAYSNRAA CYTKLGALPE GLKDAEKCIE
451 LDPTFSKGYT RKGAIQFFMK EYDKALETYQ EGLKHDPQNQ ELLDGVRRCV
501 EQLNKASRGD ISPEELKERQ AKAMQDPEIQ NILSDPVMRQ VLVDFQENPK
551 AAQEHTKNPM VMNKIQKLVN AGIVQIR
```

Matched peptide information:

Start - End	Observed	Mr(expt)	Mr(calc)	ppm	Miss	Sequence
24 - 42	2142.0923	2141.0850	2141.0654	9	0	R.HFTDAISLAPSNHVLYSNR.S (Ions score 66)
76 - 94	1967.0790	1966.0717	1966.0636	4	0	R.LGAAHLGLNQIQDAIAAYK.K (Ions score 52)
150 - 159	1268.6342	1267.6269	1267.6271	-0	0	R.MYLQQPDFVK.M (Ions score 19)
192 - 215	2634.2561	2633.2488	2633.2180	12	1	K.FRAPNAGEDMEVPEAEAPPPQPER.K (Ions score 68)
194 - 215	2331.0918	2330.0845	2330.0484	15	0	R.APNAGEDMEVPEAEAPPPQPER.K (Ions score 81)
275 - 289	1784.8239	1783.8166	1783.7934	13	0	K.AMELDDEDISYLTNR.A (Ions score 90)
485 - 497	1520.7566	1519.7493	1519.7379	8	0	K.HDPQNQELLDGVR.R (Ions score 80)
540 - 550	1316.6754	1315.6681	1315.6772	-7	0	R.QVLVDFQENPK.A (Ions score 72)
568 - 577	1082.6672	1081.6600	1081.6608	-1	0	K.LVNAGIVQIR.- (Ions score 61)

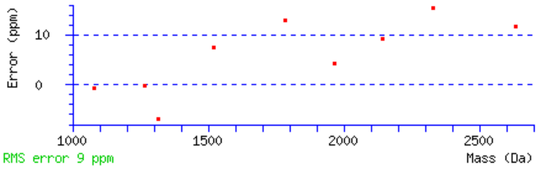

Spot No.:**90**

Accession No.: **scaffold0768_534911.mRNA1**

Protein name：**Desiccation protectant protein Lea14 homolog**

Peptide sequences:

K.IEGAIGFGKPTADVTGIHIPSINLEK.A;K.NPNPIPIPLIDINYLIESDGR.K;K.NPNPIPIPLIDINYLIESDGRK.L;K.LVSGLIPDAGTIHAHGEETVK.I;R.STYNDIKPGSIIPYR.I;R.IKVDLIVDVPVFGR.L;K.DFGSALWDMIR.G

PFF Mascot score: **[450]** Sequence coverage %: **[34]**

Matched peptides No.: **[7]**

Calculated Mr: **35055** Calculated *p*I: **4.71**

Data base searched result:

Ions score is -10*Log(P), where P is the probability that the observed match is a random event.
Individual ions scores > 30 indicate identity or extensive homology (p<0.05).
Protein scores are derived from ions scores as a non-probabilistic basis for ranking protein hits.

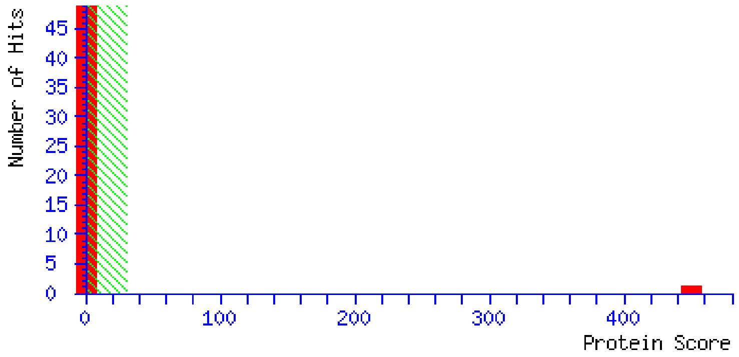

Matched peptide sequences: shown in Bold Red

```
  1 MASSDKPEVI DRDVKEKDHN EDEDKGGFID KVKDFIHDIG EKIEGAIGFG
 51 KPTADVTGIH IPSINLEKAD LVVDVLVKNP NPIPIPLIDI NYLIESDGRK
101 LVSGLIPDAG TIHAHGEETV KIPVTLIYDD IRSTYNDIKP GSIIPYRIKV
151 DLIVDVPVFG RLTLPLEKTG EIPIPYKPDI DLEKIHFERF SFEETVAMLH
201 LKLENKNDFD LGLNGLDYEV WLSDVSIGGA ELAKSTKIDK NGISYIDIPI
251 TFRPKDFGSA LWDMIRGKGT GYTMKGHINV DTPFGAMKLP ISKEGGTTRL
301 KKNKEDGDDD DDDDED
```

Matched peptide information:

Start - End	Observed	Mr(expt)	Mr(calc)	ppm	Miss	Sequence
43 - 68	2677.4763	2676.4690	2676.4487	8	1	K.IEGAIGFGKPTADVTGIHIPSINLEK.A (Ions score 58)
79 - 99	2363.2805	2362.2732	2362.2532	8	0	K.NPNPIPIPLIDINYLIESDGR.K (Ions score 145)
79 - 100	2491.3731	2490.3658	2490.3482	7	1	K.NPNPIPIPLIDINYLIESDGRK.L (Ions score 46)
101 - 121	2144.1521	2143.1448	2143.1273	8	0	K.LVSGLIPDAGTIHAHGEETVK.I (Ions score 81)
133 - 147	1723.9111	1722.9039	1722.8941	6	1	R.STYNDIKPGSIIPYR.I (Ions score 93)
148 - 161	1569.9429	1568.9356	1568.9290	4	1	R.IKVDLIVDVPVFGR.L (Ions score 91)
256 - 266	1310.6240	1309.6167	1309.6125	3	0	K.DFGSALWDMIR.G (Ions score 103)

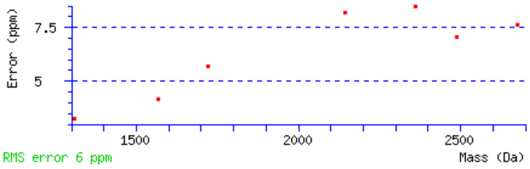

Spot No.:**91**

Accession No.: **scaffold0912_144680.mRNA1**

Protein name： **Ankyrin repeat domain-containing protein 2**

Peptide sequences:

K.SEMSSGESQSGQGR.S;R.YWNDKEVLQK.L;K.NALASGANKDEEDSEGR.T

PFF Mascot score: **[168]**

Sequence coverage %: **[11]**

Matched peptides No.: **[3]**

Calculated Mr: **37639**

Calculated *p*I: **4.50**

Data base searched result:

Ions score is -10*Log(P), where P is the probability that the observed match is a random event.
Individual ions scores > 30 indicate identity or extensive homology ($p<0.05$).
Protein scores are derived from ions scores as a non-probabilistic basis for ranking protein hits.

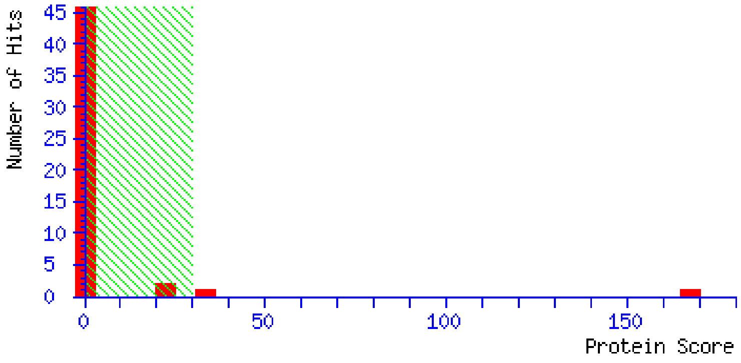

Matched peptide sequences: shown in Bold Red

1 MASNSNKDVP SDEKTGSSEG KIPK**SEMSSG ESQSGQGR**SS PSPGPGLASN
51 VFDFSAMSGL LNDPSIKELA EQISKDPAFN QMAEQLHKTL QGAGAEEGIP
101 QFDSQQYYST MQQVMQNPQF MTMAERLGNA LMQDPSMSQM LESLANPSQK
151 DQIEERMARI KEDPSLKPIL EEIESGGPAT MMR**YWNDKEV LQK**LGEAMGL
201 SVSGEAATSV ENSGQDEAED GGNEDESIVH NCASVGDFEG LK**NALASGAN**
251 **KDEEDSEGR**T ALHFACGYGE VKCAQILLEA GATVDALDKN KNTALHYAAG
301 YGRKECVALL LENGAAVTLQ NMDGKTPIDV AKLNNQHEVL KLLEKDAFL

Matched peptide information:

Start - End	Observed	Mr(expt)	Mr(calc)	ppm	Miss	Sequence
25 - 38	1426.6001	1425.5928	1425.5790	10	0	K.SEMSSGESQSGQGR.S (Ions score 81)
184 - 193	1322.6709	1321.6636	1321.6666	-2	1	R.YWNDKEVLQK.L (Ions score 71)
243 - 259	1762.8052	1761.7979	1761.7765	12	1	K.NALASGANKDEEDSEGR.T (Ions score 75)

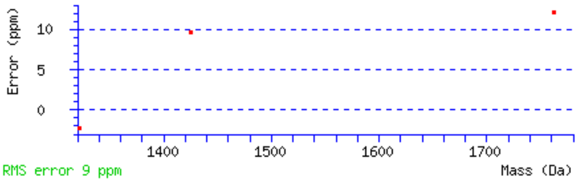

Spot No.:**92**

Accession No.: **scaffold0280_1322570.mRNA1**

Protein name：**Dynamin-related protein 4C**

Peptide sequences:

K.SSVLESLAGINLPR.G;K.TVYTDEAR.A;K.GIPDLTMIDLPGITR.V;R.GEFDEYLNDHQMHCTAR.L;R.LVEMLNLYSNELHK.C;R.NFLVEEIR.L;K.LMNQQDTFR.G;K.AHQDVLHQAFDLK.M;K.EIISELMSNQGGVIER.M

PFF Mascot score: **[505]**   Sequence coverage %: **[17]**

Matched peptides No.: **[9]**

Calculated Mr: **74315**   Calculated *p*I: **5.91**

Data base searched result:

Ions score is -10*Log(P), where P is the probability that the observed match is a random event.
Individual ions scores > 31 indicate identity or extensive homology (p<0.05).
Protein scores are derived from ions scores as a non-probabilistic basis for ranking protein hits.

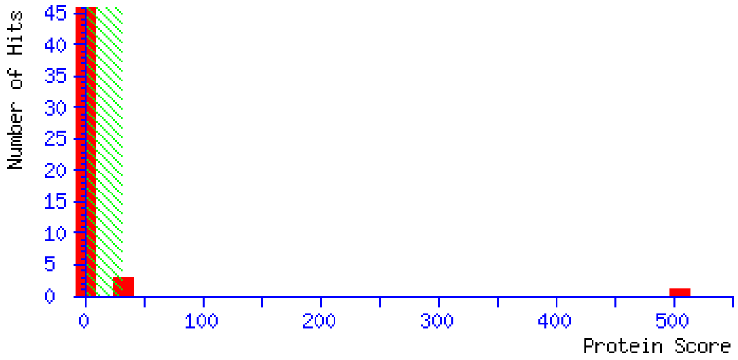

Matched peptide sequences: shown in Bold Red

```
  1 MAPISNGPDL VEYEDANMEN QVPLVSSYND HIRPLLDAVD KLRHLKVMNE
 51 GIQLPTIVVV GDQSSGKSSV LESLAGINLP RGQGICTRVP LVMRLQHQPT
101 PTPEVFLEFN GKTVYTDEAR AADAISLATD EIAGDGKGIS NTPLTLVVKK
151 KGIPDLTMID LPGITRVPVH GQPEDIYEQI TGIITEYIRP EESIILNVLS
201 ATVDFPTCES IRMSRQVDKT GERTLAVVTK SDKAPEGLLE KVAADDVNIG
251 LGYVCVKNRI GDESFEDARK EEAQLFKKHP LLSRIDKSMV GIPFLAQKLT
301 QIQATIIAKC LPDIARNINE KLNASISELN KMPRTPSSPA EAMTAFMGIV
351 GSAKESLRKI LIRGEFDEYL NDHQMHCTAR LVEMLNLYSN ELHKCSESDP
401 TRNFLVEEIR LLEESKGIKL PNFLPHSALL SILQKKVDGI SRMHIDFVEK
451 IWDYIESVVL SVLMHHSENY HQLQSSTRRA GQNLISKMKE QSIGWVTEII
501 QMEKMTDYTC NPNYMSEWSK LMNQQDTFRG KILIQGHSKA KIDGIGEVEA
551 GHIKAHQDVL HQAFDLKMRM TAYWKIVLSR LVDSMALHLQ FCVQNLVNKE
601 MEKEIISELM SNQGGVIERM MEESPSIAAK REKLNKSIKL LGESKKVLGN
651 IMDKIATYSD
```

Matched peptide information:

Start - End	Observed	Mr(expt)	Mr(calc)	ppm	Miss	Sequence
68 - 81	1455.8569	1454.8497	1454.8093	28	0	K.SSVLESLAGINLPR.G (Ions score 109)
113 - 120	954.4701	953.4628	953.4454	18	0	K.TVYTDEAR.A (Ions score 15)
152 - 166	1611.9259	1610.9186	1610.8702	30	0	K.GIPDLTMIDLPGITR.V (Ions score 112)
364 - 380	2122.9443	2121.9371	2121.8633	35	0	R.GEFDEYLNDHQMHCTAR.L (Ions score 112)
381 - 394	1702.9420	1701.9347	1701.8759	35	0	R.LVEMLNLYSNELHK.C (Ions score 50)
403 - 410	1019.5642	1018.5569	1018.5447	12	0	R.NFLVEEIR.L (Ions score 64)
521 - 529	1152.5763	1151.5690	1151.5393	26	0	K.LMNQQDTFR.G (Ions score 42)
555 - 567	1521.8292	1520.8219	1520.7736	32	0	K.AHQDVLHQAFDLK.M (Ions score 101)
604 - 619	1774.9562	1773.9489	1773.8931	31	0	K.EIISELMSNQGGVIER.M (Ions score 129)

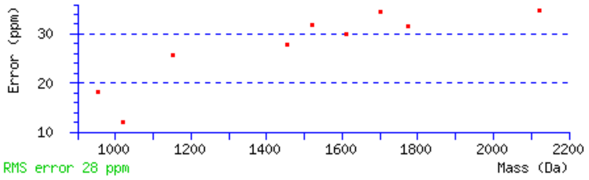

Spot No.:**93**

Accession No.: **scaffold0195_965653.mRNA1**

Protein name：**UDP-arabinopyranosemutase 3**

Peptide sequences:

K.VPEGFDYELHNR.N;K.YVYTIDDDCFVAK.D;K.DINALEQHIK.N;K.VICDHLGFGVK.T

PFF Mascot score: **[122]** Sequence coverage %: **[12]**

Matched peptides No.: **[4]**

Calculated Mr: **41793** Calculated *p*I: **5.76**

Data base searched result:

Ions score is -10*Log(P), where P is the probability that the observed match is a random event.
Individual ions scores > 32 indicate identity or extensive homology (p<0.05).
Protein scores are derived from ions scores as a non-probabilistic basis for ranking protein hits.

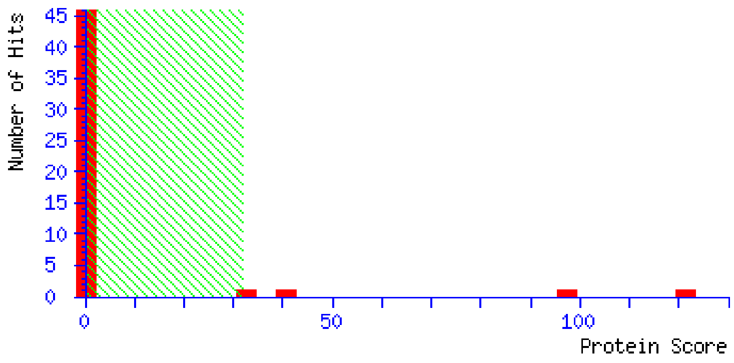

Matched peptide sequences: shown in Bold Red

```
  1 MAETAVSLTP TPLLKDELDI VIPTIRNLDF LEMWRPFFQP YHLIIVQDGD
 51 PSKTIKVPEG FDYELHNRND INRILGPKAS CISFKDSACR CFGYLVSKKK
101 YVYTIDDDCF VAKDPSGKDI NALEQHIKNL LSPSTPFFFN TLYDPYRNGA
151 DFVRGYPFSL REGVPTAVSH GLWLNIPDYD APTQLVKPLE RNTRYVDAVM
201 TIPKGTLFPM CGMNLAFNRE LIGPAMYFGL MGDGQPIGRY DDMWAGWCTK
251 VICDHLGFGV KTGLPYIYHS KASNPFVNLK KEYKGIYWQE ELIPFFQSAV
301 LPKDCTTVQK CYIELSKQVR AKLGKVDEYF IKLADAMVTW IEAWDELNSS
351 GNSGELADGT GKSNS
```

Matched peptide information:

Start	-	End	Observed	Mr(expt)	Mr(calc)	ppm	Miss	Sequence	
57	-	68	1475.7444	1474.7371	1474.6841	36	0	K.VPEGFDYELHNR.N	(Ions score 96)
101	-	113	1608.7779	1607.7707	1607.7178	33	0	K.YVYTIDDDCFVAK.D	(Ions score 46)
119	-	128	1180.6686	1179.6613	1179.6248	31	0	K.DINALEQHIK.N	(Ions score 19)
251	-	261	1244.6919	1243.6846	1243.6384	37	0	K.VICDHLGFGVK.T	(Ions score 35)

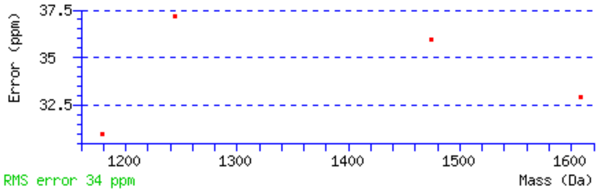

Spot No.:**94**

Accession No.: **scaffold0014_23288.mRNA1**

Protein name：**Triosephosphateisomerase, cytosolic**

Peptide sequences:

K.FFVGGNWK.C;K.GLLRPDFQVAAQNCWVR.K;K.GGAFTGEISAEMIVNLSVPWVILGHSER.R;R.RSLLNESNEFVGDK.V;R.SLLNESNEFVGDK.V;K.VIACIGETLEQR.E;K.VTNWANVVLAYEPVWAIGTGK.V;K.VATPAQAQEVHLELR.K;K.VATPAQAQEVHLELRK.W

PFF Mascot score: **[422]** Sequence coverage %: **[45]**

Matched peptides No.: **[9]**

Calculated Mr: **27533** Calculated *p*I: **5.76**

Data base searched result:

Ions score is -10*Log(P), where P is the probability that the observed match is a random event.
Individual ions scores > 30 indicate identity or extensive homology ($p<0.05$).
Protein scores are derived from ions scores as a non-probabilistic basis for ranking protein hits.

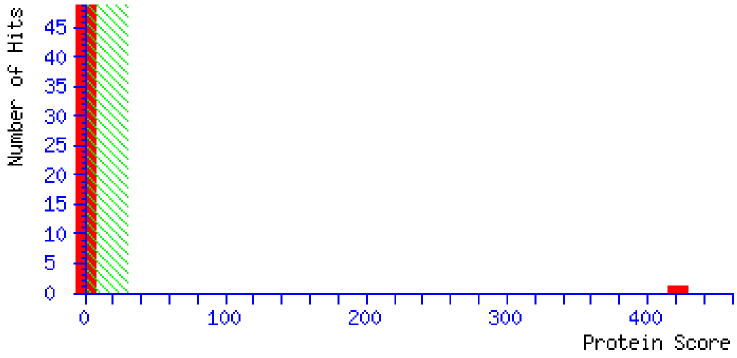

Matched peptide sequences: shown in Bold Red

```
  1 MARKFFVGGN WKCNGTTEEV KKIVTILNEA EVPSEDVVEV VVSPPFVFLS
 51 LVKGLLRPDF QVAAQNCWVR KGGAFTGEIS AEMIVNLSVP WVILGHSERR
101 SLLNESNEFV GDKVAYALSK GLKVIACIGE TLEQRESGST LAVVAAQTKA
151 IADKVTNWAN VVLAYEPVWA IGTGKVATPA QAQEVHLELR KWLHDNVSAE
201 VAASTRIIYG GSVNGANCKE LAAKPDVDGF LVGGASLKPE FIDIIKSATV
251 KKDD
```

Matched peptide information:

Start - End	Observed	Mr(expt)	Mr(calc)	ppm	Miss	Sequence
5 - 12	954.4672	953.4599	953.4760	-17	0	K.FFVGGNWK.C (Ions score 60)
54 - 70	2030.0405	2029.0333	2029.0316	1	1	K.GLLRPDFQVAAQNCWVR.K (Ions score 109)
72 - 99	2969.4958	2968.4886	2968.5117	-8	0	K.GGAFTGEISAEMIVNLSVPWVILGHSER.R (Ions score 98)
100 - 113	1607.8009	1606.7936	1606.7951	-1	1	R.RSLLNESNEFVGDK.V (Ions score 23)
101 - 113	1451.6917	1450.6844	1450.6940	-7	0	R.SLLNESNEFVGDK.V (Ions score 74)
124 - 135	1388.7200	1387.7127	1387.7129	-0	0	K.VIACIGETLEQR.E (Ions score 93)
155 - 175	2288.2056	2287.1983	2287.2001	-1	0	K.VTNWANVVLAYEPVWAIGTGK.V (Ions score 68)
176 - 190	1661.8965	1660.8892	1660.8896	-0	0	K.VATPAQAQEVHLELR.K (Ions score 89)
176 - 191	1789.9915	1788.9842	1788.9846	-0	1	K.VATPAQAQEVHLELRK.W (Ions score 8)

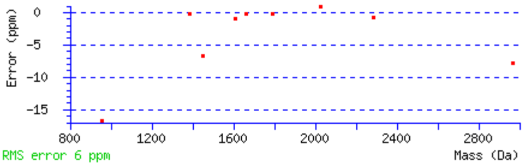

Spot No.:**95**

Accession No.: **scaffold0434_14371.mRNA1**

Protein name： **Glutathione S-transferase F9**

Peptide sequences:

K.VYGTAYASPK.R;K.RVLACLIEK.G;K.LQPFGALPVIQDGDYTLYESR.A;K.VLDVYEER.L;K.YLAGDFFSLADLSHLPFTQYLVGPINK.E;R.KHVSAWWDQISSR.P;K.HVSAWWDQISSR.P

PFF Mascot score: **[465]** Sequence coverage %: **[39]**

Matched peptides No.: **[7]**

Calculated Mr: **25501** Calculated *p*I: **8.44**

Data base searched result:

Ions score is -10*Log(P), where P is the probability that the observed match is a random event.
Individual ions scores > 31 indicate identity or extensive homology (p<0.05).
Protein scores are derived from ions scores as a non-probabilistic basis for ranking protein hits.

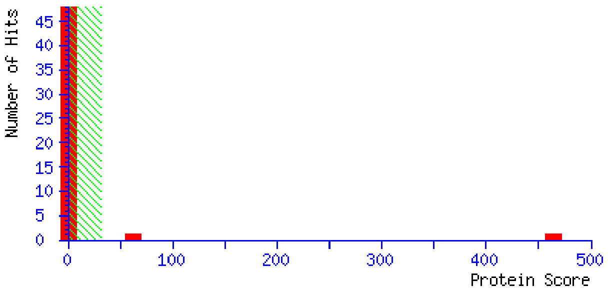

Matched peptide sequences: shown in Bold Red

```
  1 MVVKVYGTAY ASPKRVLACL IEKGIEFEAV PVDLIKGEHR SPEYLKLQPF
 51 GALPVIQDGD YTLYESRAII RYYAEKYKSQ GTDLLGKSIE ERGLVEQWLE
101 VEAQNFHPHI YNLTLHILFA SALGFPPDEK VIKESEEKLG KVLDVYEERL
151 SKSKYLAGDF FSLADLSHLP FTQYLVGPIN KEYMIRSRKH VSAWWDQISS
201 RPSWKKAAGH LGRRSSNFKM MS
```

Matched peptide information:

Start - End	Observed	Mr(expt)	Mr(calc)	ppm	Miss	Sequence
5 - 14	1056.5251	1055.5179	1055.5287	-10	0	K.VYGTAYASPK.R (Ions score 58)
15 - 23	1101.6511	1100.6438	1100.6376	6	1	K.RVLACLIEK.G (Ions score 39)
47 - 67	2382.2022	2381.1949	2381.1903	2	0	K.LQPFGALPVIQDGDYTLYESR.A (Ions score 207)
142 - 149	1022.5108	1021.5035	1021.5080	-4	0	K.VLDVYEER.L (Ions score 48)
155 - 181	3026.5620	3025.5547	3025.5589	-1	0	K.YLAGDFFSLADLSHLPFTQYLVGPINK.E (Ions score 105
189 - 201	1599.8026	1598.7953	1598.7954	-0	1	R.KHVSAWWDQISSR.P (Ions score 89)
190 - 201	1471.7056	1470.6983	1470.7004	-1	0	K.HVSAWWDQISSR.P (Ions score 98)

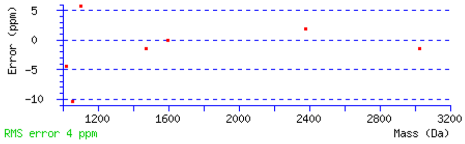

Spot No.:96

Accession No.: scaffold0139_994147.mRNA1

Protein name：Glucose and ribitol dehydrogenase

Peptide sequences:

R.FPPQTQDR.Q;K.EYLMHPLPEFLNPQYKPSNK.L;K.VALVTGGDSGIGR.A;K.AEGAKDPIAIPTDVGFEENCR.S;K.DPIAIPTDVGFEENCR.S;R.TNIFGQFFMTR.Y;K.IAALGSEVPMDR.A

PFF Mascot score: [478] Sequence coverage %: [28]

Matched peptides No.: [7]

Calculated Mr: 32309 Calculated *p*I: 5.29

Data base searched result:

Ions score is -10*Log(P), where P is the probability that the observed match is a random event.
Individual ions scores > 31 indicate identity or extensive homology ($p<0.05$).
Protein scores are derived from ions scores as a non-probabilistic basis for ranking protein hits.

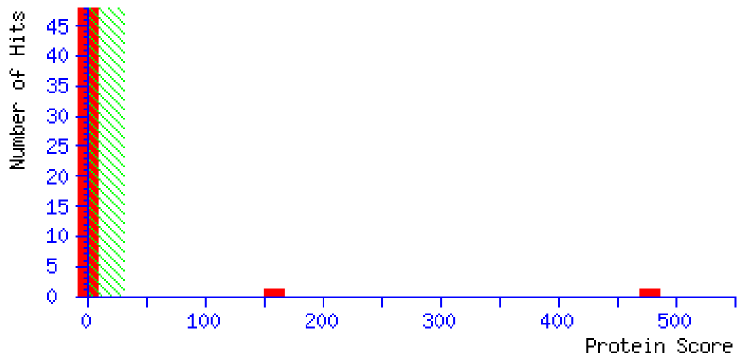

Matched peptide sequences: shown in Bold Red

1 MATQSGFR**FP PQTQDR**QPGK **EYLMHPLPEF LNPQYKPSNK** LLGK**VALVTG**

51 **GDSGIGR**AVS YYFTLEGATV AFTYVKGQED MDTDHTLKIL SESK**AEGAKD**

101 **PIAIPTDVGF EENCR**SVVDQ VMAEYGHIDI LVNNAAEQYY STTIEDITET

151 RLERVFR**TNI FGQFFMTR**YA LQHMKQGSCI INTTSVVAYA GYSGLLDYSS

201 TKGAIVAFTR SLSLQLIDKG IRVNAVAPGP VWTPLQPASL PAEK**IAALGS**

251 **EVPMDR**AAQP YEIAPSYVFL ASNECSSYIT GQVLHPNGGY IVNA

Matched peptide information:

Start	-	End	Observed	Mr(expt)	Mr(calc)	ppm	Miss	Sequence
9	-	16	988.4696	987.4623	987.4774	-15	0	R.FPPQTQDR.Q (Ions score 56)
21	-	40	2445.1939	2444.1866	2444.2198	-14	1	K.EYLMHPLPEFLNPQYKPSNK.L (Ions score 55)
45	-	57	1201.6408	1200.6335	1200.6463	-11	0	K.VALVTGGDSGIGR.A (Ions score 87)
95	-	115	2289.0610	2288.0538	2288.0743	-9	1	K.AEGAKDPIAIPTDVGFEENCR.S (Ions score 143)
100	-	115	1832.8307	1831.8234	1831.8411	-10	0	K.DPIAIPTDVGFEENCR.S (Ions score 161)
158	-	168	1361.6523	1360.6451	1360.6598	-11	0	R.TNIFGQFFMTR.Y (Ions score 90)
245	-	256	1258.6321	1257.6248	1257.6387	-11	0	K.IAALGSEVPMDR.A (Ions score 64)

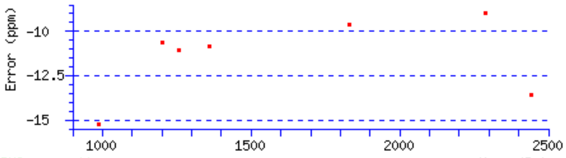

RMS error 11 ppm

Spot No.:97

Accession No.: scaffold0041_2570834.mRNA1

Protein name： Aspartic proteinase A1

Peptide sequences:

K.NYMDAQYFGEIGIGTPPQK.F;K.FTVIFDTGSSNLWVPSSK.C;K.CYFSVACYFHSR.Y;K.SADIHYGTGAISGFFSQDHVK.V;K.FDGILGLGFEEISVGK.A;K.AVPVWYNMVNQGLVK.E;K.EPVFSFWFNR.N;K.AVVAQYGETIIEMLLAK.D

PFF Mascot score: [606] Sequence coverage %: [24]

Matched peptides No.: [8]

Calculated Mr: 56526 Calculated *p*I: 5.73

Data base searched result:

Ions score is -10*Log(P), where P is the probability that the observed match is a random event.
Individual ions scores > 30 indicate identity or extensive homology (p<0.05).
Protein scores are derived from ions scores as a non-probabilistic basis for ranking protein hits.

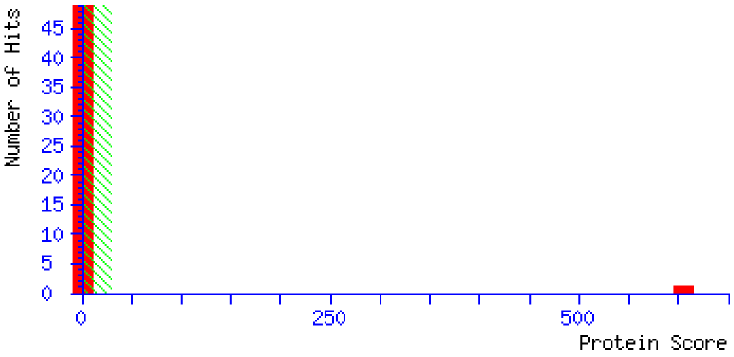

Matched peptide sequences: shown in Bold Red

1 MGTRSKPITT ALFLCFLLLP LVFAAHNDGL VRIGLKKRKF DQNNLVAAQF
51 ESKEGESLRT SFKKYHFHGN LGDAEDIDII SLK**NYMDAQY** **FGEIGIGTPP**
101 **QKFTVIFDTG** **SSNLWVPSSK** **CYFSVACYFH** **SR**YKSGHSST YKKNGK**SADI**
151 **HYGTGAISGF** **FSQDHVK**VGG LVVKDQEFIE ATKEPSITFL VAK**FDGILGL**
201 **GFEEISVGKA** **VPVWYNMVNQ** **GLVKEPVFSF** **WFNR**NADEDE GGEIVFGGMD
251 PKHYKGEHTY VPVTQKGYWQ FNMGDVLIDG KTTGICGSGC AAIADSGTSL
301 LAGPTGIIAE VNHAIGATGI VSQECK**AVVA** **QYGETIIEML** **LAK**DQPQKIC
351 SQIGLCTFDG SRGVSVGIKS VVNENIQGVA SGLRDAMCST CEMAVVWMQN
401 QLKLNNTQER ILNYANELCE RLPSPMGESA VDCGGLSTMP NVSFTIGGKV
451 FDLSPEQYVL KVGEGEAAQC ISGFTALDVP PPRGPLWILG DVFMGRFHTV
501 FDYGNKRVGF AEAA

Matched peptide information:

Start	-	End	Observed	Mr(expt)	Mr(calc)	ppm	Miss	Sequence
84	-	102	2128.9756	2127.9683	2127.9935	-12	0	K.NYMDAQYFGEIGIGTPPQK.F (Ions score 128)
103	-	120	1984.9685	1983.9612	1983.9942	-17	0	K.FTVIFDTGSSNLWVPSSK.C (Ions score 83)
121	-	132	1596.6415	1595.6342	1595.6650	-19	0	K.CYFSVACYFHSR.Y (Ions score 79)
147	-	167	2237.0288	2236.0215	2236.0549	-15	0	K.SADIHYGTGAISGFFSQDHVK.V (Ions score 165)
194	-	209	1680.8535	1679.8462	1679.8770	-18	0	K.FDGILGLGFEEISVGK.A (Ions score 98)
210	-	224	1717.8716	1716.8643	1716.9021	-22	0	K.AVPVWYNMVNQGLVK.E (Ions score 85)
225	-	234	1328.6106	1327.6033	1327.6350	-24	0	K.EPVFSFWFNR.N (Ions score 90)
327	-	343	1848.9785	1847.9712	1848.0066	-19	0	K.AVVAQYGETIIEMLLAK.D (Ions score 79)

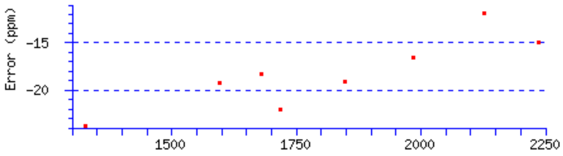

RMS error 18 ppm

Spot No.:**98**

Accession No.: **scaffold0105_276835.mRNA1**

Protein name：**Proteasome subunit beta type-1**

Peptide sequences:

R.MSTGYSILTR.E;R.FFPYYAFNVLGGLDSEGK.G;K.GCVYTYDAVGSYER.V;R.VGYSAQGSGSTLIMPFLDNQLK.S;K.SPSPLLLPAQDAVTPLSELEAVDLVK.T;R.DIYTGDKLEIVILNADGIR.H ;K.LEIVILNADGIR.H;R.HEYMELR.K

PFF Mascot score: **[498]** Sequence coverage %: **[52]**

Matched peptides No.: **[8]**

Calculated Mr: **24879** Calculated *p*I: **6.20**

Data base searched result:

Ions score is -10*Log(P), where P is the probability that the observed match is a random event.
Individual ions scores > 31 indicate identity or extensive homology (p<0.05).
Protein scores are derived from ions scores as a non-probabilistic basis for ranking protein hits.

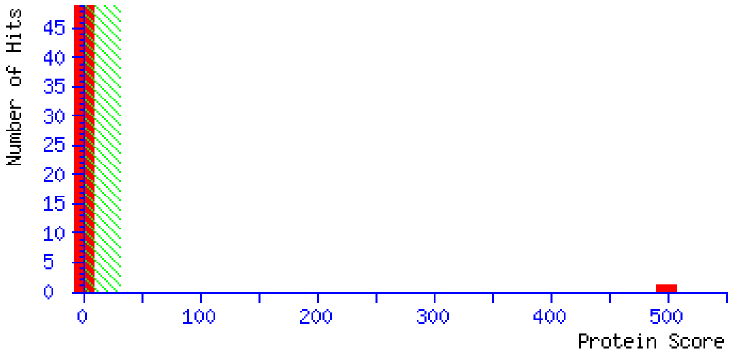

Matched peptide sequences: shown in Bold Red

```
  1 MTKQQANWSP YDNNGGSCVA IAGADYCVIA ADTRMSTGYS ILTREYSKIC
 51 KLADKSVMAS SGFQADVKAL QKHLAARHLI YQHQHNKQMS CPAMAQLLSN
101 TLYYKRFFPY YAFNVLGGLD SEGKGCVYTY DAVGSYERVG YSAQGSGSTL
151 IMPFLDNQLK SPSPLLLPAQ DAVTPLSELE AVDLVKTVFA SATERDIYTG
201 DKLEIVILNA DGIRHEYMEL RKD
```

Matched peptide information:

Start	-	End	Observed	Mr(expt)	Mr(calc)	ppm	Miss	Sequence
35	-	44	1128.5516	1127.5444	1127.5645	-18	0	R.MSTGYSILTR.E (Ions score 31)
107	-	124	2023.9409	2022.9336	2022.9727	-19	0	R.FFPYYAFNVLGGLDSEGK.G (Ions score 72)
125	-	138	1639.6799	1638.6727	1638.6984	-16	0	K.GCVYTYDAVGSYER.V (Ions score 115)
139	-	160	2326.1357	2325.1285	2325.1675	-17	0	R.VGYSAQGSGSTLIMPFLDNQLK.S (Ions score 79)
161	-	186	2702.4365	2701.4292	2701.4789	-18	0	K.SPSPLLLPAQDAVTPLSELEAVDLVK.T (Ions score 124)
196	-	214	2118.1140	2117.1067	2117.1368	-14	1	R.DIYTGDKLEIVILNADGIR.H (Ions score 186)
203	-	214	1325.7551	1324.7479	1324.7714	-18	0	K.LEIVILNADGIR.H (Ions score 42)
215	-	221	977.4186	976.4113	976.4436	-33	0	R.HEYMELR.K (Ions score 36)

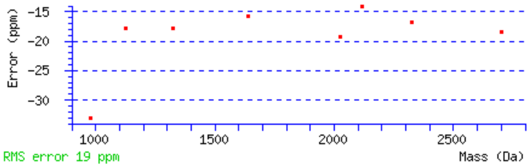

Spot No.:**99**

Accession No.: **scaffold1479_76107.mRNA1**

Protein name： **Acetyl-CoA acetyltransferase, cytosolic 1**

Peptide sequences:

M.APVAAAEIKPR.D;R.DVCIVGVAR.T;K.DGLWDVFNDVGMGSCAEICADNHSITR.E;R.EDQDNYAIHSFER.G;R.GIAAQDSGAFAWEIVPVEVSGGR.G;K.VNVHGGAVSLGHPLGCSGAR.I;R.ILVTLLGVLR.Q

PFF Mascot score: **[639]** Sequence coverage %: **[27]**

Matched peptides No.: **[7]**

Calculated Mr: **41646** Calculated *p*I: **6.01**

Data base searched result:

Ions score is -10*Log(P), where P is the probability that the observed match is a random event.
Individual ions scores > 31 indicate identity or extensive homology (p<0.05).
Protein scores are derived from ions scores as a non-probabilistic basis for ranking protein hits.

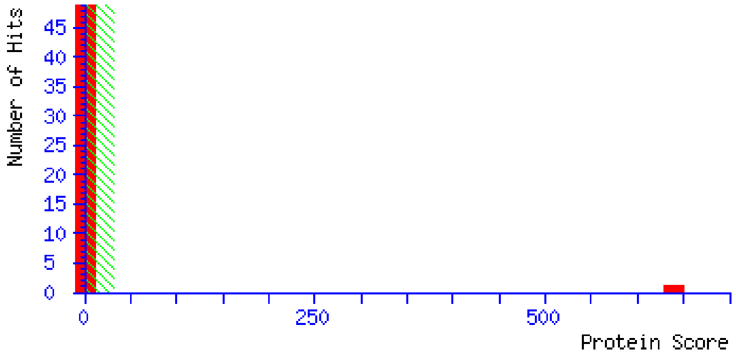

Matched peptide sequences: shown in Bold Red

```
  1 MAPVAAAEIK PRDVCIVGVA RTPMGGFLGL LSTLPATKLG SIAIEAALKR
 51 ANVDPSLVQE VFFGNVLSAN LGQAPARQAA LGAGIPNSVV CTTVNKVCAS
101 GMKATMLAAQ SIQLGINDVV VAGGMESMSN APKYLAEARK GSRLGHDSVV
151 DGMLKDGLWD VFNDVGMGSC AEICADNHSI TREDQDNYAI HSFERGIAAQ
201 DSGAFAWEIV PVEVSGGRGK PSTIVDKDEG LGKFDPVKLR KLRPSFKENG
251 GTVTAGNASS ISDGAAALVL VSGETALKLG LQVIAKITGY ADAAQAPELF
301 TTAPALAIPK AVSNAGLDAS QVDYYEINEA FAVVALANQK LLGLNPEKVN
351 VHGGAVSLGH PLGCSGARIL VTLLGVLRQK NGKYGVGGVC NGGGGASALV
401 VELL
```

Matched peptide information:

Start - End	Observed	Mr(expt)	Mr(calc)	ppm	Miss	Sequence
2 - 12	1122.6710	1121.6637	1121.6557	7	1	M.APVAAAEIKPR.D (Ions score 53)
13 - 21	988.5247	987.5174	987.5172	0	0	R.DVCIVGVAR.T (Ions score 46)
156 - 182	3039.2402	3038.2330	3038.2957	-21	0	K.DGLWDVFNDVGMGSCAEICADNHSITR.E (Ions score 120)
183 - 195	1623.6862	1622.6789	1622.6961	-11	0	R.EDQDNYAIHSFER.G (Ions score 136)
196 - 218	2316.1262	2315.1189	2315.1546	-15	0	R.GIAAQDSGAFAWEIVPVEVSGGR.G (Ions score 228)
349 - 368	1944.9669	1943.9596	1943.9748	-8	0	K.VNVHGGAVSLGHPLGCSGAR.I (Ions score 176)
369 - 378	1096.7346	1095.7273	1095.7380	-10	0	R.ILVTLLGVLR.Q (Ions score 60)

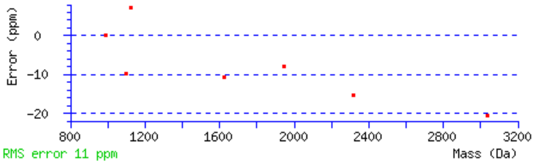

Spot No.:**100**

Accession No.: **scaffold0283_54035.mRNA1**

Protein name: **GEM-like protein 5**

Peptide sequences:

K.PSNNPFEPVIQAFNTWSR.K;K.AITEGGFESLFK.Q;K.TFACYLSTSTGPVAGTLYLSTAR.V;R.VAFCSDRPLSFMAPSGQETWCYYK.V;K.YIQMVTIDGHDFWFMGFVNFEK.A;K.ASHHLLDAVSNFR.A

PFF Mascot score: **[369]** Sequence coverage %: **[42]**

Matched peptides No.: **[7]**

Calculated Mr: **29282** Calculated *p*I: **6.55**

Data base searched result:

Ions score is -10*Log(P), where P is the probability that the observed match is a random event.
Individual ions scores > 30 indicate identity or extensive homology (p<0.05).
Protein scores are derived from ions scores as a non-probabilistic basis for ranking protein hits.

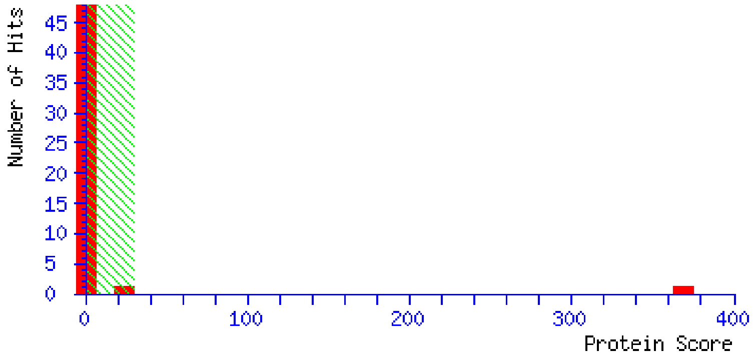

Matched peptide sequences: shown in Bold Red

```
  1 MATTSQETQI AESHEPSQPH QPPSKQEAEK WGTHVMGSPA APTLHPDNQK
 51 AASWNAADHQ QIYQQPYLVY SPVEKPSNNP FEPVIQAFNT WSRKAENIAR
101 NIWHNLTTGP SVSETAWGKV NLTAKAITEG GFESLFKQVF ETHLNEKLKK
151 TFACYLSTST GPVAGTLYLS TARVAFCSDR PLSFMAPSGQ ETWCYYKVMI
201 PLNKIGQVNP VVVKENPPEK YIQMVTIDGH DFWFMGFVNF EKASHHLLDA
251 VSNFRATGST V
```

Matched peptide information:

Start - End	Observed	Mr(expt)	Mr(calc)	ppm	Miss	Sequence
76 - 93	2103.9783	2102.9710	2103.0174	-22	0	K.PSNNPFEPVIQAFNTWSR.K (Ions score 73)
126 - 137	1298.5953	1297.5881	1297.6554	-52	0	K.AITEGGFESLFK.Q (Ions score 58)
151 - 173	2437.1489	2436.1417	2436.1995	-24	0	K.TFACYLSTSTGPVAGTLYLSTAR.V (Ions score 185)
174 - 197	2900.2046	2899.1973	2899.2768	-27	1	R.VAFCSDRPLSFMAPSGQETWCYYK.V (Ions score 12)
221 - 242	2724.1897	2723.1824	2723.2553	-27	0	K.YIQMVTIDGHDFWFMGFVNFEK.A (Ions score 63)
243 - 255	1466.7122	1465.7049	1465.7426	-26	0	K.ASHHLLDAVSNFR.A (Ions score 96)

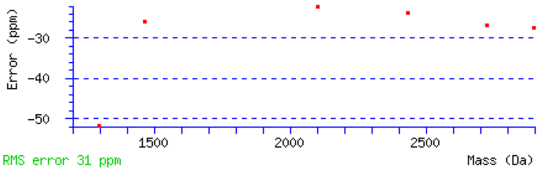

Spot No.:**101**

Accession No.: **scaffold0677_448742.mRNA1**

Protein name：**Proteasome subunit alpha type-5**

Peptide sequences:

R.GVNTFSPEGR.L;R.LFQVEYAIEAIK.L;R.FSYGEPMTVESTTQALCDLALR.F;K.AIGSGSEGADSSLQEQYNK.D;K.DLTLQEAETIALSILK.Q;K.VAPTYHLYTPAEVETVISR.L

PFF Mascot score: **[610]** Sequence coverage %: **[41]**

Matched peptides No.: **[6]**

Calculated Mr: **26165** Calculated *p*I: **4.70**

Data base searched result:

Ions score is -10*Log(P), where P is the probability that the observed match is a random event.
Individual ions scores > 30 indicate identity or extensive homology (p<0.05).
Protein scores are derived from ions scores as a non-probabilistic basis for ranking protein hits.

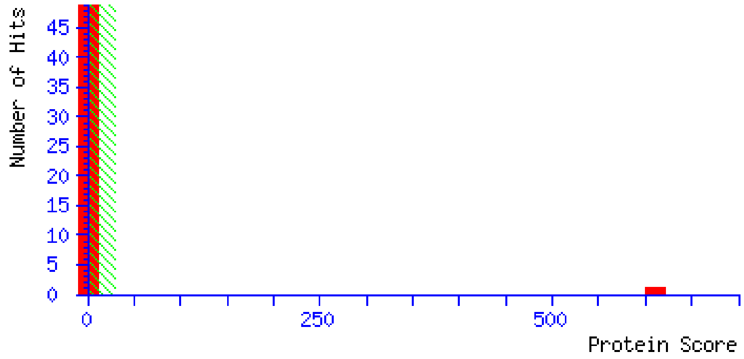

Matched peptide sequences: shown in Bold Red

1 MFLTRTEYDR **GVNTFSPEGR LFQVEYAIEA IK**LGSTAIGL KTKEGVVLAV
51 EKRITSPLLE PSSVEKVMEI DEHIGCAMSG LIADARTLVE HARVETQNHR
101 **FSYGEPMTVE STTQALCDLA LR**FGEGDEES MSRPFGVSLL IAGHDENGPS
151 LYYTDPSGTF WQCNAK**AIGS GSEGADSSLQ EQYNKDLTLQ EAETIALSIL**
201 **K**QVMEEKVTP NNVDIAK**VAP TYHLYTPAEV ETVISR**L

Matched peptide information:

Start - End	Observed	Mr(expt)	Mr(calc)	ppm	Miss	Sequence
11 - 20	1063.4816	1062.4743	1062.5094	-33	0	R.GVNTFSPEGR.L (Ions score 70)
21 - 32	1423.7302	1422.7229	1422.7758	-37	0	R.LFQVEYAIEAIK.L (Ions score 84)
101 - 122	2489.1150	2488.1077	2488.1614	-22	0	R.FSYGEPMTVESTTQALCDLALR.F (Ions score 205)
167 - 185	1940.8285	1939.8212	1939.8759	-28	0	K.AIGSGSEGADSSLQEQYNK.D (Ions score 125)
186 - 201	1757.9296	1756.9223	1756.9822	-34	0	K.DLTLQEAETIALSILK.Q (Ions score 141)
218 - 236	2146.0732	2145.0660	2145.1106	-21	0	K.VAPTYHLYTPAEVETVISR.L (Ions score 134)

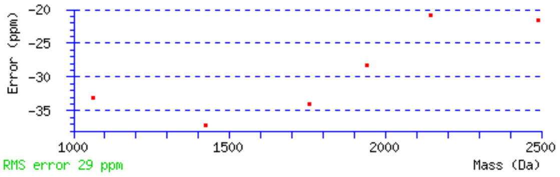

Spot No.:102

Accession No.: scaffold0017_613025.mRNA1

Protein name：Cysteine proteinase inhibitor 12

Peptide sequences:

R.DSQVAPNSVEIDALAR.F;K.ENAVLEFAR.V;K.AKEQVVTGTLHHLTIEAIEAGK.K;K.EQVVTGTLHHLTIEAIEAGK.K;K.VWVKPWLNFK.E;K.HAGDVDGGSGAPSFTSSDLGVK.K;K.EVPAHDPAVQDAASHAVK.T;R.SNSLFPYELK.E

PFF Mascot score: [671]   Sequence coverage %: [43]

Matched peptides No.: [8]

Calculated Mr: 27225   Calculated *p*I: 6.87

Data base searched result:

Ions score is -10*Log(P), where P is the probability that the observed match is a random event.
Individual ions scores > 31 indicate identity or extensive homology (p<0.05).
Protein scores are derived from ions scores as a non-probabilistic basis for ranking protein hits.

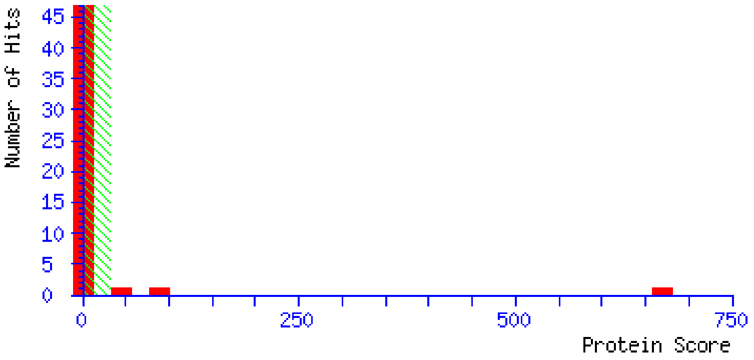

Matched peptide sequences: shown in Bold Red

```
  1 MKRYTSPILS SFSSLLFLLS LISLSHSFVI GAASAFCRDE MTTLGGVRDS
 51 QVAPNSVEID ALARFAVEEH NKKENAVLEF ARVVKAKEQV VTGTLHHLTI
101 EAIEAGKKKL YEAKVWVKPW LNFKELQEFK HAGDVDGGSG APSFTSSDLG
151 VKKDGHGPGW KEVPAHDPAV QDAASHAVKT IQQRSNSLFP YELKEIVHAK
201 AEVVDDHAKF DIILKVKRGT SEEKYKVEVH KNNEGTFLLN QMQPHA
```

Matched peptide information:

Start - End	Observed	Mr(expt)	Mr(calc)	ppm	Miss	Sequence
49 - 64	1684.8329	1683.8256	1683.8428	-10	0	R.DSQVAPNSVEIDALAR.F (Ions score 161)
74 - 82	1048.5207	1047.5135	1047.5349	-20	0	K.ENAVLEFAR.V (Ions score 89)
86 - 107	2345.2593	2344.2520	2344.2750	-10	1	K.AKEQVVTGTLHHLTIEAIEAGK.K (Ions score 44)
88 - 107	2146.1296	2145.1224	2145.1430	-10	0	K.EQVVTGTLHHLTIEAIEAGK.K (Ions score 145)
115 - 124	1316.7244	1315.7171	1315.7441	-21	1	K.VWVKPWLNFK.E (Ions score 66)
131 - 152	2060.9316	2059.9244	2059.9447	-10	0	K.HAGDVDGGSGAPSFTSSDLGVK.K (Ions score 199)
162 - 179	1841.8961	1840.8888	1840.9068	-10	0	K.EVPAHDPAVQDAASHAVK.T (Ions score 104)
185 - 194	1197.5863	1196.5790	1196.6077	-24	0	R.SNSLFPYELK.E (Ions score 69)

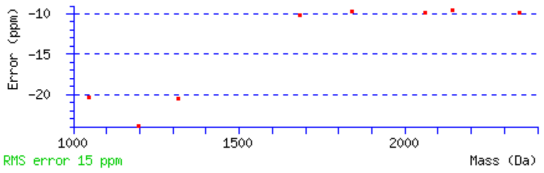

Spot No.: **103**

Accession No.: **scaffold0716_566809.mRNA1**

Protein name：**Proteasome subunit beta type-4**

Peptide sequences:

K.YKDGILMAADMGASYGSTLR.Y;K.DGILMAADMGASYGSTLR.Y;K.FNPLWNSLVLGGVK.N;K.YLGMVSMIGVNFEDNHVATGFGNHLAR.P;R.DEWHENLSFEDGVK.L;K.ITEEGVTISQPYALK.T

PFF Mascot score: **[545]** Sequence coverage %: **[36]**

Matched peptides No.: **[6]**

Calculated Mr: **27797** Calculated *p*I: **6.32**

Data base searched result:

Ions score is -10*Log(P), where P is the probability that the observed match is a random event.
Individual ions scores > 30 indicate identity or extensive homology (p<0.05).
Protein scores are derived from ions scores as a non-probabilistic basis for ranking protein hits.

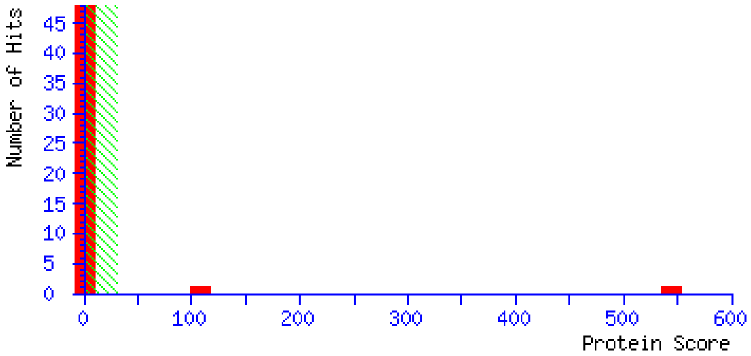

Matched peptide sequences: shown in Bold Red

```
  1 MATTMVKEVG SEPAQLLGPE SASERTLYPY VTGTSVVALK YKDGILMAAD
 51 MGASYGSTLR YKSVERMKSI GKHSLLGASG EISDFQEILH YLDELILYDN
101 MWDDGNSLGP KEVHSYLTRV MYNRRNKFNP LWNSLVLGGV KNGQKYLGMV
151 SMIGVNFEDN HVATGFGNHL ARPILRDEWH ENLSFEDGVK LLEKCMRVLL
201 YRDRSAVNKL QIAKITEEGV TISQPYALKT FWGFSAFQNP TVGAEGSW
```

Matched peptide information:

Start	-	End	Observed	Mr(expt)	Mr(calc)	ppm	Miss	Sequence
41	-	60	2119.9746	2118.9673	2119.0078	-19	1	K.YKDGILMAADMGASYGSTLR.Y (Ions score 181)
43	-	60	1828.8204	1827.8132	1827.8495	-20	0	K.DGILMAADMGASYGSTLR.Y (Ions score 133)
128	-	141	1543.8253	1542.8180	1542.8558	-25	0	K.FNPLWNSLVLGGVK.N (Ions score 62)
146	-	172	2949.3403	2948.3331	2948.4062	-25	0	K.YLGMVSMIGVNFEDNHVATGFGNHLAR.P (Ions score 138)
177	-	190	1704.7125	1703.7052	1703.7427	-22	0	R.DEWHENLSFEDGVK.L (Ions score 120)
215	-	229	1648.8370	1647.8298	1647.8719	-26	0	K.ITEEGVTISQPYALK.T (Ions score 55)

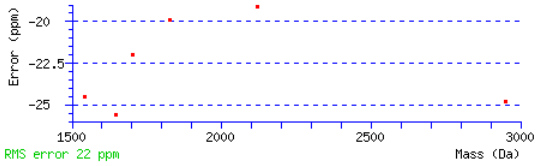

Spot No.:**104**

Accession No.: **scaffold0307_1066072.mRNA1**

Protein name：**Proteasome subunit beta type-2-A**

Peptide sequences:

R.VQFTEYIQK.N;K.NVALYQFR.N;R.NGIPLTTAAAANFTR.G;K.ETGPSLYYIDYIATLHK.I;K.GAFGYGSYFSLSMMDR.H;R.HYHSGMTVEEAIDLVDK.C;R.LVVAPPNFVIK.I

PFF Mascot score: **[529]** Sequence coverage %: **[45]**

Matched peptides No.: **[7]**

Calculated Mr: **22726** Calculated *p*I: **5.85**

Data base searched result:

Ions score is -10*Log(P), where P is the probability that the observed match is a random event.
Individual ions scores > 31 indicate identity or extensive homology (p<0.05).
Protein scores are derived from ions scores as a non-probabilistic basis for ranking protein hits.

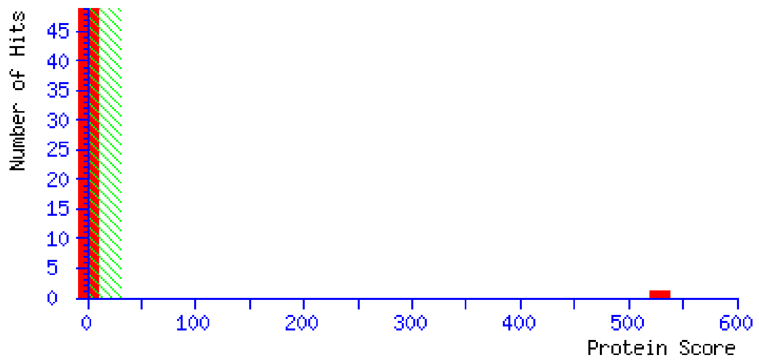

Matched peptide sequences: shown in Bold Red

```
  1 MECVFGLVGD GFALVVADTS AVHSILVHKS NEDKIMVLDS HKLLGASGES
 51 GDRVQFTEYI QKNVALYQFR NGIPLTTAAA ANFTRGELAT ALRKNPYFVN
101 ILMAGYDKET GPSLYYIDYI ATLHKIDKGA FGYGSYFSLS MMDRHYHSGM
151 TVEEAIDLVD KCIIEIRSRL VVAPPNFVIK IVDKDGAREY AWRESVKDTS
201 GAPTA
```

Matched peptide information:

Start - End	Observed	Mr(expt)	Mr(calc)	ppm	Miss	Sequence
54 - 62	1155.5731	1154.5658	1154.5972	-27	0	R.VQFTEYIQK.N (Ions score 65)
63 - 70	1010.5258	1009.5185	1009.5345	-16	0	K.NVALYQFR.N (Ions score 59)
71 - 85	1517.7859	1516.7786	1516.7998	-14	0	R.NGIPLTTAAAANFTR.G (Ions score 100)
109 - 125	1983.9711	1982.9638	1982.9989	-18	0	K.ETGPSLYYIDYIATLHK.I (Ions score 116)
129 - 144	1788.7501	1787.7428	1787.7647	-12	0	K.GAFGYGSYFSLSMMDR.H (Ions score 145)
145 - 161	1943.8798	1942.8725	1942.9095	-19	0	R.HYHSGMTVEEAIDLVDK.C (Ions score 146)
170 - 180	1196.7009	1195.6937	1195.7329	-33	0	R.LVVAPPNFVIK.I (Ions score 82)

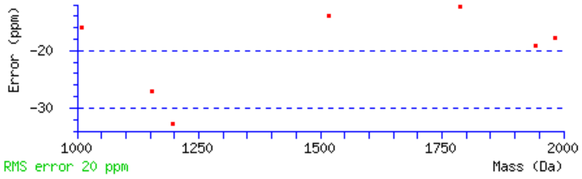

Spot No.:105

Accession No.: scaffold0356_425272.mRNA1

Protein name：DnaJ protein homolog

Peptide sequences:

K.ELAHAYEVLSDPEKR.D;R.DIYDQYGEDALK.E;R.QIGLGMIQQMQHICPECR.G;K.IVFEGQADEAPDTVTGDIVFVLQLR.D;R.TLNLTEALCGFQFVLTHLDGR.Q;K.LYIHFNVDFPDSR.I;R.ILSPEQCHTIETILPLR.S

PFF Mascot score: [499] Sequence coverage %: [17]

Matched peptides No.: [7]

Calculated Mr: 77568 Calculated *p*I: 6.04

Data base searched result:

Ions score is -10*Log(P), where P is the probability that the observed match is a random event.
Individual ions scores > 30 indicate identity or extensive homology (p<0.05).
Protein scores are derived from ions scores as a non-probabilistic basis for ranking protein hits.

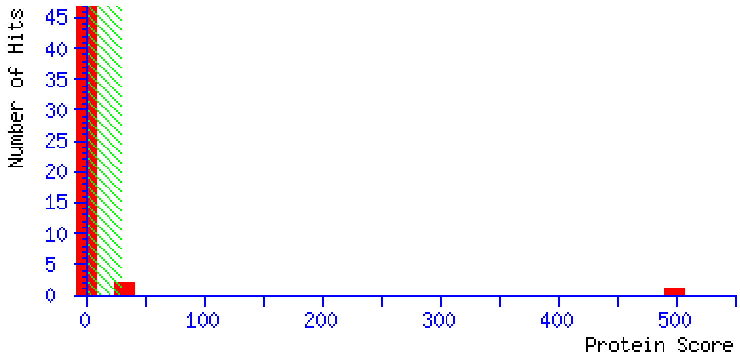

Matched peptide sequences: shown in Bold Red

```
  1 MFGYGGRSDN TKYYEILGVS KNASQDEMKK AYKKAAIKNH PDKGGDPEKF
 51 KELAHAYEVL SDPEKRDIYD QYGEDALKEG MGGGGGSGVH NPFDIFESLF
101 GRSFGGGGSS RGRRQKRGDD VVQTLKVTLE DLYNGTTKKL SLSRNFMCTK
151 CKGKGSKSGA SGMCYGCQGS GMKITTRQIG LGMIQQMQHI CPECRGSGEV
201 ISEKDKCPQC RGKKVSQEKK VLEVHVEKGM HHGQKIVFEG QADEAPDTVT
251 GDIVFVLQLR DHPKFERKVD DLIVERTLNL TEALCGFQFV LTHLDGRQLL
301 IKSNPGEVIK PGQYKAINDE GMPQHGRPFM KGKLYIHFNV DFPDSRILSP
351 EQCHTIETIL PLRSRKHLSD MELDECEETI LHDVNMAEEE KRRKQQQRYE
401 AYEYDEDDEP SMSGSICFIA CAFELSQMIC YFALVDIYLP LICFRSDVLR
451 RLEFVFHSQA SQMENHINLE IDAQGINFPA SSSTTSRSSS SSFSSSSSSI
501 TFSCSKTEDN SLAAPKESRK AQELENGNDE SSKRQKTDVE NGNNNKHPTY
551 RGVRMRSWGK WVSEIREPRK KSRIWLGTYP TAEMAARAHD VAALAIKGSS
601 AFLNFPELAH ELPRPATKSP KDIQAAAAKA ATAAFPARAQ CQAEAEHISN
651 TLVLDNTQES TSFPSADSDD TLFDLPDLFI DDLLDIY
```

Matched peptide information:

Start	-	End	Observed	Mr(expt)	Mr(calc)	ppm	Miss	Sequence	
52	-	66	1756.8960	1755.8887	1755.8791	5	1	K.ELAHAYEVLSDPEKR.D	(Ions score 80)
67	-	78	1429.6495	1428.6423	1428.6409	1	0	R.DIYDQYGEDALK.E	(Ions score 74)
178	-	195	2199.0308	2198.0235	2198.0217	1	0	R.QIGLGMIQQMQHICPECR.G	(Ions score 65)
236	-	260	2732.4016	2731.3943	2731.4069	-5	0	K.IVFEGQADEAPDTVTGDIVFVLQLR.D	(Ions score 145)
277	-	297	2405.2251	2404.2178	2404.2209	-1	0	R.TLNLTEALCGFQFVLTHLDGR.Q	(Ions score 106)
334	-	346	1622.7983	1621.7911	1621.7889	1	0	K.LYIHFNVDFPDSR.I	(Ions score 96)
347	-	363	2020.0894	2019.0821	2019.0823	-0	0	R.ILSPEQCHTIETILPLR.S	(Ions score 94)

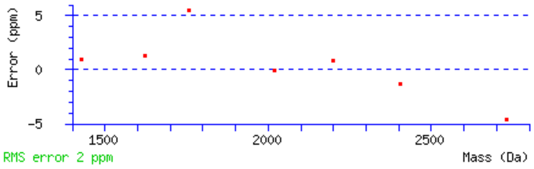

Spot No.:**106**

Accession No.: **scaffold0178_730225.mRNA1**

Protein name： **Probable aldo-ketoreductase 2**

Peptide sequences:

K.RLDVDCIDLYYQHR.V;R.LDVDCIDLYYQHR.V;R.AHAVHPITAVQLEWSLWSR.D;R.DVEEEIVPTCR.E

PFF Mascot score: **[250]** Sequence coverage %: **[12]**

Matched peptides No.: **[4]**

Calculated Mr: **37976** Calculated *p*I: **6.03**

Data base searched result:

Ions score is -10*Log(P), where P is the probability that the observed match is a random event.
Individual ions scores > 30 indicate identity or extensive homology (p<0.05).
Protein scores are derived from ions scores as a non-probabilistic basis for ranking protein hits.

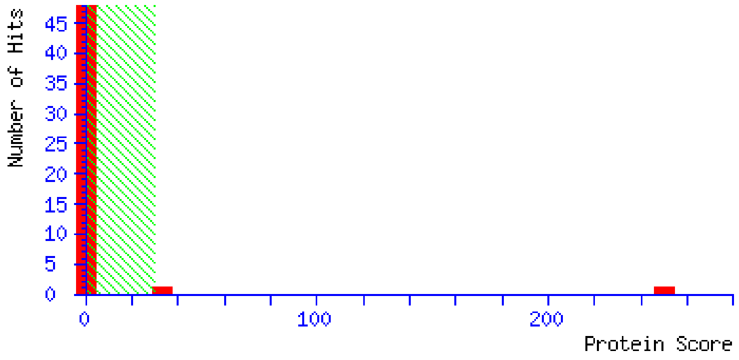

Matched peptide sequences: shown in Bold Red

```
  1 MAVKRIKLGS QGLEVSAQGL GCMGMSAFYG PPKPESDMIA LIHHAINTGV
 51 TFLDTSDVYG PHTNEILLGK ALQGEMRKRV ELATKFAVSF KDGKREIRGD
101 PAYVRAACEA SLKRLDVDCI DLYYQHRVDT SIPIEVTIGE LKKLVEEGKI
151 KYIGLSEASA STIRRAHAVH PITAVQLEWS LWSRDVEEEI VPTCRELGIG
201 IVAYSPLGRG FLSSGPKLVE TLSEGDFRKY LPRFQSENLE HNKQLFERIN
251 EIAARKQCTP SQLALAWVHH QGDDVCPIPG TTKIENFIQN VGALSVKLTP
301 EDMAELESIA SASAVKGDRY ESNNFTFKDS DTPPLSSWKA V
```

Matched peptide information:

Start - End	Observed	Mr(expt)	Mr(calc)	ppm	Miss	Sequence
114 - 127	1865.9227	1864.9155	1864.8890	14	1	K.RLDVDCIDLYYQHR.V (Ions score 86)
115 - 127	1709.8203	1708.8130	1708.7879	15	0	R.LDVDCIDLYYQHR.V (Ions score 86)
166 - 184	2201.1912	2200.1839	2200.1541	14	0	R.AHAVHPITAVQLEWSLWSR.D (Ions score 95)
185 - 195	1346.6467	1345.6395	1345.6184	16	0	R.DVEEEIVPTCR.E (Ions score 67)

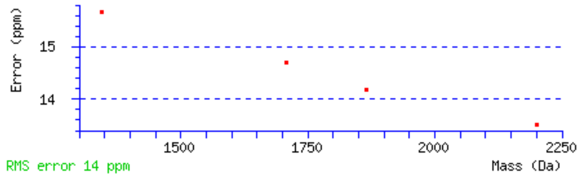

Spot No.:**107**

Accession No.: **scaffold4512_855.mRNA1**

Protein name：**Osmotin-like protein OSM34**

Peptide sequences:

R.NNCPYTVWAAASPGGGR.R;R.LDQGQTWELNVPAGTSMAR.I;K.APGGCNNPCTVFK.T;R.CSDAYSYPQDDPSSTFTCPGGTNYR.V

PFF Mascot score: **[350]** Sequence coverage %: **[30]**

Matched peptides No.: **[4]**

Calculated Mr: **27682** Calculated *p*I: **5.23**

Data base searched result:

Ions score is -10*Log(P), where P is the probability that the observed match is a random event.
Individual ions scores > 31 indicate identity or extensive homology (p<0.05).
Protein scores are derived from ions scores as a non-probabilistic basis for ranking protein hits.

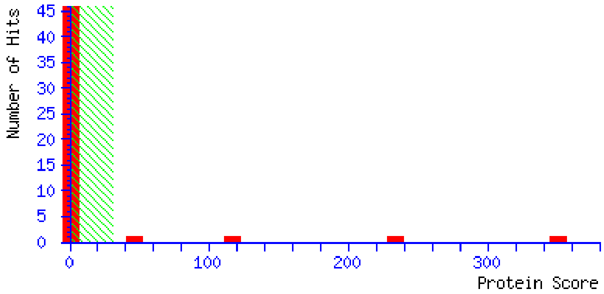

Matched peptide sequences: shown in Bold Red

```
  1 MSNFNIFLIS IFLLSALFFT SSDGATFTIR NNCPYTVWAA ASPGGGRRLD
 51 QGQTWELNVP AGTSMARIWG RTNCNFDGSG KGHCQTGDCG GILACQGWGV
101 PPNTLAEYAL NQFGNLDFYD ISLVDGFNIP IEFSPTSGAK DKCRPLFCTA
151 DINGQCPNQL KAPGGCNNPC TVFKTNEYCC TEGYGTCGPT EFSKFFKSRC
201 SDAYSYPQDD PSSTFTCPGG TNYRVVFCPA RSPHFPLEMV REKDVE
```

Matched peptide information:

Start - End	Observed	Mr(expt)	Mr(calc)	ppm	Miss	Sequence
31 - 47	1777.8057	1776.7984	1776.8002	-1	0	R.NNCPYTVWAAASPGGGR.R (Ions score 135)
49 - 67	2073.9998	2072.9925	2072.9949	-1	0	R.LDQGQTWELNVPAGTSMAR.I (Ions score 108)
162 - 174	1421.6172	1420.6099	1420.6228	-9	0	K.APGGCNNPCTVFK.T (Ions score 55)
200 - 224	2846.1089	2845.1016	2845.1232	-8	0	R.CSDAYSYPQDDPSSTFTCPGGTNYR.V (Ions score 137)

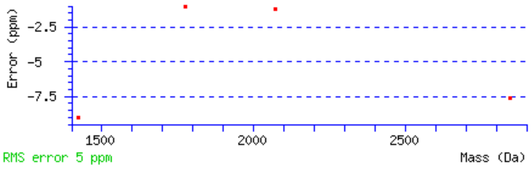

Spot No.:**108**

Accession No.: **scaffold1222_136753.mRNA1**

Protein name： **Rubber elongation factor protein**

Peptide sequences:

K.YLGFVQDAATYAVTTFSNVYLFAK.D;K.NVAVPLYNR.F;K.FVDSTVVASVTIIDR.S;K.DASIQVVSAIR.A

PFF Mascot score: **[312]**   Sequence coverage %: **[42]**

Matched peptides No.: **[4]**

Calculated Mr: **14713**   Calculated *p*I: **5.04**

Data base searched result:

Ions score is -10*Log(P), where P is the probability that the observed match is a random event.
Individual ions scores > 31 indicate identity or extensive homology (p<0.05).
Protein scores are derived from ions scores as a non-probabilistic basis for ranking protein hits.

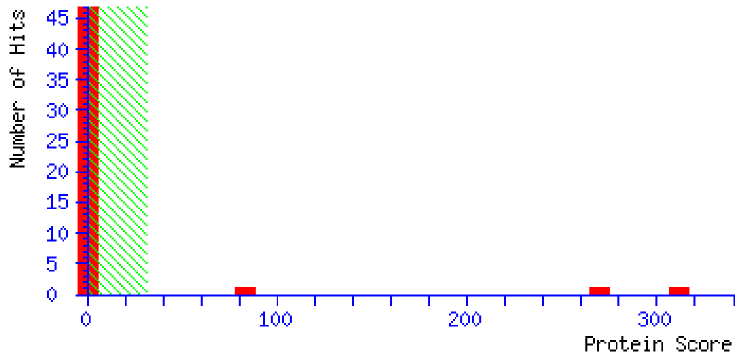

Matched peptide sequences: shown in Bold Red

```
  1 MAEDEDNQQG QGEGLKYLGF VQDAATYAVT TFSNVYLFAK DKSGPLQPGV
 51 DIIEGPVKNV AVPLYNRFSY IPNGALKFVD STVVASVTII DRSLPPIVKD
101 ASIQVVSAIR AAPEAARSLA SSLPGQTKIL AKVFYGEN
```

Matched peptide information:

Start - End	Observed	Mr(expt)	Mr(calc)	ppm	Miss	Sequence
17 - 40	2689.3569	2688.3497	2688.3476	1	0	K.YLGFVQDAATYAVTTFSNVYLFAK.D (Ions score 100)
59 - 67	1045.5713	1044.5640	1044.5716	-7	0	K.NVAVPLYNR.F (Ions score 65)
78 - 92	1621.8794	1620.8721	1620.8723	-0	0	K.FVDSTVVASVTIIDR.S (Ions score 145)
100 - 110	1158.6433	1157.6360	1157.6404	-4	0	K.DASIQVVSAIR.A (Ions score 85)

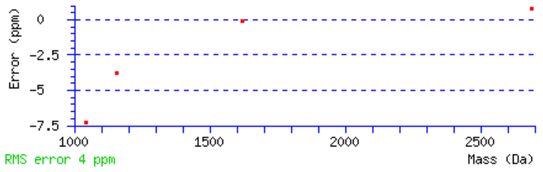

Spot No.:**109**

Accession No.: **scaffold0548_575390.mRNA1**

Protein name： **Stem-specific protein TSJT1**

Peptide sequences:

R.LFAVVDDIFCLFQGHIDNVAVLK.Q;K.TANEVIIVIEAYR.T;R.DRGPYPADQVVR.D;R.GPYPADQVVR.D;K.FAFILYDSSSK.T;K.GCFFTTSGGLR.S;R.SFEHPLNELKPVPR.V

PFF Mascot score: **[373]**   Sequence coverage %: **[33]**

Matched peptides No.: **[7]**

Calculated Mr: **27274**   Calculated *p*I: **5.56**

Data base searched result:

Ions score is -10*Log(P), where P is the probability that the observed match is a random event.
Individual ions scores > 31 indicate identity or extensive homology (p<0.05).
Protein scores are derived from ions scores as a non-probabilistic basis for ranking protein hits.

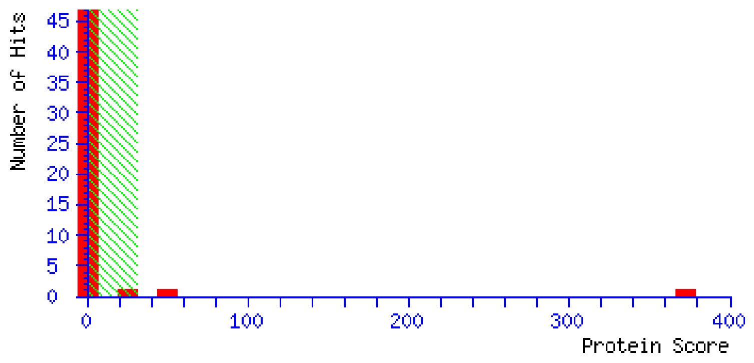

Matched peptide sequences: shown in Bold Red

```
  1 MLAVFDKTVA KCPEALQSPN SGSNSAMKDG FLAKHFGSVH PGSVTVNLGS
 51 AGVIAYSLDK QNPLLPRLFA VVDDIFCLFQ GHIDNVAVLK QQYGLNKTAN
101 EVIIVIEAYR TLRDRGPYPA DQVVRDIQGK FAFILYDSSS KTTFIAADTD
151 GSVPFFWGAD SEDNLVLSDD VQIVQQTCGK SFAPFPKGCF FTTSGGLRSF
201 EHPLNELKPV PRVDSSGQIC GATFKVDAET KKEGGMPRVG SAYDWSSNY
```

Matched peptide information:

Start - End	Observed	Mr(expt)	Mr(calc)	ppm	Miss	Sequence
68 - 90	2633.3994	2632.3921	2632.3724	8	0	R.LFAVVDDIFCLFQGHIDNVAVLK.Q (Ions score 72)
98 - 110	1490.8308	1489.8235	1489.8140	6	0	K.TANEVIIVIEAYR.T (Ions score 121)
114 - 125	1372.6975	1371.6902	1371.6895	1	1	R.DRGPYPADQVVR.D (Ions score 66)
116 - 125	1101.5677	1100.5605	1100.5615	-1	0	R.GPYPADQVVR.D (Ions score 62)
131 - 141	1277.6437	1276.6364	1276.6339	2	0	K.FAFILYDSSSK.T (Ions score 58)
188 - 198	1202.5636	1201.5563	1201.5550	1	0	K.GCFFTTSGGLR.S (Ions score 62)
199 - 212	1662.9117	1661.9045	1661.8889	9	1	R.SFEHPLNELKPVPR.V (Ions score 107)

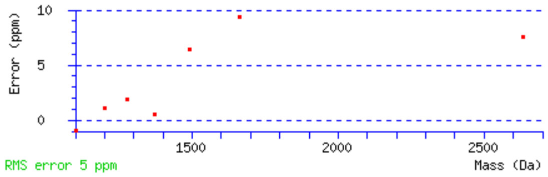

Spot No.:**110**

Accession No.: **scaffold0139_994147.mRNA1**

Protein name： **Glucose and ribitol dehydrogenase**

Peptide sequences:

R.FPPQTQDR.Q;K.AEGAKDPIAIPTDVGFEENCR.S;K.DPIAIPTDVGFEENCR.S;R.TNIFGQFFMTR.Y

PFF Mascot score: **[154]** Sequence coverage %: **[13]**

Matched peptides No.: **[4]**

Calculated Mr: **32309** Calculated *p*I: **5.29**

Data base searched result:

Ions score is -10*Log(P), where P is the probability that the observed match is a random event.
Individual ions scores > 31 indicate identity or extensive homology (p<0.05).
Protein scores are derived from ions scores as a non-probabilistic basis for ranking protein hits.

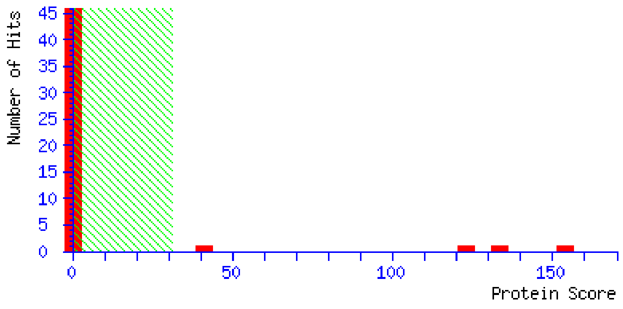

Matched peptide sequences: shown in **Bold Red**

```
  1 MATQSGFRFP PQTQDRQPGK EYLMHPLPEF LNPQYKPSNK LLGKVALVTG
 51 GDSGIGRAVS YYFTLEGATV AFTYVKGQED MDTDHTLKIL SESKAEGAKD
101 PIAIPTDVGF EENCRSVVDQ VMAEYGHIDI LVNNAAEQYY STTIEDITET
151 RLERVFRTNI FGQFFMTRYA LQHMKQGSCI INTTSVVAYA GYSGLLDYSS
201 TKGAIVAFTR SLSLQLIDKG IRVNAVAPGP VWTPLQPASL PAEKIAALGS
251 EVPMDRAAQP YEIAPSYVFL ASNECSSYIT GQVLHPNGGY IVNA
```

Matched peptide information:

Start - End	Observed	Mr(expt)	Mr(calc)	ppm	Miss	Sequence
9 - 16	988.4888	987.4815	987.4774	4	0	R.FPPQTQDR.Q (Ions score 49)
95 - 115	2289.1182	2288.1109	2288.0743	16	1	K.AEGAKDPIAIPTDVGFEENCR.S (Ions score 64)
100 - 115	1832.8770	1831.8697	1831.8411	16	0	K.DPIAIPTDVGFEENCR.S (Ions score 85)
158 - 168	1361.6887	1360.6814	1360.6598	16	0	R.TNIFGQFFMTR.Y (Ions score 39)

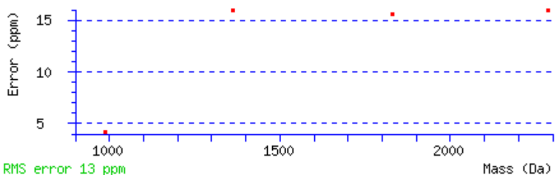

Spot No.:**111**

Accession No.: **scaffold0100_712967.mRNA1**

Protein name: **Patatin-like protein 2**

Peptide sequences:

K.ITVLSIDGGGIR.G;K.LQDLDGPDAR.I;K.DIKDFYLENCPK.I;R.DTYDPIHSIGPIYDGEYLR.E;K.LLLPVIFSSDDAK.C;R.LADVCISTSAAPVLLPAHSFTTEDDK.N ;K.SLDCEDYYLR.I;R.IQDDTLTGEESSGHIATEENLQR.L

PFF Mascot score: **[539]** Sequence coverage %: **[28]**

Matched peptides No.: **[8]**

Calculated Mr: **49142** Calculated *p*I: **5.13**

Data base searched result:

Ions score is -10*Log(P), where P is the probability that the observed match is a random event.
Individual ions scores > 31 indicate identity or extensive homology (p<0.05).
Protein scores are derived from ions scores as a non-probabilistic basis for ranking protein hits.

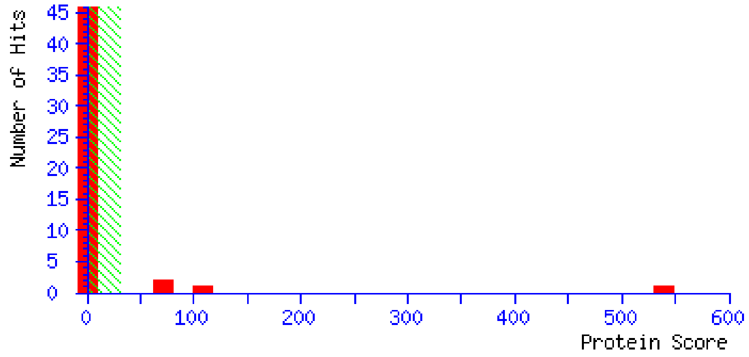

Matched peptide sequences: shown in Bold Red

```
  1 MCLKSMLDIH DVTVHASTIL YIPKLHAQLH SHSQLVLINL LYEDRELSSL
 51 DSNMATGSTT LTQGKKITVL SIDGGGIRGI IPGIILASLE SKLQDLDGPD
101 ARIADYFDII AGTSTGGLIT TMLTAPNEDK KPIYQAKDIK DFYLENCPKI
151 FPKESRDTYD PIHSIGPIYD GEYLRELCNN LLKDLTVKDT LTDVIIPAFD
201 IKLLLPVIFS SDDAKCNALK NARLADVCIS TSAAPVLLPA HSFTTEDDKN
251 IHTFELIDGG VAATNPTLLA LTHIRNEIIR QNPRFIGANL TESKSRLVLS
301 LGTGKSEYKE KYNADMTSKW RLYNWALYNG NSPAVDIFSN ASSDMVDFHL
351 SALFKSLDCE DYYLRIQDDT LTGEESSGHI ATEENLQRLV EIGTELLEKQ
401 ESRINLDTGR LESIPGAPTN EAAIAKFAKL LSEERKLRQL K
```

Matched peptide information:

Start - End	Observed	Mr(expt)	Mr(calc)	ppm	Miss	Sequence
67 - 78	1200.7044	1199.6971	1199.6874	8	0	K.ITVLSIDGGGIR.G (Ions score 100)
93 - 102	1099.5477	1098.5405	1098.5305	9	0	K.LQDLDGPDAR.I (Ions score 58)
138 - 149	1541.7451	1540.7378	1540.7232	10	1	K.DIKDFYLENCPK.I (Ions score 92)
157 - 175	2224.0901	2223.0828	2223.0484	15	0	R.DTYDPIHSIGPIYDGEYLR.E (Ions score 108)
203 - 215	1417.8004	1416.7931	1416.7864	5	0	K.LLLPVIFSSDDAK.C (Ions score 55)
224 - 249	2757.5417	2756.5345	2757.3531	-297	0	R.LADVCISTSAAPVLLPAHSFTTEDDK.N (Ions score 20)
356 - 365	1333.5887	1332.5815	1332.5656	12	0	K.SLDCEDYYLR.I (Ions score 67)
366 - 388	2543.2258	2542.2186	2542.1783	16	0	R.IQDDTLTGEESSGHIATEENLQR.L (Ions score 236)

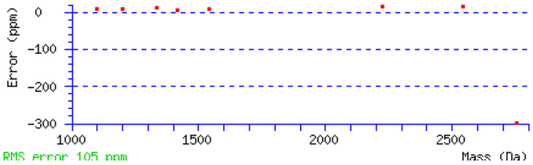

Spot No.:**112**

Accession No.: **scaffold0031_1390956.mRNA1**

Protein name：**Malate dehydrogenase, cytoplasmic**

Peptide sequences:

R.VLVTGAAGQIGYALVPMIAR.G;R.ELIKDDEWLNAEFITTVQQR.G;
K.GVVATTDVVEACTGVNIAVMVGGFPR.K;K.IVQGLHIDEFSR.K

PFF Mascot score: **[407]** Sequence coverage %: **[23]**

Matched peptides No.: **[4]**

Calculated Mr: **36173** Calculated *p*I: **6.19**

Data base searched result:

Ions score is -10*Log(P), where P is the probability that the observed match is a random event.
Individual ions scores > 30 indicate identity or extensive homology (p<0.05).
Protein scores are derived from ions scores as a non-probabilistic basis for ranking protein hits.

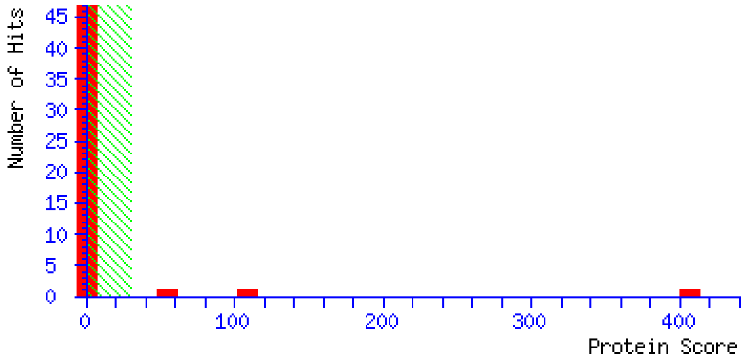

Matched peptide sequences: shown in Bold Red

```
  1 MAKETVRVLV TGAAGQIGYA LVPMIARGVM LGPDQPVILH MLDIPPAAEA
 51 LNGVKMELVD AAFPLLKGVV ATTDVVEACT GVNIAVMVGG FPRKEGMERK
101 DVMSKNVSIY RSQASALEKH AAANCKVLVV ANPANTNALI LKEFAPSIPE
151 KNITCLTRLD HNRALGQISE RLNVQVSDVK NVIIWGNHSS TQYPDVNHAT
201 VKTPSGEKSV RELIKDDEWL NAEFITTVQQ RGAAIIKARK LSSALSAASS
251 ACDHIRDWVL GTPEGTWVSM GVYSDGSYNV PAGLIYSFPV TCQNGEWKIV
301 QGLHIDEFSR KKLDLTADEL SEEKALAYSC LS
```

Matched peptide information:

Start - End	Observed	Mr(expt)	Mr(calc)	ppm	Miss	Sequence
8 - 27	2000.1635	1999.1562	1999.1288	14	0	R.VLVTGAAGQIGYALVPMIAR.G (Ions score 68)
68 - 93	2619.3621	2618.3548	2618.3197	13	0	K.GVVATTDVVEACTGVNIAVMVGGFPR.K (Ions score 158)
212 - 231	2448.2739	2447.2667	2447.2332	14	1	R.ELIKDDEWLNAEFITTVQQR.G (Ions score 169)
299 - 310	1413.7676	1412.7603	1412.7412	14	0	K.IVQGLHIDEFSR.K (Ions score 100)

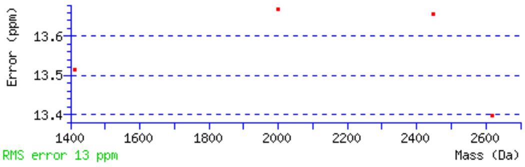

Spot No.:**113**

Accession No.: **scaffold0558_100711.mRNA1**

Protein name：**ATP-citrate synthase alpha chain protein 2**

Peptide sequences:

K.SAQVTESTDFNELAEKEPWLLSGK.L;K.SGLVALNLDLAEVAVFVK.E;K.GPITTFIVEPFIPHNEEFYLNIVSER.L;K.WGNIGFPMPFGR.V;R.VMSSTESFIHGLDEK.T;R.ALVIGGGIANFTDVAATFNGIIR.A;R.SLGEEIGLPIEVYGPEATMTSICK.Q

PFF Mascot score: **[479]**　　Sequence coverage %: **[27]**

Matched peptides No.: **[7]**

Calculated Mr: **57297**　　Calculated *p*I: **5.89**

Data base searched result:

Ions score is -10*Log(P), where P is the probability that the observed match is a random event.
Individual ions scores > 30 indicate identity or extensive homology (p<0.05).
Protein scores are derived from ions scores as a non-probabilistic basis for ranking protein hits.

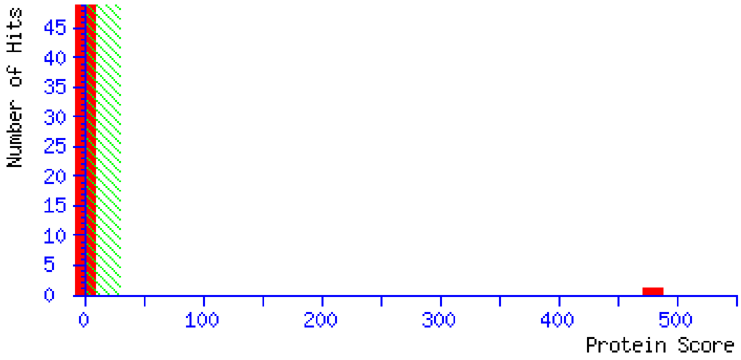

Matched peptide sequences: shown in Bold Red

```
  1 MVGAACGYQQ DMVNKFPRTN IDSEFQTQQT HFLLFSDFLL KISINYAEFL
 51 TINKLLEPHQ IQVFVLLVVA LKLPNPLIFP LSFSANGKQM ARKKIREYDS
101 KRLLKDHFKR LSGYELPIKS AQVTESTDFN ELAEKEPWLL SGKLVVKPDM
151 LFGKRGKSGL VALNLDLAEV AVFVKERLGK EVEMSGCKGP ITTFIVEPFI
201 PHNEEFYLNI VSERLGCSIS FSDCGGIEIE ENWDKVKTIY VPTGSSFTSE
251 TCAPLVATLP LEIKREIEEF IKSIFALFQD LDFTFLEMNP FTLVNGKPYP
301 LDMRGELDDT AAFKNFKKWG NIGFPMPFGR VMSSTESFIH GLDEKTSASL
351 KFTVLNPKGR IWTMVAGGGA SVIYADTVGD LGYASELGNY AEYSGAPNEE
401 EVLQYARVVI DCATSDPDGR KRALVIGGGI ANFTDVAATF NGIIRALKEK
451 ESKLKAARMH MYVRRGGPNY QKGLAKMRSL GEEIGLPIEV YGPEATMTSI
501 CKQAIECISA AA
```

Matched peptide information:

Start - End	Observed	Mr(expt)	Mr(calc)	ppm	Miss	Sequence
120 - 143	2679.3599	2678.3526	2678.3075	17	1	K.SAQVTESTDFNELAEKEPWLLSGK.L (Ions score 69)
158 - 175	1858.0945	1857.0872	1857.0611	14	0	K.SGLVALNLDLAEVAVFVK.E (Ions score 106)
189 - 214	3061.6116	3060.6043	3060.5597	15	0	K.GPITTFIVEPFIPHNEEFYLNIVSER.L (Ions score 103)
319 - 330	1378.6980	1377.6907	1377.6652	19	0	K.WGNIGFPMPFGR.V (Ions score 74)
331 - 345	1679.8206	1678.8133	1678.7872	16	0	R.VMSSTESFIHGLDEK.T (Ions score 75)
423 - 445	2290.2986	2289.2913	2289.2481	19	0	R.ALVIGGGIANFTDVAATFNGIIR.A (Ions score 140)
479 - 502	2594.3184	2593.3111	2593.2655	18	0	R.SLGEEIGLPIEVYGPEATMTSICK.Q (Ions score 72)

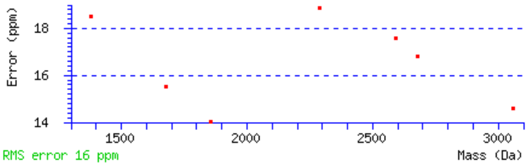

Spot No.:**114**

Accession No.: **scaffold0041_2570834.mRNA1**

Protein name: **Aspartic proteinase A1**

Peptide sequences:

K.NYMDAQYFGEIGIGTPPQK.F;K.FTVIFDTGSSNLWVPSSK.C;K.CYFSVACYFHSR.Y;K.SADIHYGTGAISGFFSQDHVK.V;K.FDGILGLGFEEISVGK.A;K.AVPVWYNMVNQGLVK.E;K.EPVFSFWFNR.N;K.AVVAQYGETIIEMLLAK.D

PFF Mascot score: **[631]** Sequence coverage %: **[21]**

Matched peptides No.: **[8]**

Calculated Mr: **56526** Calculated *p*I: **5.73**

Data base searched result:

Ions score is -10*Log(P), where P is the probability that the observed match is a random event.
Individual ions scores > 30 indicate identity or extensive homology (p<0.05).
Protein scores are derived from ions scores as a non-probabilistic basis for ranking protein hits.

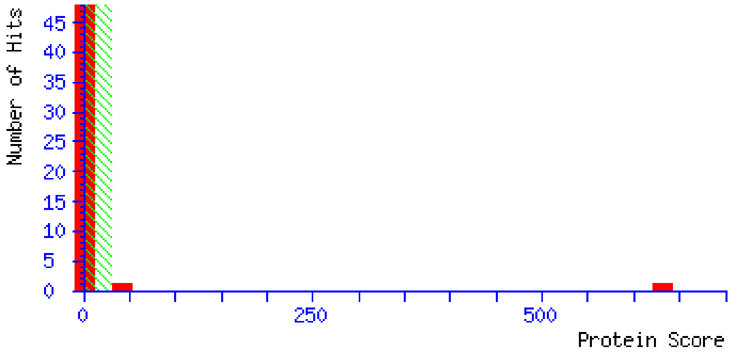

Matched peptide sequences: shown in Bold Red

```
  1 MGTRSKPITT ALFLCFLLLP LVFAAHNDGL VRIGLKKRKF DQNNLVAAQF
 51 ESKEGESLRT SFKKYHFHGN LGDAEDIDII SLKNYMDAQY FGEIGIGTPP
101 QKFTVIFDTG SSNLWVPSSK CYFSVACYFH SRYKSGHSST YKKNGKSADI
151 HYGTGAISGF FSQDHVKVGG LVVKDQEFIE ATKEPSITFL VAKFDGILGL
201 GFEEISVGKA VPVWYNMVNQ GLVKEPVFSF WFNRNADEDE GGEIVFGGMD
251 PKHYKGEHTY VPVTQKGYWQ FNMGDVLIDG KTTGICGSGC AAIADSGTSL
301 LAGPTGIIAE VNHAIGATGI VSQECKAVVA QYGETIIEML LAKDQPQKIC
351 SQIGLCTFDG SRGVSVGIKS VVNENIQGVA SGLRDAMCST CEMAVVWMQN
401 QLKLNNTQER ILNYANELCE RLPSPMGESA VDCGGLSTMP NVSFTIGGKV
451 FDLSPEQYVL KVGEGEAAQC ISGFTALDVP PPRGPLWILG DVFMGRFHTV
501 FDYGNKRVGF AEAA
```

Matched peptide information:

Start - End	Observed	Mr(expt)	Mr(calc)	ppm	Miss	Sequence
84 - 102	2129.0337	2128.0264	2127.9935	15	0	K.NYMDAQYFGEIGIGTPPQK.F (Ions score 133)
103 - 120	1985.0304	1984.0231	1983.9942	15	0	K.FTVIFDTGSSNLWVPSSK.C (Ions score 93)
121 - 132	1596.6991	1595.6918	1595.6650	17	0	K.CYFSVACYFHSR.Y (Ions score 93)
147 - 167	2237.1023	2236.0950	2236.0549	18	0	K.SADIHYGTGAISGFFSQDHVK.V (Ions score 160)
194 - 209	1680.9033	1679.8960	1679.8770	11	0	K.FDGILGLGFEEISVGK.A (Ions score 109)
210 - 224	1717.9290	1716.9217	1716.9021	11	0	K.AVPVWYNMVNQGLVK.E (Ions score 74)
225 - 234	1328.6610	1327.6537	1327.6350	14	0	K.EPVFSFWFNR.N (Ions score 90)
327 - 343	1849.0392	1848.0319	1848.0066	14	0	K.AVVAQYGETIIEMLLAK.D (Ions score 86)

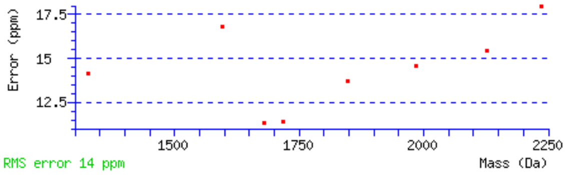

Spot No.:**115**

Accession No.: **scaffold0050_2842364.mRNA1**

Protein name：**Cell division cycle protein 48 homolog**

Peptide sequences:

K.VIETDPAEYCVVAPDTEIFCEGEPVR.R;R.EDENRLDEVGYDDVGGVR.K;R.AHVIVIGATNRPNSIDPALR.R;R.EIDIGVPDEVGR.L;K.DTHGYVGADLAALCTEAALQCIR.E;K.GPELLTMWFGESEANVR.E;R.QSAPCVLFFDELDSIATQR.G;R.LDQLIYIPLPDEDSR.H;K.YTQGFSGADITEICQR.A;K.YQAFAQTLQQSR.G

PFF Mascot score: **[647]** Sequence coverage %: **[22]**

Matched peptides No.: **[10]**

Calculated Mr: **90311** Calculated *p*I: **5.15**

Data base searched result:

Ions score is -10*Log(P), where P is the probability that the observed match is a random event.
Individual ions scores > 30 indicate identity or extensive homology (p<0.05).
Protein scores are derived from ions scores as a non-probabilistic basis for ranking protein hits.

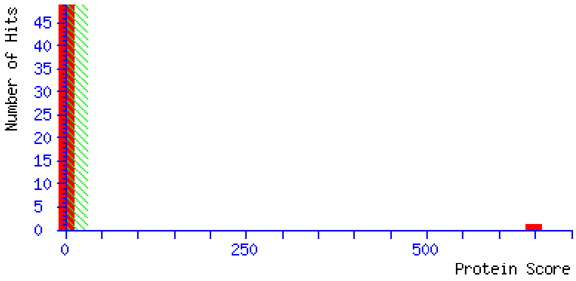

Matched peptide sequences: shown in Bold Red

```
  1 MSNQAESSDS KGTKRDFSTA ILERKKAPNR LVVDEATNDD NSVVSLHPDT
 51 MEKLQLFRGD TILIKGKKRK DTICIALADD TCDEPKIRMN KVVRSNLRVR
101 LGDVVSVHQC PDVKYGKRVH ILPIDDTIEG VTGNLFDAYL KPYFLEAYRP
151 VRKGDLFLVR GGMRSVEFKV IETDPAEYCV VAPDTEIFCE GEPVRREDEN
201 RLDEVGYDDV GGVRKQMAQI RELVELPLRH PQLFKSIGVK PPKGILLYGP
251 PGSGKTLIAR AVANETGAFF FCINGPEIMS KLAGESESNL RKAFEEAEKN
301 APSIIFIDEI DSIAPKREKT HGEVERRIVS QLLTLMDGLK SRAHVIVIGA
351 TNRPNSIDPA LRRFGRFDRE IDIGVPDEVG RLEVLRIHTK NMKLAEDVDL
401 ERIAKDTHGY VGADLAALCT EAALQCIREK MDVIDLEDES IDAEILNSMA
451 VTNEHFQTAL GTSNPSALRE TVVEVPNVSW EDIGGLENVK RELQETVQYP
501 VEHPEKFEKF GMSPSKGVLF YGPPGCGKTL LAKAIANECQ ANFISVKGPE
551 LLTMWFGESE ANVREIFDKA RQSAPCVLFF DELDSIATQR GSSVGDAGGA
601 ADRVLNQLLT EMDGMSAKKT VFIIGATNRP DIIDPALLRP GRLDQLIYIP
651 LPDEDSRHQI FKACLRKSPV SKDVDLRALA KYTQGFSGAD ITEICQRACK
701 YAIRENIEKD IERERRREN PEAMEEDVED DVAEIKAAHF EESMKYARRS
751 VSDADIRKYQ AFAQTLQQSR GFGTEFRFSE ASGAATGTDP FVASAGGADD
801 DDLYN
```

Matched peptide information:

Start - End	Observed	Mr(expt)	Mr(calc)	ppm	Miss	Sequence
170 - 195	2995.3987	2994.3914	2994.3627	10	0	K.VIETDPAEYCVVAPDTEIFCEGEPVR.R (Ions score 120)
197 - 214	2036.9453	2035.9380	2035.9083	15	1	R.EDENRLDEVGYDDVGGVR.K (Ions score 78)
343 - 362	2114.2092	2113.2020	2113.1756	12	1	R.AHVIVIGATNRPNSIDPALR.R (Ions score 53)
370 - 381	1298.6676	1297.6603	1297.6514	7	0	R.EIDIGVPDEVGR.L (Ions score 106)
406 - 428	2505.2202	2504.2129	2504.1788	14	0	K.DTHGYVGADLAALCTEAALQCIR.E (Ions score 102)
548 - 564	1951.9480	1950.9407	1950.9146	13	0	K.GPELLTMWFGESEANVR.E Oxidation (M) (Ions score 92)
572 - 590	2197.0906	2196.0833	2196.0521	14	0	R.QSAPCVLFFDELDSIATQR.G (Ions score 100)
643 - 657	1786.9448	1785.9375	1785.9149	13	0	R.LDQLIYIPLPDEDSR.H (Ions score 107)
682 - 697	1845.8678	1844.8605	1844.8363	13	0	K.YTQGFSGADITEICQR.A (Ions score 73)
759 - 770	1440.7340	1439.7267	1439.7157	8	0	K.YQAFAQTLQQSR.G (Ions score 66)

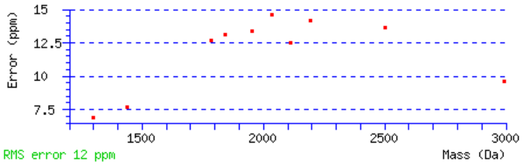

Spot No.:**116**

Accession No.: **scaffold0333_708001.mRNA1**

Protein name: **Natterin-4**

Peptide sequences:

R.YAYYMTESEALR.G;K.WSCTLFEPTLGNDGFLYFR.H;R.TNGVGSWDITSR.V;K.IDCLNAAVSTITTPAR.L;R.LQVEELVFER.Q;R.QINNVIFR.M;K.IEVTAEVTTSLEWNR.T;R.KAEATYQAVVPPMTR.V;R.VTIDYVATQGTCNVPFSYTQR.D;K.IDGTYTGVNYYSFHFR.Q

PFF Mascot score: **[517]** Sequence coverage %: **[30]**

Matched peptides No.: **[10]**

Calculated Mr: **54249** Calculated *p*I: **5.86**

Data base searched result:

Ions score is -10*Log(P), where P is the probability that the observed match is a random event.
Individual ions scores > 31 indicate identity or extensive homology (p<0.05).
Protein scores are derived from ions scores as a non-probabilistic basis for ranking protein hits.

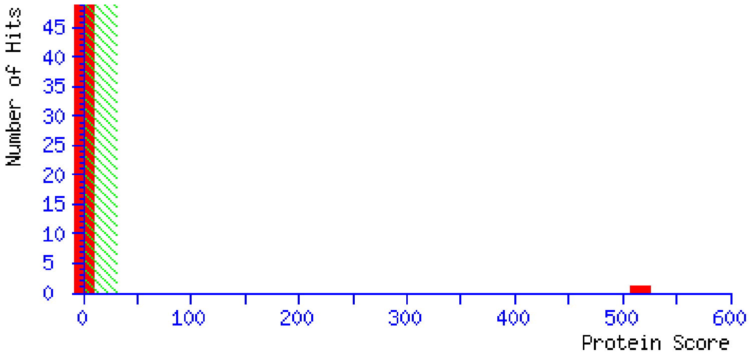

Matched peptide sequences: shown in Bold Red

```
  1 MAHALPKFVV LKSVLSNRYA YYMTESEALR GYVRCQEEEV FSPFAKIEVE
 51 TAKINTRFVH LRFCSNNKYW ARLGADRNDI WIVAKADQPE EDMSKWSCTL
101 FEPTLGNDGF LYFRHVQTGK RVRTNGVGSW DITSRVYVDY SDNGDSTPWY
151 AFTFVDWDTL VIMPKYVTFK GYNDKYLKAG WIERHEYHQF SSDDGNKEET
201 GYEVIMNPDG RLRIKSKFFN KFWRRSPNWI WADSTDTTSN NIDTLFWPVK
251 VNDNTIALRN AGNNRFCSSL TTEGKIDCLN AAVSTITTPA RLQVEELVFE
301 RQINNVIFRM EDARIFDERA VVAGIGSGVN DSPHESLIRV TVSFEDTSSY
351 TFSNSLSIMA GVTTSIQTGF ARIVEGKIEV TAEVTTSLEW NRTTTETRKA
401 EATYQAVVPP MTRVTIDYVA TQGTCNVPFS YTQRDKLSHD GSSLTTQKID
451 GTYTGVNYYS FHFRQVNFET I
```

Matched peptide information:

Start - End	Observed	Mr(expt)	Mr(calc)	ppm	Miss	Sequence
19 - 30	1496.7137	1495.7065	1495.6653	28	0	R.YAYYMTESEALR.G (Ions score 47)
96 - 114	2323.1445	2322.1373	2322.0780	26	0	K.WSCTLFEPTLGNDGFLYFR.H (Ions score 81)
124 - 135	1292.6567	1291.6495	1291.6157	26	0	R.TNGVGSWDITSR.V (Ions score 45)
276 - 291	1702.9293	1701.9220	1701.8720	29	0	K.IDCLNAAVSTITTPAR.L (Ions score 103)
292 - 301	1261.7101	1260.7028	1260.6714	25	0	R.LQVEELVFER.Q (Ions score 74)
302 - 309	1003.5914	1002.5842	1002.5611	23	0	R.QINNVIFR.M (Ions score 48)
378 - 392	1747.9334	1746.9261	1746.8788	27	0	K.IEVTAEVTTSLEWNR.T (Ions score 98)
399 - 413	1661.9148	1660.9075	1660.8607	28	1	R.KAEATYQAVVPPMTR.V (Ions score 84)
414 - 434	2420.2158	2419.2085	2419.1478	25	0	R.VTIDYVATQGTCNVPFSYTQR.D (Ions score 122)
449 - 464	1939.9495	1938.9422	1938.8901	27	0	K.IDGTYTGVNYYSFHFR.Q (Ions score 81)

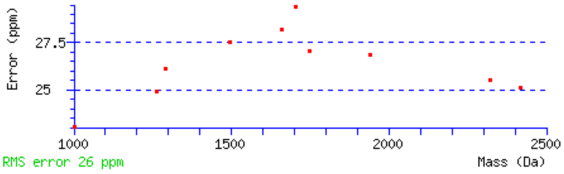

RMS error 26 ppm

Spot No.:117

Accession No.: scaffold1413_46445.mRNA1

Protein name：Cell division cycle protein 48 homolog

Peptide sequences:

K.VIDTDPGEYCVVAPDTEIFCEGEPVR.R;R.ELVELPLR.H;R.AHVIVIGATNRPNSIDPALR.R;R.EIDIGVPDEVGR.L;K.DTHGYVGADLAALCTEAALQCIR.E;K.GPELLTMWFGESEANVR.E;R.QSAPCVLFFDELDSIATQR.G;R.LDQLIYIPLPDEDSR.H;K.YTQGFSGADITEICQR.A;K.YQAFAQTLQQSR.G

PFF Mascot score: **[603]** Sequence coverage %: **[20]**

Matched peptides No.: **[10]**

Calculated Mr: **93897** Calculated *p*I: **5.33**

Data base searched result:

Ions score is -10*Log(P), where P is the probability that the observed match is a random event.
Individual ions scores > 30 indicate identity or extensive homology (p<0.05).
Protein scores are derived from ions scores as a non-probabilistic basis for ranking protein hits.

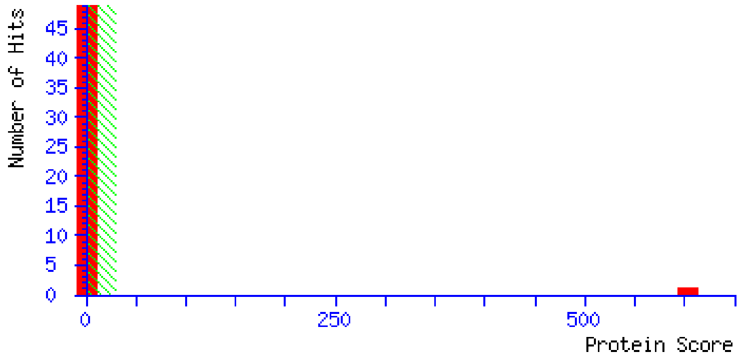

Matched peptide sequences: shown in Bold Red

```
  1 MRTHYRKTSL LLICFAKISP FQYEIPTCTM SHQAESSDSK SAKKDFSTAI
 51 LERKKSPNRL IVDEAINDDN SVVSMHPETM EKLQLFRGDT ILVKGKKRKD
101 TICIALADDT CEPPKIRMNK VVRSNLRVRL GDVVSVHQCP DVKYGKRVHI
151 LPIDDTIEGV TGNLFDAYLK PYFLEAYRPV RKGDLFLVRG GMRSVEFKVI
201 DTDPGEYCVV APDTEIFCEG EPVRREDEDR LDEVGYDDVG GVRKQMAQIR
251 ELVELPLRHP QLFKSIGVKP PKGILLYGPP GSGKTLIARA VANETGAFFF
301 CINGPEIMSK LAGESESNLR KAFEEAEKNA PSIIFIDEID SIAPKREKTH
351 GEVERRIVSQ LLTLMDGLKS RAHVIVIGAT NRPNSIDPAL RRFGRFDREI
401 DIGVPDEVGR LEVLRIHTKN MKLSDEVDLE RIAKDTHGYV GADLAALCTE
451 AALQCIREKM DVIDLEDDSI DAEILNSMAV TNEHFQTALG TSNPSALRET
501 VVEVPNVSWD DIGGLENVKR ELQETVQYPV EHPEKFEKFG MSPSKGVLFY
551 GPPGCGKTLL AKAIANECQA NFISVKGPEL LTMWFGESEA NVREIFDKAR
601 QSAPCVLFFD ELDSIATQRG SSVGDAGGAA DRVLNQLLTE MDGMSAKKTV
651 FIIGATNRPD IIDPALLRPG RLDQLIYIPL PDEDSRHQIF KACLRKSPVS
701 KDVDLRALAK YTQGFSGADI TEICQRACKY AIRENIEKDI EKEKRRRENP
751 EAMDEDVEDE VAEIKAAHFE ESMKFARRSV SDADIRKYQA FAQTLQQSRG
801 FGTEFRFSET RTSGAASDPF TTSAGGAEDD DLYG
```

Matched peptide information:

Start - End	Observed	Mr(expt)	Mr(calc)	ppm	Miss	Sequence
199 - 224	2967.3591	2966.3519	2966.3314	7	0	K.VIDTDPGEYCVVAPDTEIFCEGEPVR.R (Ions score 108)
251 - 258	968.5915	967.5843	967.5702	15	0	R.ELVELPLR.H (Ions score 34)
372 - 391	2114.2146	2113.2073	2113.1756	15	1	R.AHVIVIGATNRPNSIDPALR.R (Ions score 39)
399 - 410	1298.6845	1297.6772	1297.6514	20	0	R.EIDIGVPDEVGR.L (Ions score 92)
435 - 457	2505.2222	2504.2149	2504.1788	14	0	K.DTHGYVGADLAALCTEAALQCIR.E (Ions score 102)
577 - 593	1951.9496	1950.9423	1950.9146	14	0	K.GPELLTMWFGESEANVR.E Oxidation (M) (Ions score 71)
601 - 619	2197.0906	2196.0833	2196.0521	14	0	R.QSAPCVLFFDELDSIATQR.G (Ions score 113)
672 - 686	1786.9530	1785.9457	1785.9149	17	0	R.LDQLIYIPLPDEDSR.H (Ions score 116)
711 - 726	1845.8776	1844.8703	1844.8363	18	0	K.YTQGFSGADITEICQR.A (Ions score 125)
788 - 799	1440.7518	1439.7446	1439.7157	20	0	K.YQAFAQTLQQSR.G (Ions score 60)

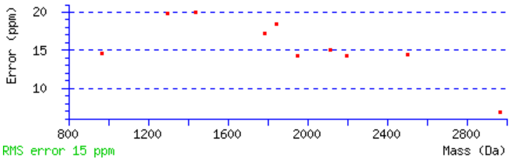

Spot No.:**118**

Accession No.: **scaffold0014_190374.mRNA1**

Protein name：**Cysteine proteinase inhibitor**

Peptide sequences:

K.EVEGSANSVEINSLAR.Y;R.YAVDDYNQK.Q;K.QNALLEFK.K;K.VWEKPWLNFK.E

PFF Mascot score: **[245]** Sequence coverage %: **[42]**

Matched peptides No.: **[4]**

Calculated Mr: **11236** Calculated *p*I: **5.45**

Data base searched result:

Ions score is -10*Log(P), where P is the probability that the observed match is a random event.
Individual ions scores > 31 indicate identity or extensive homology (p<0.05).
Protein scores are derived from ions scores as a non-probabilistic basis for ranking protein hits.

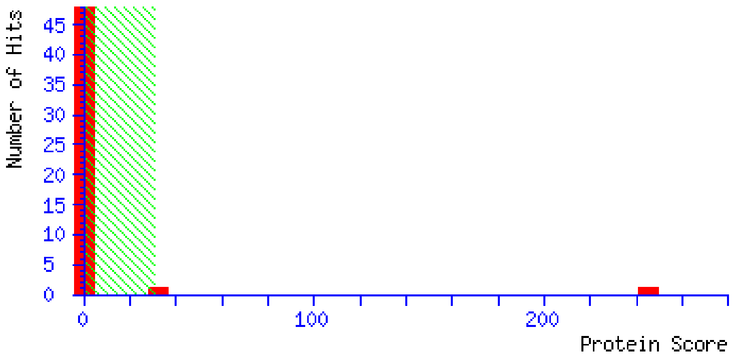

Matched peptide sequences: shown in Bold Red

```
  1 MAKLGGVKEV EGSANSVEIN SLARYAVDDY NQKQNALLEF KKVVNAKQQV
 51 VAGTIYYITL EVTDGGQKKV YEAKVWEKPW LNFKEVQEFK LIGDAPSDST
101 A
```

Matched peptide information:

Start - End	Observed	Mr(expt)	Mr(calc)	ppm	Miss	Sequence
9 - 24	1674.8412	1673.8339	1673.8220	7	0	K.EVEGSANSVEINSLAR.Y (Ions score 158)
25 - 33	1115.4902	1114.4830	1114.4931	-9	0	R.YAVDDYNQK.Q (Ions score 63)
34 - 41	962.5088	961.5015	961.5233	-23	0	K.QNALLEFK.K (Ions score 50)
75 - 84	1346.7189	1345.7116	1345.7183	-5	1	K.VWEKPWLNFK.E (Ions score 69)

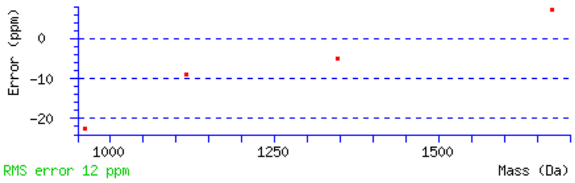

Spot No.:**119**

Accession No.: **scaffold0239_773114.mRNA1**

Protein name： **Probable prefoldin subunit 4**

Peptide sequences:

K.ETNENLEDASNELILADEEVVR.F;R.FQIGEVFAHVPK.E;R.FQIGEVFAHVPKEEVETR.I

PFF Mascot score: **[229]** Sequence coverage %: **[31]**

Matched peptides No.: **[3]**

Calculated Mr: **14561** Calculated *p*I: **4.45**

Data base searched result:

Ions score is -10*Log(P), where P is the probability that the observed match is a random event.
Individual ions scores > 30 indicate identity or extensive homology (p<0.05).
Protein scores are derived from ions scores as a non-probabilistic basis for ranking protein hits.

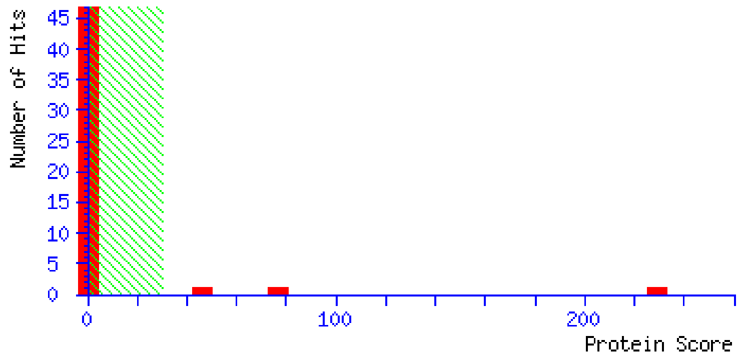

Matched peptide sequences: shown in Bold Red

```
  1 MQQGGGSETE VTWEDQQNIN KFGRLNNRFH ELEDEIKIAK ETNENLEDAS
 51 NELILADEEV VRFQIGEVFA HVPKEEVETR IEQMKEVTSK NLEKLEEEKD
101 SVLAQMAELK KILYGKFGDS INLEED
```

Matched peptide information:

Start - End	Observed	Mr(expt)	Mr(calc)	ppm	Miss	Sequence
41 - 62	2502.1797	2501.1724	2501.1769	-2	0	K.ETNENLEDASNELILADEEVVR.F (Ions score 168)
63 - 74	1371.7278	1370.7205	1370.7347	-10	0	R.FQIGEVFAHVPK.E (Ions score 38)
63 - 80	2115.0898	2114.0826	2114.0797	1	1	R.FQIGEVFAHVPKEEVETR.I (Ions score 79)

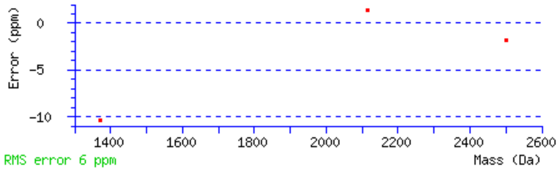

Spot No.:**120**

Accession No.: **scaffold0677_45450.mRNA1**

Protein name：**carboxylesterase 6**

Peptide sequences:

K.LVEEVSGWLR.L;R.LYDDGSVDR.T ;K.FMADPVPAHQDFIDGVATR.D;R.LPAACDDGFSALLWLR.S;R.SSEPWLNDYADFNR.V;R.VFLIGDSSGGNLVHEVAAR.A;R.LAGGIPVHPGFVR.S

PFF Mascot score: **[329]** Sequence coverage %: **[29]**

Matched peptides No.: **[7]**

Calculated Mr: **37612** Calculated *p*I: **5.02**

Data base searched result:

Ions score is -10*Log(P), where P is the probability that the observed match is a random event.
Individual ions scores > 31 indicate identity or extensive homology (p<0.05).
Protein scores are derived from ions scores as a non-probabilistic basis for ranking protein hits.

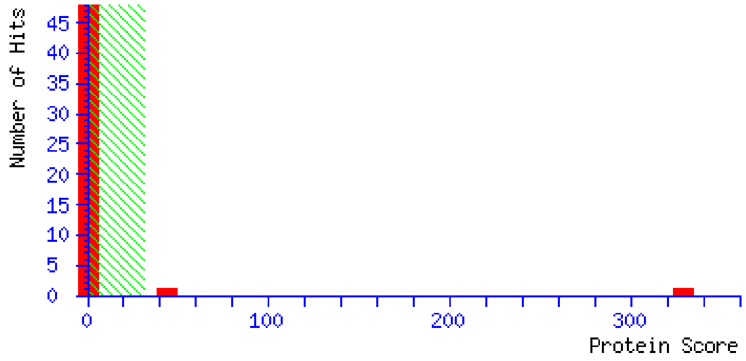

Matched peptide sequences: shown in Bold Red

```
  1 MVRERKLVEE VSGWLRLYDD GSVDRTWTGS PQVKFMADPV PAHQDFIDGV
 51 ATRDVTIDEN SGLRVRIYLP EPNSEDLNKL PVILHFHGGG FCISQADWYM
101 YYNIYTRLAK SVRAICVSVY LRLAPEHRLP AACDDGFSAL LWLRSLAQGR
151 SSEPWLNDYA DFNRVFLIGD SSGGNLVHEV AARAGNVDLS PLRLAGGIPV
201 HPGFVRSERS KSEMEQPESP FLTLDMVDKF LGLALPVGCT KDHPITCPMG
251 TAAPQLDSLN LPPLLLCVAE MDLIKDTEME YYEATKKANK DVELLISPGM
301 GHSFYLNKIA LDMDPPTAEQ TTALIEGIQE FINKH
```

Matched peptide information:

Start - End	Observed	Mr(expt)	Mr(calc)	ppm	Miss	Sequence
7 - 16	1187.6427	1186.6354	1186.6346	1	0	K.LVEEVSGWLR.L (Ions score 54)
17 - 25	1039.4690	1038.4617	1038.4618	-0	0	R.LYDDGSVDR.T (Ions score 47)
35 - 53	2087.0154	2086.0081	2085.9942	7	0	K.FMADPVPAHQDFIDGVATR.D (Ions score 47)
129 - 144	1804.9196	1803.9123	1803.8978	8	0	R.LPAACDDGFSALLWLR.S (Ions score 99)
151 - 164	1713.7648	1712.7575	1712.7430	8	0	R.SSEPWLNDYADFNR.V (Ions score 109)
165 - 183	1941.0314	1940.0241	1940.0116	6	0	R.VFLIGDSSGGNLVHEVAAR.A (Ions score 113)
194 - 206	1319.7634	1318.7562	1318.7510	4	0	R.LAGGIPVHPGFVR.S (Ions score 35)

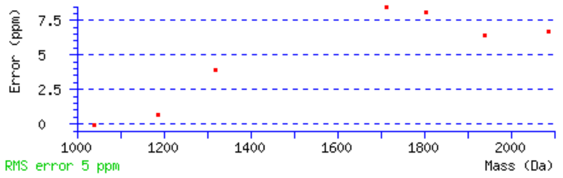

Spot No.:**121**

Accession No.: **scaffold1789_16143.mRNA1**

Protein name：**Cyclase-associated protein 1**

Peptide sequences:

R.GVADFGGADVTVDPSIVAFDDLLGQFFGR.V;R.RSDFFNHLK.S;K.ELYLPGLR.D;K.SHYPLGPTWGVSGK.A;K.APAPPPPPPASLFSAESSQPSSSKPK.E;R.KWVVENQIGR.K;K.WVVENQIGR.K

PFF Mascot score: **[212]** Sequence coverage %: **[20]**

Matched peptides No.: **[7]**

Calculated Mr: **51990** Calculated *p*I: **6.64**

Data base searched result:

Ions score is -10*Log(P), where P is the probability that the observed match is a random event.
Individual ions scores > 31 indicate identity or extensive homology (p<0.05).
Protein scores are derived from ions scores as a non-probabilistic basis for ranking protein hits.

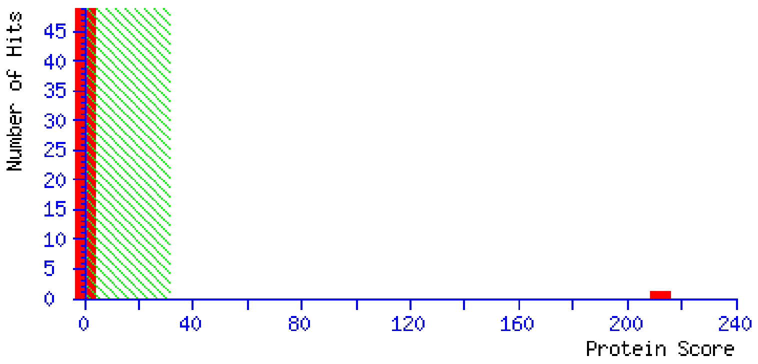

Matched peptide sequences: shown in Bold Red

```
  1 MEEKLISRLE SAVARLEALS VSAFRDRGVA DFGGADVTVD PSIVAFDDLL
 51 GQFFGRVSAA AEKIGGQVLE VTKIVQEAFR VQKELLVKAK QTQKPDLAEL
101 AEFLKPLNEV IMKANAMTEG RRSDFFNHLK SAADSLTALA WIAYTGKDCG
151 MSMPIAHVEE SWQMAEFYNN KILVEYKSKD PNHVEWAKAM KELYLPGLRD
201 YVKSHYPLGP TWGVSGKAPA FAPSKAPAPP PPPPASLFSA ESSQPSSSKP
251 KEGMAAVFQE INTSKSVTAG LRKVTADMKT KNRADRTGVV GTSEKEGRTS
301 SPSFSKAGPP KLELQMGRKW VVENQIGRKN LVIDDCDAKQ SVYVFGCKDS
351 VLQIQGKVNN ITVDKCTKMG VVFTDVVAAC EIVNCSGVEV QCQGSSPTVS
401 VDNTSGCLLY LSKDSLGASI TTAKSSEINV LIPGAESNGD WHRKTFSECI
451 EYKNVRHYFE ILFAFDFYEL SI
```

Matched peptide information:

Start - End	Observed	Mr(expt)	Mr(calc)	ppm	Miss	Sequence
28 - 56	2985.4482	2984.4410	2984.4557	-5	0	R.GVADFGGADVTVDPSIVAFDDLLGQFFGR.V (Ions score 114)
122 - 130	1163.5933	1162.5860	1162.5883	-2	1	R.RSDFFNHLK.S (Ions score 7)
192 - 199	960.5393	959.5320	959.5440	-13	0	K.ELYLPGLR.D (Ions score 49)
204 - 217	1485.7457	1484.7385	1484.7412	-2	0	K.SHYPLGPTWGVSGK.A (Ions score 44)
226 - 251	2559.3098	2558.3025	2558.3016	0	1	K.APAPPPPPPASLFSAESSQPSSSKPK.E (Ions score 47)
319 - 328	1228.6832	1227.6760	1227.6724	3	1	R.KWVVENQIGR.K (Ions score 57)
320 - 328	1100.5826	1099.5754	1099.5774	-2	0	K.WVVENQIGR.K (Ions score 43)

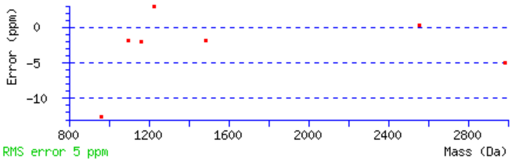

Spot No.:**122**

Accession No.: **scaffold0047_1944984.mRNA1**

Protein name：**SUMO-conjugating enzyme UBC9**

Peptide sequences: **K.EQWSPALTISK.V;K.VLLSICSLLTDPNPDDPLVPEIAHMYK.T**

PFF Mascot score: **[105]** Sequence coverage %: **[25]**

Matched peptides No.: **[2]**

Calculated Mr: **16749** Calculated *p*I: **7.71**

Data base searched result:

Ions score is -10*Log(P), where P is the probability that the observed match is a random event.
Individual ions scores > 30 indicate identity or extensive homology (p<0.05).
Protein scores are derived from ions scores as a non-probabilistic basis for ranking protein hits.

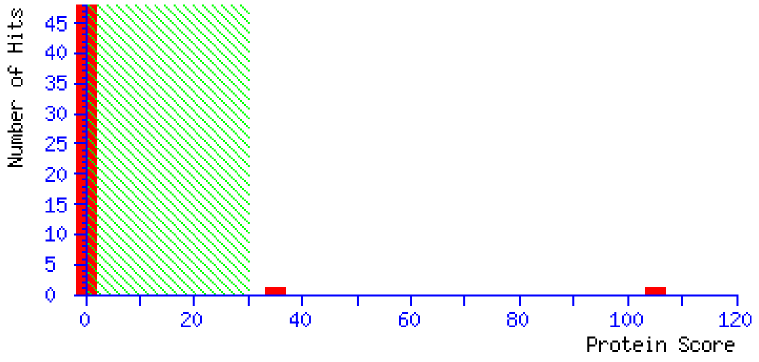

Matched peptide sequences: shown in **Bold Red**

```
  1 MASKRILKEL KDLQKDPPTS CSAGPVAEDM FHWQATIMGP PDSPYAGGVF
 51 LVTIHFPPDY PFKPPKVAFR TKVFHPNINS NGSICLDILK EQWSPALTIS
101 KVLLSICSLL TDPNPDDPLV PEIAHMYKTD RNKYETTART WTQKYAMG
```

Matched peptide information:

Start - End	Observed	Mr(expt)	Mr(calc)	ppm	Miss	Sequence	
91 - 101	1259.6403	1258.6330	1258.6557	-18	0	K.EQWSPALTISK.V	(Ions score 67)
102 - 128	3050.5125	3049.5052	3049.5504	-15	0	K.VLLSICSLLTDPNPDDPLVPEIAHMYK.T	(Ions score 61)

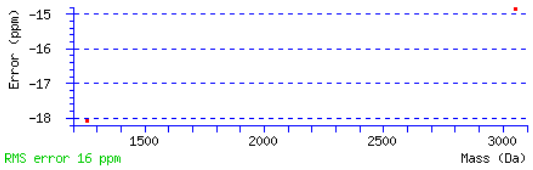

Spot No.:**123**

Accession No.: **scaffold0592_368115.mRNA1**

Protein name： **Ankyrin repeat domain-containing protein 2**

Peptide sequences:

K.SETSSGESQSGQGR.S;R.SPPSSGPGFPPNVFDFSAMSGLLNDPSIK.E;K.DPSFNQMADQLHK.T;R.YWNDKEVLQK.L;K.NAMASGANKDEEDSEGR.T;R.TALHFACGYGEVK.C;K.NTALHYAAGYGR.K

PFF Mascot score: **[341]** Sequence coverage %: **[30]**

Matched peptides No.: **[7]**

Calculated Mr: **37708** Calculated *p*I: **4.50**

Data base searched result:

Ions score is -10*Log(P), where P is the probability that the observed match is a random event.
Individual ions scores > 30 indicate identity or extensive homology (p<0.05).
Protein scores are derived from ions scores as a non-probabilistic basis for ranking protein hits.

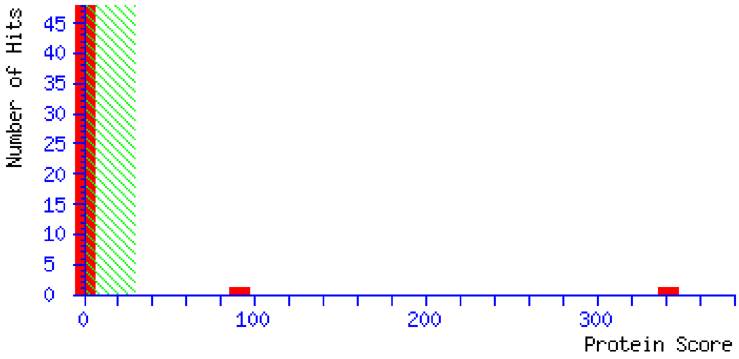

Matched peptide sequences: shown in Bold Red

```
  1 MASNSNKDVP SGEKTGSTET KIPKSETSSG ESQSGQGRSP PSSGPGFPPN
 51 VFDFSAMSGL LNDPSIKELA EQIAKDPSFN QMADQLHKTL QGVGAEDEIP
101 QFDSQQYYST MQQVMQNPQF MAMAERLGSA LMQDPSMSQM LESLANPAQK
151 DQIEERMARI KDDPSLKPIL EEIESGGPAA MMRYWNDKEV LQKLGEAMGL
201 AVSGEATTSV ENPGLDEEEE AGNEDESIVH HCASVGDVEG LKNAMASGAN
251 KDEEDSEGRT ALHFACGYGE VKCAQILLEA GATVDALDKN KNTALHYAAG
301 YGRKECVALL LENGAAVTLQ NMDGKTPIDV AKLNNQHDVL KLLEKDAFL
```

Matched peptide information:

Start - End	Observed	Mr(expt)	Mr(calc)	ppm	Miss	Sequence
25 - 38	1396.6012	1395.5939	1395.5862	6	0	K.SETSSGESQSGQGR.S (Ions score 67)
39 - 67	2962.4026	2961.3953	2961.4219	-9	0	R.SPPSSGPGFPPNVFDFSAMSGLLNDPSIK.E (Ions score 31)
76 - 88	1530.6979	1529.6906	1529.6933	-2	0	K.DPSFNQMADQLHK.T (Ions score 110)
184 - 193	1322.6528	1321.6456	1321.6666	-16	1	R.YWNDKEVLQK.L (Ions score 63)
243 - 259	1780.7436	1779.7364	1779.7329	2	1	K.NAMASGANKDEEDSEGR.T (Ions score 61)
260 - 272	1452.6897	1451.6824	1451.6867	-3	0	R.TALHFACGYGEVK.C (Ions score 87)
292 - 303	1293.6346	1292.6274	1292.6262	1	0	K.NTALHYAAGYGR.K (Ions score 105)

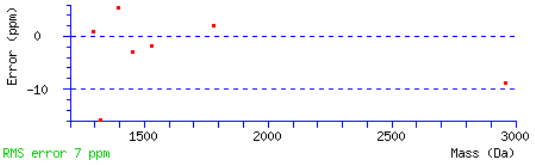

Spot No.:**124**

Accession No.: **scaffold2041_42337.mRNA1**

Protein name：**Probable calcium-binding protein CML49**

Peptide sequences:

K.DKPNSYASPPAPGYYPR.S;R.VLSSCDQSFSLR.T;R.LLMYHFTNTNTR.K

PFF Mascot score: **[205]**   Sequence coverage %: **[14]**

Matched peptides No.: **[3]**

Calculated Mr: **31736**   Calculated *p*I: **7.48**

Data base searched result:

Ions score is -10*Log(P), where P is the probability that the observed match is a random event.
Individual ions scores > 31 indicate identity or extensive homology (p<0.05).
Protein scores are derived from ions scores as a non-probabilistic basis for ranking protein hits.

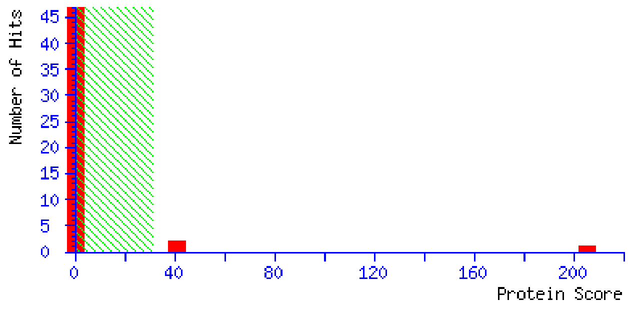

Matched peptide sequences: shown in Bold Red

```
  1 MKSSPQLIIV GKSSGLISIS QKIPDFFCRV IISKLDVDGR DRGIAKPGDD
 51 LMIFTIPILK TPCDAPCSAP PPSDKPPKDK PNSYASPPAP GYYPRSAPYG
101 SPFASLVPSA FPPGTDPNVV AGFRLADQDG SGFIDDKELQ RVLSSCDQSF
151 SLRTVRLLMY HFTNTNTRKI GPKEFTSVYY SLQNWRAMFE RFDKDSSGRI
201 DPNELREALC SLGFAVSPVV LDLLVSKFDK SGGKDKAIEY DNFIECSLTV
251 KGLTEKFREK DCTYSGSATF TYEAFMLTVL PLLAV
```

Matched peptide information:

Start - End	Observed	Mr(expt)	Mr(calc)	ppm	Miss	Sequence	
79 - 95	1879.8685	1878.8613	1878.8900	-15	1	K.DKPNSYASPPAPGYYPR.S	(Ions score 110)
142 - 153	1398.6416	1397.6343	1397.6609	-19	0	R.VLSSCDQSFSLR.T	(Ions score 80)
157 - 168	1510.7184	1509.7111	1509.7398	-19	0	R.LLMYHFTNTNTR.K	(Ions score 67)

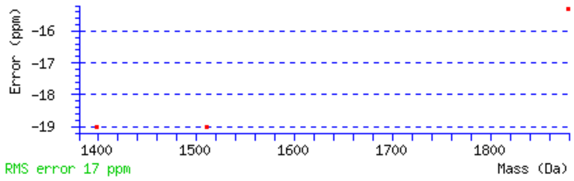

Spot No.:**125**

Accession No.: **scaffold1222_136753.mRNA1**

Protein name: **Rubber elongation factor protein**

Peptide sequences:

K.YLGFVQDAATYAVTTFSNVYLFAK.D;K.DKSGPLQPGVDIIEGPVK.N;K.NVAVPLYNR.F;K.FVDSTVVASV TIIDR.S;K.DASIQVVSAIR.A

PFF Mascot score: **[497]** Sequence coverage %: **[55]**

Matched peptides No.: **[5]**

Calculated Mr: **14713** Calculated *p*I: **5.04**

Data base searched result:

Ions score is -10*Log(P), where P is the probability that the observed match is a random event.
Individual ions scores > 31 indicate identity or extensive homology (p<0.05).
Protein scores are derived from ions scores as a non-probabilistic basis for ranking protein hits.

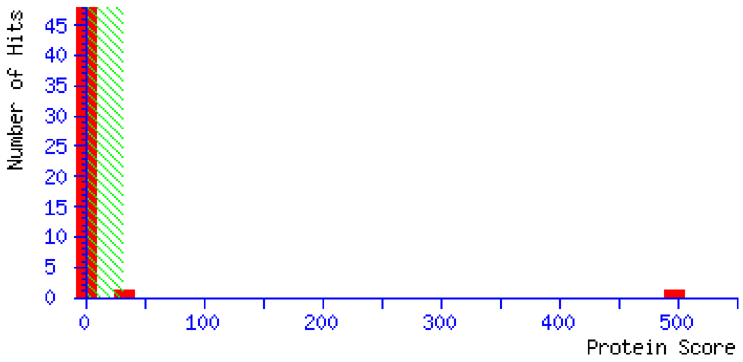

Matched peptide sequences: shown in Bold Red

```
  1 MAEDEDNQQG QGEGLKYLGF VQDAATYAVT TFSNVYLFAK DKSGPLQPGV
 51 DIIEGPVKNV AVPLYNRFSY IPNGALKFVD STVVASVTII DRSLPPIVKD
101 ASIQVVSAIR AAPEAARSLA SSLPGQTKIL AKVFYGEN
```

Matched peptide information:

Start - End	Observed	Mr(expt)	Mr(calc)	ppm	Miss	Sequence
17 - 40	2689.3342	2688.3270	2688.3476	-8	0	K.YLGFVQDAATYAVTTFSNVYLFAK.D (Ions score 173)
41 - 58	1848.9872	1847.9799	1847.9993	-10	1	K.DKSGPLQPGVDIIEGPVK.N (Ions score 140)
59 - 67	1045.5602	1044.5529	1044.5716	-18	0	K.NVAVPLYNR.F (Ions score 65)
78 - 92	1621.8652	1620.8580	1620.8723	-9	0	K.FVDSTVVASVTIIDR.S (Ions score 140)
100 - 110	1158.6335	1157.6263	1157.6404	-12	0	K.DASIQVVSAIR.A (Ions score 104)

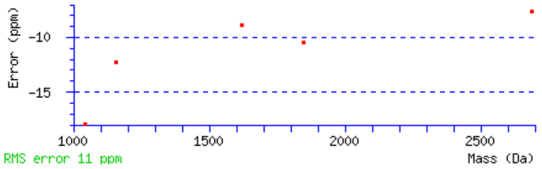

Spot No.:**126**

Accession No.: **scaffold0456_1002067.mRNA1**

Protein name：**Fructokinase-2**

Peptide sequences:

K.APGGAPANVAIAVAR.L;R.NPSADMLLKPEELNLELIR.S;K.VFHYGSISLIVEPCR.S;R.LPLWPSPEEAR.E

PFF Mascot score: **[318]** Sequence coverage %: **[18]**

Matched peptides No.: **[4]**

Calculated Mr: **35625** Calculated *p*I: **5.22**

Data base searched result:

Ions score is -10*Log(P), where P is the probability that the observed match is a random event.
Individual ions scores > 31 indicate identity or extensive homology (p<0.05).
Protein scores are derived from ions scores as a non-probabilistic basis for ranking protein hits.

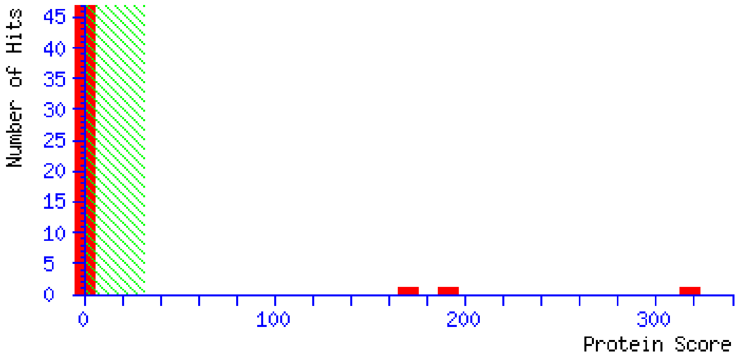

Matched peptide sequences: shown in Bold Red

```
  1 MASVVSNGGS GSSLIVSFGE MLIDFVPTVS GVSLAEAPGF LKAPGGAPAN
 51 VAIAVARLGG KAAFVGKLGD DEFGHMLAGI LKQNGVIGDG INFDKGARTA
101 LAFVTLRADG EREFMFYRNP SADMLLKPEE LNLELIRSAK VFHYGSISLI
151 VEPCRSAHLK AMEVAKDAGA LLSYDPNLRL PLWPSPEEAR EQIMSIWDKA
201 EVIKVSDVEL EFLTGSDKID DADALSLWHP NLKLLLVTLG EKGCRYYTKN
251 FHGSVEAFHV KTVDTTGAGD SFVGALLCKI VDDRSILEDE PRLREVLKFA
301 NACGAITTTK KGAIPALPTE QDALTLIKGS E
```

Matched peptide information:

Start	-	End	Observed	Mr(expt)	Mr(calc)	ppm	Miss	Sequence	
43	-	57	1334.7293	1333.7220	1333.7466	-18	0	K.APGGAPANVAIAVAR.L	(Ions score 100)
119	-	137	2195.1538	2194.1465	2194.1667	-9	1	R.NPSADMLLKPEELNLELIR.S	(Ions score 144)
141	-	155	1776.8922	1775.8849	1775.9029	-10	0	K.VFHYGSISLIVEPCR.S	(Ions score 80)
180	-	190	1294.6565	1293.6492	1293.6717	-17	0	R.LPLWPSPEEAR.E	(Ions score 86)

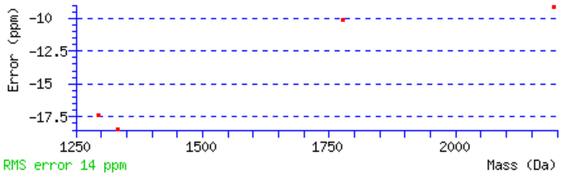

Spot No.:127

Accession No.: scaffold1032_279744.mRNA1

Protein name：Urease accessory protein G

Peptide sequences:

R.VYHSHDGLAPHSHEPIYSPGYFSR.R;R.AVETGGCPHAAIR.E;K.ADLLLCESGGDNLAANFSR.E;K.TDLAPAVGADLAVMER.D;R.DGGPFVFAQIK.H;K.HGLGVEEIVNHILQAWEGATGK.K

PFF Mascot score: [305] Sequence coverage %: [20]

Matched peptides No.: [6]

Calculated Mr: 57303 Calculated *p*I: 7.02

Data base searched result:

Ions score is -10*Log(P), where P is the probability that the observed match is a random event.
Individual ions scores > 31 indicate identity or extensive homology (p<0.05).
Protein scores are derived from ions scores as a non-probabilistic basis for ranking protein hits.

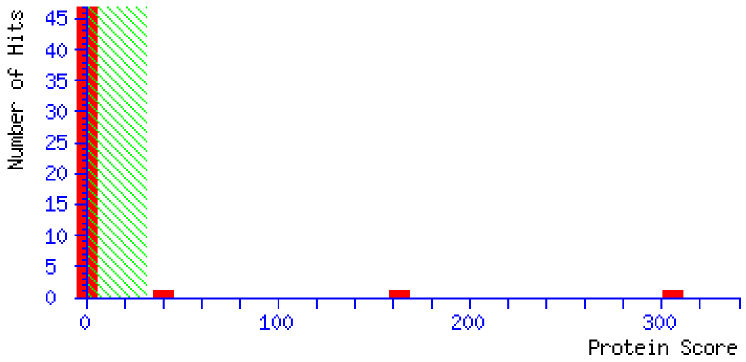

Matched peptide sequences: shown in Bold Red

1 MKSDVALHGV LLWLSMGFLA PLGILIIRMS HREKGGSRGK VFFYLHVILQ
51 ALSVLLVTSG AIMSMKSFEN SFDNNHQRIG VALYGVVWVQ AVIGFLRPHR
101 GSKRRSTWYF LHWMLGTVIT LVGIINTYTG LEAYHKKISA NTRIWTIIFT
151 AQVSFMAFFY LFQDKWEYMQ KQGVILGNIE PIMTPTTITT QSDTQKIGGN
201 NSLSNQSEEN KNLIKLEIRA TKVIHSIEQN LCGLELMASH GHHTHDYHHH
251 DHDNDHHHHT HDQTTSWVGP EGR**VYHSHDG** **LAPHSHEPIY** **SPGYFSR**RAP
301 PILTRDFNER AFTVGIGGPV GTGKTALMLA ICKFLRDKYS LAAVTNDIFT
351 KEDGEFLIKH GALPEERIR**A** **VETGGCPHAA** **IR**EDISINLG PLEELSKLFK
401 **ADLLLCESGG** **DNLAANFSR**E LADYIIYIID VSGGDKIPRK GGPGITQADL
451 LVINK**TDLAP** **AVGADLAVME** **R**DALRIR**DGG** **PFVFAQIKHG** **LGVEEIVNHI**
501 **LQAWEGATGK** KQH

Matched peptide information:

Start - End	Observed	Mr(expt)	Mr(calc)	ppm	Miss	Sequence
274 - 297	2753.3357	2752.3284	2752.2782	18	0	R.VYHSHDGLAPHSHEPIYSPGYFSR.R (Ions score 75)
370 - 382	1338.6998	1337.6926	1337.6510	31	0	R.AVETGGCPHAAIR.E (Ions score 42)
401 - 419	2023.0054	2021.9981	2021.9476	25	0	K.ADLLLCESGGDNLAANFSR.E (Ions score 157)
456 - 471	1628.8639	1627.8566	1627.8239	20	0	K.TDLAPAVGADLAVMER.D (Ions score 117)
478 - 488	1178.6266	1177.6193	1177.6132	5	0	R.DGGPFVFAQIK.H (Ions score 24)
489 - 510	2358.2441	2357.2369	2357.2128	10	0	K.HGLGVEEIVNHILQAWEGATGK.K (Ions score 20)

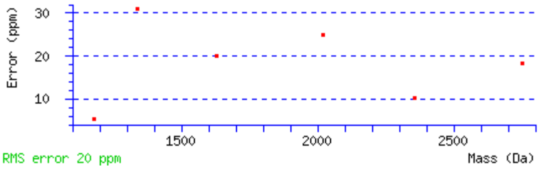

Spot No.:**128**

Accession No.: **scaffold0005_28735.mRNA1**

Protein name: **S-adenosylmethionine synthase 2**

Peptide sequences:

K.VLVYIEQQSPDIAQGVHGHLTK.R;K.NGTCPWLRPDGK.T;K.TQVTVEYYNDNGAMVPVR.V;R.VHTVLISTQHDETVTNDEIAADLK.E;K.TIFHLNPSGR.F;R.FVIGGPHGDAGLTGR.K;K.IIIDTYGGWGAHGGGAFSGK.D;K.ENFDFRPGMISINLDLK.R;K.TAAYGHFGR.D

PFF Mascot score: **[520]**

Sequence coverage %: **[37]**

Matched peptides No.: **[9]**

Calculated Mr: **43540**

Calculated *p*I: **5.59**

Data base searched result:

Ions score is -10*Log(P), where P is the probability that the observed match is a random event.
Individual ions scores > 31 indicate identity or extensive homology (p<0.05).
Protein scores are derived from ions scores as a non-probabilistic basis for ranking protein hits.

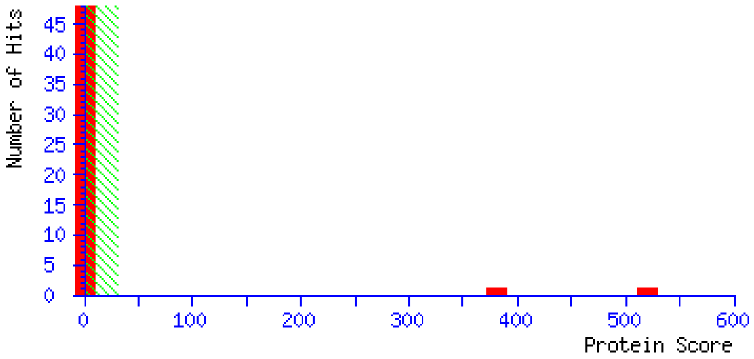

Matched peptide sequences: shown in Bold Red

```
  1 METFLYTSES VNEGHPDKLC DQVSDAILDA CLEQDPDSKV ACETCTKTNM
 51 VMVFGEITTK ANVDYEKIVR DTCRSIGFVS DDVGLDADKC KVLVYIEQQS
101 PDIAQGVHGH LTKRPEEIGA GDQGHMFGYA TDETPELMPL SHVLATKLGA
151 RLTEVRKNGT CPWLRPDGKT QVTVEYYNDN GAMVPVRVHT VLISTQHDET
201 VTNDEIAADL KEHVIKPVIP EKYLDEKTIF HLNPSGRFVI GGPHGDAGLT
251 GRKIIIDTYG GWGAHGGGAF SGKDPTKVDR SGAYIVRQAA KSIVASGLAR
301 RCIVQVSYAI GVPEPLSVFV DTYGTGKIPD KEILKIVKEN FDFRPGMISI
351 NLDLKRGGNG RFLKTAAYGH FGRDDADFTW EVVKPLKWEK PQA
```

Matched peptide information:

Start - End	Observed	Mr(expt)	Mr(calc)	ppm	Miss	Sequence
92 - 113	2432.2786	2431.2713	2431.2860	-6	0	K.VLVYIEQQSPDIAQGVHGHLTK.R (Ions score 84)
158 - 169	1400.6670	1399.6597	1399.6667	-5	1	K.NGTCPWLRPDGK.T (Ions score 50)
170 - 187	2055.9756	2054.9683	2054.9731	-2	0	K.TQVTVEYYNDNGAMVPVR.V (Ions score 102)
188 - 211	2649.3247	2648.3174	2648.3293	-4	0	R.VHTVLISTQHDETVTNDEIAADLK.E (Ions score 98)
228 - 237	1141.6016	1140.5943	1140.6040	-9	0	K.TIFHLNPSGR.F (Ions score 68)
238 - 252	1453.7452	1452.7380	1452.7474	-6	0	R.FVIGGPHGDAGLTGR.K (Ions score 121)
254 - 273	1963.9570	1962.9498	1962.9588	-5	0	K.IIIDTYGGWGAHGGGAFSGK.D (Ions score 117)
339 - 355	2009.0089	2008.0016	2008.0088	-4	1	K.ENFDFRPGMISINLDLK.R (Ions score 50)
365 - 373	979.4598	978.4525	978.4672	-15	0	K.TAAYGHFGR.D (Ions score 63)

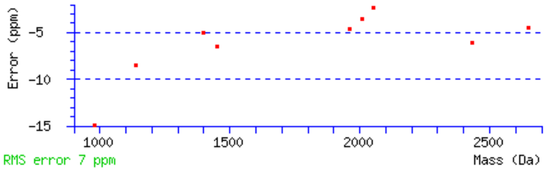

Spot No.:**129**

Accession No.: **scaffold1735_70085.mRNA1**

Protein name：**ricin B-like lectin EULS3**

Peptide sequences:

K.ADPNFFLTIR.D;K.VVLAPPDPSDEFQHWYK.D;K.VVLAPPDPSDEFQHWYKDEK.F;K.FSTMVKDEEGFPSFALVNK.A;K.DEEGFPSFALVNK.A;K.HSFGPTHPVLLTPYDPDDLEASILWTESK.D;K.VVLAYWNK.G

PFF Mascot score: **[440]** Sequence coverage %: **[46]**

Matched peptides No.: **[7]**

Calculated Mr: **20724** Calculated *p*I: **7.77**

Data base searched result:

Ions score is -10*Log(P), where P is the probability that the observed match is a random event.
Individual ions scores > 30 indicate identity or extensive homology (p<0.05).
Protein scores are derived from ions scores as a non-probabilistic basis for ranking protein hits.

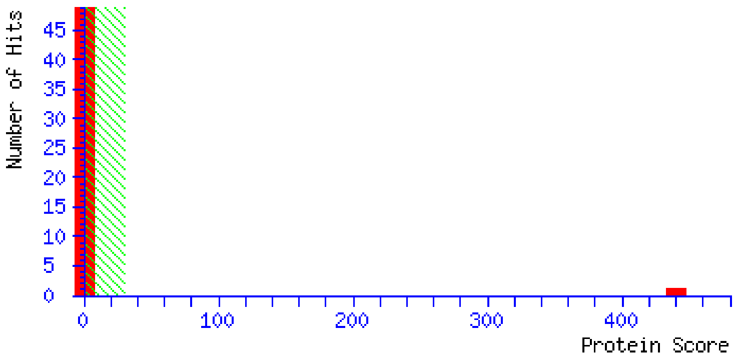

Matched peptide sequences: shown in Bold Red

```
  1 MEELGSSHEK TESESSHCRK PHLPSGSYLS KKPSYKVYCK ADPNFFLTIR
 51 DGKVVLAPPD PSDEFQHWYK DEKFSTMVKD EEGFPSFALV NKASGKALKH
101 SFGPTHPVLL TPYDPDDLEA SILWTESKDL GDGYRIVRML NNIRLNVEAL
151 RGDKKSGGVS NGTKVVLAYW NKGDHQKWRI APL
```

Matched peptide information:

Start - End	Observed	Mr(expt)	Mr(calc)	ppm	Miss	Sequence
41 - 50	1193.6271	1192.6198	1192.6241	-4	0	K.ADPNFFLTIR.D (Ions score 85)
54 - 70	2027.9949	2026.9876	2026.9789	4	0	K.VVLAPPDPSDEFQHWYK.D (Ions score 108)
54 - 73	2400.1587	2399.1514	2399.1434	3	1	K.VVLAPPDPSDEFQHWYKDEK.F (Ions score 64)
74 - 92	2146.0603	2145.0530	2145.0452	4	1	K.FSTMVKDEEGFPSFALVNK.A (Ions score 54)
80 - 92	1452.6997	1451.6924	1451.6933	-1	0	K.DEEGFPSFALVNK.A (Ions score 100)
100 - 128	3265.6077	3264.6004	3264.5979	1	0	K.HSFGPTHPVLLTPYDPDDLEASILWTESK.D (Ions score 142)
165 - 172	992.5513	991.5441	991.5491	-5	0	K.VVLAYWNK.G (Ions score 60)

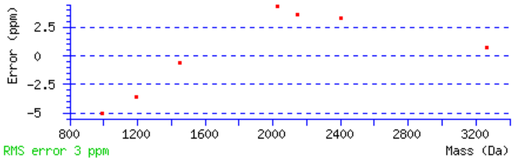

Spot No.:130

Accession No.: scaffold1194_13551.mRNA1

Protein name：(+)-neomenthol dehydrogenase

Peptide sequences:

K.GIGFEICR.Q;R.QLASNGIVVVLTAR.D;K.TDINLNMGILSVEEGAESPAR.L;K.VSTQTYESAEECLTINYYGAK.R;K.EVFVDADNLSEER.I;K.EVFVDADNLSEERIDEVLGK.Y;R.LALLPNDGPSGCFFFR.K

PFF Mascot score: [397]   Sequence coverage %: [12]

Matched peptides No.: [7]

Calculated Mr: 88348   Calculated *p*I: 7.08

Data base searched result:

Ions score is -10*Log(P), where P is the probability that the observed match is a random event.
Individual ions scores > 31 indicate identity or extensive homology (p<0.05).
Protein scores are derived from ions scores as a non-probabilistic basis for ranking protein hits.

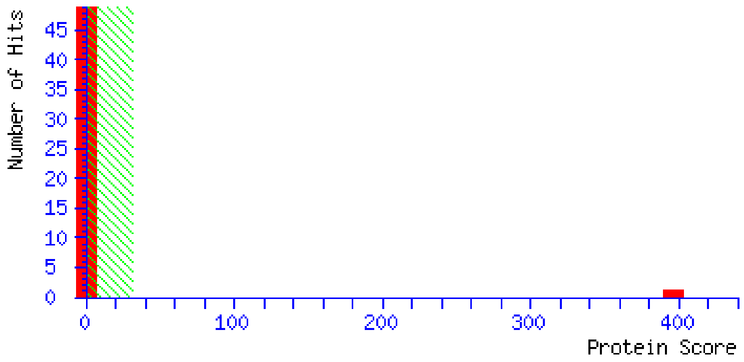

Matched peptide sequences: shown in Bold Red

1 MTEALIPLLQ LSDSPRIVNV SSSMGKLKNV TNEWAKEVFV AADNLSEERI
51 DEVLSKYLKD YKEGSLESKR WPAFMSAYIL SKAAMNAYTR ILAKKLPNFR
101 INCVCPGFVK TDINLNMGVL SVEEGAESPA RLALLPNDGP SGCFFSLTHK
151 YYLQWHKQPR GNSFLIHIHR QYSYLYLPCP LLRLLLTTSS SSFFLFRYAV
201 VTGANK**GIGF EICRQLASNG IVVVLTAR**DE KRGLEAFQKL KDSGFSDLLV
251 FHQLDVADAA SIAALVDFIK TKFGKLDIMV NNAGVLGIKV DGDAFITEPG
301 KEGASINWHK THQTYELAEE CLTINYYGAK RMVEVLIPLL QLSDSPRIVN
351 VSSSMGKLKN VTNEWAKEVF VAADNLPEER IDEVLSKYLK DYKEGSLESK
401 GWPAFMSAYI LSKAAMNAYT RILAKKLPTF RINCVCPGFV K**TDINLNMGI**
451 **LSVEEGAESP AR**LALLPNDG PSGCFFTVIL FYLLLLLLRL LVTNCSFFFF
501 RYAVVTGANK GIGFEICRQL ASNGIVVVLT ARDEKRGLEA FQKLKDSGFS
551 DLLVFHQLDV ADAASIATLA NFIKTQFGKL DILVWNNAGV GGIEVDVDAL
601 KAESSKDSGI NWHK**VSTQTY ESAEECLTIN YYGAK**RMTEA LIPLLQLSDS
651 PRIVNVSSSM GKLKNVTNEW AK**EVFVDADN LSEERIDEVL GK**YLKDYKEG
701 SLESKGWPAF MSAYILSKAA MNAYTRILAK KLPTFRINCV CPGFVKTDIN
751 LNMGILSVEE GAESPAR**LAL LPNDGPSGCF FFR**KEESPF

Matched peptide information:

Start - End	Observed	Mr(expt)	Mr(calc)	ppm	Miss	Sequence
207 - 214	951.4736	950.4664	950.4644	2	0	K.GIGFEICR.Q (Ions score 26)
215 - 228	1440.8709	1439.8636	1439.8460	12	0	R.QLASNGIVVVLTAR.D (Ions score 98)
442 - 462	2216.1206	2215.1133	2215.0790	15	0	K.TDINLNMGILSVEEGAESPAR.L (Ions score 166)
615 - 635	2427.1367	2426.1294	2426.0947	14	0	K.VSTQTYESAEECLTINYYGAK.R (Ions score 48)
673 - 685	1522.7268	1521.7195	1521.6947	16	0	K.EVFVDADNLSEER.I (Ions score 116)
673 - 692	2277.1580	2276.1507	2276.1172	15	1	K.EVFVDADNLSEERIDEVLGK.Y (Ions score 19)
768 - 783	1810.9238	1809.9166	1809.8872	16	0	R.LALLPNDGPSGCFFFR.K (Ions score 75)

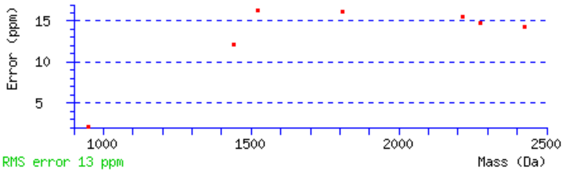

Spot No.:**131**

Accession No.: **scaffold0378_586227.mRNA1**

Protein name: **2,3-bisphosphoglycerate-independent phosphoglyceratemutase**

Peptide sequences:

R.AHGTAVGLPTEDDMGNSEVGHNALGAGR.I;K.ALEYENFDKFER.V;K.LPSHYLVSPPEIER.T;K.FGHVTFFWNGNR.S;K.SGNIQILTSHTLQPVPIAIGGPGLAPGVR.F

PFF Mascot score: **[360]** Sequence coverage %: **[16]**

Matched peptides No.: **[5]**

Calculated Mr: **44084** Calculated *p*I: **4.91**

Data base searched result:

Ions score is -10*Log(P), where P is the probability that the observed match is a random event.
Individual ions scores > 30 indicate identity or extensive homology (p<0.05).
Protein scores are derived from ions scores as a non-probabilistic basis for ranking protein hits.

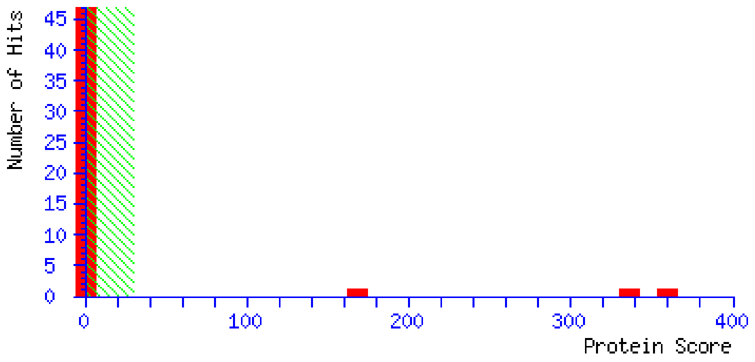

Matched peptide sequences: shown in Bold Red

```
  1 MGSSGEFTWK LADHPKLPKG KIIAMVVLDG WGEAKPDKYN CIHVAETPTM
 51 DSFKKTAPEK WRLIRAHGTA VGLPTEDDMG NSEVGHNALG AGRIYAQGAK
101 LVDLALASGK IFDGEGFKYI QESFASGTLH LIGLLSDGGV HSRLDQLQLL
151 LKGAAEHGAK RIRVHVLTDG RDVIDGTSVG FVETLEKDLA NLREKGVDAQ
201 IASGGGRMHV TMDRYENDWN VVKRGWDAQV LGEAPHKFRN AVEAVKKLRE
251 VPKANDQYLP PFVIVDENGK PVGPVVDGDA VVTFNFRADH RMVMLAKALE
301 YENFDKFERV RYPKIRYAGM LEYDGELKLP SHYLVSPPEI ERTSGEYLVH
351 NGVCTFACSE TVKFGHVTFF WNGNRSGYFN PEMEEYVEIP SDAGITFNVK
401 PKMKAIEIAE KARDAILGRK FHQVRVNLPN SDMVGHTGDI DATIVACKAA
451 DDAVKMIIDA IEQVGGIYVV TADHGNAEDM VKRDKSGKPA VDKSGNIQIL
501 TSHTLQPVPI AIGGPGLAPG VRFRNDVPSG GLANVAATVI NLHGFEAPCD
551 YEPTLIEVVD
```

Matched peptide information:

Start - End	Observed	Mr(expt)	Mr(calc)	ppm	Miss	Sequence
66 - 93	2733.3008	2732.2935	2732.2573	13	0	R.AHGTAVGLPTEDDMGNSEVGHNALGAGR.I (Ions score 128)
298 - 309	1560.7548	1559.7475	1559.7256	14	1	K.ALEYENFDKFER.V (Ions score 57)
329 - 342	1636.8910	1635.8837	1635.8620	13	0	K.LPSHYLVSPPEIER.T (Ions score 110)
364 - 375	1481.7269	1480.7197	1480.7000	13	0	K.FGHVTFFWNGNR.S (Ions score 64)
494 - 522	2863.6519	2862.6446	2862.6080	13	0	K.SGNIQILTSHTLQPVPIAIGGPGLAPGVR.F (Ions score 110)

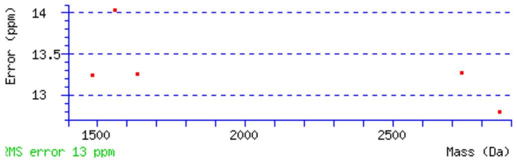

Spot No.:132

Accession No.: scaffold0143_850373.mRNA1

Protein name：Hevamine-A

Peptide sequences:

K.VMLSLGGGIGSYTLASQADAK.N;R.PLGDAVLDGIDFDIEHGSTLYWDDLAR.Y;K.KVYLTAAPQCPFPDR.Y;K.VYLTAAPQCPFPDR.Y;K.IFLGLPAAPEAAGSGYVPPDVLISR.I;K.YGGVMLWSK.F

PFF Mascot score: [450]

Sequence coverage %: [31]

Matched peptides No.: [6]

Calculated Mr: 34013

Calculated *p*I: 8.07

Data base searched result:

Ions score is -10*Log(P), where P is the probability that the observed match is a random event.
Individual ions scores > 30 indicate identity or extensive homology (p<0.05).
Protein scores are derived from ions scores as a non-probabilistic basis for ranking protein hits.

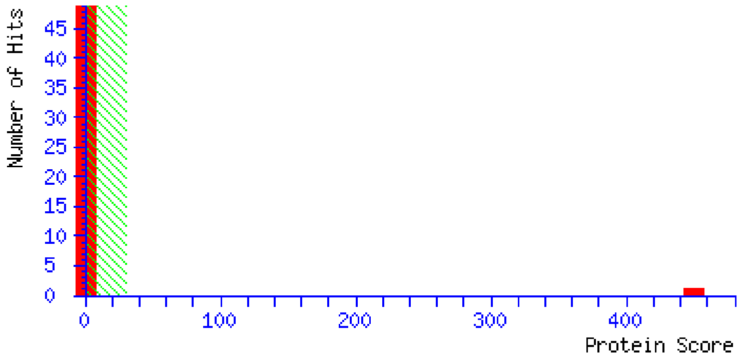

Matched peptide sequences: shown in Bold Red

```
  1 MATRTQAILL LLLAISLIMS SSHVDGGGIA IYWGQNGNEG TLTETCSTGK
 51 YSYVNIAFLN KFGNGQTPQI NLAGHCNPAA GGCTIVSNGI RSCQIQGIKV
101 MLSLGGGIGS YTLASQADAK NVADYLWNNF LGGKSSSRPL GDAVLDGIDF
151 DIEHGSTLYW DDLARYLSAY SKQGKKVYLT AAPQCPFPDR YLGTALNTGL
201 FDYVWVQFYN NPPCQYSSGN INNIINSWNR WTTSINAGKI FLGLPAAPEA
251 AGSGYVPPDV LISRILPEIK KSPKYGGVML WSKFYDDKNG YSSSIRDSVL
301 FLHSEKCMTV L
```

Matched peptide information:

Start - End	Observed	Mr(expt)	Mr(calc)	ppm	Miss	Sequence
100 - 120	2039.0862	2038.0789	2038.0405	19	0	K.VMLSLGGGIGSYTLASQADAK.N (Ions score 106)
139 - 165	3003.4998	3002.4925	3002.4298	21	0	R.PLGDAVLDGIDFDIEHGSTLYWDDLAR.Y (Ions score 151)
176 - 190	1762.9397	1761.9324	1761.8872	26	1	K.KVYLTAAPQCPFPDR.Y (Ions score 69)
177 - 190	1634.8411	1633.8338	1633.7923	25	0	K.VYLTAAPQCPFPDR.Y (Ions score 58)
240 - 264	2510.4211	2509.4139	2509.3580	22	0	K.IFLGLPAAPEAAGSGYVPPDVLISR.I (Ions score 160)
275 - 283	1040.5293	1039.5220	1039.5161	6	0	K.YGGVMLWSK.F (Ions score 50)

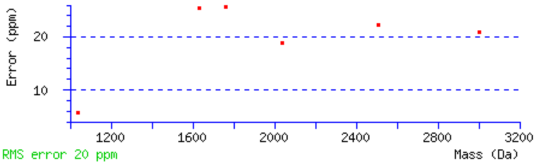

Spot No.:**133**

Accession No.: **scaffold0753_102318.mRNA1**

Protein name：**Hsp70-Hsp90 organizing protein 3**

Peptide sequences:

K.GNAAFSAGDYAGAVR.H;R.HFTDAISLAPSNHVLYSNR.S;R.LGAAHLGLNQIQDAIAAYK.K;R.VMQALGVLLNVK.F;K.FRAPNAGEDMEVPEAEAPPPQPER.K;R.APNAGEDMEVPEAEAPPPQPER.K;K.AMELDDEDISYLTNR.A;K.HDPQNQELLDGVR.R;K.LVNAGIVQIR.-

PFF Mascot score: **[559]**   Sequence coverage %: **[22]**

Matched peptides No.: **[9]**

Calculated Mr: **65314**   Calculated *p*I: **5.94**

Data base searched result:

Ions score is -10*Log(P), where P is the probability that the observed match is a random event.
Individual ions scores > 30 indicate identity or extensive homology (p<0.05).
Protein scores are derived from ions scores as a non-probabilistic basis for ranking protein hits.

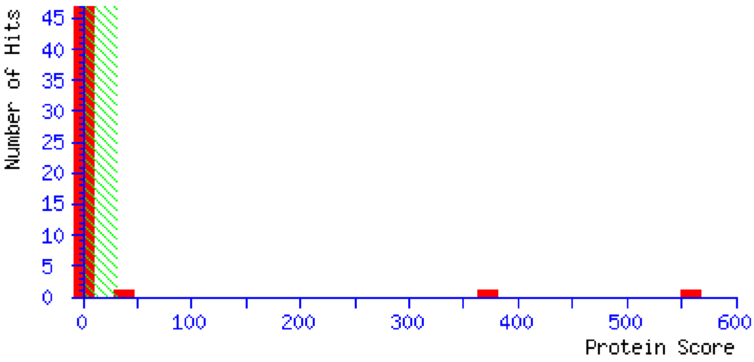

Matched peptide sequences: shown in Bold Red

1 MAEEAKAKGN AAFSAGDYAG AVRHFTDAIS LAPSNHVLYS NRSAALASLH
51 NYADALADAK KTVELKPDWS KGYSRLGAAH LGLNQIQDAI AAYKKGLEID
101 PNNEALKSGL ADAQAAASRS RSPPPPNPFG DAFSGPEMWA KLTADPTTRM
151 YLQQPDFVKM MQEIQKNPNN LNIYLKDQRV MQALGVLLNV KFRAPNAGED
201 MEVPEAEAPP PQPERKPEPT KEEKVSEPEP EPMEVSEGEK EAKERKAQAV
251 KEKELGNAAY KKKDFETAIA HYTKAMELDD EDISYLTNRA AVYLEMGKYE
301 ECIEDCDKAV ERGRELRSDF KMISRALTRK GTTLVKIAKC SKDYDLAIET
351 FQKALTEHRN PDTLKKLNDA ERAKKELEQQ EYFDSKLADE EREKGNEYFK
401 QQKYPEAVKH YTESLRRNPK DPKAYSNRAA CYTKLGALPE GLKDAEKCIE
451 LDPTFSKGYT RKGAIQFFMK EYDKALETYQ EGLKHDPQNQ ELLDGVRRCV
501 EQLNKASRGD ISPEELKERQ AKAMQDPEIQ NILSDPVMRQ VLVDFQENPK
551 AAQEHTKNPM VMNKIQKLVN AGIVQIR

Matched peptide information:

Start - End	Observed	Mr(expt)	Mr(calc)	ppm	Miss	Sequence
9 - 23	1426.6951	1425.6878	1425.6637	17	0	K.GNAAFSAGDYAGAVR.H (Ions score 84)
24 - 42	2142.1194	2141.1121	2141.0654	22	0	R.HFTDAISLAPSNHVLYSNR.S (Ions score 128)
76 - 94	1967.1074	1966.1001	1966.0636	19	0	R.LGAAHLGLNQIQDAIAAYK.K (Ions score 117)
180 - 191	1284.7798	1283.7725	1283.7635	7	0	R.VMQALGVLLNVK.F (Ions score 56)
192 - 215	2634.2842	2633.2769	2633.2180	22	1	K.FRAPNAGEDMEVPEAEAPPPQPER.K (Ions score 66)
194 - 215	2331.1133	2330.1060	2330.0484	25	0	R.APNAGEDMEVPEAEAPPPQPER.K (Ions score 83)
275 - 289	1784.8390	1783.8317	1783.7934	21	0	K.AMELDDEDISYLTNR.A (Ions score 110)
485 - 497	1520.7756	1519.7684	1519.7379	20	0	K.HDPQNQELLDGVR.R (Ions score 82)
568 - 577	1082.6830	1081.6757	1081.6608	14	0	K.LVNAGIVQIR.- (Ions score 65)

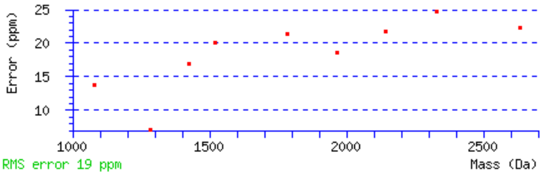

Spot No.:**134**

Accession No.: **scaffold0456_1002067.mRNA1**

Protein name：**Fructokinase-2**

Peptide sequences:

K.APGGAPANVAIAVAR.L;R.NPSADMLLKPEELNLELIR.S;K.VFHYGSISLIVEPCR.S;K.DAGALLSYDPNLR.L;R.LPLWPSPEEAR.E

PFF Mascot score: **[331]** Sequence coverage %: **[22]**

Matched peptides No.: **[5]**

Calculated Mr: **35625** Calculated *p*I: **5.22**

Data base searched result:

Ions score is -10*Log(P), where P is the probability that the observed match is a random event.
Individual ions scores > 31 indicate identity or extensive homology (p<0.05).
Protein scores are derived from ions scores as a non-probabilistic basis for ranking protein hits.

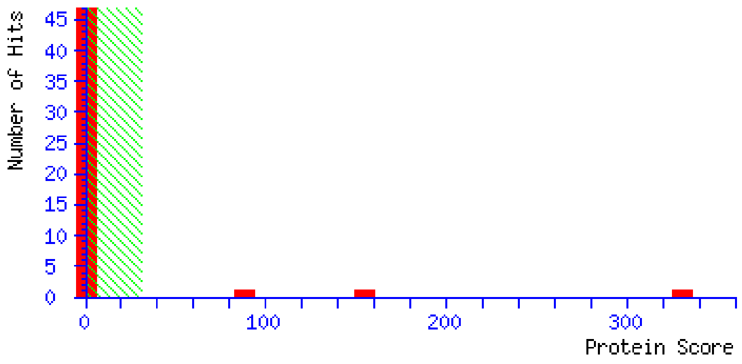

Matched peptide sequences: shown in Bold Red

```
  1 MASVVSNGGS GSSLIVSFGE MLIDFVPTVS GVSLAEAPGF LKAPGGAPAN
 51 VAIAVARLGG KAAFVGKLGD DEFGHMLAGI LKQNGVIGDG INFDKGARTA
101 LAFVTLRADG EREFMFYRNP SADMLLKPEE LNLELIRSAK VFHYGSISLI
151 VEPCRSAHLK AMEVAKDAGA LLSYDPNLRL PLWPSPEEAR EQIMSIWDKA
201 EVIKVSDVEL EFLTGSDKID DADALSLWHP NLKLLLVTLG EKGCRYYTKN
251 FHGSVEAFHV KTVDTTGAGD SFVGALLCKI VDDRSILEDE PRLREVLKFA
301 NACGAITTTK KGAIPALPTE QDALTLIKGS E
```

Matched peptide information:

Start - End	Observed	Mr(expt)	Mr(calc)	ppm	Miss	Sequence
43 - 57	1334.7847	1333.7774	1333.7466	23	0	K.APGGAPANVAIAVAR.L (Ions score 89)
119 - 137	2195.2307	2194.2234	2194.1667	26	1	R.NPSADMLLKPEELNLELIR.S (Ions score 107)
141 - 155	1776.9575	1775.9502	1775.9029	27	0	K.VFHYGSISLIVEPCR.S (Ions score 122)
167 - 179	1404.7432	1403.7359	1403.7045	22	0	K.DAGALLSYDPNLR.L (Ions score 54)
180 - 190	1294.7062	1293.6989	1293.6717	21	0	R.LPLWPSPEEAR.E (Ions score 78)

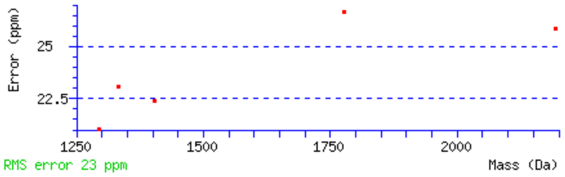

Spot No.:**135**

Accession No.: **scaffold1104_58290.mRNA1**

Protein name： **V-type proton ATPase subunit E**

Peptide sequences:

K.QIQQMVR.F;K.ANEISVSAEEEFNIEK.L;K.IRQEYER.K;K.KIEYSMQLNASR.I;K.IEYSMQLNASR.I;K.EAAGNDLLNVSHDHHVYK.K;K.DLIVQSLLR.L;K.IVCENTLDAR.L

PFF Mascot score: **[359]** Sequence coverage %: **[34]**

Matched peptides No.: **[8]**

Calculated Mr: **26506** Calculated *p*I: **7.13**

Data base searched result:

Ions score is -10*Log(P), where P is the probability that the observed match is a random event.
Individual ions scores > 31 indicate identity or extensive homology (p<0.05).
Protein scores are derived from ions scores as a non-probabilistic basis for ranking protein hits.

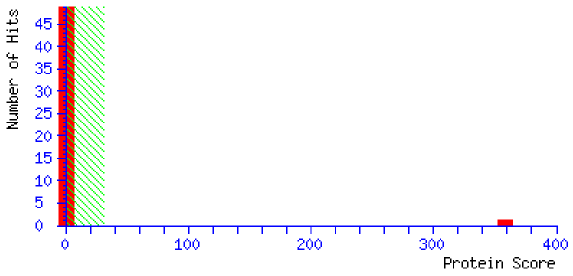

Matched peptide sequences: shown in Bold Red

```
  1 MNDADVSKQI QQMVRFIRQE AEEKANEISV SAEEEFNIEK LQLVEAEKKK
 51 IRQEYERKEK QVDVRKKIEY SMQLNASRIK VLQAQDDVVN AMKEAAGNDL
101 LNVSHDHHVY KKLLKDLIVQ SLLRLKEPSV LLRCRKDDLH LVQSVLDSAK
151 QEYAEKVNVH APEIIVDNHV YLPPAPSHHD AHGPYCSGGV VLASRDGKIV
201 CENTLDARLD VVFRKKLPEI RKRLFGQVAT
```

Matched peptide information:

Start - End	Observed	Mr(expt)	Mr(calc)	ppm	Miss	Sequence
9 - 15	902.5116	901.5043	901.4804	27	0	K.QIQQMVR.F (Ions score 36)
25 - 40	1808.8999	1807.8926	1807.8475	25	0	K.ANEISVSAEEEFNIEK.L (Ions score 133)
51 - 57	993.5243	992.5170	992.5039	13	1	K.IRQEYER.K (Ions score 43)
67 - 78	1439.7640	1438.7568	1438.7238	23	1	K.KIEYSMQLNASR.I (Ions score 43)
68 - 78	1311.6644	1310.6572	1310.6288	22	0	K.IEYSMQLNASR.I (Ions score 75)
94 - 111	2019.0238	2018.0165	2017.9606	28	0	K.EAAGNDLLNVSHDHHVYK.K (Ions score 87)
116 - 124	1056.6648	1055.6575	1055.6339	22	0	K.DLIVQSLLR.L (Ions score 65)
199 - 208	1190.6072	1189.5999	1189.5761	20	0	K.IVCENTLDAR.L (Ions score 85)

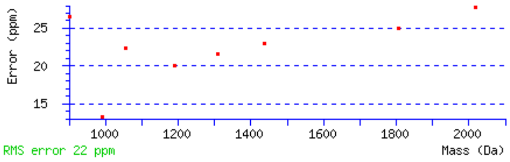

Spot No.:**136**

Accession No.: **scaffold0014_190374.mRNA1**

Protein name：**Cysteine proteinase inhibitor**

Peptide sequences:

K.EVEGSANSVEINSLAR.Y;R.YAVDDYNQK.Q;K.QNALLEFK.K;K.QQVVAGTIYYITLEVTDGGQK.K;K.VWEKPWLNFK.E

PFF Mascot score: **[437]** Sequence coverage %: **[63]**

Matched peptides No.: **[5]**

Calculated Mr: **11236** Calculated *p*I: **5.45**

Data base searched result:

Ions score is -10*Log(P), where P is the probability that the observed match is a random event.
Individual ions scores > 31 indicate identity or extensive homology (p<0.05).
Protein scores are derived from ions scores as a non-probabilistic basis for ranking protein hits.

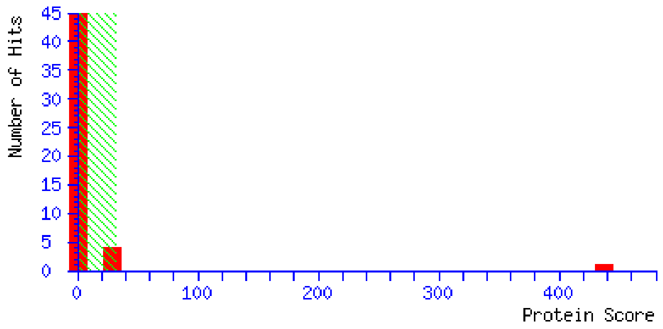

Matched peptide sequences: shown in Bold Red

```
  1 MAKLGGVKEV EGSANSVEIN SLARYAVDDY NQKQNALLEF KKVVNAKQQV
 51 VAGTIYYITL EVTDGGQKKV YEAKVWEKPW LNFKEVQEFK LIGDAPSDST
101 A
```

Matched peptide information:

Start - End	Observed	Mr(expt)	Mr(calc)	ppm	Miss	Sequence
9 - 24	1674.8823	1673.8750	1673.8220	32	0	K.EVEGSANSVEINSLAR.Y (Ions score 175)
25 - 33	1115.5195	1114.5123	1114.4931	17	0	R.YAVDDYNQK.Q (Ions score 63)
34 - 41	962.5356	961.5283	961.5233	5	0	K.QNALLEFK.K (Ions score 70)
48 - 68	2283.2544	2282.2471	2282.1794	30	0	K.QQVVAGTIYYITLEVTDGGQK.K (Ions score 186)
75 - 84	1346.7539	1345.7466	1345.7183	21	1	K.VWEKPWLNFK.E (Ions score 67)

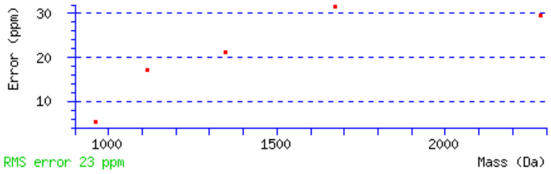

Spot No.:**137**

Accession No.: **scaffold0156_1465807.mRNA1**

Protein name：**Calmodulin-related protein**

Peptide sequences: **R.VFDKDQNGFISAAELR.H;R.EADVDGDGQINYEEFVK.V**

PFF Mascot score: **[157]** Sequence coverage %: **[22]**

Matched peptides No.: **[2]**

Calculated Mr: **16894** Calculated *p*I: **4.11**

Data base searched result:

Ions score is -10*Log(P), where P is the probability that the observed match is a random event.
Individual ions scores > 30 indicate identity or extensive homology (p<0.05).
Protein scores are derived from ions scores as a non-probabilistic basis for ranking protein hits.

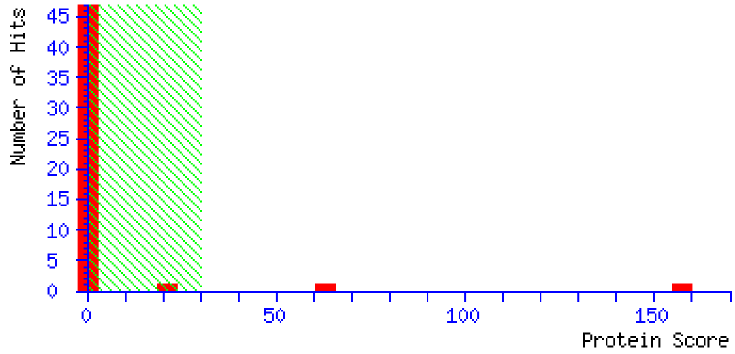

Matched peptide sequences: shown in Bold Red

```
  1 MADQLTDDQI SEFKEAFSLF DKDGDGCITT KELGTVMRSL GQNPTEAELQ
 51 DMINEVDADG NGTIDFPEFL NLMARKMKDT DSEEELKEAF RVFDKDQNGF
101 ISAAELRHVM TNLGEKLTDE EVDEMIREAD VDGDGQINYE EFVKVMMAK
```

Matched peptide information:

Start - End	Observed	Mr(expt)	Mr(calc)	ppm	Miss	Sequence	
92 - 107	1809.9544	1808.9471	1808.9057	23	1	R.VFDKDQNGFISAAELR.H	(Ions score 117)
128 - 144	1927.8925	1926.8852	1926.8483	19	0	R.EADVDGDGQINYEEFVK.V	(Ions score 70)

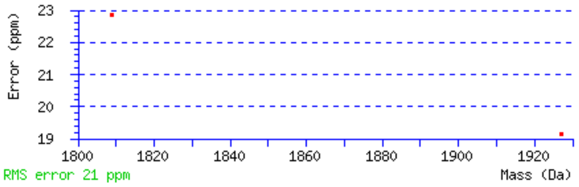

Spot No.:**138**

Accession No.: **scaffold0582_11826.mRNA1**

Protein name： **Pyrophosphate--fructose 6-phosphate 1-phosphotransferase subunit beta**

Peptide sequences:

R.FASVYSELQASR.I;K.IGVVLSGGQAPGGHNVISGIFDYLQER.A;K.GSILYGFR.G;K.YVELTADFIYPYR.N;R.DKIETPEQFK.Q;K.IYSEMIGNVMIDAR.S;R.AASHITLECALQTHPNITIIGEEVAAK.K;K.LTSQSLQLFEFLPLAIR.E;K.GQSHFFGYEGR.C;K.TGLISSVGNLGAPVAEWTVGGTALTSMMDVER.R

PFF Mascot score: **[581]** Sequence coverage %: **[30]**

Matched peptides No.: **[10]**

Calculated Mr: **62382** Calculated *p*I: **7.18**

Data base searched result:

Ions score is -10*Log(P), where P is the probability that the observed match is a random event.
Individual ions scores > 31 indicate identity or extensive homology (p<0.05).
Protein scores are derived from ions scores as a non-probabilistic basis for ranking protein hits.

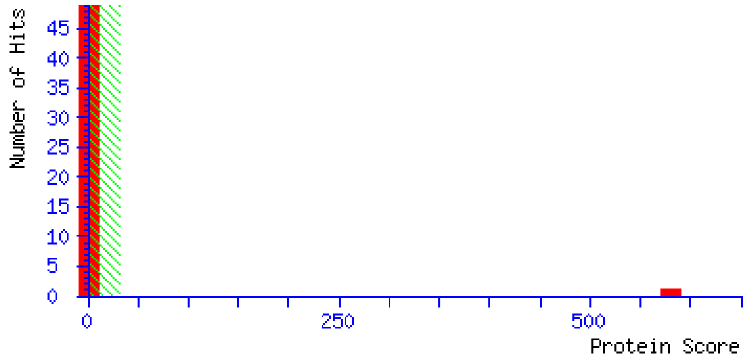

Matched peptide sequences: shown in Bold Red

```
  1 MAPSFVINGE VPTVKSSPAT GRFASVYSEL QASRIDHSLP VPSVLKNPFK
 51 VVEGPPSSAA GNPDEIAKLF PNLFGQPSSM FVPNGADAVH SNLKLKIGVV
101 LSGGQAPGGH NVISGIFDYL QERAKGSILY GFRGGPAGIM KCKYVELTAD
151 FIYPYRNQGG FDMICSGRDK IETPEQFKQA EETALKLDLD GLVVIGGDDS
201 NTNACLLAEN FRTKNMKTRV IGCPKTIDGD LKCKEVPTSF GFDTACKIYS
251 EMIGNVMIDA RSTGKYYHFV RLMGRAASHI TLECALQTHP NITIIGEEVA
301 AKKLTLKNVT DYIVDIICKR ADLGYNYGVI LIPEGLIDFI PEVQHLIAEL
351 NEILARDVVD EGGLWKKKLT SQSLQLFEFL PLAIREQLML ERDPHGNVQV
401 AKIETEKMLI QMVETELEKR KQQGSYKAQF KGQSHFFGYE GRCGLPTNFD
451 ATYCYALGYG AGALLHSGKT GLISSVGNLG APVAEWTVGG TALTSMMDVE
501 RRHGKFKPVI KKAMVELEGA PFKKFASMRN EWALKNRYIS PGPIQFMGPG
551 SDAVNHTLLL ELGVHA
```

Matched peptide information:

Start - End	Observed	Mr(expt)	Mr(calc)	ppm	Miss	Sequence
23 - 34	1357.6910	1356.6838	1356.6674	12	0	R.FASVYSELQASR.I (Ions score 64)
97 - 123	2783.4915	2782.4842	2782.4402	16	0	K.IGVVLSGGQAPGGHNVISGIFDYLQER.A (Ions score 132)
126 - 133	912.5013	911.4940	911.4865	8	0	K.GSILYGFR.G (Ions score 20)
144 - 156	1649.8518	1648.8445	1648.8137	19	0	K.YVELTADFIYPYR.N (Ions score 78)
169 - 178	1234.6469	1233.6396	1233.6241	13	1	R.DKIETPEQFK.Q (Ions score 66)
248 - 261	1611.8114	1610.8041	1610.7796	15	0	K.IYSEMIGNVMIDAR.S (Ions score 89)
276 - 302	2887.5410	2886.5337	2886.4909	15	0	R.AASHITLECALQTHPNITIIGEEVAAK.K (Ions score 18)
369 - 385	1976.1608	1975.1535	1975.1142	20	0	K.LTSQSLQLFEFLPLAIR.E (Ions score 135)
432 - 442	1284.5919	1283.5846	1283.5683	13	0	K.GQSHFFGYEGR.C (Ions score 70)
470 - 501	3219.6440	3218.6368	3218.5952	13	0	K.TGLISSVGNLGAPVAEWTVGGTALTSMMDVER.R (Ions score 153)

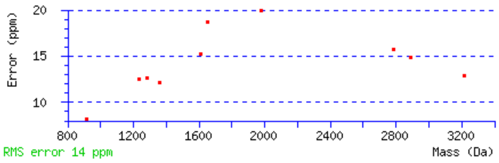

Spot No.:**139**

Accession No.: **scaffold0280_1344905.mRNA1**

Protein name： **Dynamin-related protein 4C**

Peptide sequences:

K.SSVLESLAGINLPR.G;K.GIPDLTMIDLPGITR.V;R.IGDESFEDAR.K;K.ILIGGEFDEYLNDHQMHCTAR.L;R.LVEMLNLYSYELHNCSESDPTR.N;R.NFLVEEIR.V;K.LPNFLPHGAFLSILQR.K;K.LMNQQDTIFR.L;K.EIISELMSNQGDVIGR.M

PFF Mascot score: **[321]** Sequence coverage %: **[20]**

Matched peptides No.: **[9]**

Calculated Mr: **74278** Calculated *p*I: **5.73**

Data base searched result:

Ions score is -10*Log(P), where P is the probability that the observed match is a random event.
Individual ions scores > 31 indicate identity or extensive homology (p<0.05).
Protein scores are derived from ions scores as a non-probabilistic basis for ranking protein hits.

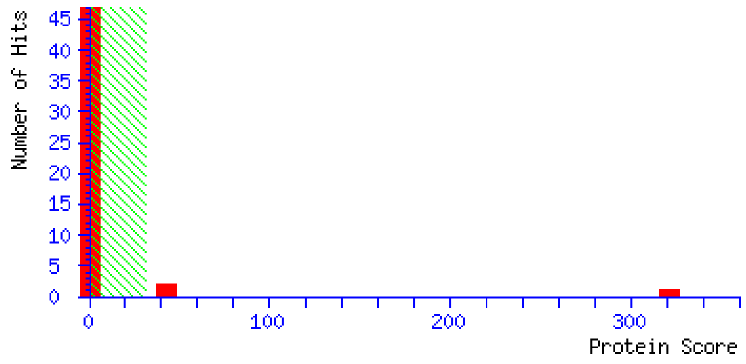

Matched peptide sequences: shown in Bold Red

1	MAHFSNGPDL	VEYEDANMET	HVPLVSSYND	HIRPLLDAVD	KLRHLKIMNE
51	GIKLPTIVVV	GDQSSGKSSV	LESLAGINLP	RGQGICTRVP	LVMRLQHQPT
101	PTPEVFLEFN	GKTVYTDEAR	AADAISLATD	EIAGDGKGIS	NTPLTLVVKK
151	KGIPDLTMID	LPGITRVPVH	GQPEDIYEQI	AGIVTEYIRP	EESIILNVLS
201	ATVDFPTCES	IRMSRQVDKT	GERTLAVVTK	SDKEPEGLLE	KVAADDLNIG
251	LGYVCVKNRI	GDESFEDARK	EEARLFKNHP	LLSRIDKSMV	GIPFLAQKLT
301	QIQATIIAKC	LPDIARNINE	KLNASISELN	KMPRTPSSLA	EAMTAFIGIV
351	GSAKESLRKI	LIGGEFDEYL	NDHQMHCTAR	LVEMLNLYSY	ELHNCSESDP
401	TRNFLVEEIR	VLEESRGIKL	PNFLPHGAFL	SILQRKVDGI	SRMPIDFVEK
451	IWAYIESVVL	SVLTHHSENY	HQLQLSTSGA	AKNLISKMKE	QSISWVTEII
501	QMEKMTDYTC	SPDYMSEWSK	LMNQQDTIFR	LILTQGYSKV	KIEGIGEVEA
551	GHIKAHQNVL	HQAFDLKMRM	TAYWMIVLRR	LVESMALHLQ	FSVQNLVNKE
601	MEKEIISELM	SNQGDVIGRM	MEESPSIAAK	REKLNKSIKL	LGESKKVLGN
651	IMDKIATYSD				

Matched peptide information:

Start - End	Observed	Mr(expt)	Mr(calc)	ppm	Miss	Sequence
68 - 81	1455.8456	1454.8383	1454.8093	20	0	K.SSVLESLAGINLPR.G (Ions score 89)
152 - 166	1611.9130	1610.9057	1610.8702	22	0	K.GIPDLTMIDLPGITR.V (Ions score 81)
260 - 269	1138.5250	1137.5177	1137.4938	21	0	R.IGDESFEDAR.K (Ions score 37)
360 - 380	2519.1965	2518.1893	2518.1369	21	0	K.ILIGGEFDEYLNDHQMHCTAR.L (Ions score 50)
381 - 402	2670.2712	2669.2640	2669.2101	20	0	R.LVEMLNLYSYELHNCSESDPTR.N (Ions score 53)
403 - 410	1019.5681	1018.5608	1018.5447	16	0	R.NFLVEEIR.V (Ions score 63)
420 - 435	1823.0776	1822.0704	1822.0254	25	0	K.LPNFLPHGAFLSILQR.K (Ions score 43)
521 - 530	1265.6520	1264.6447	1264.6234	17	0	K.LMNQQDTIFR.L (Ions score 41)
604 - 619	1760.9257	1759.9184	1759.8774	23	0	K.EIISELMSNQGDVIGR.M (Ions score 89)

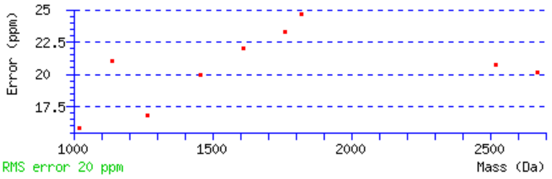

Spot No.:**140**

Accession No.: **scaffold0402_442046.mRNA1**

Protein name：**2,3-bisphosphoglycerate-independent phosphoglyceratemutase**

Peptide sequences:

K.YIQESFATGTLHLIGLLSDGGVHSR.L;K.ALEYENFDKFDR.V;K.LPSHYLVSPPEIER.T;K.FGHVTFFWNGNR.S;K.IQILTSHTLQPVPIAIGGPGLAPGVR.F

PFF Mascot score: **[300]**   Sequence coverage %: **[15]**

Matched peptides No.: **[5]**

Calculated Mr: **61547**   Calculated *p*I: **5.80**

Data base searched result:

Ions score is -10*Log(P), where P is the probability that the observed match is a random event.
Individual ions scores > 30 indicate identity or extensive homology (p<0.05).
Protein scores are derived from ions scores as a non-probabilistic basis for ranking protein hits.

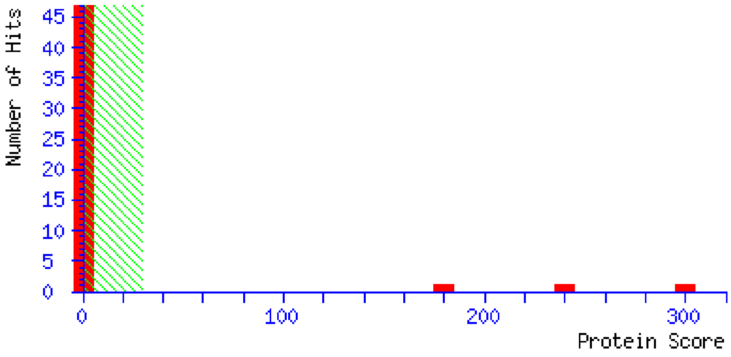

Matched peptide sequences: shown in Bold Red

```
  1 MRSSGEFTWK LADHPKLPKG KIIAMVVLDG WGEAKPDQYN CIHVADTPTM
 51 DSFKKTAPEK WRLIRAHGTA VGLPTEDDMG NSEVGHNALG AGRIYAQGAK
101 LVDHALASGK IYDGEGFKYI QESFATGTLH LIGLLSDGGV HSRLDQLQLL
151 LKGAAEHGAK RIRVHVLTDG RDVIDGTSVG FVETLEKDLA NLREKGFDAQ
201 IASGGGRMYV TMDRYENDWN VVKRGWDAQV LGEAPHKFRN AVEAVKKLRE
251 DPKANDQYLT SFVIVDENGK PVGPIVDGDA VVTFNFRADR MVMLAKALEY
301 ENFDKFDRVR FPKIHYAGML QYDGELKLPS HYLVSPPEIE RTSGEYLVHN
351 GVRTFACSET VKFGHVTFFW NGNRSGYFNA EMEEYVEIPS DVGITFNIQP
401 KMKAIEIAEK ARDAILSRKF NQVRVNLPNG DMVGHTGDID ATIVACKAAD
451 DAVKMIIDAV EQVGGIYVVT ADHGNAEDMV KRDKSGKPVV DKSGKIQILT
501 SHTLQPVPIA IGGPGLAPGV RFRNDVPSGG LANVAATVMN LHGFEAPNDY
551 EPTLIEVVDN
```

Matched peptide information:

Start - End	Observed	Mr(expt)	Mr(calc)	ppm	Miss	Sequence
119 - 143	2671.4351	2670.4278	2670.3766	19	0	K.YIQESFATGTLHLIGLLSDGGVHSR.L (Ions score 70)
297 - 308	1546.7465	1545.7392	1545.7099	19	1	K.ALEYENFDKFDR.V (Ions score 83)
328 - 341	1636.8998	1635.8925	1635.8620	19	0	K.LPSHYLVSPPEIER.T (Ions score 102)
363 - 374	1481.7333	1480.7260	1480.7000	18	0	K.FGHVTFFWNGNR.S (Ions score 66)
496 - 521	2605.5696	2604.5623	2604.5116	19	0	K.IQILTSHTLQPVPIAIGGPGLAPGVR.F (Ions score 91)

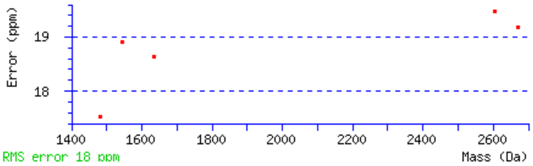

RMS error 18 ppm

Spot No.:**141**

Accession No.: **scaffold0014_3373206.mRNA1**

Protein name：**UDP-glucose 6-dehydrogenase 1**

Peptide sequences:

K.CPSIEVAVVDISVSR.I;K.FQILSNPEFLAEGTAISDLLSPDR.V;R.ETPEGQAAIEALKDVYAQWVPEDR.I;K.LAANAFLAQR.I;R.LSIYDPQVTDDQIQR.D;K.KFDWDHPLHLQPTSPTTVK.Q

PFF Mascot score: **[360]** Sequence coverage %: **[22]**

Matched peptides No.: **[6]**

Calculated Mr: **53491** Calculated *p*I: **5.90**

Data base searched result:

Ions score is -10*Log(P), where P is the probability that the observed match is a random event.
Individual ions scores > 31 indicate identity or extensive homology (p<0.05).
Protein scores are derived from ions scores as a non-probabilistic basis for ranking protein hits.

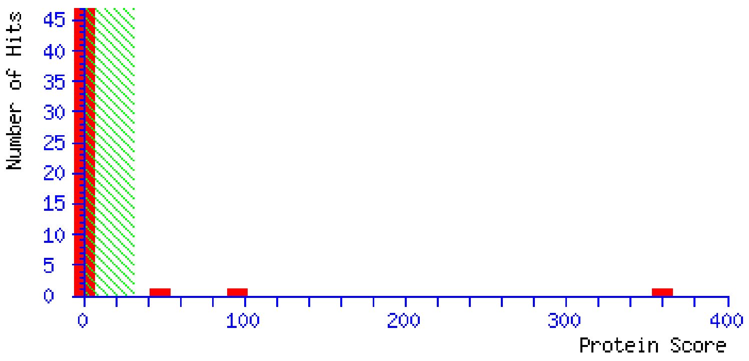

Matched peptide sequences: shown in Bold Red

```
  1 MVKICCIGAG YVGGPTMAVI ALKCPSIEVA VVDISVSRIN AWNSEQLPIY
 51 EPGLDGVVKE CRGRNLFFST EVEKHVSEAD IVFVSVNTPT KTRGLGAGKA
101 ADLTYWESAA RMIADVSKSN KIVVEKSTVP VKTAEAIEKI LTHNSKGIKF
151 QILSNPEFLA EGTAISDLLS PDRVLIGGRE TPEGQAAIEA LKDVYAQWVP
201 EDRILTTNLW SAELSKLAAN AFLAQRISSV NAMSALCEAT GADVTQVSYA
251 VGKDTRIGPK FLNASVGFGG SCFQKDILNL VYICECNGLP EVAEYWKQVI
301 KINDYQKNRF VNRVVSSMFN TVSNKKIAIL GFAFKKDTGD TRETPAIDVC
351 KGLLGDKARL SIYDPQVTDD QIQRDLTMKK FDWDHPLHLQ PTSPTTVKQV
401 TVVWDAYEAT KDAHGLCFLT EWDEFKTLDY KRIYDNMQKP AFVFDGRNVV
451 NVDKLREIGF IVYSIGKPLD AWLKDMPAVA
```

Matched peptide information:

Start - End	Observed	Mr(expt)	Mr(calc)	ppm	Miss	Sequence
24 - 38	1630.8677	1629.8604	1629.8396	13	0	K.CPSIEVAVVDISVSR.I (Ions score 116)
150 - 173	2633.3855	2632.3782	2632.3384	15	0	K.FQILSNPEFLAEGTAISDLLSPDR.V (Ions score 138)
180 - 203	2715.3701	2714.3628	2714.3187	16	1	R.ETPEGQAAIEALKDVYAQWVPEDR.I (Ions score 69)
217 - 226	1074.6060	1073.5987	1073.5981	1	0	K.LAANAFLAQR.I (Ions score 61)
360 - 374	1790.9198	1789.9125	1789.8846	16	0	R.LSIYDPQVTDDQIQR.D (Ions score 91)
380 - 398	2247.1870	2246.1797	2246.1484	14	1	K.KFDWDHPLHLQPTSPTTVK.Q (Ions score 24)

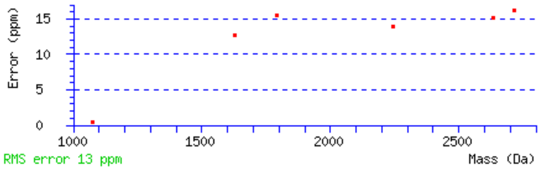

Spot No.:**142**

Accession No.: **scaffold0233_1484662.mRNA1**

Protein name：**26S proteasome non-ATPase regulatory subunit 4 homolog**

Peptide sequences:

M.VLEATMICIDNSEWMR.N;R.FQAQADAVNLICGAK.T;R.IIVFAGSPIK.H;K.SNVALDIVDFGEEEDGKAEK.L;K.GGEQPSSSQDATMTESASVAASEADNKR.N;R.NDLTDEENALLQQALAMSMDEPASSHELR.D;K.LLADQSFVSSILASLPGVDPNDPSVK.D

PFF Mascot score: **[494]** Sequence coverage %: **[35]**

Matched peptides No.: **[7]**

Calculated Mr: **42727** Calculated *p*I: **4.57**

Data base searched result:

Ions score is -10*Log(P), where P is the probability that the observed match is a random event.
Individual ions scores > 30 indicate identity or extensive homology (p<0.05).
Protein scores are derived from ions scores as a non-probabilistic basis for ranking protein hits.

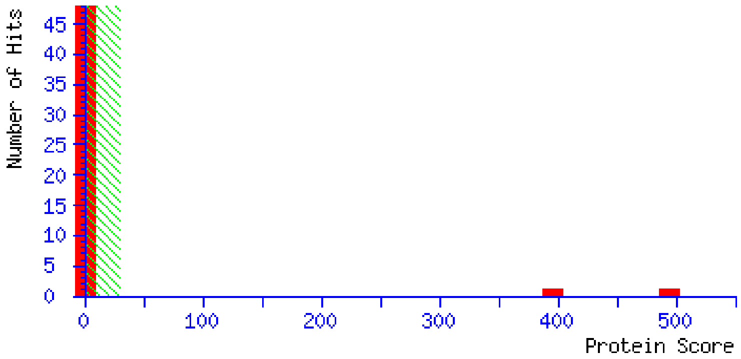

Matched peptide sequences: shown in Bold Red

```
  1 MVLEATMICI DNSEWMRNGD YSPSRFQAQA DAVNLICGAK TQSNPENTVG
 51 VLTMAGKGVR VLVTPTSDLG KILACMHGLE IGGEMNLAAG IQVAQLALKH
101 RQNKKQQQRI IVFAGSPIKH DKKTLEMIGR KLKKSNVALD IVDFGEEEDG
151 KAEKLEALLA AVNTNDTSHI VHVPPGPNAL SDVLISTPIF TGDGEGGSGF
201 AAAAAAAAAG GVSGFEFGVD PNLDPELALA LRVSMEEERA RQEAAAKKAA
251 EEGSKQEKGG EQPSSSQDAT MTESASVAAS EADNKRNDLT DEENALLQQA
301 LAMSMDEPAS SHELRDTDMS EAAADDPDLA LALQLSVQDS TKDSGSQTDM
351 SKLLADQSFV SSILASLPGV DPNDPSVKDL LASMQSQSEP PEKKDEDKPK
401 EEK
```

Matched peptide information:

Start - End	Observed	Mr(expt)	Mr(calc)	ppm	Miss	Sequence
2 - 17	1967.9331	1966.9258	1966.8951	16	0	M.VLEATMICIDNSEWMR.N (Ions score 123)
26 - 40	1605.8146	1604.8073	1604.7981	6	0	R.FQAQADAVNLICGAK.T (Ions score 109)
110 - 119	1044.6395	1043.6323	1043.6379	-5	0	R.IIVFAGSPIK.H (Ions score 74)
135 - 154	2165.0459	2164.0386	2164.0172	10	1	K.SNVALDIVDFGEEEDGKAEK.L (Ions score 94)
259 - 286	2811.2859	2810.2786	2810.2260	19	1	K.GGEQPSSSQDATMTESASVAASEADNKR.N (Ions score 94)
287 - 315	3228.5105	3227.5032	3227.4710	10	0	R.NDLTDEENALLQQALAMSMDEPASSHELR.D (Ions score 84)
353 - 378	2669.4382	2668.4310	2668.3960	13	0	K.LLADQSFVSSILASLPGVDPNDPSVK.D (Ions score 79)

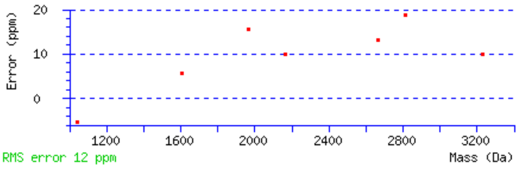

Spot No.:143

Accession No.: scaffold0347_688965.mRNA1

Protein name： Annexin D4

Peptide sequences:

R.STHFFIEDER.S;R.FENALVLWAMHPWER.D;R.VILEIACTR.S;K.AYHSLYDHSIEEDVAIHVTGSER.K;K.LLVALVSAYR.Y;K.EIKEEYNSLYGVPLTQK.I;K.DLLLALMTR.D

PFF Mascot score: [355] Sequence coverage %: [27]

Matched peptides No.: [7]

Calculated Mr: 39535 Calculated *p*I: 7.72

Data base searched result:

Ions score is -10*Log(P), where P is the probability that the observed match is a random event.
Individual ions scores > 31 indicate identity or extensive homology (p<0.05).
Protein scores are derived from ions scores as a non-probabilistic basis for ranking protein hits.

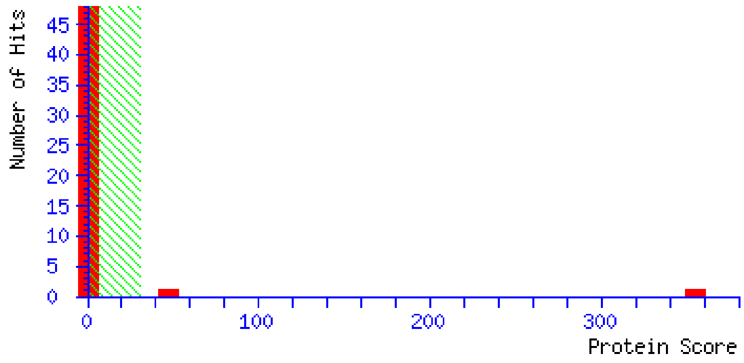

Matched peptide sequences: shown in Bold Red

```
  1 MTIITFLFYK YSSISFSLLQ NTKKKTWLIF KNWKLSPKLS QDLELNEKSL
 51 ISILGKSDPA RRTTFRQRST HFFIEDERSF ERWDDHRINL LRLEFVRFEN
101 ALVLWAMHPW ERDARLLHEA LTLVPQSYRV ILEIACTRSS EELLGARKAY
151 HSLYDHSIEE DVAIHVTGSE RKLLVALVSA YRYEGPKVSE DAAKSEAKLI
201 AKAIKNGDKK NPIDDDEVIR ILTTRSKPHL KAIYKHYKEV SGKNINEDIE
251 AADFILKETA ECLCNPHAYF SKVVDEAIRN DADHNTKKAL TRVIVTRADV
301 DLKEIKEEYN SLYGVPLTQK IDDNANGNYK DLLLALMTRD
```

Matched peptide information:

Start - End	Observed	Mr(expt)	Mr(calc)	ppm	Miss	Sequence
69 - 78	1280.5922	1279.5849	1279.5833	1	0	R.STHFFIEDER.S (Ions score 77)
98 - 112	1898.9557	1897.9484	1897.9297	10	0	R.FENALVLWAMHPWER.D (Ions score 101)
130 - 138	1074.5952	1073.5879	1073.5903	-2	0	R.VILEIACTR.S (Ions score 42)
149 - 171	2628.2495	2627.2422	2627.2252	6	0	K.AYHSLYDHSIEEDVAIHVTGSER.K (Ions score 161)
173 - 182	1104.6757	1103.6684	1103.6703	-2	0	K.LLVALVSAYR.Y (Ions score 59)
304 - 320	2011.0418	2010.0345	2010.0309	2	1	K.EIKEEYNSLYGVPLTQK.I (Ions score 45)
331 - 339	1045.6039	1044.5966	1044.6001	-3	0	K.DLLLALMTR.D (Ions score 47)

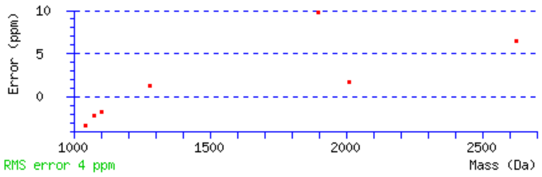

Spot No.:**144**

Accession No.: **scaffold0786_345204.mRNA1**

Protein name：**UTP--glucose-1-phosphate uridylyltransferase**

Peptide sequences:

K.NGFISLVSR.Y;R.YLSGEAQQVEWSK.I;R.LVVEDFTPLPSK.G;K.DGWYPPGHGDVFPSLK.N;K.VQLLEIAQVPDEHVSEFK.S;K.VLQLETAAGAAIR.F;K.ATSDLLLVQSDLYTLDDGYVVR.N;K.VTGDVWFGAGVILK.G

PFF Mascot score: **[449]** Sequence coverage %: **[24]**

Matched peptides No.: **[8]**

Calculated Mr: **51656** Calculated *p*I: **6.08**

Data base searched result:

Ions score is -10*Log(P), where P is the probability that the observed match is a random event.
Individual ions scores > 31 indicate identity or extensive homology (p<0.05).
Protein scores are derived from ions scores as a non-probabilistic basis for ranking protein hits.

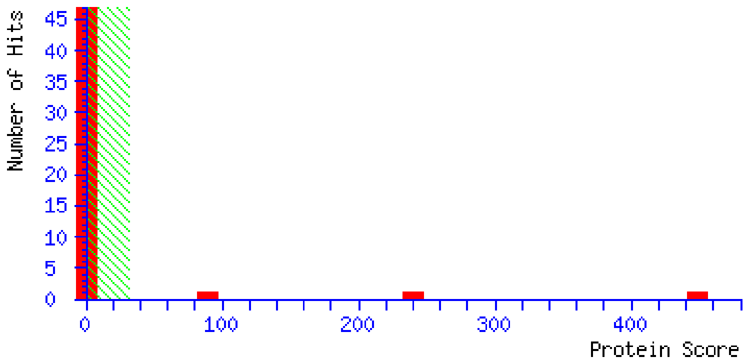

Matched peptide sequences: shown in Bold Red

1 MAVATNKLTP LKSAVAGLNQ ISDSEK**NGFI SLVSRYLSGE AQQVEWSK**IQ
51 TPTDEVVVPY DSLAPIPDDP AETKKLLDKL VVLKLNGGLG TTMGCTGPKS
101 VIEVRNGLTF LDLIVIQIEN LNSKYGCNVP LLLMNSFNTH DDTQKIIEKY
151 SKSNVEIHSF NQSQYPR**LVV EDFTPLPSK**G QSGK**DGWYPP GHGDVFPSLK**
201 NSGKLDSFLL QGKEYVFVAN SDNLGAIVDL KILNHLIRNK NEYCMEVTPK
251 TLADVKGGTL ISYEGK**VQLL EIAQVPDEHV SEFK**SIEKFK IFNTNNLWVS
301 LSAIKRLVEA DALKMEIIPN PKEVDGVK**VL QLETAAGAAI R**FFDRAIGIN
351 VPRSRFLPVK **ATSDLLLVQS DLYTLDDGYV VR**NKARQNRA NPAIELGPEF
401 KKVANFLSRF KSIPSVIELD SLK**VTGDVWF GAGVILK**GKV NIAAKPGVKL
451 EIPDGAIIEN KDINGPEDL

Matched peptide information:

Start - End	Observed	Mr(expt)	Mr(calc)	ppm	Miss	Sequence
27 - 35	992.5731	991.5658	991.5451	21	0	K.NGFISLVSR.Y (Ions score 50)
36 - 48	1524.7565	1523.7492	1523.7256	15	0	R.YLSGEAQQVEWSK.I (Ions score 60)
168 - 179	1344.7621	1343.7548	1343.7337	16	0	R.LVVEDFTPLPSK.G (Ions score 53)
185 - 200	1771.8677	1770.8604	1770.8366	13	0	K.DGWYPPGHGDVFPSLK.N (Ions score 89)
267 - 284	2081.1235	2080.1163	2080.0841	15	0	K.VQLLEIAQVPDEHVSEFK.S (Ions score 106)
329 - 341	1312.7793	1311.7720	1311.7510	16	0	K.VLQLETAAGAAIR.F (Ions score 73)
361 - 382	2456.2981	2455.2908	2455.2482	17	0	K.ATSDLLLVQSDLYTLDDGYVVR.N (Ions score 169)
424 - 437	1461.8239	1460.8166	1460.8028	9	0	K.VTGDVWFGAGVILK.G (Ions score 63)

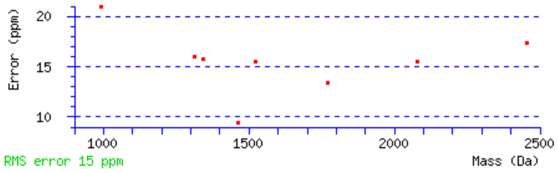

Spot No.:**145**

Accession No.: **scaffold0992_72818.mRNA1**

Protein name: **Acetyl-CoA acetyltransferase, cytosolic 1**

Peptide sequences:

M.APAAATAVAAEIKPR.D;R.ANVDPSLVQEVFFGNVLSANLGQAPAR.Q;K.DGLWDVYNDVGMGSCAEICADNHSITR.E;R.EDQDKFAIHSFER.G;K.FAIHSFER.G;R.GIAAQESGAFAWEIVPVEVSGGR.G;K.VNVHGGAVSLGHPLGCSGAR.I;R.ILVTLLGVLR.Q

PFF Mascot score: **[819]** Sequence coverage %: **[33]**

Matched peptides No.: **[8]**

Calculated Mr: **42105** Calculated *p*I: **6.54**

Data base searched result:

Ions score is -10*Log(P), where P is the probability that the observed match is a random event.
Individual ions scores > 31 indicate identity or extensive homology (p<0.05).
Protein scores are derived from ions scores as a non-probabilistic basis for ranking protein hits.

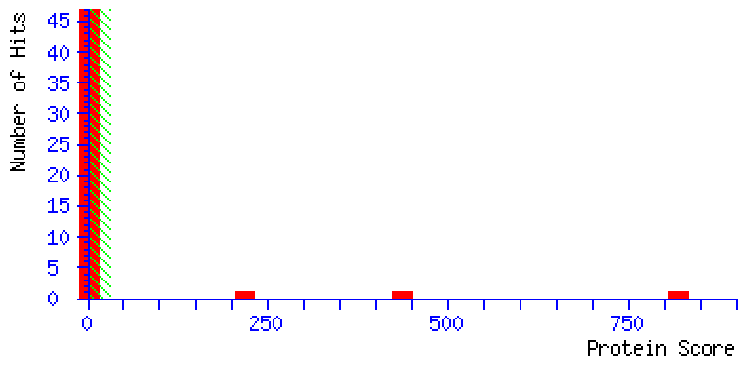

Matched peptide sequences: shown in Bold Red

```
  1 MAPAAATAVA AEIKPRDVCI VGVARTPMGG FLGSLSTLSA TKLGSIAIEA
 51 ALKRANVDPS LVQEVFFGNV LSANLGQAPA RQAALGAGIP NSVVCTTVNK
101 VCASGMKATM LAAQSIQLGI NDVVVAGGME SMSNAPKYLA EARKGSRLGH
151 DSLVDGMLKD GLWDVYNDVG MGSCAEICAD NHSITREDQD KFAIHSFERG
201 IAAQESGAFA WEIVPVEVSG GRGKPLTIVD KDEGLGKFDP VKLRKLRPSF
251 KENGGTVTAG NASSISDGAA ALILVSGETA LKLGLQVIAK IRGYADAAQA
301 PELFTTSPAL AIPKTIANGG LDASQVDYYE INEAFAVVAL ANQKLLGLNP
351 EKVNVHGGAV SLGHPLGCSG ARILVTLLGV LRQKNAKYGV GGVCNGGGGA
401 SALVVELL
```

Matched peptide information:

Start - End	Observed	Mr(expt)	Mr(calc)	ppm	Miss	Sequence
2 - 16	1436.8387	1435.8315	1435.8147	12	1	M.APAAATAVAAEIKPR.D (Ions score 98)
55 - 81	2813.4836	2812.4764	2812.4508	9	0	R.ANVDPSLVQEVFFGNVLSANLGQAPAR.Q (Ions score 223)
160 - 186	3055.3186	3054.3113	3054.2906	7	0	K.DGLWDVYNDVGMGSCAEICADNHSITR.E (Ions score 149)
187 - 199	1621.7765	1620.7692	1620.7532	10	1	R.EDQDKFAIHSFER.G (Ions score 123)
192 - 199	1006.5171	1005.5098	1005.5032	7	0	K.FAIHSFER.G (Ions score 47)
200 - 222	2330.2063	2329.1990	2329.1703	12	0	R.GIAAQESGAFAWEIVPVEVSGGR.G (Ions score 214)
353 - 372	1945.0064	1943.9991	1943.9748	12	0	K.VNVHGGAVSLGHPLGCSGAR.I (Ions score 123)
373 - 382	1096.7533	1095.7460	1095.7380	7	0	R.ILVTLLGVLR.Q (Ions score 57)

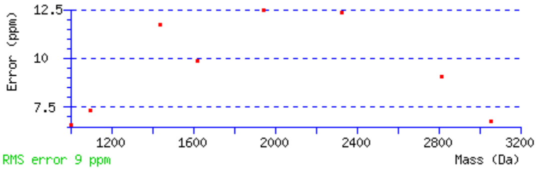

Spot No.:**146**

Accession No.: **scaffold0624_614895.mRNA1**

Protein name：**Uncharacterized protein**

Peptide sequences:

K.PTAASAVGLAGPGAAEVLPFVDADLKR.I;K.SVDDVIYEVGEGDAR.N;K.HHASILVVGSHGYGAIK.R

PFF Mascot score: **[372]** Sequence coverage %: **[41]**

Matched peptides No.: **[3]**

Calculated Mr: **15624** Calculated *p*I: **5.74**

Data base searched result:

Ions score is -10*Log(P), where P is the probability that the observed match is a random event.
Individual ions scores > 30 indicate identity or extensive homology (p<0.05).
Protein scores are derived from ions scores as a non-probabilistic basis for ranking protein hits.

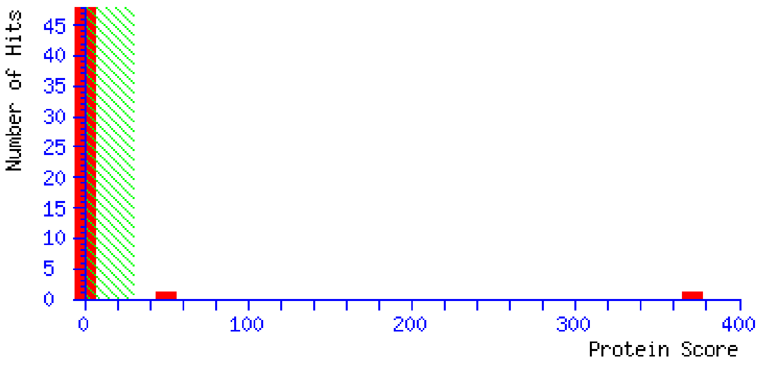

Matched peptide sequences: shown in Bold Red

```
  1 MAAAEKQVMV VGVDDSEHSL YALGWTLDHF FVPFASNSPF NLVVVHAKPT
 51 AASAVGLAGP GAAEVLPFVD ADLKRIAARV IEKTKEICMS KSVDDVIYEV
101 GEGDARNVLC ETVEKHHASI LVVGSHGYGA IKRYWHPIFC LNQ
```

Matched peptide information:

Start - End	Observed	Mr(expt)	Mr(calc)	ppm	Miss	Sequence
49 - 75	2593.4565	2592.4493	2592.3911	22	1	K.PTAASAVGLAGPGAAEVLPFVDADLKR.I (Ions score 179)
92 - 106	1623.7826	1622.7753	1622.7424	20	0	K.SVDDVIYEVGEGDAR.N (Ions score 140)
116 - 132	1745.9943	1744.9870	1744.9373	28	0	K.HHASILVVGSHGYGAIK.R (Ions score 114)

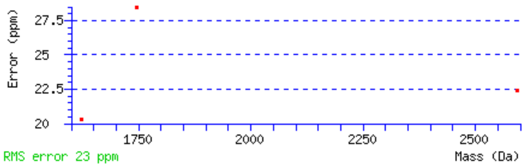

Spot No.:**147**

Accession No.: **scaffold0198_1471103.mRNA1**

Protein name：**Peroxiredoxin-2B**

Peptide sequences:

M.APIAAGDTVPEGTLAYFDDQDQLQQVSIHSLAAGK.K;K.VVIVGVPGAFTPTCSLK.H;K.HVPGFIER.A;R.RFALLVDDLK.V;R.FALLVDDLK.V;K.AANLEQGGEFTVSSVDEILK.A

PFF Mascot score: **[449]** Sequence coverage %: **[55]**

Matched peptides No.: **[6]**

Calculated Mr: **17542** Calculated *p*I: **5.55**

Data base searched result:

Ions score is -10*Log(P), where P is the probability that the observed match is a random event.
Individual ions scores > 30 indicate identity or extensive homology (p<0.05).
Protein scores are derived from ions scores as a non-probabilistic basis for ranking protein hits.

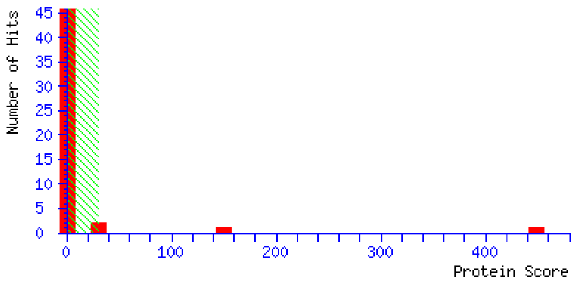

Matched peptide sequences: shown in **Bold Red**

```
  1 MAPIAAGDTV PEGTLAYFDD QDQLQQVSIH SLAAGKKVVI VGVPGAFTPT
 51 CSLKHVPGFI ERAEELKSKG VVEILCISVN DPFVMKAWAK TYPENKHVKF
101 LADGSATYTH ALGLELDLNE KGLGTRSRRF ALLVDDLKVK AANLEQGGEF
151 TVSSVDEILK AL
```

Matched peptide information:

Start - End	Observed	Mr(expt)	Mr(calc)	ppm	Miss	Sequence
2 - 36	3626.8015	3625.7942	3625.7900	1	0	M.APIAAGDTVPEGTLAYFDDQDQLQQVSIHSLAAGK.K (Ions score 144)
38 - 54	1744.9675	1743.9603	1743.9594	1	0	K.VVIVGVPGAFTPTCSLK.H (Ions score 80)
55 - 62	954.5038	953.4965	953.5083	-12	0	K.HVPGFIER.A (Ions score 66)
129 - 138	1189.6890	1188.6817	1188.6866	-4	1	R.RFALLVDDLK.V (Ions score 63)
130 - 138	1033.5828	1032.5755	1032.5855	-10	0	R.FALLVDDLK.V (Ions score 61)
141 - 160	2107.0669	2106.0596	2106.0481	5	0	K.AANLEQGGEFTVSSVDEILK.A (Ions score 182)

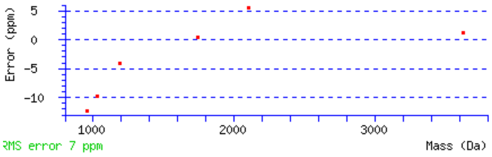

Spot No.:**148**

Accession No.: **scaffold0026_327123.mRNA1**

Protein name：**Chlorophyllase type 0**

Peptide sequences:

K.EIEFAAEVGNWLLSGLQSVLPEK.V;R.GGNIAFALALGYSK.T;K.ISALVGLDPVGR.V

PFF Mascot score: **[147]** Sequence coverage %: **[14]**

Matched peptides No.: **[3]**

Calculated Mr: **37785** Calculated *p*I: **6.30**

Data base searched result:

Ions score is -10*Log(P), where P is the probability that the observed match is a random event.
Individual ions scores > 31 indicate identity or extensive homology (p<0.05).
Protein scores are derived from ions scores as a non-probabilistic basis for ranking protein hits.

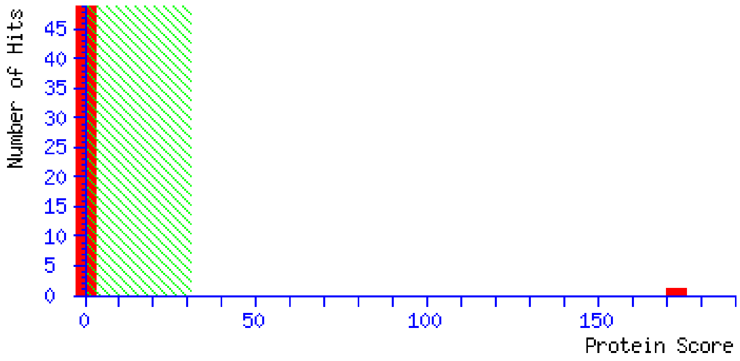

Matched peptide sequences: shown in Bold Red

1 MLVTLLVILL ASALEAKPQF PTVVLLETKP VQDILDVFVT GSFPTKSIDV

51 KKSNPASPPK PLLIVSPITD GTYPVFMFLH GTCLENYFYS NLLPHIASHG

101 FIVVAPQVYS CINWLIPKLP IRESK**EIEFA AEVGNWLLSG LQSVLPEK**VT

151 WDQDKLALGG HNR**GGNIAFA LALGYSK**TPL EVK**ISALVGL DPVGR**VSTDP

201 KILTNVPHSF NLSIPVTVIG TGLGNESVCG VVGLACAPNY MNHVKFYNKC

251 KAPASHFVTT DYGHMDMLDD NPTGILAIIA NSICKNSKDP RDQMRRTVGG

301 LIVAFLKAYF QADSGDFMTI LNEPSVAPAK LDPVQFKEEQ NHAQV

Matched peptide information:

Start - End	Observed	Mr(expt)	Mr(calc)	ppm	Miss	Sequence
126 - 148	2529.3799	2528.3726	2528.3162	22	0	K.EIEFAAEVGNWLLSGLQSVLPEK.V (Ions score 88)
164 - 177	1381.7810	1380.7737	1380.7401	24	0	R.GGNIAFALALGYSK.T (Ions score 64)
184 - 195	1196.7229	1195.7156	1195.6925	19	0	K.ISALVGLDPVGR.V (Ions score 79)

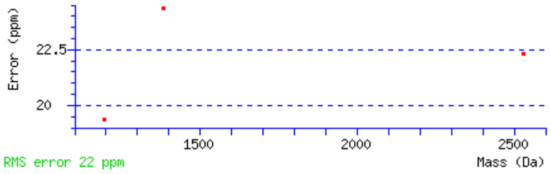

Spot No.:**149**

Accession No.: **scaffold0912_275877.mRNA1**

Protein name：**Probable rhamnose biosynthetic enzyme 1**

Peptide sequences:

K.LCESQGIDYTYGSGR.L;R.VSLEADIVNVKPSHVFNAAGVTGR.P;R.TNVVGTLTLADVCR.E;K.EEDTPNFIGSFYSK.T;K.NYENVCTLR.V;R.MPISSDLSNPR.N;R.NLTGIWNFTNPGVVSHNEILEMYR.D;R.DYVDPNFAWK.N

PFF Mascot score: **[493]** Sequence coverage %: **[39]**

Matched peptides No.: **[8]**

Calculated Mr: **34254** Calculated *p*I: **6.47**

Data base searched result:

Ions score is -10*Log(P), where P is the probability that the observed match is a random event.
Individual ions scores > 31 indicate identity or extensive homology (p<0.05).
Protein scores are derived from ions scores as a non-probabilistic basis for ranking protein hits.

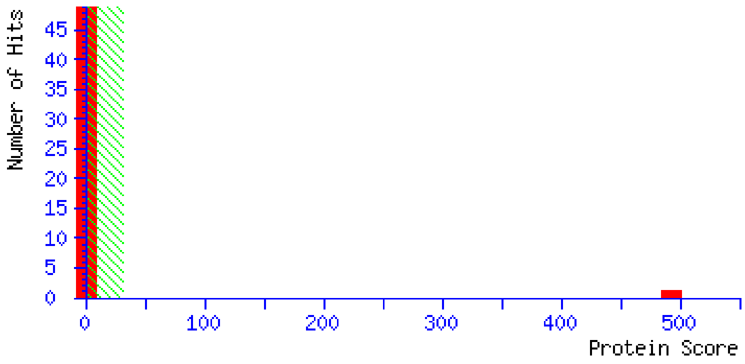

Matched peptide sequences: shown in Bold Red

```
  1 MGFPSNGTAS DTSKPLKFLI YGRTGWIGGL LGKLCESQGI DYTYGSGRLE
 51 NRVSLEADIV NVKPSHVFNA AGVTGRPNVD WCESHKVETI RTNVVGTLTL
101 ADVCREKGLI LINYATGCIF EYDTNHPLGS GIGFKEEDTP NFIGSFYSKT
151 KAMVEELLKN YENVCTLRVR MPISSDLSNP RNFITKITRY EKVVNIPNSM
201 TILDELLPIS IEMSKRNLTG IWNFTNPGVV SHNEILEMYR DYVDPNFAWK
251 NFTLEEQAKV IVAPRSNNEL DATKLSKEFP EMLPIKESLI KYVFKPNQKT
301 AAV
```

Matched peptide information:

Start - End	Observed	Mr(expt)	Mr(calc)	ppm	Miss	Sequence
34 - 48	1705.7692	1704.7619	1704.7413	12	0	K.LCESQGIDYTYGSGR.L (Ions score 130)
53 - 76	2480.3513	2479.3440	2479.3183	10	1	R.VSLEADIVNVKPSHVFNAAGVTGR.P (Ions score 113)
92 - 105	1518.8182	1517.8110	1517.7872	16	0	R.TNVVGTLTLADVCR.E (Ions score 84)
136 - 149	1633.7499	1632.7426	1632.7308	7	0	K.EEDTPNFIGSFYSK.T (Ions score 84)
160 - 168	1168.5523	1167.5450	1167.5342	9	0	K.NYENVCTLR.V (Ions score 46)
171 - 181	1216.6174	1215.6102	1215.5918	15	0	R.MPISSDLSNPR.N (Ions score 54)
217 - 240	2804.4009	2803.3936	2803.3752	7	0	R.NLTGIWNFTNPGVVSHNEILEMYR.D (Ions score 116)
241 - 250	1254.5885	1253.5812	1253.5717	8	0	R.DYVDPNFAWK.N (Ions score 70)

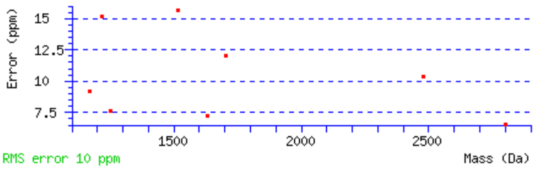

RMS error 10 ppm

Spot No.:**150**

Accession No.: **scaffold0290_149234.mRNA1**

Protein name：**26S proteasome non-ATPase regulatory subunit 14 homolog**

Peptide sequences:

R.MFAGAGGALGHPPPDSPTLDSSEQVYISSLALLK.M;R.AGVPMEVMGLMLGEFVDEYTVR.V;R.LINPQTMMLGQEPR.Q;R.QTTSNLGHLNKPSIQALIHGLNR.H;R.HYYSIAINYR.K;K.WTDGLTLR.R;K.TNEQTVQEMLNLAIK.Y

PFF Mascot score: **[348]** Sequence coverage %: **[40]**

Matched peptides No.: **[7]**

Calculated Mr: **34884** Calculated *p*I: **6.31**

Data base searched result:

Ions score is -10*Log(P), where P is the probability that the observed match is a random event.
Individual ions scores > 31 indicate identity or extensive homology (p<0.05).
Protein scores are derived from ions scores as a non-probabilistic basis for ranking protein hits.

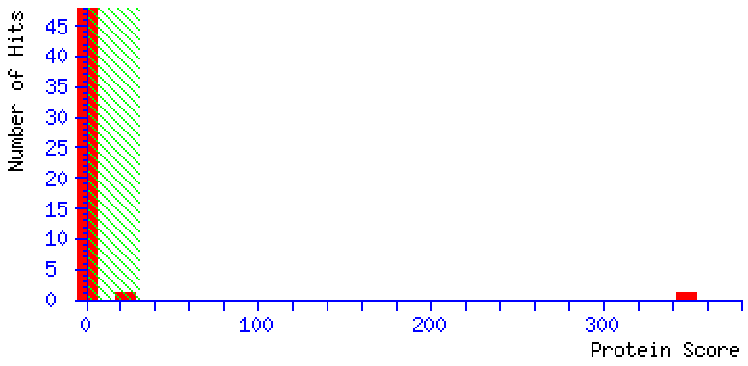

Matched peptide sequences: shown in Bold Red

```
  1 MSGMERLQRM FAGAGGALGH PPPDSPTLDS SEQVYISSLA LLKMLKHGRA
 51 GVPMEVMGLM LGEFVDEYTV RVVDVFAMPQ SGTGVSVEAV DHVFQTNMLD
101 MLKQTGRPEM VVGWYHSHPG FGCWLSGVDI NTQQSFEALN QRAVAVVVDP
151 IQSVKGKVVI DAFRLINPQT MMLGQEPRQT TSNLGHLNKP SIQALIHGLN
201 RHYYSIAINY RKNELEEKML LNLHKKKWTD GLTLRRFDTH SKTNEQTVQE
251 MLNLAIKYNK AVQEEDELPP EKLAIANVGR QDAKKHLEEH VSNLMSSNIV
301 QTLGTMLDTV VF
```

Matched peptide information:

Start - End	Observed	Mr(expt)	Mr(calc)	ppm	Miss	Sequence
10 - 43	3426.8225	3425.8152	3425.7177	28	0	R.MFAGAGGALGHPPPDSPTLDSSEQVYISSLALLK.M (Ions score 24
50 - 71	2443.2244	2442.2171	2442.1633	22	0	R.AGVPMEVMGLMLGEFVDEYTVR.V (Ions score 147)
165 - 178	1627.8840	1626.8768	1626.8222	34	0	R.LINPQTMMLGQEPR.Q (Ions score 81)
179 - 201	2512.4514	2511.4441	2511.3670	31	1	R.QTTSNLGHLNKPSIQALIHGLNR.H (Ions score 69)
202 - 211	1299.6796	1298.6723	1298.6407	24	0	R.HYYSIAINYR.K (Ions score 81)
228 - 235	961.5314	960.5242	960.5029	22	0	K.WTDGLTLR.R (Ions score 35)
243 - 257	1731.9482	1730.9410	1730.8873	31	0	K.TNEQTVQEMLNLAIK.Y (Ions score 76)

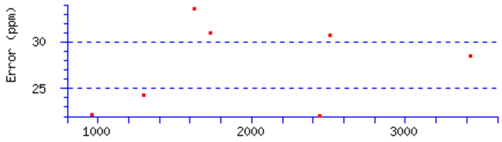

RMS error 27 ppm

Spot No.:**151**

Accession No.: **scaffold1222_136753.mRNA1**

Protein name: **Rubber elongation factor protein**

Peptide sequences: **K.NVAVPLYNR.F;K.FVDSTVVASVTIIDR.S;K.DASIQVVSAIR.A**

PFF Mascot score: **[67]** Sequence coverage %: **[25]**

Matched peptides No.: **[3]**

Calculated Mr:**14713** Calculated *p*I: **5.04**

Data base searched result:

Ions score is -10*Log(P), where P is the probability that the observed match is a random event.
Individual ions scores > 31 indicate identity or extensive homology (p<0.05).
Protein scores are derived from ions scores as a non-probabilistic basis for ranking protein hits.

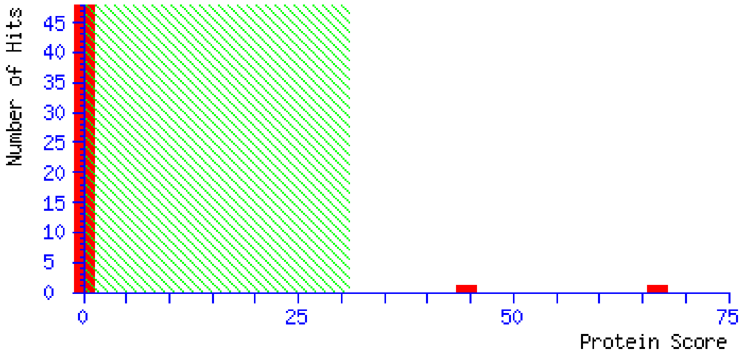

Matched peptide sequences: shown in Bold Red

```
  1 MAEDEDNQQG QGEGLKYLGF VQDAATYAVT TFSNVYLFAK DKSGPLQPGV
 51 DIIEGPVKNV AVPLYNRFSY IPNGALKFVD STVVASVTII DRSLPPIVKD
101 ASIQVVSAIR AAPEAARSLA SSLPGQTKIL AKVFYGEN
```

Matched peptide information:

Start - End	Observed	Mr(expt)	Mr(calc)	ppm	Miss	Sequence
59 - 67	1045.5967	1044.5894	1044.5716	17	0	K.NVAVPLYNR.F (Ions score 18)
78 - 92	1621.9149	1620.9076	1620.8723	22	0	K.FVDSTVVASVTIIDR.S (Ions score 67)
100 - 110	1158.6715	1157.6642	1157.6404	21	0	K.DASIQVVSAIR.A (Ions score 30)

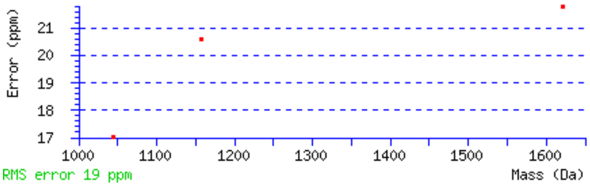

Spot No.:**152**

Accession No.: **scaffold2538_3915.mRNA1**

Protein name：**REF/SRPP-like protein At3g05500**

Peptide sequences:

K.AGPLKPGVETVEGTVK.S;K.SVVGPVYYK.F;K.AEQCAVTAWR.R;R.LNQLPLFPQVAQVVVPTAAYCSEK.Y;K.YNQTVLSTFEK.G;R.VSSYLPLVPTER.I

PFF Mascot score: **[241]** Sequence coverage %: **[33]**

Matched peptides No.: **[6]**

Calculated Mr: **27100** Calculated *p*I: **6.36**

Data base searched result:

Ions score is -10*Log(P), where P is the probability that the observed match is a random event.
Individual ions scores > 31 indicate identity or extensive homology (p<0.05).
Protein scores are derived from ions scores as a non-probabilistic basis for ranking protein hits.

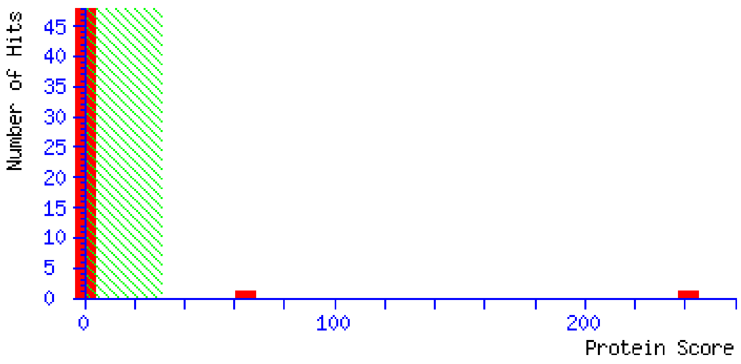

Matched peptide sequences: shown in Bold Red

1 MAEGEGNVNM QQQMENEEEE RLKYLEFVQV AVIHAVVTFT NLYLYAKEK**A**
51 **GPLKPGVETV EGTVKSVVGP VYYK**FHDVPN EVLKFVDRKV DESVTSLDSR
101 VPPVVKQVSA QAYSVAREAP VAARAVASEV HQSGVKETAS GLAKTLYTKY
151 EPKAKELYSK YEPK**AEQCAV TAWR**R**LNQLP LFPQVAQVVV PTAAYCSEKY**
201 **NQTVLSTFEK** GYR**VSSYLPL VPTER**IAKVF SDDVAQSMPL VSS

Matched peptide information:

Start - End	Observed	Mr(expt)	Mr(calc)	ppm	Miss	Sequence
50 - 65	1581.9200	1580.9128	1580.8774	22	1	K.AGPLKPGVETVEGTVK.S (Ions score 112)
66 - 74	1011.5655	1010.5582	1010.5437	14	0	K.SVVGPVYYK.F (Ions score 46)
165 - 174	1191.5790	1190.5717	1190.5502	18	0	K.AEQCAVTAWR.R (Ions score 68)
176 - 199	2672.4807	2671.4734	2671.4044	26	0	R.LNQLPLFPQVAQVVVPTAAYCSEK.Y (Ions score 38)
200 - 210	1329.6919	1328.6846	1328.6612	18	0	K.YNQTVLSTFEK.G (Ions score 48)
214 - 225	1360.7778	1359.7706	1359.7398	23	0	R.VSSYLPLVPTER.I (Ions score 70)

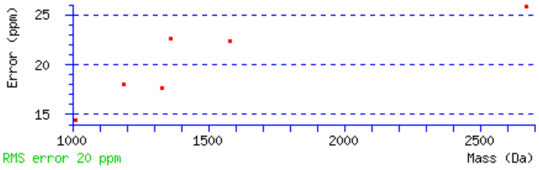

Spot No.:**153**

Accession No.: **scaffold1222_100110.mRNA1**

Protein name：**Rubber elongation factor protein**

Peptide sequences:

K.YLDFVQAATVYAR.A;K.SVVRPVYNK.F;R.RVDAYVTVLDR.I;R.VDAYVTVLDR.I;R.ASIQAYSVAPGAAR.A;R.AVASYLPLHTK.R

PFF Mascot score: **[241]** Sequence coverage %: **[33]**

Matched peptides No.: **[6]**

Calculated Mr: **19612** Calculated *p*I: **5.28**

Data base searched result:

Ions score is -10*Log(P), where P is the probability that the observed match is a random event.
Individual ions scores > 31 indicate identity or extensive homology (p<0.05).
Protein scores are derived from ions scores as a non-probabilistic basis for ranking protein hits.

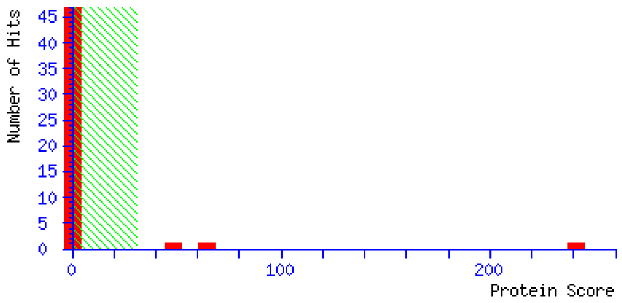

Matched peptide sequences: shown in Bold Red

```
  1 MAEGEEEVNI QEEANKGEEN PQEEANIQEE TNKGEENIQE EANIQEEANK
 51 EEESLKYLDF VQAATVYARA SFSKLYLFAK DKSGPFKPGV NTVESRFKSV
101 VRPVYNKFQP VPNKVLKFAD RRVDAYVTVL DRIVPPIVKR ASIQAYSVAP
151 GAARAVASYL PLHTKRLSKV LYGDG
```

Matched peptide information:

Start - End	Observed	Mr(expt)	Mr(calc)	ppm	Miss	Sequence
57 - 69	1516.8251	1515.8178	1515.7722	30	0	K.YLDFVQAATVYAR.A (Ions score 106)
99 - 107	1061.6381	1060.6308	1060.6029	26	1	K.SVVRPVYNK.F (Ions score 23)
122 - 132	1306.7440	1305.7367	1305.7041	25	1	R.RVDAYVTVLDR.I (Ions score 83)
123 - 132	1150.6491	1149.6418	1149.6030	34	0	R.VDAYVTVLDR.I (Ions score 60)
141 - 154	1361.7565	1360.7492	1360.7099	29	0	R.ASIQAYSVAPGAAR.A (Ions score 85)
155 - 165	1199.7106	1198.7033	1198.6710	27	0	R.AVASYLPLHTK.R (Ions score 34)

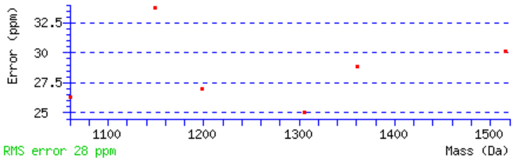

Spot No.:**154**

Accession No.: **scaffold3444_7763.mRNA1**

Protein name：**Elicitor-responsive protein 3**

Peptide sequences:

M.PLGTVEVLLVGAK.G;K.GLENTDFLNGVDPYVVLACR.T;K.GSEPEWNEK.F;K.FSFEVSDGDTELTLK.I;K.IMDSDVGAADDFVGEATIPLEPLFLEGNLPSTAYK.V;K.GEITVGLTFTPEVEMDNVGVDGYDFR.L

PFF Mascot score: **[467]** Sequence coverage %: **[84]**

Matched peptides No.: **[6]**

Calculated Mr: **15249** Calculated *p*I: **4.06**

Data base searched result:

Ions score is -10*Log(P), where P is the probability that the observed match is a random event.
Individual ions scores > 31 indicate identity or extensive homology (p<0.05).
Protein scores are derived from ions scores as a non-probabilistic basis for ranking protein hits.

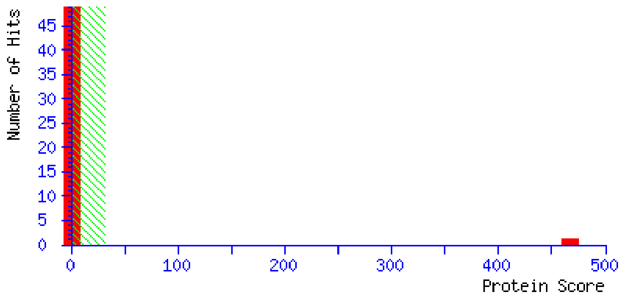

Matched peptide sequences: shown in Bold Red

```
  1 MPLGTVEVLL VGAKGLENTD FLNGVDPYVV LACRTQEQKS SVASGKGSEP
 51 EWNEKFSFEV SDGDTELTLK IMDSDVGAAD DFVGEATIPL EPLFLEGNLP
101 STAYKVVKEQ EYKGEITVGL TFTPEVEMDN VGVDGYDFRL
```

Matched peptide information:

Start - End	Observed	Mr(expt)	Mr(calc)	ppm	Miss	Sequence
2 - 14	1295.8228	1294.8155	1294.7860	23	0	M.PLGTVEVLLVGAK.G (Ions score 108)
15 - 34	2252.1633	2251.1561	2251.0943	27	0	K.GLENTDFLNGVDPYVVLACR.T (Ions score 53)
47 - 55	1075.4912	1074.4839	1074.4618	21	0	K.GSEPEWNEK.F (Ions score 53)
56 - 70	1687.8541	1686.8469	1686.7989	28	0	K.FSFEVSDGDTELTLK.I (Ions score 149)
71 - 105	3695.9111	3694.9039	3694.7964	29	0	K.IMDSDVGAADDFVGEATIPLEPLFLEGNLPSTAYK.V (Ions score 79)
114 - 139	2860.4143	2859.4070	2859.3273	28	0	K.GEITVGLTFTPEVEMDNVGVDGYDFR.L (Ions score 173)

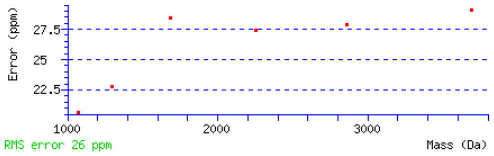

Spot No.:**155**

Accession No.: **scaffold1479_76107.mRNA1**

Protein name： **Acetyl-CoA acetyltransferase, cytosolic 1**

Peptide sequences:

R.ANVDPSLVQEVFFGNVLSANLGQAPAR.Q;K.DGLWDVFNDVGMGSCAEICADNHSITR.E;R.EDQDNYAIHSFER.G;R.GIAAQDSGAFAWEIVPVEVSGGR.G;K.VNVHGGAVSLGHPLGCSGAR.I;R.ILVTLLGVLR.Q

PFF Mascot score: **[523]** Sequence coverage %: **[29]**

Matched peptides No.: **[6]**

Calculated Mr: **41646** Calculated *p*I: **6.01**

Data base searched result:

Ions score is -10*Log(P), where P is the probability that the observed match is a random event.
Individual ions scores > 31 indicate identity or extensive homology (p<0.05).
Protein scores are derived from ions scores as a non-probabilistic basis for ranking protein hits.

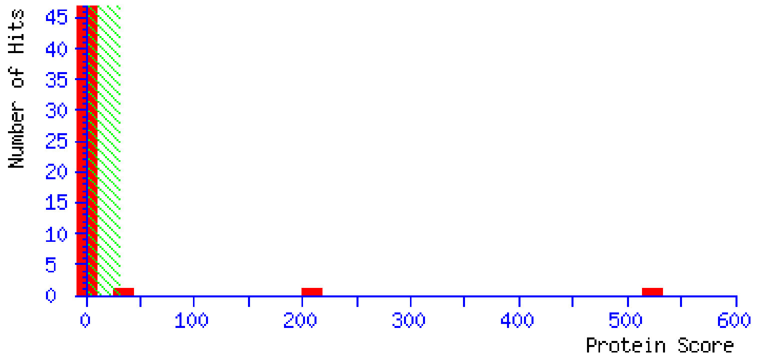

Matched peptide sequences: shown in Bold Red

```
  1 MAPVAAAEIK PRDVCIVGVA RTPMGGFLGL LSTLPATKLG SIAIEAALKR
 51 ANVDPSLVQE VFFGNVLSAN LGQAPARQAA LGAGIPNSVV CTTVNKVCAS
101 GMKATMLAAQ SIQLGINDVV VAGGMESMSN APKYLAEARK GSRLGHDSVV
151 DGMLKDGLWD VFNDVGMGSC AEICADNHSI TREDQDNYAI HSFERGIAAQ
201 DSGAFAWEIV PVEVSGGRGK PSTIVDKDEG LGKFDPVKLR KLRPSFKENG
251 GTVTAGNASS ISDGAAALVL VSGETALKLG LQVIAKITGY ADAAQAPELF
301 TTAPALAIPK AVSNAGLDAS QVDYYEINEA FAVVALANQK LLGLNPEKVN
351 VHGGAVSLGH PLGCSGARIL VTLLGVLRQK NGKYGVGGVC NGGGGASALV
401 VELL
```

Matched peptide information:

Start - End	Observed	Mr(expt)	Mr(calc)	ppm	Miss	Sequence
51 - 77	2813.5635	2812.5562	2812.4508	37	0	R.ANVDPSLVQEVFFGNVLSANLGQAPAR.Q (Ions score 147)
156 - 182	3039.4089	3038.4017	3038.2957	35	0	K.DGLWDVFNDVGMGSCAEICADNHSITR.E (Ions score 86)
183 - 195	1623.7706	1622.7634	1622.6961	41	0	R.EDQDNYAIHSFER.G (Ions score 101)
196 - 218	2316.2549	2315.2476	2315.1546	40	0	R.GIAAQDSGAFAWEIVPVEVSGGR.G (Ions score 194)
349 - 368	1945.0641	1944.0568	1943.9748	42	0	K.VNVHGGAVSLGHPLGCSGAR.I (Ions score 80)
369 - 378	1096.7860	1095.7787	1095.7380	37	0	R.ILVTLLGVLR.Q (Ions score 57)

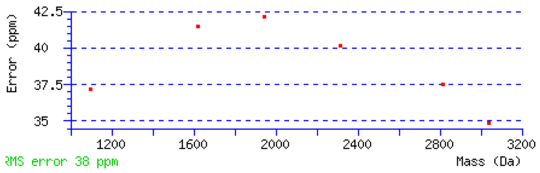

RMS error 38 ppm

Spot No.:**156**

Accession No.: **scaffold1375_37486.mRNA1**

Protein name：**Profilin-1**

Peptide sequences: **K.YMVIQGEAGAVIR.G;K.TNQALIIGIYDEPMTPGQCNMIVER.L**

PFF Mascot score: **[182]** Sequence coverage %: **[29]**

Matched peptides No.: **[2]**

Calculated Mr: **14238** Calculated *p*I: **4.63**

Data base searched result:

Ions score is -10*Log(P), where P is the probability that the observed match is a random event.
Individual ions scores > 30 indicate identity or extensive homology (p<0.05).
Protein scores are derived from ions scores as a non-probabilistic basis for ranking protein hits.

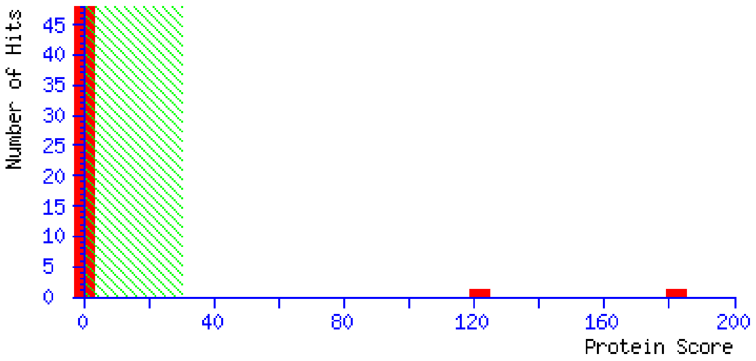

Matched peptide sequences: shown in Bold Red

```
  1 MSWQTYVDEH LMCEIEGNHL TAAAIIGQDG SVWAQSSNFP QFKSEEITAI
 51 MSDFDEPGTL APTGLHLGGT KYMVIQGEAG AVIRGKKGPG GVTVKKTNQA
101 LIIGIYDEPM TPGQCNMIVE RLGDYLMDQG L
```

Matched peptide information:

Start - End	Observed	Mr(expt)	Mr(calc)	ppm	Miss	Sequence
72 - 84	1406.7952	1405.7879	1405.7388	35	0	K.YMVIQGEAGAVIR.G (Ions score 124)
97 - 121	2863.4705	2862.4632	2862.3714	32	0	K.TNQALIIGIYDEPMTPGQCNMIVER.L (Ions score 88)

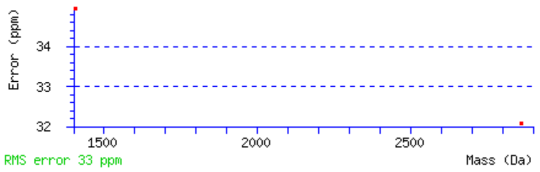

Spot No.:**157**

Accession No.: **scaffold0434_14371.mRNA1**

Protein name：**Glutathione S-transferase F9**

Peptide sequences:

K.LQPFGALPVIQDGDYTLYESR.A;K.VLDVYEER.L;K.YLAGDFFSLADLSHLPFTQYLVGPINK.E;K.HVSAWWDQISSR.P

PFF Mascot score: **[213]** Sequence coverage %: **[30]**

Matched peptides No.: **[4]**

Calculated Mr: **25501** Calculated *p*I: **8.44**

Data base searched result:

Ions score is -10*Log(P), where P is the probability that the observed match is a random event.
Individual ions scores > 31 indicate identity or extensive homology (p<0.05).
Protein scores are derived from ions scores as a non-probabilistic basis for ranking protein hits.

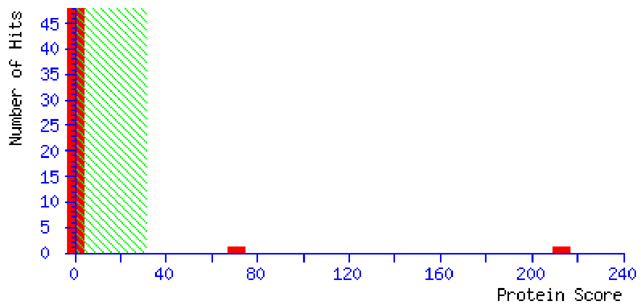

Matched peptide sequences: shown in **Bold Red**

```
  1 MVVKVYGTAY ASPKRVLACL IEKGIEFEAV PVDLIKGEHR SPEYLKLQPF
 51 GALPVIQDGD YTLYESRAII RYYAEKYKSQ GTDLLGKSIE ERGLVEQWLE
101 VEAQNFHPHI YNLTLHILFA SALGFPPDEK VIKESEEKLG KVLDVYEERL
151 SKSKYLAGDF FSLADLSHLP FTQYLVGPIN KEYMIRSRKH VSAWWDQISS
201 RPSWKKAAGH LGRRSSNFKM MS
```

Matched peptide information:

Start - End	Observed	Mr(expt)	Mr(calc)	ppm	Miss	Sequence
47 - 67	2382.2937	2381.2864	2381.1903	40	0	K.LQPFGALPVIQDGDYTLYESR.A (Ions score 153)
142 - 149	1022.5476	1021.5403	1021.5080	32	0	K.VLDVYEER.L (Ions score 50)
155 - 181	3026.6775	3025.6702	3025.5589	37	0	K.YLAGDFFSLADLSHLPFTQYLVGPINK.E (Ions score 48)
190 - 201	1471.7573	1470.7500	1470.7004	34	0	K.HVSAWWDQISSR.P (Ions score 33)

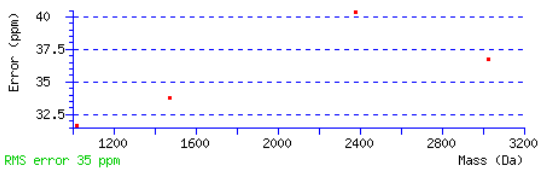

Spot No.:**158**

Accession No.: **scaffold0427_434785.mRNA1**

Protein name：**Superoxide dismutase [Mn], mitochondrial**

Peptide sequences:

K.FNGGGHVNHSIFWK.N;R.EGGGEPPHSSLGWAIDTDFGSLEK.L;K.INAEGAALQGSGWVWLALDK.E;K.GPSLIPLLGIDVWEHAYYLQYK.N;K.NVRPDYLK.N

PFF Mascot score: **[393]** Sequence coverage %: **[37]**

Matched peptides No.: **[5]**

Calculated Mr: **26153** Calculated *p*I: **7.82**

Data base searched result:

Ions score is -10*Log(P), where P is the probability that the observed match is a random event.
Individual ions scores > 30 indicate identity or extensive homology (p<0.05).
Protein scores are derived from ions scores as a non-probabilistic basis for ranking protein hits.

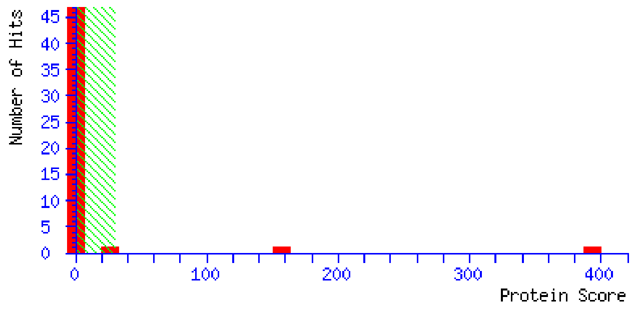

Matched peptide sequences: shown in Bold Red

```
  1 MALRSLVARK TISSAFKAAT GLRLGQLRGI QTYSLPDLPY DYGALEPVIS
 51 GEIMQLHHQK HHQTYVTNFN TALEQLNDAM EKGDPAAVVK LQSAIKFNGG
101 GHVNHSIFWK NLAPVREGGG EPPHSSLGWA IDTDFGSLEK LIQKINAEGA
151 ALQGSGWVWL ALDKELKKLV VETTANQDPL VTKGPSLIPL LGIDVWEHAY
201 YLQYKNVRPD YLKNIWKVMN WKYASEVYAK ECPSS
```

Matched peptide information:

Start - End	Observed	Mr(expt)	Mr(calc)	ppm	Miss	Sequence
97 - 110	1599.8340	1598.8267	1598.7743	33	0	K.FNGGGHVNHSIFWK.N (Ions score 78)
117 - 140	2486.2393	2485.2320	2485.1398	37	0	R.EGGGEPPHSSLGWAIDTDFGSLEK.L (Ions score 144)
145 - 164	2099.1626	2098.1553	2098.0847	34	0	K.INAEGAALQGSGWVWLALDK.E (Ions score 126)
184 - 205	2575.4517	2574.4444	2574.3522	36	0	K.GPSLIPLLGIDVWEHAYYLQYK.N (Ions score 128)
206 - 213	1004.5823	1003.5751	1003.5451	30	1	K.NVRPDYLK.N (Ions score 32)

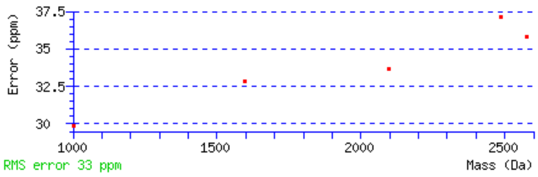

Spot No.:**159**

Accession No.: **scaffold0168_73087.mRNA1**

Protein name： **Abscisic stress-ripening protein 1**

Peptide sequences: **K.HHHHHPFHHHK.D; R.HKIEEEAAAALAVGAGGFAFHEK.H**

PFF Mascot score: **[136]** Sequence coverage %: **[24]**

Matched peptides No.: **[2]**

Calculated Mr: **15800** Calculated *p*I: **6.13**

Data base searched result:

Ions score is -10*Log(P), where P is the probability that the observed match is a random event.
Individual ions scores > 30 indicate identity or extensive homology (p<0.05).
Protein scores are derived from ions scores as a non-probabilistic basis for ranking protein hits.

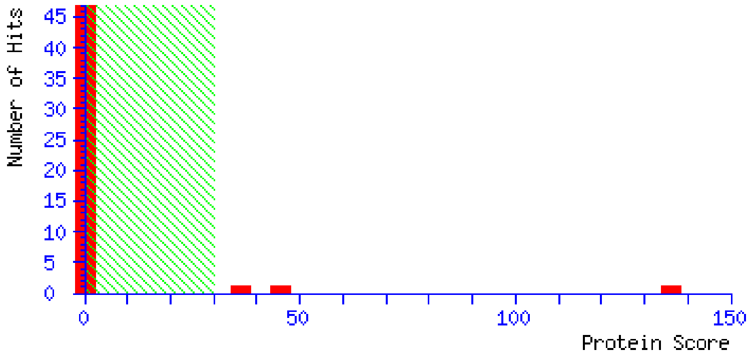

Matched peptide sequences: shown in Bold Red

```
  1 MAEEKHHHHH PFHHHKDEEK PIETAAVYSE TSEFSETTAY PTNIPPPHDA
 51 PDHKKEEKHH KHLEHVGELG AAAAGAYALY EKHEAKKDPE HAHRHKIEEE
101 AAAALAVGAG GFAFHEKHEK KEAKREDEEA HGKKQHHLF
```

Matched peptide information:

Start - End	Observed	Mr(expt)	Mr(calc)	ppm	Miss	Sequence	
6 - 16	1487.7816	1486.7743	1486.6980	51	0	K.HHHHHPFHHHK.D	(Ions score 47)
95 - 117	2353.3240	2352.3167	2352.1862	55	1	R.HKIEEEAAAALAVGAGGFAFHEK.H	(Ions score 118)

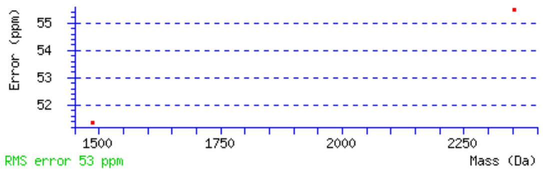

Spot No.:**160**

Accession No.: **scaffold0135_1578719.mRNA1**

Protein name：**Probable glutathione S-transferase**

Peptide sequences:

K.SPLLLQMNPVHK.K;K.IPVLIHNGKPICESLIAVQYVDEVWK.D;R.FWADFIDK.K;R.FWADFIDKK.I;K.VYEFVLVLK.K

PFF Mascot score: **[267]** Sequence coverage %: **[25]**

Matched peptides No.: **[5]**

Calculated Mr: **25563** Calculated *p*I: **6.23**

Data base searched result:

Ions score is -10*Log(P), where P is the probability that the observed match is a random event.
Individual ions scores > 31 indicate identity or extensive homology (p<0.05).
Protein scores are derived from ions scores as a non-probabilistic basis for ranking protein hits.

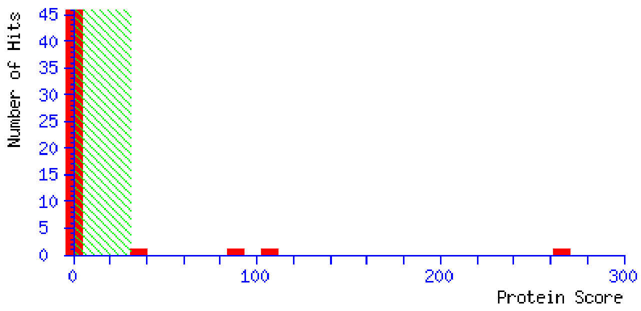

Matched peptide sequences: shown in Bold Red

```
  1 MAEEVILLDF WSSPFGMRVR IALAEKGVKY EYREEDLRNK SPLLLQMNPV
 51 HKKIPVLIHN GKPICESLIA VQYVDEVWKD KSPLLPSDPY QRAQARFWAD
101 FIDKKIYDIG RKIWTTKGDE QEAAKKEFIE ALKLLEGELG NKPYFGGESM
151 GYVDVALIPF YSWFYAYETC GNFSIEPECP VLIAWAKRCL QKESVSKSLP
201 DPQKVYEFVL VLKKKFGIE
```

Matched peptide information:

Start - End	Observed	Mr(expt)	Mr(calc)	ppm	Miss	Sequence
41 - 52	1376.8154	1375.8082	1375.7646	32	0	K.SPLLLQMNPVHK.K (Ions score 83)
54 - 79	3020.7327	3019.7254	3019.6205	35	1	K.IPVLIHNGKPICESLIAVQYVDEVWK.D (Ions score 128)
97 - 104	1041.5253	1040.5180	1040.4967	20	0	R.FWADFIDK.K (Ions score 50)
97 - 105	1169.6270	1168.6197	1168.5917	24	1	R.FWADFIDKK.I (Ions score 65)
205 - 213	1109.6831	1108.6758	1108.6532	20	0	K.VYEFVLVLK.K (Ions score 67)

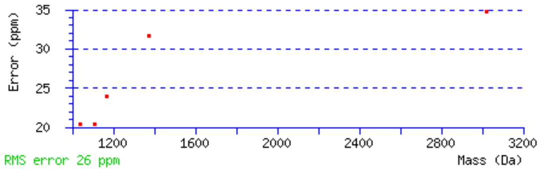

Spot No.:**161**

Accession No.: **scaffold0299_222985.mRNA1**

Protein name: **Aspartic proteinase A1**

Peptide sequences: **K.GEEGGEIVFGGIDSNHYK.G;K.AIADSGTSLLAGPTTVITQINR.A**

PFF Mascot score: **[211]** Sequence coverage %: **[7]**

Matched peptides No.: **[2]**

Calculated Mr: **55900** Calculated *p*I: **5.03**

Data base searched result:

Ions score is -10*Log(P), where P is the probability that the observed match is a random event.
Individual ions scores > 31 indicate identity or extensive homology (p<0.05).
Protein scores are derived from ions scores as a non-probabilistic basis for ranking protein hits.

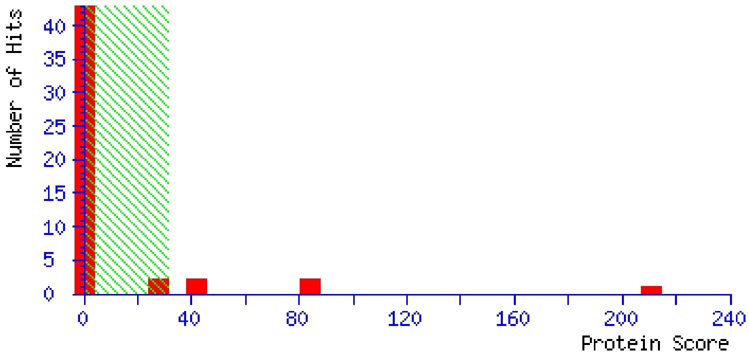

Matched peptide sequences: shown in Bold Red

```
  1 MGTKFSALWM AFLLLFPVVL STHNGGLIRI GLKKKKLDQV NHPVGTLDST
 51 EGEAMRVATK KYNIYDNIGN SGDTDVVALK NYLDAQYYGE IAIGTPSQTF
101 TVIFDTGSSN LWVPSSKCYF SLACYFHSKY KSSESSTYEK NGTSAAIQYG
151 TGSIAGFFSQ DNVKVGDFVV RNQDFIEATK EPGVTFLAAK FDGILGLGFQ
201 EISVGKAVPV WYNMVNQGLV TEQVFSFWLN RNVKGEEGGE IVFGGIDSNH
251 YKGEHTYVPV TQKGYWQFDM GDILVGNEST GLCGNGCKAI ADSGTSLLAG
301 PTTVITQINR AIGASGIVSQ ECKTVVAQYG KVILEMLMAQ AKPQKICSQI
351 GFCTFDGARG VSTNIESVVD ETTDEVSNGV QDAMCAACEM MIVWMQNRIK
401 LNQTEDQILN YVNELCDRLP SPNGESAVDC GSLSSMPHVS FTIGGKVFGL
451 TPEQYVLKVG EGVSAQCISG FTALDVPPPH GPLWILGDVF MGPYHTVFDY
501 GNLRVGFAEA A
```

Matched peptide information:

Start - End	Observed	Mr(expt)	Mr(calc)	ppm	Miss	Sequence
235 - 252	1907.9475	1906.9402	1906.8697	37	0	K.GEEGGEIVFGGIDSNHYK.G (Ions score 84)
289 - 310	2199.2810	2198.2737	2198.1907	38	0	K.AIADSGTSLLAGPTTVITQINR.A (Ions score 155)

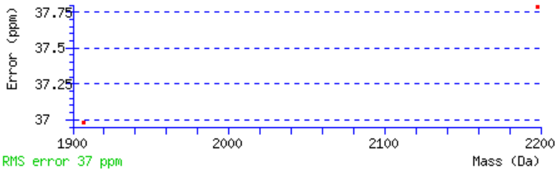

Spot No.:**162**

Accession No.: **scaffold1222_175215.mRNA1**

Protein name：**Rubber elongation factor protein**

Peptide sequences: **K.FADNQVDASVTLVLR.Y;R.AALALVSYLPLPTNR.L**

PFF Mascot score: **[178]** Sequence coverage %: **[13]**

Matched peptides No.: **[2]**

Calculated Mr: **23621** Calculated *p*I: **5.33**

Data base searched result:

Ions score is -10*Log(P), where P is the probability that the observed match is a random event.
Individual ions scores > 31 indicate identity or extensive homology ($p<0.05$).
Protein scores are derived from ions scores as a non-probabilistic basis for ranking protein hits.

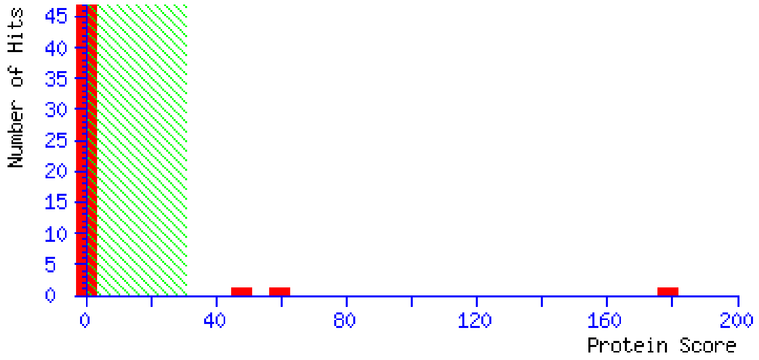

Matched peptide sequences: shown in Bold Red

```
  1 MASLLGAASN VINAASNVVE EAVKGVGNAQ QEVANAVSNP SNIVKDVASA
 51 ATDIVEEAAK VVDNVQQGVV SAASNVVEEA AKGVGNIQEK VDDEEEDTLK
101 YLDIVQAALV LALVSSSKLY LFVKDKSGPL KPGVDTAEVT IKSVVRPFYY
151 RFHDVPNKVL KFADNQVDAS VTLVLRYAPP VVKQVSTRAY SVARNAPRAA
201 LALVSYLPLP TNRLCKLLSE DK
```

Matched peptide information:

Start - End	Observed	Mr(expt)	Mr(calc)	ppm	Miss	Sequence	
162 - 176	1647.9070	1646.8997	1646.8628	22	0	K.FADNQVDASVTLVLR.Y	(Ions score 137)
199 - 213	1598.9597	1597.9524	1597.9191	21	0	R.AALALVSYLPLPTNR.L	(Ions score 73)

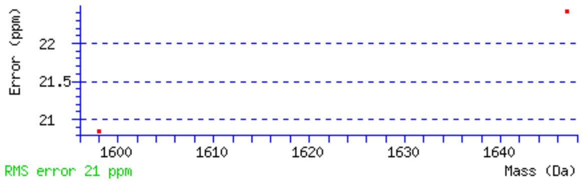

Spot No.:**163**

Accession No.: **scaffold0888_274749.mRNA1**

Protein name：**Putative lactoylglutathionelyase**

Peptide sequences:

R.FLHVVYR.V;K.FYTECFGMK.L;K.DPDGYIFELIQR.G;R.GPTPEPLCQVMLR.V;K.GNAYAQVAIGTDDVYK.S;K.SAEVVNLVTQELGGK.I;K.ITSFLDPDGWK.T

PFF Mascot score: **[380]** Sequence coverage %: **[28]**

Matched peptides No.: **[7]**

Calculated Mr: **32858** Calculated *p*I: **5.06**

Data base searched result:

Ions score is -10*Log(P), where P is the probability that the observed match is a random event.
Individual ions scores > 31 indicate identity or extensive homology (p<0.05).
Protein scores are derived from ions scores as a non-probabilistic basis for ranking protein hits.

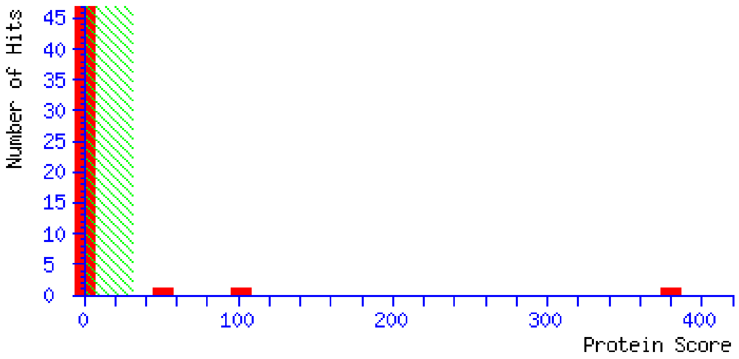

Matched peptide sequences: shown in Bold Red

```
  1 MAEVAPNAEL LEWPKKDKRR FLHVVYRVGD LDRTIKFYTE CFGMKLLRKR
 51 DVPEEKYSNA FLGFGSEETN FVVELTYNYG VTSYDIGDGF GHFAIATQDV
101 YKLVEDIRAK GGIISREPGP VKGGTTVIAF AKDPDGYIFE LIQRGPTPEP
151 LCQVMLRVGD LDRSIQFYEK ALGMKFLRKV DNPQYKYTLA MMGYADEYET
201 TVLELTYNYG VTEYSKGNAY AQVAIGTDDV YKSAEVVNLV TQELGGKITR
251 QPGPLPGINT KITSFLDPDG WKTVLVDNED FLKELQKGDS A
```

Matched peptide information:

Start - End	Observed	Mr(expt)	Mr(calc)	ppm	Miss	Sequence
21 - 27	933.5487	932.5414	932.5232	20	0	R.FLHVVYR.V (Ions score 32)
37 - 45	1182.5283	1181.5210	1181.4886	27	0	K.FYTECFGMK.L (Ions score 50)
133 - 144	1465.7834	1464.7762	1464.7249	35	0	K.DPDGYIFELIQR.G (Ions score 114)
145 - 157	1497.8112	1496.8039	1496.7480	37	0	R.GPTPEPLCQVMLR.V (Ions score 86)
217 - 232	1684.8834	1683.8761	1683.8104	39	0	K.GNAYAQVAIGTDDVYK.S (Ions score 88)
233 - 247	1543.8859	1542.8786	1542.8253	35	0	K.SAEVVNLVTQELGGK.I (Ions score 92)
262 - 272	1278.6768	1277.6695	1277.6292	32	0	K.ITSFLDPDGWK.T (Ions score 97)

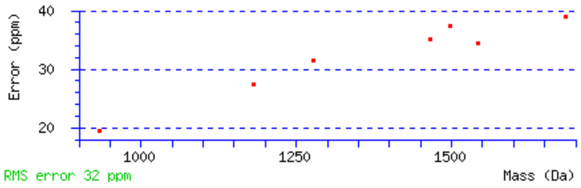

Spot No.:**164**

Accession No.: **scaffold1102_118530.mRNA1**

Protein name：**Proteasome subunit alpha type-2-A**

Peptide sequences:

K.IQILTPNIGVVYSGMGPDFR.V;R.LYKEPIPVTQLVR.E;K.EPIPVTQLVR.E;R.ETAAVMQEFTQSGGVR.P;R.ETAAVMQEFTQSGGVRPFGVSLLVAGYDDK.G

PFF Mascot score: **[245]** Sequence coverage %: **[55]**

Matched peptides No.: **[5]**

Calculated Mr: **25614** Calculated *p*I: **5.73**

Data base searched result:

Ions score is -10*Log(P), where P is the probability that the observed match is a random event.
Individual ions scores > 31 indicate identity or extensive homology (p<0.05).
Protein scores are derived from ions scores as a non-probabilistic basis for ranking protein hits.

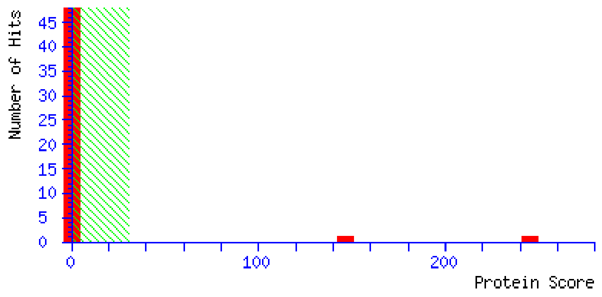

Matched peptide sequences: shown in Bold Red

```
  1 MGDSQYSFSL TTFSPSGKLV QIEHALTAVG SGQTSLGIKA ANGVVIATEK
 51 KLPSILVDET SVQKIQILTP NIGVVYSGMG PDFRVLVRKS RKQAEQYHRL
101 YKEPIPVTQL VRETAAVMQE FTQSGGVRPF GVSLLVAGYD DKGPQLYQVD
151 PSGSYFSWKA SAMGKNVSNA KTFLEKRYTD DMELDDAVHT AILTLKEGFE
201 GQISGKNIEI GIIGADKKFR VLTSAEIDDY LAEVE
```

Matched peptide information:

Start - End	Observed	Mr(expt)	Mr(calc)	ppm	Miss	Sequence
65 - 84	2177.2549	2176.2476	2176.1351	52	0	K.IQILTPNIGVVYSGMGPDFR.V (Ions score 53)
100 - 112	1555.9998	1554.9925	1554.9133	51	1	R.LYKEPIPVTQLVR.E (Ions score 84)
103 - 112	1151.7327	1150.7254	1150.6710	47	0	K.EPIPVTQLVR.E (Ions score 61)
113 - 128	1710.8988	1709.8915	1709.8043	51	0	R.ETAAVMQEFTQSGGVR.P (Ions score 67)
113 - 142	3172.6892	3171.6819	3171.5547	40	1	R.ETAAVMQEFTQSGGVRPFGVSLLVAGYDDK.G (Ions score 79)

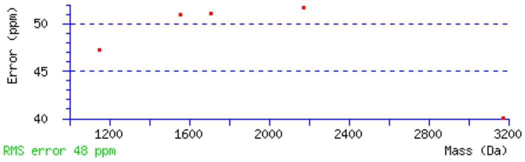

Spot No.:**165**

Accession No.: **scaffold0582_11826.mRNA1**

Protein name：**Pyrophosphate--fructose 6-phosphate 1-phosphotransferase subunit beta**

Peptide sequences:

R.FASVYSELQASR.I;K.IGVVLSGGQAPGGHNVISGIFDYLQER.A;K.YVELTADFIYPYR.N;R.DKIETPEQFK.Q;K.IYSEMIGNVMIDAR.S;K.LTSQSLQLFEFLPLAIR.E;K.GQSHFFGYEGR.C

PFF Mascot score: **[313]** Sequence coverage %: **[18]**

Matched peptides No.: **[7]**

Calculated Mr: **62382** Calculated *p*I: **7.18**

Data base searched result:

Ions score is -10*Log(P), where P is the probability that the observed match is a random event.
Individual ions scores > 31 indicate identity or extensive homology (p<0.05).
Protein scores are derived from ions scores as a non-probabilistic basis for ranking protein hits.

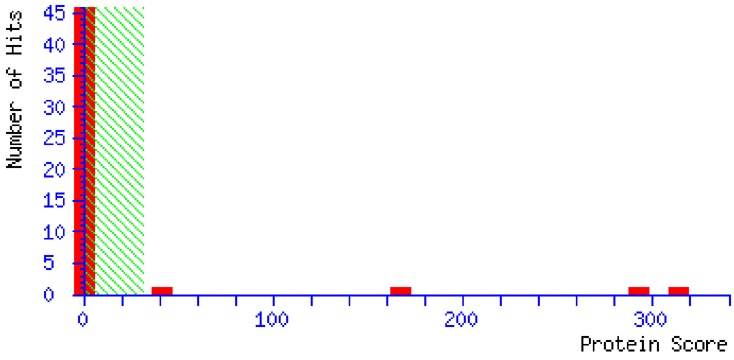

Matched peptide sequences: shown in Bold Red

```
  1 MAPSFVINGE VPTVKSSPAT GRFASVYSEL QASRIDHSLP VPSVLKNPFK
 51 VVEGPPSSAA GNPDEIAKLF PNLFGQPSSM FVPNGADAVH SNLKLKIGVV
101 LSGGQAPGGH NVISGIFDYL QERAKGSILY GFRGGPAGIM KCKYVELTAD
151 FIYPYRNQGG FDMICSGRDK IETPEQFKQA EETALKLDLD GLVVIGGDDS
201 NTNACLLAEN FRTKNMKTRV IGCPKTIDGD LKCKEVPTSF GFDTACKIYS
251 EMIGNVMIDA RSTGKYYHFV RLMGRAASHI TLECALQTHP NITIIGEEVA
301 AKKLTLKNVT DYIVDIICKR ADLGYNYGVI LIPEGLIDFI PEVQHLIAEL
351 NEILARDVVD EGGLWKKKLT SQSLQLFEFL PLAIREQLML ERDPHGNVQV
401 AKIETEKMLI QMVETELEKR KQQGSYKAQF KGQSHFFGYE GRCGLPTNFD
451 ATYCYALGYG AGALLHSGKT GLISSVGNLG APVAEWTVGG TALTSMMDVE
501 RRHGKFKPVI KKAMVELEGA PFKKFASMRN EWALKNRYIS PGPIQFMGPG
551 SDAVNHTLLL ELGVHA
```

Matched peptide information:

Start - End	Observed	Mr(expt)	Mr(calc)	ppm	Miss	Sequence
23 - 34	1357.7308	1356.7236	1356.6674	41	0	R.FASVYSELQASR.I (Ions score 54)
97 - 123	2783.5642	2782.5569	2782.4402	42	0	K.IGVVLSGGQAPGGHNVISGIFDYLQER.A (Ions score 65)
144 - 156	1649.8954	1648.8881	1648.8137	45	0	K.YVELTADFIYPYR.N (Ions score 93)
169 - 178	1234.6804	1233.6731	1233.6241	40	1	R.DKIETPEQFK.Q (Ions score 58)
248 - 261	1611.8594	1610.8521	1610.7796	45	0	K.IYSEMIGNVMIDAR.S (Ions score 84)
369 - 385	1976.2034	1975.1961	1975.1142	41	0	K.LTSQSLQLFEFLPLAIR.E (Ions score 66)
432 - 442	1284.6302	1283.6230	1283.5683	43	0	K.GQSHFFGYEGR.C (Ions score 68)

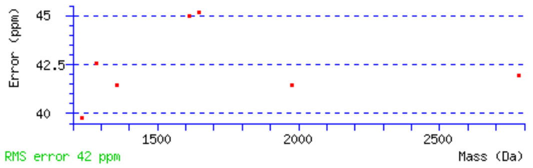

Spot No.:**166**

Accession No.: **scaffold1222_136753.mRNA1**

Protein name：**Rubber elongation factor protein**

Peptide sequences:

K.YLGFVQDAATYAVTTFSNVYLFAK.D;K.DKSGPLQPGVDIIEGPVK.N;K.NVAVPLYNR.F;R.FSYIPNGALK.F;K.FVDSTVVASVTIIDR.S;K.DASIQVVSAIR.A

PFF Mascot score: **[368]** Sequence coverage %: **[63]**

Matched peptides No.: **[6]**

Calculated Mr: **14713** Calculated *p*I: **5.04**

Data base searched result:

Ions score is -10*Log(P), where P is the probability that the observed match is a random event.
Individual ions scores > 31 indicate identity or extensive homology (p<0.05).
Protein scores are derived from ions scores as a non-probabilistic basis for ranking protein hits.

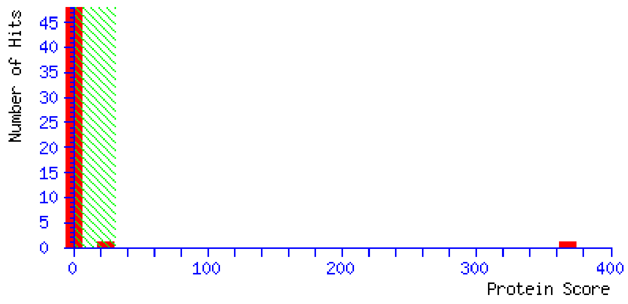

Matched peptide sequences: shown in Bold Red

```
  1 MAEDEDNQQG QGEGLKYLGF VQDAATYAVT TFSNVYLFAK DKSGPLQPGV
 51 DIIEGPVKNV AVPLYNRFSY IPNGALKFVD STVVASVTII DRSLPPIVKD
101 ASIQVVSAIR AAPEAARSLA SSLPGQTKIL AKVFYGEN
```

Matched peptide information:

Start - End	Observed	Mr(expt)	Mr(calc)	ppm	Miss	Sequence
17 - 40	2689.4204	2688.4131	2688.3476	24	0	K.YLGFVQDAATYAVTTFSNVYLFAK.D (Ions score 104)
41 - 58	1849.0483	1848.0411	1847.9993	23	1	K.DKSGPLQPGVDIIEGPVK.N (Ions score 55)
59 - 67	1045.5978	1044.5905	1044.5716	18	0	K.NVAVPLYNR.F (Ions score 68)
68 - 77	1109.6137	1108.6064	1108.5917	13	0	R.FSYIPNGALK.F (Ions score 47)
78 - 92	1621.9231	1620.9158	1620.8723	27	0	K.FVDSTVVASVTIIDR.S (Ions score 145)
100 - 110	1158.6746	1157.6673	1157.6404	23	0	K.DASIQVVSAIR.A (Ions score 99)

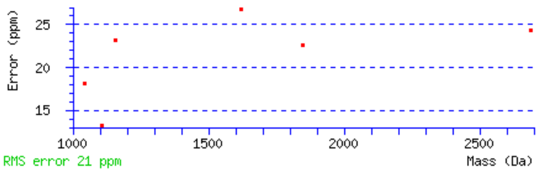

Spot No.:**167**

Accession No.: **scaffold0530_34184.mRNA1**

Protein name：**Heat shock 70 kDa protein 5**

Peptide sequences:

K.AIGIDLGTTYSCVGVWQIDR.V;R.TTPSYVAFTDTER.L;K.NAVITVPAYFNDSQR.Q;R.TLSSTTQTTIEIDSL YEGIDFYATITR.A;R.ARFEELNMDLFR.K;R.FEELNMDLFR.K;K.EQIFSTYSDNQTSVLIQVYEGER.A;K.LEN YAYNMR.N

PFF Mascot score: **[604]** Sequence coverage %: **[18]**

Matched peptides No.: **[8]**

Calculated Mr: **72149** Calculated *p*I: **5.36**

Data base searched result:

Ions score is -10*Log(P), where P is the probability that the observed match is a random event.
Individual ions scores > 30 indicate identity or extensive homology (p<0.05).
Protein scores are derived from ions scores as a non-probabilistic basis for ranking protein hits.

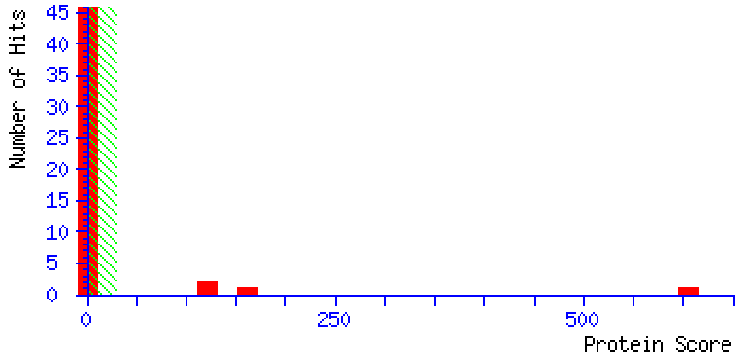

Matched peptide sequences: shown in Bold Red

```
  1 MASKSEGKAI GIDLGTTYSC VGVWQIDRVE IIANDQGNRT TPSYVAFTDT
 51 ERLIGDAAKN QVAMNPQNTV FDAKRLIGRR FSDPTVQSDM KHWPFKVISG
101 PGDKPMIVVQ YKGEEKQFAP EEISSMVLTK MKEIAEAYLG HTVKNAVITV
151 PAYFNDSQRQ ATKDAGAIAG LNVMRIINEP TAAAIAYGLD KKGSRTGEKN
201 VLIFDLGGGT FDVSLLTIEE GIFEVKATAG DTHLGGEDFD NRLVNHFVAE
251 FKRKHKKDIS TNARALRRLR TACERAKRTL SSTTQTTIEI DSLYEGIDFY
301 ATITRARFEE LNMDLFRKCM EPVEKCLRDA KIDKSQVHDI VLVGGSTRIP
351 KVQQLLLDFF NGKELCKSIN PDEAVAYGAA VQAAILTGEG DQKVQDLLLL
401 DVTPLSLGIE TAGGVMTVLI PRNTTIPAKK EQIFSTYSDN QTSVLIQVYE
451 GERARTKDNN LLGTFELKGI PPAPRGVPQI SVCFDIDANG ILNVSAEDKT
501 AGVKNKITIT NDKGRLSKEE IERMVQEAEK YKAEDEEVKK KVEAKNKLEN
551 YAYNMRNTVK DEKIAGKLNP ADKQKIEKAI DETIEWLDGN QLAEVDEFED
601 KLKELEGLCN PIISKMYQGG GGEDVPMGGG AQPGSGYGPS SSGGSGAGPK
651 IEEVD
```

Matched peptide information:

Start - End	Observed	Mr(expt)	Mr(calc)	ppm	Miss	Sequence
9 - 28	2224.1648	2223.1575	2223.0994	26	0	K.AIGIDLGTTYSCVGVWQIDR.V (Ions score 152)
40 - 52	1487.7353	1486.7281	1486.6940	23	0	R.TTPSYVAFTDTER.L (Ions score 99)
145 - 159	1694.8928	1693.8855	1693.8424	25	0	K.NAVITVPAYFNDSQR.Q (Ions score 133)
279 - 305	3039.5737	3038.5665	3038.4972	23	0	R.TLSSTTQTTIEIDSLYEGIDFYATITR.A (Ions score 99)
306 - 317	1540.7919	1539.7846	1539.7504	22	1	R.ARFEELNMDLFR.K (Ions score 42)
308 - 317	1313.6434	1312.6362	1312.6122	18	0	R.FEELNMDLFR.K (Ions score 70)
431 - 453	2706.3579	2705.3506	2705.2820	25	0	K.EQIFSTYSDNQTSVLIQVYEGER.A (Ions score 159)
548 - 556	1173.5496	1172.5423	1172.5284	12	0	K.LENYAYNMR.N (Ions score 57)

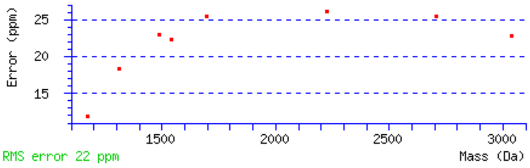

Spot No.:168

Accession No.: **scaffold0470_417147.mRNA1**

Protein name：**Phosphoinositide phospholipase C 2**

Peptide sequences:

R.VIELDIWPNSNKDNVEVLHGR.T;R.TLTAPVELIK.C;R.SIQEHAFTASDYPVIITLEDHLTPDLQAK.V;K.SSPHGDVFNPR.V;K.VYMGEGWFNDFQGK.S;K.SFDAFSSPDFYVR.V;K.FPLTVPELALLR.I;R.IEVYDYDMSEKDDFAGQTCLPVFELR.R;K.DDFAGQTCLPVFELR.R

PFF Mascot score: **[614]** Sequence coverage %: **[24]**

Matched peptides No.: **[9]**

Calculated Mr: **63738** Calculated *p*I: **5.38**

Data base searched result:

Ions score is -10*Log(P), where P is the probability that the observed match is a random event.
Individual ions scores > 31 indicate identity or extensive homology (p<0.05).
Protein scores are derived from ions scores as a non-probabilistic basis for ranking protein hits.

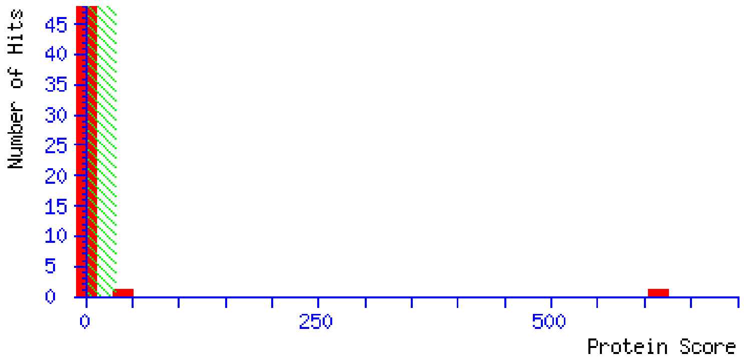

Matched peptide sequences: shown in Bold Red

```
  1 MAVAVAPEEI KALFERYSDN GIMTIDHLRR FLVEVQKEDK ATTGDAQDIF
 51 YHLHELQLGL NFEAFFKYLF GDINPPLDLK RGVHHDMTAP LSHYFIFTGH
101 NSYLTGNQLS SDCSDVPIID ALQSGVRVIE LDIWPNSNKD NVEVLHGRTL
151 TAPVELIKCL RSIQEHAFTA SDYPVIITLE DHLTPDLQAK VAEMITQTFG
201 DILFSPGSEC LKEFPSPEFL KKRIIISTKP PKEYLEAKQT KHRDSEEAYG
251 KETSDLKGHI QAGDKNDLDE DSDDEEDADE GIMRSTRNVV PEYKQLIAIH
301 AGKPKGGLEE CLKVDPEKVR RLSLSEPQLE KAAETYGKEI VRFSQRNILR
351 VYPKGIRINS SNYNPLIGWT HGAQMVAFNM QGHGKSLWLM RGMFRANGGC
401 GYVKKPDFLL KSSPHGDVFN PRVELPVKTI LKVKVYMGEG WFNDFQGKSF
451 DAFSSPDFYV RVGIAGVPAD TVMKETETQM NNWMPVWNEE FKFPLTVPEL
501 ALLRIEVYDY DMSEKDDFAG QTCLPVFELR RGIRAVPLYN FDGVKYNSIK
551 LLMQFDFI
```

Matched peptide information:

Start	-	End	Observed	Mr(expt)	Mr(calc)	ppm	Miss	Sequence
128	-	148	2447.3289	2446.3216	2446.2605	25	1	R.VIELDIWPNSNKDNVEVLHGR.T (Ions score 108)
149	-	158	1084.6733	1083.6661	1083.6539	11	0	R.TLTAPVELIK.C (Ions score 63)
162	-	190	3252.7151	3251.7078	3251.6350	22	0	R.SIQEHAFTASDYPVIITLEDHLTPDLQAK.V (Ions score 74)
412	-	422	1212.6019	1211.5947	1211.5683	22	0	K.SSPHGDVFNPR.V (Ions score 86)
435	-	448	1677.7789	1676.7717	1676.7293	25	0	K.VYMGEGWFNDFQGK.S (Ions score 90)
449	-	461	1537.7415	1536.7342	1536.6885	30	0	K.SFDAFSSPDFYVR.V (Ions score 76)
493	-	504	1368.8589	1367.8516	1367.8177	25	0	K.FPLTVPELALLR.I (Ions score 102)
505	-	530	3140.4944	3139.4871	3139.4155	23	1	R.IEVYDYDMSEKDDFAGQTCLPVFELR.R (Ions score 150)
516	-	530	1767.8872	1766.8799	1766.8298	28	0	K.DDFAGQTCLPVFELR.R (Ions score 94)

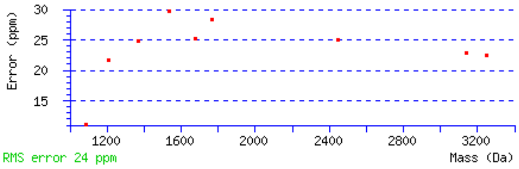

Spot No.:**169**

Accession No.: **scaffold0470_417147.mRNA1**

Protein name： **Phosphoinositide phospholipase C 2**

Peptide sequences:

R.VIELDIWPNSNKDNVEVLHGR.T;R.SIQEHAFTASDYPVIITLEDHLTPDLQAK.V;K.SSPHGDVFNPR.V;K.VYMGEGWFNDFQGK.S;K.SFDAFSSPDFYVR.V;K.FPLTVPELALLR.I;R.IEVYDYDMSEKDDFAGQTCLPVFELR.R;K.DDFAGQTCLPVFELR.R

PFF Mascot score: **[549]** Sequence coverage %: **[22]**

Matched peptides No.: **[8]**

Calculated Mr: **63738** Calculated *p*I: **5.38**

Data base searched result:

Ions score is -10*Log(P), where P is the probability that the observed match is a random event.
Individual ions scores > 30 indicate identity or extensive homology (p<0.05).
Protein scores are derived from ions scores as a non-probabilistic basis for ranking protein hits.

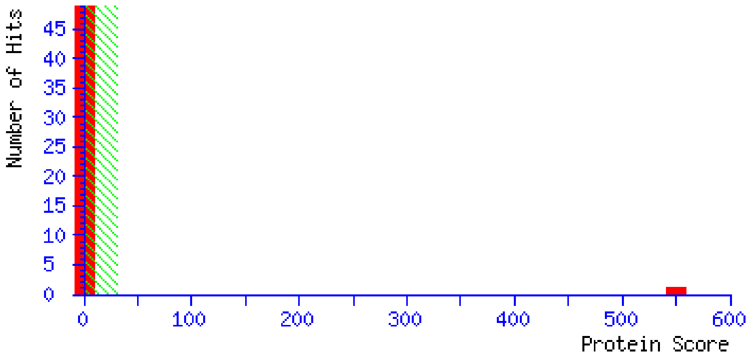

Matched peptide sequences: shown in Bold Red

```
  1 MAVAVAPEEI KALFERYSDN GIMTIDHLRR FLVEVQKEDK ATTGDAQDIF
 51 YHLHELQLGL NFEAFFKYLF GDINPPLDLK RGVHHDMTAP LSHYFIFTGH
101 NSYLTGNQLS SDCSDVPIID ALQSGVRVIE LDIWPNSNKD NVEVLHGRTL
151 TAPVELIKCL RSIQEHAFTA SDYPVIITLE DHLTPDLQAK VAEMITQTFG
201 DILFSPGSEC LKEFPSPEFL KKRIIISTKP PKEYLEAKQT KHRDSEEAYG
251 KETSDLKGHI QAGDKNDLDE DSDDEEDADE GIMRSTRNVV PEYKQLIAIH
301 AGKPKGGLEE CLKVDPEKVR RLSLSEPQLE KAAETYGKEI VRFSQRNILR
351 VYPKGIRINS SNYNPLIGWT HGAQMVAFNM QGHGKSLWLM RGMFRANGGC
401 GYVKKPDFLL KSSPHGDVFN PRVELPVKTI LKVKVYMGEG WFNDFQGKSF
451 DAFSSPDFYV RVGIAGVPAD TVMKETETQM NNWMPVWNEE FKFPLTVPEL
501 ALLRIEVYDY DMSEKDDFAG QTCLPVFELR RGIRAVPLYN FDGVKYNSIK
551 LLMQFDFI
```

Matched peptide information:

Start - End	Observed	Mr(expt)	Mr(calc)	ppm	Miss	Sequence
128 - 148	2447.2913	2446.2840	2446.2605	10	1	R.VIELDIWPNSNKDNVEVLHGR.T (Ions score 102)
162 - 190	3252.6538	3251.6465	3251.6350	4	0	R.SIQEHAFTASDYPVIITLEDHLTPDLQAK.V (Ions score 73)
412 - 422	1212.5942	1211.5870	1211.5683	15	0	K.SSPHGDVFNPR.V (Ions score 93)
435 - 448	1677.7589	1676.7516	1676.7293	13	0	K.VYMGEGWFNDFQGK.S (Ions score 58)
449 - 461	1537.7221	1536.7148	1536.6885	17	0	K.SFDAFSSPDFYVR.V (Ions score 81)
493 - 504	1368.8463	1367.8390	1367.8177	16	0	K.FPLTVPELALLR.I (Ions score 109)
505 - 530	3140.4363	3139.4290	3139.4155	4	1	R.IEVYDYDMSEKDDFAGQTCLPVFELR.R (Ions score 168)
516 - 530	1767.8619	1766.8547	1766.8298	14	0	K.DDFAGQTCLPVFELR.R (Ions score 75)

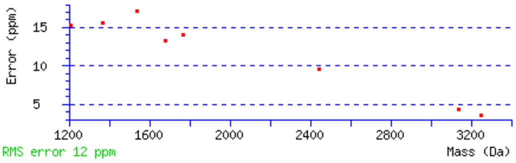

Supplement: FIGURE S2 — Detail information for MS identification of DEPs in rubber latex. [file Presentation_2.PDF]
