# Supplementary material for: Physiological and Proteomic Analyses of Molybdenum- and Ethylene-Responsive Mechanisms in Rubber Latex
Source: Front Plant Sci. 2018 May 15;9:621. doi: 10.3389/fpls.2018.00621 (PMC5962772; doi:10.3389/fpls.2018.00621)
Supplement: FIGURE 3 — All of the identified 169 DEPs from the latex treated with Eth, Mo, and EMo were performed venn diagram analysis (A). Then, the upregulated (B) and downregulated (C) proteins were classified to show their changed patterns after different treatments. [file Presentation_3.PDF]

Supplementary Figure S3

Venn diagram analysis of the finally identified DEPs in latex after Eth, Mo and EMo treatments

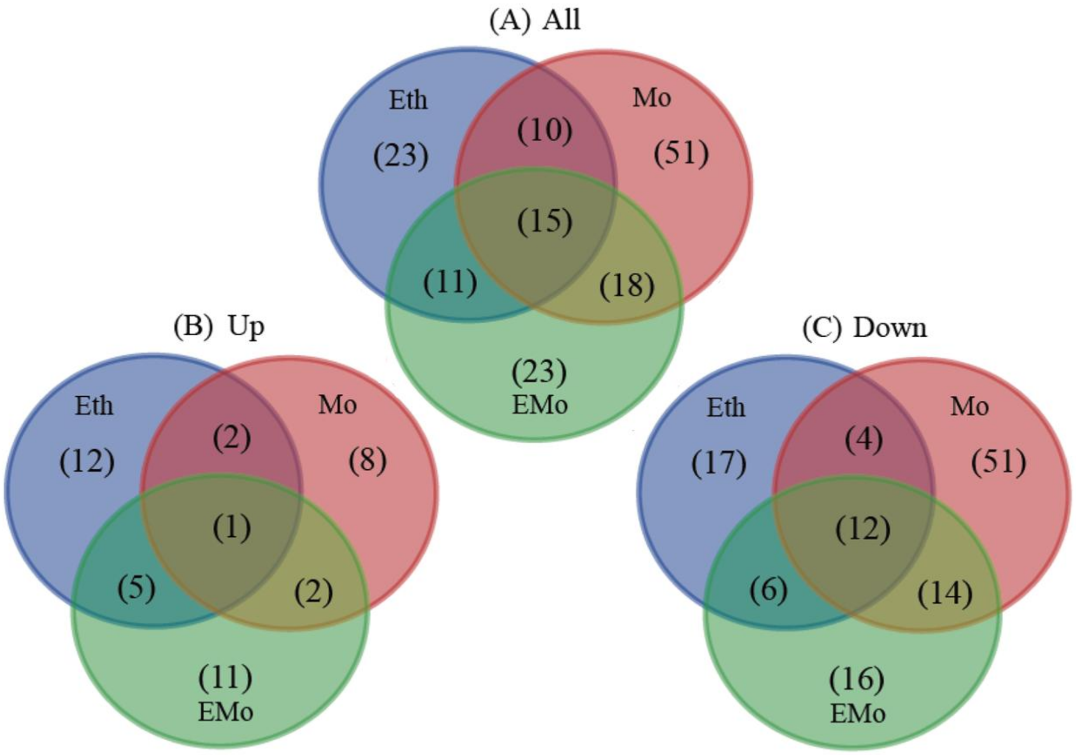

All of the identified 169 DEPs from the latex treated with Eth, Mo and EMo were performed venn diagram analysis (A). Then, the up (B) and down (C) regulated proteins were classified to show their changed patterns after different treatments.
